# Supplementary material for: A randomized trial: The safety, pharmacokinetics and preliminary pharmacodynamics of ropivacaine oil delivery depot in healthy subjects
Source: PLoS One. 2023 Sep 19;18(9):e0291793. doi: 10.1371/journal.pone.0291793 (PMC10508611; doi:10.1371/journal.pone.0291793)
Supplement: S1 File — (PDF) [file pone.0291793.s002.pdf]

Appendix：Clinical study protocol

Title: A randomized, single-blind, positive-controlled phase I clinical trial of long-acting ropivacaine injection: the safety, tolerability, pharmacokinetics, and pharmacodynamics of a single subcutaneous administration in healthy subjects

Protocol No: LB-RSRI-I-AICOMER;
Clinical trial stage: Phase I;
Clinical sites: Beijing Youan Hospital, Capital Medical University;
Applicant: Xi 'an Libang Pharmaceutical Co. LTD;
Principal Investigator: Dr. Ping Chi and Dr. Mei-xia Wang;
Project ID: CR Ropivacaine Injection;
Version number: V version 1.6;
Clinical trial Approval Number: 2017L00490.

Research background

In 1992, Astrazeneca developed a new amide topical anesthetic, ropivacaine. It mainly works by blocking sodium ion entering the nerve fiber membrane and causing reversible block of impulses along the nerve fiber. In 1996,opivacaine was first marketed in the Netherlands, and in 1998, ropivacaine entered the chinese market. At present, the preparation of clinical use is ropivacaine salt (mesylate or hydrochloric acid) aqueous solution.

Local anesthetics may have a similar effect on excitable cell membranes such as brain cells and cardiomyocytes, and if excessive amounts of the drug rapidly enter the systemic circulation, the central nervous system and cardiovascular system will show signs and symptoms of toxicity. Intravenous ropivacaine was well tolerated in healthy volunteers, and clinical application suggested that ropivacaine had a good safety range. Indirect cardiovascular effects (hypotension, bradycardia) may occur with epidural use of ropivacaine, depending on the degree of parasympathetic block. Within the safe dose range, the effective duration of nerve block analgesia with infiltration of ropivacaine at the surgical site after surgery was only 8h, while the peak of postoperative pain in clinical patients was the first 3 days after surgery. The efficacy of ordinary ropivacaine aqueous solution was far from meeting the analgesic requirements of patients. Long-acting ropivacaine injection was ropivacaine free alkali oil solution, has the characteristics of drug loadings and slow release. In animal experiments, compared with the aqueous solution of ropivacaine salt, the dosage of long-acting ropivacaine injection was increased 3.4 times, the efficacy increased about 3-6 times. the result suggestted that the peak time would cover clinical postoperative pain.

Summary of preclinical studies:

Efficacy:

① **Effect of long-acting ropivacaine injection on postoperative pain in rats:** when the dosage was 12mg/ mouse, the analgesia of ropivacaine hydrochloride injection lasted for 4h, and 50% of animals died, 50% of animals responded to general anesthesia, which recovered within 2h; The analgesic duration of long-acting ropivacaine injection was 10h. ② **The blocking effect of long-acting ropivacaine injection on nerve conduction in anesthetized rabbits:** In ropivacaine mesylate injection group, local nerve conduction was almost completely inhibited about 5min after administration, and the effect lasted for 2~3h; Long-acting ropivacaine injection group 1(0.89mg/ rat), normal saline group and blank solvent group had no effect. Long-acting

ropivacaine injection group 2(3mg/ rat) had a slower effect, and the inhibition duration of nerve impulse conduction was 12-16h. Compared with ropivacaine mesylate group, the sensory nerve block time was 6-7 times longer when the dose of long-acting ropivacaine injection was increased by 3.4 times, and the long-acting ropivacaine injection had a good sustained release effect. ③**The effects of long-acting ropivaine injection on the duration and latency of epidural anesthesia were as follows:** The duration of lower limb paralysis of ropivaine mesylate injection in control group was 4.6, 14.6 and 29.5min when rats were given equal doses (60, 180 and 600 μ g/ rat); The duration of lower limb paralysis in long-acting ropivacaine injection group was 15.4, 53.3 and 237.2min, respectively. Compared with the control group, the duration of epidural anesthesia in the long-acting ropivacaine injection group was 3.3, 3.6 and 8.1 times, and the long-acting ropivacaine injection significantly prolonged the effect of epidural anesthesia.

Safety:

The drug of long-acting ropivacaine injection adopts the free base form of ropivacaine, and the solvent system includes benzyl alcohol, benzyl benzoate, and soybean oil. Both the drug and excipients have a history of clinical injection, so their genetic and reproductive toxicity evaluation is safe. The clinical design dose of long-acting ropivacaine injection, whether as the main drug or as an excipient, exceeds the current clinical dose. Therefore, the key points of safety evaluation of long-acting ropivacaine injection are as follows: The acute toxicity of long-acting ropivacaine injection was evaluated by rats and dogs, and the indexes of blood, biochemistry and pathological changes of main organs were investigated. TK test was carried out at the same time. The experimental results showed that the safety of ropivacaine injection was very good under the current designed dosage conditions.

The evaluation results of irritation and wound healing showed that long-acting ropivacaine injection had recoverable local irritation and had no effect on wound healing, and the evaluation results of allergic reaction were good. Compared with ropivacaine hydrochloride injection, PK test results showed that long-acting ropivacaine injection could significantly reduce T_{max} and prolong MRT_{last} in vivo after equal doses injection, and reduced the cardiovascular and neurotoxicity caused by rapid release of ropivacaine solution. In conclusion, compared with ropivacaine hydrochloride injection, long-acting ropivacaine injection has a good safety guarantee and can fully meet the clinical requirements.

Subject (Ethical requirements and informed consent)

Ethics Committee:

Before the study begins, the study protocol and the informed consent of the subjects shall be submitted to the ethics Committee of the research unit for approval. The ethics committee shall hand over the approved informed consent to the investigator. Researchers should submit copies of the documents approved by the ERC and the membership and qualifications of the ERC to the sponsor. If there is any revision of the plan and informed consent, it also needs to be approved again by the ethics committee. Any serious adverse events that occur during the study should be reported by the investigator to the ethics committee. The ethics committee should be notified at the end of the study.

Informed consent:

Informed consent shall comply with the requirements of the latest version of the declaration of helsinki and the rules and guidelines formulated by the National Medical Products Administration of China. Subjects should be informed of the nature of the investigational drug, the purpose of the study, the possible benefits, and the possible risks before being screened. The investigator should explain the process and possible problems to the subjects clearly, and the subjects should sign and date the informed consent form to indicate their consent. Subject's verbal

consent, signed and dated by the witness, is obtained when a third party witness is permitted under special circumstances. The subject or his or her legal representative will retain one signed informed consent form and another signed informed consent form will be retained in the clinical investigator's study record.

Inclusion criteria

1.The voluntarily participate in the test and sign the informed consent approved by the ethics committee, and fully understand the purpose and requirements of the test.

2.Chinese adult males and females aged 18-50 (including 18 and 50), both male and female;

3.Body mass index (BMI) of 19.0-26.0 kg/m2 (including the critical value); BMI= weight (kg)/height 2 (m2);

Subject exclusion criteria:

1. Subjects are allergic to the test drug or any component of the test drug or similar drugs, or have allergic constitution;
2. Vital signs examination, physical examination, clinical laboratory examination (blood routine test, urine routine test, blood biochemical test, serological test, etc.), 12-lead electrocardiogram, and the results are abnormal and clinically significant as judged by the researcher;
3. Subjects with a history of chronic or serious diseases such as liver, kidney, respiratory, blood or lymph, endocrine, immune (including HIV positive or other immune deficiency diseases), mental or gastrointestinal system, or surgery or disease that may affect drug absorption, distribution, metabolism or excretion;
4. Subjects have been assessed to be at risk of potentially difficult airway; Patients with past or current respiratory diseases such as bronchial asthma, chronic obstructive pulmonary disease, sleep apnea syndrome;
5. Subjects with past or current cardiovascular and cerebrovascular diseases such as postural hypotension, arrhythmia, hypertension and epilepsy;
6. Subjects with long QT syndrome or family history (grandparents, parents and siblings), or QTc interval > 450 ms; Indoor or left and right bundle branch block and/or QRS > 120ms; Ventricular ectopic beats were frequent (⩾ 1 premature ventricular beats occurred in ECG every 10s during screening period); Or abnormal resting heart rate (> 100 BPM);
7. Patients with history of anesthesia accidents;
8. Porphyria;
9. Patients who cannot tolerate venipuncture blood collection;
10. Scar constitution;

11 History of malnutrition or hypovolemic shock;

12. Subjects who have used any prescription drugs, non-prescription drugs, health care products, Chinese herbal medicine or Proprietary Chinese medicine within 2 weeks prior to screening;
13. Subjects used of sedatives, sleeping pills or other addictive drugs or positive results of drug abuse screening;
14. Subjects (including men) had fertility, sperm donation, egg donation plans or did not agree to use effective non-drug contraceptive methods during the study period and for 3 months after the study period;
15. Women of childbearing age whose blood pregnancy test results are higher than the normal range or those in lactation;
16. Participants who have consumed excessive amounts of tea, coffee and/or caffeinated beverages in the past for a long time (more than 8 cups per day, 1 cup =250mL), or cannot give up drinking tea, coffee and other beverages during the study period;

17. Smoked more than 5 cigarettes a day in the last 3 months or could not give up smoking throughout the study period;
18. History of alcohol abuse in the 12 months prior to screening (i.e., more than 28 standard units per week for men and 21 standard units per week for women; 1 unit is equivalent to 285mL of beer, 25 mL of spirits, or 150 mL of wine), or those who drank regularly (more than 14 standard units per week) in the 6 months prior to screening, or who tested positive for alcohol breath, or who could not give up drinking throughout the study period;
19. Subjects had donated blood or lost blood ≥400mL within 3 months prior to screening, or who planned to donate blood during the study period and within 3 months after the study;
20. Subjects had participated in clinical trials of other drugs within 3 months prior to screening, or plan to participate in clinical trials of other drugs during the study period;
21. Subjects are expected to require surgery or hospitalization during the study period;
22. The researcher considered that the subjects with abnormal pain sensation were not suitable for the study;
Other circumstances that the investigator considers inappropriate for participation in this study.

Research methods
The main objectives of the study were as follows:
1.To investigate the safety and tolerability of long-acting ropivacaine injection in healthy subjects in a single subcutaneous injection of different doses;
2.To investigate the pharmacokinetics of long-acting ropivacaine injection during a single subcutaneous administration in healthy subjects;
3.The recommended single-point subcutaneous dose of long-acting ropivacaine injection was investigated.
Secondary purpose:
1.To investigate the efficacy of single subcutaneous administration of long-acting ropivacaine injection in healthy subjects;
2.The analgesic onset time, duration and analgesic intensity of long-acting ropivacaine injection were investigated to determine the optimal dose spacing.
Study design:
A randomized, single-blind, positive-controlled, single-dose subcutaneous, dose-increasing clinical study based on the characteristics of laparoscopic surgery.
Test drug: Long-acting ropivacaine injection Specification: 200mg/10mL; Manufacturer: Xi 'an Libang Pharmaceutical Co., LTD. Period of validity: 24 months;
Control drug: Ropivacaine hydrochloride injection, specification: 100mg/10mL; Manufacturer: AstraZeneca AB; Validity: 36 months.
Number of planned subjects: This study plans to enroll 50 healthy subjects, with unlimited male and female ratio. Whole blood and blank plasma samples were collected from 6 healthy subjects. An exploratory trial was conducted to investigate the optimal single point subcutaneous dose of long-acting ropivacaine injection in 3 healthy subjects. The safety, tolerability, pharmacokinetics, and pharmacodynamics of long-acting ropivacaine injection in a single subcutaneous administration of different doses were investigated in 41 healthy subjects.

Dose design:
The guidelines for estimating the maximum recommended initial dose of drugs for the first clinical trial in healthy adult volunteers issued by the state food and drug administration (2012-05-15) and the relevant guidelines issued

by FDA were referred to. The initial dose was determined to be 150mg/ time. Referring to the pre-clinical pharmacodynamic test results of long-acting ropivacaine injection in SD rats, when the dose of ropivacaine was greater than or equal to 0.4mL (8mg), the pain threshold of rat heat stimulation was the highest at each time point after administration, and there was no significant difference. The estimated effective dose for human body was about 5.2 mg/kg, based on the adult body weight of 60kg. The clinical effective dose is about 300mg/ time. Maximum dose: 400 mg/ time.

Methods for increasing dose:

1. Exploration of optimal subcutaneous dose for single point of long-acting ropivacaine injection. The initial single-point dose of the study included five doses: 0.6mL (12mg), 0.8mL (16mg), 1.0mL (20mg), 1.2mL (24mg) and 1.5mL (30mg), totaling 5.1mL (102mg). Three healthy subjects were given five different doses at different dosing sites in a single dose.

2. Safety, tolerability and pharmacokinetics tests of single subcutaneous administration of long-acting ropivacaine injection at different doses: based on preclinical animal test results, 150 mg was determined as the initial dose for healthy subjects. There are five cohorts from the lowest initial dose to the maximum dose, and the dose escalation scheme is shown in the table below.

Table 1：Dose escalation protocol for single dose trials in healthy subjects

array	Dose（mg）	Number of healthy subjects (n)	
		Experimental group long-acting ropivacaine injection	Control group ropivacaine hydrochloride injection
A	150	6	3
B	230	8	--
C	300	8	--
D	350	8	--
E	400	8	--
Total		38	3

Positive control was set for cohort A, and no positive control was set for other dose groups. The study began at the minimum dose, with each subject receiving only one dose and only one dose cohort at a time. Safety assessments were conducted on days 7 and 21 after dosing, and the next cohort/batch trial was determined based on the results. If no significant adverse effects were observed in the maximum dose cohort (400mg), dose-escalation was not continued.

Trial period:

This study was divided into three stages: screening period, trial period and follow-up period. The screening period was from -14 days before administration to -1 day before administration. Trial period: Trial 1 lasted from day 1 to day 2 of administration, and trial 2 lasted from day 1 to day 4 after administration. The follow-up period was after dosing (trial 1: Day 3; Experiment 2 was conducted from day 5 to day 21, with one follow-up on day 7 and day 21.

Termination criteria:

1.If there is any SAE associated with the investigational drug during the course of the trial, the investigator and sponsor will fully discuss the cause and determine the impact on subsequent trials before deciding whether to continue the trial.

2.According to the Evaluation Criteria for Common Adverse Events (CTCAE) (V5.0, published on November 27, 2017), if ≥1/2 of the subjects in the trial cohort have grade ≥ ii adverse events or ≥1/3 of the subjects have grade ≥ iii adverse events (adverse events may be determined by the investigator to be related to the test drug), the investigator and the sponsor shall discuss. Whether to terminate dose escalation;
3.If no significant adverse effects were observed in the maximum dose cohort (400mg), dose escalation was discontinued.
4.The sponsor requested a complete termination of the test;
5.The National Medical Products Administration or the ethics committee ordered the trial to be terminated for some reason.

Test Method:

Optimal single point subcutaneous dose for long-acting ropivacaine injection:

There were 3 healthy subjects. The six regions of the abdomen of each subject were marked as I, II, III, IV, V and VI clockwise from the xiphoid process.
The initial administration area was determined by computer randomization and marked clockwise as 1, 2, 3, 4 and 5. Five doses of the test drug were given subcutaneous single injection of 0.6mL (12mg), 0.8mL (16mg), 1.0mL (20mg), 1.2mL (24mg) and 1.5mL (30mg). Total 5.1mL (102mg); The sixth point is used as the blank comparison point. The local skin irritation reaction score and analgesic range of the administration site were investigated after subcutaneous injection of different doses of test drugs into the abdomen. At the same time observe the subjects areas of the adverse reactions and different doses of local skin irritation response, including clinical symptoms and signs, laboratory examination, adverse events (including systemic adverse reactions and the injection site appearance, pain or tenderness at the injection site, the injection site erythema or redness, injection site induration or swelling, itchy at the injection site, etc.).
The optimal single-point dose (L) and the average diameter (D) of the analgesic range of the dose were determined.
Only one dose was given at each injection site (from lowest dose to highest dose);
Only one dose was administered at each injection site;
Selection method of abdominal injection point: Through umbilical horizontal line, and on the left and right iliac spine to the belly line on the halfway point of the attachment for the two vertical line abdomen can be divided into six areas (figure 1), starting from xiphoid process parts respectively clockwise as I, II, III, IV, V, VI, freely choosing a point for each area injection points, the distance between any two points not less than 5 cm;
Local skin irritant reactions at the injection site: Local skin irritant reactions were observed at the injection site after administration, and implement the next administration or terminate the next dose after evaluation (the interval between two injections should be ≥20min) :
(1) When skin irritation is less than or equal to 2, the next dose can be injected;
(2) When the skin irritation reaction was greater than or equal to 3 minutes, observe it once every 10 minutes until the skin irritation reaction was less than or equal to 2 minutes, record the time, and continue the next dose of injection;
(3) Skin irritation reaction ≥3 points, and no relief was observed for 2 hours, the next dose of injection was terminated, and observation or follow-up was performed until the reaction disappeared; Evaluation of local skin irritation reaction at the injection site: During the test, local skin irritation reaction at the injection site was evaluated according to the skin irritation scoring system [30], which was scored 20min, 40min, 1h, 2h,

6h, and 24h after each administration. Local skin irritation reactions were determined and recorded according to the Evaluation Criteria for Common Adverse Events (CTCAE) (V5.0, published on November 27, 2017), including pain or tenderness at the injection site, erythema or redness at the injection site, induration or swelling at the injection site, and itching at the injection site. Subjects were hospitalized for observation for at least 12 hours after single dose.

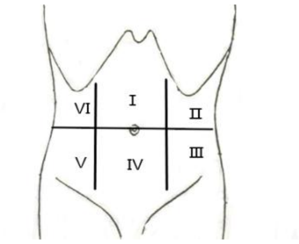

Figure 1: Schematic diagram of six zones of abdomen

Safety, tolerability and pharmacokinetics of a single subcutaneous dose of long-acting Ropivacaine injection in healthy subjects:

Each subject, on the basis of laparoscopic surgery in the abdominal area, according to the abdomen four partition method (i.e. through umbilical in vertical and horizontal stroke, the two lines intersect the abdomen is divided into four areas, as shown in figure 2) with four areas, from the xiphoid process position clockwise ordinal annotation for Ⅰ, Ⅱ, Ⅲ, Ⅳ, random initial dosing area is determined, and in turn clockwise with dosing area for 1, 2, 3, The last area is the blank control area.

Any one point was chose in each area as the center of the circle (the distance between any two centers is not less than 80mm) and mark a circle with a diameter of 40mm.

The number of injection points in each queue was calculated by 1.2ml/needle. That is, the number of injection points in each queue = the dose of the queue /1.2, the number of injection points on each circle = the number of injection points in each queue /3, and the number of injection points on the first and second circles was determined by integer, and the remaining dose was allocated to the third circle.

Injection points on each circumference shall be evenly distributed; The labeled injection points were 1a, 1b, 1c. 2a,2b,2c ; 3a, 3b, 3c (the serial number of injection points on each circle should be ordered clockwise from the top of the circle, that is, from the head direction);

Mark the pain test area: mark the point on circle 1 or 2 where the center of the circle (1/2) is 1cm(A), 3cm(B) and 4cm(C) away from the center as the pain test point;

Each acupuncture measuring point was taken as the center of the circle, and a circle with a diameter of about 1cm was marked as the measurement area of acupuncture pain. The pain test areas were recorded as center 1 and center 2 respectively. 1aA,1bA,1cA ; 2aB,2bB,2cB ; 3aC,3bC,3cC ;

Acupuncture pain intensity was measured only on circle 1 and circle 2.

The test drug (test preparation or control preparation) shall be injected subcutaneously at a position enantiograms on the circumference; Observation of local skin irritation reaction at the injection site: The evaluation of local skin irritation reaction at the injection site was evaluated according to the skin irritation scoring system, and the scores were performed before and after administration at 20min,40min,1h,2h,6h,24h 48h 72h, as shown in the skin irritation reaction rating table.

Local skin irritation reactions, including pain or tenderness at the injection site, erythema or redness at the injection site, induration at the injection site, swelling at the injection site, itching at the injection site, etc., were determined and recorded according to the evaluation criteria for Common adverse events (CTCAE) (V5.0,

published on November 27,2017).
The onset time of analgesic maintenance time and the intensity of acupuncture pain were measured.
The linear distance between two adjacent injection sites on the same circumference was measured and recorded.
The drug administration investigator and the pain measurement investigator should not be the same person;
Subjects should be hospitalized for observation for 72 h after administration before discharge

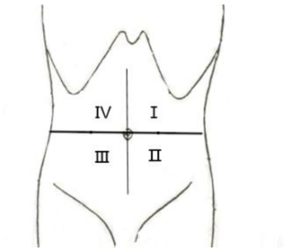

Figure 2: Schematic diagram of four abdominal regions

Pharmacokinetics

Drug dose: there were five cohorts , or five dose groups: 150 mg, 230mg, 300mg, 350mg and 400 mg.

Time of blood sample collection: According to the guiding principles of human pharmacokinetic research, and combined with the characteristics of long-acting ropivacaine injection, the preliminarily planned time of blood sample collection from healthy subjects was shown in **Table 2** The blood collection point of the subsequent cohort can be adjusted according to the actual drug time curve obtained from the low-dose cohort of healthy subjects.

Table 2 Blood sample collection time for pharmacokinetic studies

No	Blood collection time point	Time window
1	Within 15 min before administration	/
2	0.5h after the end of administration	±2 min
3	1 h	±2 min
4	1.5 h	±2 min
5	2 h	±2 min
6	4 h	±5 min
7	6 h	±5 min
8	8 h	±5 min
9	10 h	±5 min
10	12 h	±5 min
11	14 h	±5 min
12	21h	±5 min
13	24 h	±5 min
14	27 h	±5 min
15	30 h	±5 min
16	33h	±5 min
17	36 h	±5 min
18	48h	±5 min
19	72 h	±5 min

Biological sample processing procedures

Identification of biological samples: Before blood sample collection, the blood vessel collection and plasma sample cryopreservation tubes should be numbered uniformly, and special labels should be pasted to identify the words: The first item in the label is the hospital project number; Number of the plan of conduct; The third line #### is the subject number, XX is the blood collection site number; The fourth behavior sample type: whole blood is used for vacuum vascular collection, plasma is used for cryopreservation tube, A is plasma detection, B is backup plasma; **min/h represents the blood collection point of the subject.

PK sample collection: 4mL of venous whole blood was taken into a blood collection tube containing EDTA-K2 anticoagulant. Immediately, the test tube was gently reversed 4-5 times after blood collection to ensure that the blood and anticoagulant were fully mixed, and to avoid prolonged contact between the blood sample and rubber plug. Centrifugation was completed within 1h after collection (4℃ , 2000g, 10min), the upper plasma was divided into two cryopreservation tubes (specification :2mL)(test plasma A and backup plasma B), and the plasma samples were transferred to the ultra-low temperature refrigerator (-60 ~-90) within 2h(calculated from the blood sample collection) for biological analysis.

PK biological sample transfer: After sample collection of the whole test, the plasma will be transported by cold chain company in a sample transport box at -60℃~-90℃ to the testing company for plasma concentration determination.. Temperature during transportation is monitored in real time and biological samples cannot be reversed..

Determination of drug concentration in plasma: The concentration of ropivacaine free base in human plasma was determined by LC-MS/MS method. This method strictly complies with relevant requirements for sufficient confirmation, including specificity; matrix effect; standard curve, linear range, lower limit of quantification; intra-batch and inter-batch variation; residual and dilution effects; recovery test; standard, internal standard placement stability test, mother liquid stability test, whole blood placement stability test, freeze-thaw stability test, repeated injection stability test, post-preparation stability test, room temperature placement stability, long-term stability, etc.

Estimation and evaluation of pharmacokinetic parameters: Pharmacokinetic curves and average pharmacokinetic curves of all subjects were drawn according to the drug concentration-time data measured in the trial, and pharmacokinetic parameters (mean and standard deviation) were calculated. The main pharmacokinetic parameters included: Tmax, C_{max}, $AUC_{0\text{-}t}$, $AUC_{0\text{-}\infty}$, Vd, Kel, $T_{1/2}$, CL etc.

Pharmacodynamics methods:

Experimental design, (1) Analgesic range measurement (Experiment 1) : After the single point of administration, pain points were determined in two directions (when the VAS value of the test point (n) 1cm away from the point of administration was ≥4, The measurement can be terminated at (n+1) time point) at 15min, 30min, 45min±3min, 1h, 1.5h, 2h, 3h, 4h, 5h, 6h, 7h, 8h, 9h, 10h, 11h, 12h ±5min, respectively. The distance between pain point and drug administration point (R1, R2) was measured and recorded, and the analgesic range diameter D = (R1 + R2).

(2) Analgesic maintenance time, onset time and acupuncture pain intensity were measured (Test 2): the intensity of acupuncture pain was measured before and after 15min, 1h, 4h, 8h, 10h, 12h, 14h, 18h, 21h, 24h, 27h, 30h, 33h, 36h, 48h, 72h;

Baseline pain measurement: Before drug administration, visual analogue scale (VAS) was used to measure the pain pressure baseline value (g) of VAS=4,5,6 in the pain test area, repeated for 3 times, and average value was taken respectively, namely the baseline value;

Acupuncture pain intensity measurement: a baseline value of VAS=4 was applied to the blank area to remind subjects of the sensation; In the pain test area, the baseline VAS=4 pressure was applied to the area, and the subjects were asked to compare the feeling with the blank area, and the feeling was "weaker", "similar", "stronger"?

When it was "weaker", baseline pressure at VAS= 5,6 was successively given and the inquiry process was repeated. When VAS=6 pressure was applied, subjects felt weaker and did not continue to increase the pressure. When it was "similar", record the maximum pressure value applied at this time; When it was stronger, the maximum pressure of the previous application was recorded and the onset time of analgesia maintenance time and the intensity of acupuncture pain were counted.

Observation indicators:

The main indicators include:

(1) the safety and tolerability experiment observation indexes: including clinical symptoms and signs laboratory tests local skin irritation at the injection site reactions assessment of adverse events (including systemic adverse reactions, and the injection site appearance at the injection site pain, tenderness at the injection site erythema or redness at the injection site induration , swelling At the injection site itching, etc.);

(2) The observation indexes of single point optimal subcutaneous dose exploration test included skin irritation score at injection site and average diameter of analgesic range (Test 1);

(3) Pharmacokinetic test detection: pharmacokinetic parameters of ropivacaine concentration in plasma.

Secondary indicators: Pharmacodynamic test observation indicators: The analgesic effect duration, acupuncture pain intensity and analgesic onset time of the two drugs in experimental group and positive control group were observed and compared. Duration of analgesia; Analgesic onset time; Acupuncture pain intensity; Analgesic range of injection site (study 1); The straight-line distance between two adjacent dosing points on the same circumference.

Pharmacokinetics: Experiment 2 was conducted to investigate pharmacokinetics. Blood concentration-time data and pharmacokinetic parameters of single dose were investigated. Combined with the characteristics of ropivacaine injection, and referring to the clinical pharmacokinetic test report of experimental drug in beagle dogs, the blood sample collection time of healthy subjects was preliminarily determined as follows: Within 15min before administration and 0.5h, 1h, 1.5h, 2h, 4h, 6h, 8h, 10h, 12h, 14h, 21h, 24h, 27h, 30h, 33h, 36h, 48h and 72h after administration. The concentration of ropivacaine in human plasma was determined by LC-MS/MS. The pharmacokinetic parameters were calculated by drawing the pharmacokinetic curves and average pharmacokinetic curves of each subject according to the concentration time data of each subject measured in the experiment.

Pharmacodynamics:

(1) Maintenance time of analgesia, onset time and intensity of acupuncture pain were measured: the intensity of acupuncture pain was measured before and after administration at 15min, 1h, 4h, 8h, 10h, 12h, 14h, 21h, 24h, 27h, 30h, 33h, 36h, 48h and 72h.

(2) Analgesic range measurement: 15min, 30min, 45min, 1h, 1.5h, 2h, 3h, 4h, 5h, 6h, 7h, 8h, 9h, 10h, 11h, and 12h after single point administration, analgesic range was measured in two directions (study 1).

Pharmacodynamics index evaluation

Analgesic onset time:

Evaluation of analgesic onset time: analgesic onset time was recorded as the time when the threshold of measured pain sensation was greater than the threshold of baseline pain sensation of the subject (i.e., baseline

VAS=4 average acupuncture pressure) when the pain sensation was measured after drug administration.

Pain threshold: the minimum stimulus intensity when the human body feels pain. Pain threshold usually refers to a stimulus intensity that can cause pain sensation in human body, which is generally divided into pain sensation threshold and pain tolerance threshold (the maximum stimulus intensity that human body can tolerate) .

Duration of analgesia: Effective analgesia refers to the pain threshold greater than the baseline pain threshold during pain measurement. Theoretically, the duration of analgesia is the difference between the effective start time and the effective end time of analgesia. However, in the actual operation of this study, real-time dynamic monitoring could not be achieved, so the duration of analgesia in this study was divided into minimum duration of analgesia and maximum duration of analgesia. The true value of analgesic duration was between the minimum duration and the maximum duration.

Minimum duration of analgesia = last time from measurement to effective analgesia - first time from measurement to effective analgesia

Maximum duration of analgesia = (last measured to effective analgesia +1) measurement time - first measured to effective analgesia time

Table 3： Acupuncture pain intensity: The intensity of acupuncture pain in this study was divided into 3 levels as described in the table below:

Three Grade	Pain threshold was measured ≥ baseline VAS=6 acupuncture pressure
Second Grade	Baseline VAS=6 acupuncture pressure > threshold of pain sensation ≥ baseline VAS=5 acupuncture pressure
First Grade	Baseline VAS=5 acupuncture pressure value > threshold of pain sensation ≥ baseline VAS=4 acupuncture pressure value

Analgesic range of injection site (Study 1);

The straight-line distance between two adjacent dosing points on the same circumference.

Study preparation (protocols and drugs)

Study protocol revision: The study will undergo necessary protocol revision based on the clinical trial data obtained. The revised study protocol must be approved by the sponsor, investigator, and ethics committee.

Production and storage of drugs: Investigational drugs and control drugs should meet the requirements of GCP, be produced under conditions that meet the requirements of GMP, and must have drug inspection reports.

Preparation of label: The study drug will be packaged in a pre-labeled cartridge for use by subjects. The label will also be attached to the smallest package of the drug when it leaves the factory. The name of the drug, drug number, specification, usage, storage, expiration date and manufacturer are listed on the label of each cartridge, and the label is marked "for clinical research only".

Drug number: Drug number in the form of T#*** or Z#***, where T stands for experimental drug and Z stands for control drug; # is X or Y, where X represents test 1 and Y represents test 2; *** is a 3-digit serial number, starting from 001 to the desired number. Drug delivery, distribution and inventory: The investigational drug shall be transported to the study center in accordance with the standard operating procedures of Xi 'an Libon Pharmaceutical Co., LTD. Experimental drugs will be stored according to label instructions. The investigator shall be responsible for dispensing and inventorying test drugs. The investigational drug is to be used only on the subject of the clinical trial, and the dosage and usage of the investigational drug shall be in accordance with the protocol, and the remaining intact investigational drug package shall be returned to the sponsor and shall not be

transferred to any non-clinical trial participant.

Administration plan and study period: This product is given subcutaneously in a single dose, the injection site is abdomen subcutaneously. This product cannot be injected intravenously or intramuscularly. On the day of administration, the corresponding dose of the test drug was injected subcutaneously through the abdomen in a single multi-point injection according to the dose cohort to which the subject belonged.

Research procedures

Screening: The benefits and risks of the trial should be explained in detail to the subjects. Prior to screening evaluation, subjects should be informed and sign informed consent. After signing the informed consent, each subject will undergo screening examination and can be enrolled after meeting the inclusion criteria through various examinations.

Enrollment: Subjects who meet the screening criteria for enrollment will be enrolled in this study.

Randomization

① **Optimal single point subcutaneous dose test of long-acting ropivacaine injection (study 1) :** Six areas were selected in the abdomen of the subject abdominal injection site, starting from the xiphoid region, marked clockwise as I, II, III, IV, V and VI, and the first administration area was randomly determined, the first injection site was marked in the first administration area, and the drug was marked clockwise (the dose increased from small to large). The last area after randomization was the blank control area.

② **Safety, tolerability and pharmacokinetics of long-acting ropivacaine injection in a single subcutaneous administration of different doses in healthy subjects (study2) :** Each subject, according to the abdomen four partition method (that is, through the umbilicus in vertical and horizontal stroke, the two lines intersect the abdomen is divided into four area) with four areas, from the xiphoid process position clockwise ordinal annotation for I, II, III, IV, random initial dosing area is determined, and in turn clockwise in round 1, 2, 3, round 4 final area as blank control area.

Upon signing the informed consent, subjects will be assigned a S+ 3-digit subject screening number, ascending from 001; Eligible subjects in each dose cohort will be assigned a 4-digit subject number upon enrollment. Subject number assignment information for each dose cohort is shown in the following table 4:

Table 4 Subject Numbering table

Queue	Subject Number	dosage（mg）
A	1001～1009	150
B	2001～2008	230
C	3001～3008	300
D	4001～4008	350
E	5001～5008	400

Subject numbers for each dose group will be assigned from the smallest to the largest in accordance with the order of enrollment of the subject. Subject numbers will not be omitted. Cohort A 150mg group was randomly divided into experimental group and control group, and then divided into 3 batches for the test, with 1 case in the first group and 1 case in the positive control group. In the second group, there were 2 cases in experimental group and 1 case in positive control group. In the third group, there were 3 cases in experimental group and 1 case in positive control group. The randomization statistician used SAS software to generate the randomization allocation table of

subjects in Cohort 1001-1009 in batches, according to which drugs were given to the experimental group and control group.
Note : (1) the random grouping of cohort A should follow the blind design, and the subjects should not know their own group; (2) The drug administration researcher and the pain measurement researcher should not be the same person.

Administration: After the subjects were admitted to the clinical trial ward, they were examined for vital signs (including armpit temperature, pulse, decubial blood pressure, etc.) in the morning of the next day (the day of administration) before subcutaneous administration.
Optimal single point subcutaneous dose test of long-acting ropivacaine injection (study 1) : 0.6mL (12mg), 0.8mL (16mg), 1.0mL (20mg), 1.2mL (24mg) and 1.5mL (30mg) were subcutaneously injected according to points 1, 2, 3, 4 and 5 marked on the abdomen of the subjects respectively.
Safety, tolerability and pharmacokinetic of a single subcutaneous dose of long-acting ropivacaine injection in healthy subjects (Experiment 2): Each subject in the same cohort was assigned four areas according to the abdominal area of laparoscopic surgery, and the initial drug administration area was randomly determined according to the four-region abdominal method. Injection sites were marked, and subcutaneous injection was performed successively according to the serial number of injection sites. According to the designed dosing dose for each cohort, the dosing points were evenly distributed on the circumference of circles 1,2 and 3 with diameters of 40mm, and the dose was administered each time until the total dose designed for each cohort was reached. If the number of dosing points is not evenly distributed on three circles, one dosing point is reduced or added on the third circle, but the dosing points in the third circle are still evenly distributed (i.e., the dosing dose is evenly distributed on the circle).

Adverse events (including SAE) : In the event of any perceived symptoms, new or worsening symptoms, subjects should be immediately given appropriate medical assistance. The relevant adverse events shall be handled by referring to the section of "Possible adverse events and rescue measures" in this plan. If they meet the definition, they shall be reported as adverse events or SAE reports. Record the most severe level of adverse events during the trial. All pregnancies detected after administration and their outcomes (spontaneous abortion, selective termination of pregnancy, ectopic pregnancy, normal delivery or congenital malformations, etc.) should be reported in writing. Male subjects should report the pregnancy of their spouse after dosing. Pregnancy itself is not reported as an adverse event. If the pregnancy course/outcome meets SAE criteria, the investigator should report it according to SAE reporting procedures. Pregnancy outcomes should be followed up and recorded even if subjects drop out of the study.
Local skin irritation reaction at the injection site: score local skin irritation reaction at the injection site according to the skin irritation reaction evaluation scale.
Upon withdrawal from the study, the subjects will undergo further examination if there are new or increased outliers from laboratory tests. Results should be recorded in the case report form or in the corresponding section of the eCRF.
All adverse events and SAE present at the time of study termination should be followed up to their baseline remission level or be stable or lost to follow-up, unless, in the opinion of the investigator, remission is not likely due to the subject's own disease. For these cases, the investigator should record them on the medical record.

Drug combination

Drugs not permitted during the study: Any drug is prohibited from the screening period until the completion of the final clinical observation and laboratory examination. Including any prescription drugs, over-the-counter drugs, chinese herbal medicine, special medical use formula food, health care products.

Drugs allowed during the study: In case of emergency, consideration with the safety and health of the subjects, if it is necessary to use other drugs according to the judgment of the researcher, the drugs can be used under the guidance of the researcher, and the sponsor shall be informed of the drug use in a timely manner. All drug combinations and treatments must be recorded on the case report form or at the corresponding location on the eCRF. Include the name of the product (chemical and trade name), dosage, method of administration, purpose, and start and end dates.

Blinded design

Principles of blinded design: Because the packaging and properties of the test drug and the control drug in this study were different, it was impossible to blind the researchers. Therefore, the blind method was adopted in this study to ensure that the subjects did not know which drug they received, so as to avoid the bias caused by the subjective factors of the subjects on the efficacy. The randomized grouping of cohort A in trial 2 of this study should follow the blind design. Subjects should not know their grouping, and the drug administration researcher and pain measurement researcher should not be the same person.

Blind storage: In cohort A of trial 2 of this study, the order of each subject receiving test preparations or control preparations will be determined by a random table, which randomly generated by statistical units using SAS(9.4 or higher version) in 1:1 block groups. The selected block length and random seed number are sealed in the blind as confidential data.

Blind emergency letters: At the same time as the drug was blindfolded, clinical trial research centers should be provided with emergency letters. In case of emergency unblinding, the investigator can obtain the grouping information of the subjects according to the prompt of the emergency letter.

In the emergency letter, the contact information and address of the unit and the date should be reported immediately in case of emergency. If the emergency letter is opened, please indicate who opened it, the date opened, the reason and record it in the electronic medical record report form.

Provisions on opening and reading emergency letters: In the event of an emergency (such as serious adverse reaction) or the participants need to rescue must know receive what kind of processing, subject to consent by the center is a major researcher, emergency letters are opened by researchers. Once the emergency mail is opened, the subject with this number will withdraw from the study, the reason should be recorded in the medical report, and inform the bid and CRA. All emergency correspondence will be recalled at the end of the trial for blind post-trial review.

Safety evaluation

All subjects who received one trial drug will be eligible for safety analysis. Subjects' physical examination results, vital signs, adverse events, and laboratory outliers were summarized. Subjects must be closely monitored for adverse events. The severity of adverse events was graded according to CTCAE (V5.0) standard, and the severity, severity and relationship with the experimental drug should be evaluated.

The investigator was responsible for evaluating the association of all adverse events with the investigational drug. However, the principal investigator may authorize the judgment of other qualified clinicians participating in the study, but is still responsible. The investigator must provide a list of qualified and authorized personnel.

Adverse events

Definition of adverse events

Adverse events refer to all adverse medical events that occur after a subject receives an investigational drug, can show the signs and symptoms of disease or laboratory abnormalities, but does not necessarily have causality with experiment with drugs In the clinical study, adverse events can happen is to accept test after using drugs, any time bad health condition.

Examples of AE include:

1) Exacerbation of pre-existing chronic or intermittent diseases, including increased frequency and/or intensity;

2) New diseases discovered or diagnosed after the trial drug was administered, even though they may have existed before the study began;

3) Physical signs, symptoms or clinical sequelae of suspected interactions;

4) Experimental drugs or combinations of drugs, suspicious signs, symptoms, or clinical sequelae due to drug overdose;

5) Signs and symptoms that are time-dependent with treatment;

Unexpected adverse events

An unintended adverse event is any adverse drug event whose character or severity is inconsistent with the investigator's manual (or the control drug label). The addition of significant information about the nature or severity of known or documented adverse events is also part of the reporting of unintended adverse events. For example, events that are more special or severe than those described in the researcher's manual should be considered "unexpected".

Observation, recording and reporting of adverse events

All adverse events that occurred after the subject received the investigational drug, which will need to be fully documented on the subject's case report form or eCRF.

Description of each adverse event should include its start and end date, whether it conforms to a serious adverse event, measures taken (such as study treatment changes, other treatments and follow-up tests) and outcome, and request the investigator to conduct a causal evaluation (relationship to study treatment). Adverse events should be graded and changes recorded on the corresponding case report form or eCRF. Documentation must be supported by original data.

Methods for detecting adverse events

At each follow-up, adverse events can be detected by:

Unsolicited information provided by the subject or caregiver

At each visit, subjects were asked open-ended, non-leading questions: How are you feeling? Have you had any medical problems since your last visit?

Abnormalities observed by the investigator, other medical staff, and family members.

Abnormalities detected by medical tests and examinations

Other Abnormal conditions

Adverse event collection time

Adverse events were recorded from the time subjects received the investigational drug to 21 days after the drug was administered.

Criteria for judging the severity of adverse events

The severity of adverse events was determined according to CTCAE (V5.0) criteria. In case of unlisted adverse events, the following table can be used for reference:

Table 5：Criteria for determining adverse events

Adverse event classification	Severity description
Grade I：	Mild, with no or mild clinical symptoms; Only clinical or laboratory tests are abnormal; No treatment is required.
Grade II：	Moderate, requiring minimal, local, or non-invasive treatment; Age-appropriate Activities of Daily Living (ADL) using tools are limited, which means cooking, shopping, making phone calls, etc.
Grade III：	Serious or medically serious condition but not life-threatening for the time being; Resulting in hospitalization or prolonged hospitalization; Cause disability; Self care ADL is limited. Daily self-care refers to taking a bath, dressing, undressing, eating, going to the bathroom, taking medicine, etc., and being not bedridden.
Grade IV：	Life-threatening and in need of urgent medical attention.
Grade V：	Death due to adverse events.

Evaluation of association between adverse events and experimental drugs

According to the judgment criteria of causality between drugs and adverse events, the correlation between adverse events and the application of tested drugs was divided into five levels: positively relevant, probably relevant, possibly relevant, possibly irrelevant and definitely irrelevant. The positive, probable and probable adverse drug reactions were classified as adverse drug reactions. Taking the total number of adr cases as the molecule, all selected cases for evaluation of adverse reactions as the denominator, the incidence of adverse reactions was calculated.

① **Positively relevant:** Fits the known pattern of reactions to the drug in question, consistent with a reasonable post-medication chronology, adverse events reduced or disappeared after dosage reduction or withdrawal. This adverse event occurred again after repeated administration..

② **Probably relevant:** Fits the known pattern of reactions to the drug in question, consistent with a reasonable post-medication chronology, adverse events reduced or disappeared after dosage reduction or withdrawal. However, the subject's clinical status or other reasons may also produce this event.

③ **Possibly relevant:** Fits the known pattern of reactions to the drug in question, consistent with a reasonable post-medication chronology, the adverse event was reduced or not significant after dosing reduction or withdrawal, but the clinical status of the subject or other reasons could explain the event.

④ **Possibly irrelevant:** Not very consistent with the known type of reaction of the drug in question, not very consistent with a reasonable time sequence after administration. The subject's clinical status or other causes may also have contributed to this event.

⑤ **Definitely irrelevant:** Does not conform to the known reaction type of the drug in question, does not conform to a reasonable time sequence after administration, the response can also be explained by the subject's clinical status or other causes, and the event abates or disappears when clinical symptoms or other causes are excluded.

Table 6 Criteria for judging causality of adverse events

	Positively relevant	Probably relevant	Possibly relevant	Possibly irrelevant	Definitely irrelevant
Have a reasonable time sequence with the test drug	+	+	+	—	—

Known types of adverse drug reactions	+	+	+	—	—
The reaction relieved or disappeared after drug withdrawal	+	+	±	±	—
Adverse reactions returned after repeated administration	+	?	?	?	—
It can't be explained by the subject's disease	+	+	±	±	—

Note：(1)“+”certainty；“－”negative；“±”It is difficult to say or deny；“?”unclear situation；(2)Definitely related, may be related, can not determine the adverse reaction caused by drugs.

Treatment, follow-up, and duration of cases with adverse events

The investigator shall follow up the subject closely after the initial adverse event report, take appropriate treatment measures if necessary, and provide relevant information to the sponsor. All adverse events during the study, which should be managed according to the clinical trial center SOP, should be followed up to baseline or stable or lost to follow-up.

Determination and treatment of abnormal laboratory test indicators

Abnormalities in laboratory values must first be compared with baseline by the investigator to determine whether they are clinically significant; Adverse events were reported if laboratory abnormalities were judged to be clinically significant and varied from baseline. Non-clinically significant laboratory numerical abnormalities are not reported as adverse events. All abnormal laboratory test values/vital signs should be accurately recorded on the relevant case report form or eCRF.

Possible adverse events and rescue measures

Possible adverse events

According to the clinical experience of similar drugs at home and abroad, the clinical adverse reactions of ropivacaine analogues are very common (≥1/10) : Nausea, hypotension. Common clinical adverse reactions (≥ 1/100) include: elevated body temperature, rigidity, back pain, bradycardia, tachycardia, hypertension, paresthesia, dizziness, headache, vomiting, and urinary retention. Occasionally seen clinical adverse reactions (≥1/1000) include: hypothermia, fainting, anxiety, toxic symptoms of the central nervous system (convulsions, grand mal seizures, epileptic seizures, dizziness, perioral paresthesia, tongue numbness, auditory hypersensitivity, tinnitus, visual impairment, dysarticulation, muscle twitching, tremor), hypesthesia, dyspnea. Rare clinical adverse reactions (<1/1000) include: anaphylaxis, the most serious cases are anaphylactic shock, cardiac arrest, arrhythmia.

Based on existing preclinical studies of the investigational drug, Long-acting ropivacaine injection is mainly subcutaneous, and the solvent is irritating. The main adverse reactions observed in preclinical toxicology tests included: Erythema, induration, ulceration, scab, mild acute/subacute subcutaneous inflammation, mild or mild granulation tissue formation/fibrosis.

The irritant reaction may recover after withdrawal.

Table 7 The main adverse reactions are listed as follows:

Very common (≥1/10)	
the whole body	naupathia
circulatory system	hypotension

Common (1/100) or higher	
the whole body	Elevated temperature, stiffness, back pain
circulatory system	Bradycardia, tachycardia, hypertension
central nervous system	Paresthesia, dizziness, headache
digestive system	emesis
Kidney and urinary system	uroschesis
Occasional (1/1000) or higher	
the whole body	hypothermia
circulatory system	faint
central nervous system	Anxiety, toxic symptoms of the central nervous system (convulsions, grand mal seizures, epileptic seizures, dizziness, perioral paresthesia, tongue numbness, hyperhearing, tinnitus, visual disturbances, dyskinesia, muscle twitches, tremors), hypoesthesia
respiratory system	breathing difficulties
Rare (< 1/1000)	
the whole body	Anaphylaxis, its most severe form anaphylactic shock
circulatory system	Cardiac arrest, arrhythmia

For the above adverse reactions, timely measures should be taken to ensure the safety of long-acting ropivacaine injection in the first phase I clinical study in humans, and the following risk control measures for expected adverse events should be formulated.

Risk management of adverse events

The most common adverse reactions of ropivacaine injection included nausea and hypotension as well as systemic nervous system, circulatory system, respiratory system, kidney and urinary system; Occasional or rare adverse reactions: For the possible adverse reactions of ropivacaine, risk control should be focused on the following aspects in clinical studies.

(1) First, when screening the enrolled subjects, exclude people allergic to similar drugs or belonging to allergic constitution.

(2) Subjects should be fully informed of the possible adverse reactions caused by the treatment plan to reduce their anxiety before medication.

(3) During and within 30 minutes after administration, clinicians with anesthesia experience should be present, and the rest of the observation period in hospital should be closely monitored by professionally trained clinical medical staff.

(4) Ecg monitoring should be carried out when necessary.

(5) In view of the risk of blood injection, the standard method of subcutaneous injection should be strictly followed. Before drug administration, suction should be carefully used to prevent injection into the hemostatic tube, and the injection speed should be controlled and slowly injected.

(6) For vomiting, a common adverse reaction, subjects should eat a light diet during the test.

(7) At the same time of injection, closely observe the patient's vital signs and continue to talk with the patient. If symptoms of poisoning appear, immediately stop the injection.

(8) The test drug can only be opened and used once, and the remaining liquid must be discarded.

(9) Local anesthesia will slightly affect mental status and mutual aid coordination, and temporarily impair

movement and flexibility. Therefore, subjects' free movement is restricted after administration.
(10) Measures and examinations taken to support treatment should be properly kept as original documents. Record and report in strict accordance with relevant SOP.

Treatment and rescue of adverse reactions

(1) The injection must be stopped immediately in case of acute systemic toxicity.
(2) In case of central nervous system toxicity such as convulsion, emergency treatment should be carried out in accordance with professional SOP.
(3) If circulatory system toxicity reactions such as circulatory failure occur, emergency treatment should be carried out in accordance with professional SOP in time.
(4) If cardiac arrest occurs, the resuscitation time may be prolonged in order to improve the success rate of resuscitation.
(5) In case of respiratory toxicity, emergency treatment should be carried out in accordance with professional SOP.
(6) If the needle punctures the blood vessel, remove the needle immediately and press the injection site.
(7) In case of other situations, the researcher shall make corresponding emergency treatment according to the corresponding situation.

Serious adverse event

Definition of serious adverse events

Serious adverse event: Refers to the adverse medical events such as death, life threatening, permanent or severe disability or loss of function, the need for hospitalization or prolonged hospitalization of the subject, and congenital abnormalities or birth defects after the subject receives the test drug.
Death: Irreversible coma or brain death, described as sudden death when the cause of death is cardiac. Death and sudden death are distinct and should not be interchanged.
Life-threatening: In the definition of "severe", the term "life-threatening" means that the patient was in danger of death at the time of the incident/reaction, it does not refer to an event/reaction that may cause death if it is assumed to be more severe in the future.
Hospitalization: Any adverse event that results in a subject's hospitalization or prolonged stay of a hospitalized subject is considered serious unless one of the following exceptions is met:
Spending time in the hospital for no more than 12 hours;
Admission to hospital is pre-planned (i.e. an operation scheduled or elective before an informed consent is signed);
Admission to a hospital, regardless of adverse events (such as being admitted for therapeutic purposes).
Disability: means a person's ability to carry out everyday life is severely impaired.

Suspicious and unexpected severe adverse reactions

Suspected and unexpected severe adverse reactions SUSAR (Suspected Unexpected Serious Adverse Reaction) refers to a suspicious and unexpected serious adverse reaction whose nature and severity of clinical manifestations exceed information already available, such as the investigatory drug investigator's manual, the specification of the marketed drug, or the summary of product characteristics.

Record and report of serious adverse events/pregnancy

Subjects should report any serious adverse events that occur after receiving the investigational drug up to 21 days after completion of the drug.

The procedures for reporting serious adverse events are as follows:

The principal investigator had to fill out a "Serious Adverse Event Report Form" and report it to the sponsor within 24 hours of learning about SAE.

The sponsor promptly evaluates the safety information received, and reports suspicious and unexpected serious adverse reactions to all participating clinical trial researchers, clinical trial institutions and ethics committees, as well as to the drug regulatory authorities and health authorities. It is also communicated to all researchers participating in clinical trials (pharmacovigilance, data management, etc.).

Sponsors may request additional information from researchers to ensure timely and accurate completion of safety reports.

The investigator must use all necessary therapeutic measures to address SAE. Any medications required to manage SAE must be documented on the subject's case report form or in combination with the eCRF.

Each SAE should be followed up to baseline or stable or lost to follow-up, and an updated report submitted to the designated person. A simple laboratory anomaly is not reported by SAE unless the investigator considers that the anomaly meets SAE standards. Laboratory abnormalities should be recorded on the "Laboratory Data" page and periodically checked by the clinical inspector.

Pregnancy: all pregnancy after administration and its outcome (spontaneous abortion, selective termination of pregnancy, ectopic pregnancy, normal delivery or congenital malformation, etc.) should be reported to the ETHICS Committee and the sponsor in writing. Male subjects also need to report their partner's pregnancy after receiving the drug.

Pregnancy itself is not reported as an adverse event or as a serious adverse event. If the pregnancy course/outcome meets SAE criteria, the investigator should report it according to SAE reporting procedures. Pregnancy outcomes should be followed up and recorded even if subjects drop out of the study.

If pregnancy occurs within 21 days of completion of dosing, the investigator should notify the sponsor within 24 hours of the pregnancy.

Data management

CRF design: CRF shall be designed according to the test steps and flow charts stipulated in the scheme. After the preliminary draft is formed, project managers, data and statistics personnel, scheme writers and other project team members shall jointly review the CRF to conform to the scheme and comply with relevant laws and regulations, and the version control process shall be completely recorded.

CRF filling guide: The CRF filling guide provides detailed filling instructions for each page of the case report form and each data point according to the study protocol. Ensure that the clinical trial center has access to the CRF and its completion guidelines prior to subject enrollment, and train clinical trial center staff on the protocol, CRF completion and data submission process, which should be documented.

CRF annotation: Annotation CRF is the annotation of blank CRF, which records the location of each CRF data item as well as the variable names and codes in the database. All data items in CRF need to be annotated. DM review is required.

Database design

The database should be set up according to the data set name, variable name, variable type, and variable length in the annotation CRF, and adhere to the standard database structure and setup as much as possible. After the establishment of the database, the database test shall be carried out, and the database test report shall be issued, which shall be signed and confirmed by the principal of data management.

Permission Allocation

The system administrator creates accounts and grants different permissions for different roles.

eCRF

The researchers shall collect the data of the subjects according to the requirements of the study protocol, and fill in the eCRF with accurate, timely, complete and standardized information according to the original data and the filling guide. Modifications to CRF data must follow standard operating procedures, leaving traces of modifications.

Challenge sending and resolution

The data management department shall list the detailed data verification plan, and the verification plan shall be reviewed by the programmer and the data management department and signed for confirmation if there is no objection. After the data is input into EDC, the system will check the data according to the procedures established in the data verification plan, and the data in doubt will be automatically questioned by the system.

The data that cannot be set for the system to issue questions will be sent through EDC by manual questions. Input personnel or researchers will confirm and answer manual questions and system questions, and modify the wrong data if necessary until the questions are solved. If the answer fails to resolve the query, the data manager and clinical inspector can re-query the data point, and all traces are stored in the EDC database.

Data modification and review

Data entry personnel or researchers can modify the data after verifying the data, and fill in the reason for modification in the system as prompted. The investigator has the authority to review all final data.

Medical coding

History, adverse events, and drug combinations collected during clinical trials should be coded using a standard dictionary. The standard dictionary commonly used is MedDRA, ATC. The encoded dataset should clearly record the dictionary and version used in the encoding.

SAE consistency comparison

All SAE-related data points in the CRF are compared with those in the PV library using the program. Inconsistent data needs to be communicated to PV personnel until there are no discrepancies.

Data review meeting

Before the database locked, the draft data management report and all data lists are collected,

Bidders, researchers, data management and statistical analysts in the blind state under the joint audit data does not solve the problem, and according to the statistical analysis has been made to the clinical trial division and verification report serious adverse events and treatment records, such as data audit report after the meeting to be finalized data management and the crowd partition plan, etc.

Locking and unlocking the database

Database locking is an important milestone in clinical research. The process and time of locking should be clearly documented. Locking is revoking the right to edit the database and logging the withdrawal of the right to edit the data in the document.

If there is any modification after the database locked, an application shall be submitted, which shall be discussed and signed by the sponsor, researcher, input personnel, clinical supervisor and data management personnel, and the reasons for unlocking shall be recorded in detail.

Save the data

Store all collected raw data (such as CRF and electronic data) in a secure location during clinical trials. These original documents are part of the audit path that traces the original data and should be protected as strictly as the electronic audit path records any changes or backups to the database.

Data privacy

Data confidentiality is a basic principle that must be observed in the process of clinical research and development. Institutions involved in drug research and development should establish appropriate procedures to ensure the confidentiality of the database, including establishing and signing confidentiality agreements to regulate the behavior of relevant personnel, and establishing confidentiality systems to prevent the disclosure of the database.

The data set must be encrypted before transmission. The data set and password cannot be transmitted in the same email.

Statistical analysis

Single point optimal subcutaneous dose test: descriptive statistical analysis; Safety and tolerability tests: descriptive statistical analysis; Pharmacokinetic test: calculate the blood concentration-time data of each subject, draw the concentration-time curve, and draw the average blood concentration-time curve. The pharmacokinetic parameters of single dose were calculated.

Pharmacodynamics test: analysis and statistics of the level changes of each indicator before and after medication; The groups were compared using a least square mean of the difference from baseline (with baseline as a covariable) and a 95% confidence interval.

Analysis of the crowd

Safety Set (SS) : Includes the composition of all subjects given at least one trial drug. The security analysis set is used for baseline characterization and security analysis.

PK Concentration Set (PKCS) : includes all subjects receiving the investigative drug with at least one blood Concentration data. The pharmacokinetic concentration analysis set was used for correlation analysis of pharmacokinetic concentration data.

PK Parameter Set (PKPS) : includes all subjects receiving the investigative drug with at least one pharmacokinetic Parameter. Pharmacokinetic parameter analysis set was used for correlation analysis of pharmacokinetic parameter data.

Pharmacodynamic analysis Data Set (PD Set) : includes all enrolled subjects who used the investigational drug at least once and have pharmacodynamic evaluation data after administration. Pharmacodynamic analysis datasets are used for correlation analysis of pharmacodynamic data.

Statistical analysis method

Enrollment and completion status

The number and percentage of subjects who were screened, enrolled, dropped off, and completed the test were calculated, respectively, and the number and percentage of subjects who were summarized for each analysis set according to the different dose groups.

Baseline characteristic analysis

Baseline data included demographic characteristics, laboratory tests, vital signs, and drug combinations, and were aggregated by dose group. The measurement data are described by means, standard deviation, median, maximum and minimum. For counting data, frequency and percentage are used to describe. Baseline indicators were compared between groups using population T test or Wilcoxon rank sum test and Fisher's exact probability method. Baseline analysis was performed based on the security analysis set.

Safety/tolerability analysis

Safety/resistance analysis included adverse events, evaluation of clinical laboratory results, and vital signs. The

safety/tolerability analysis was based on the safety analysis data set.

Adverse events

Any subject receiving the investigational drug should be assessed for safety. The evaluation period shall be from the time of receiving the investigant drug to 21 days after the end of study or administration. AEs or SAEs occurring during this period shall be followed until they return to normal or stable (except for subjects who are dead, lost to follow-up, or have withdrawn their informed consent).

Adverse events were coded using MedDRA.

In this study, the adverse events (TEAE) occurred during treatment were mainly analyzed statistically. The adverse events occurred during the screening period to before the first administration were listed, but the statistical analysis of adverse events was not included.

Adverse events referred to below refer to adverse events occurring during treatment.

The incidence, number and incidence of all adverse events, drug related adverse events, serious adverse events and abruption related adverse events in each group were calculated.

According to systematic organ classification (SOC) and preferred term (PT) classification, the number of adverse events, the number of cases and the incidence of adverse events in the two groups were calculated.

A list of adverse events related to the study drug during treatment, a list of all adverse events, a list of adverse events resulting in shedding, and a list of serious adverse events were listed separately.

Evaluation of clinical laboratory results

Laboratory data will be summarized according to the type of laboratory examination.

The normal range and abnormal meaningful criteria will be referenced in the summary of laboratory data.

Changes compared to baseline will be analyzed using a pre - and post-treatment cross-table.

Vital signs

The mean, standard deviation, median, minimum, and maximum of axillary body temperature, pulse, and decubitus blood pressure (systolic and diastolic blood pressure), as well as changes from baseline, were calculated at each specified follow-up time point.

Pharmacokinetic analysis

C-T linear and semilog plots were drawn using the blood concentration (C) - time (t) data of each subject. At the same time, blood drug concentration at each time point was statistically described, including the number of cases, geometric mean, coefficient of variation, minimum and maximum.

Pharmacokinetic parameters of each subject were calculated, including Tmax, Cmax, AUC0-T, AUC0-∞, Vd, Kel, T1/2, CL and MRT. Statistical description of pharmacokinetic parameters of different dose groups and experimental and control groups, including number of cases, geometric mean, coefficient of variation, geometric coefficient of variation, minimum and maximum, etc.

Pharmacodynamics analysis

Pharmacodynamics indexes were described by mean, standard deviation, maximum, minimum and median, and compared with the basic value in screening period. Paired T test was used to compare the differences before and after treatment in groups.

The groups were compared using a least square mean of the difference from baseline (with baseline as a covariable) and a 95% confidence interval.

The mixed effect model was used to analyze the change of acupuncture pain intensity at different time points after

administration compared with that before administration. The model took the change value of pain intensity at different time points in different administration areas compared with baseline as the dependent variable, and the pain intensity before administration as the covariable. Group (experimental group, control group), administration area/blank area, visit site, and interaction between group and visit site were fixed effects and individual factors were considered.

According to the model, the mean value of change in pain intensity at different time points of administration from baseline and its 95% confidence interval were calculated, as well as the mean value of difference between the experimental group and the control group and its 95% confidence interval.

Similar methods were used to analyze the range of analgesia.

Kaplan-meier method was used to statistically analyze the onset time and maintenance time of analgesia, and the median, Q1, Q3 and 95% confidence interval of the onset time and maintenance time of analgesia were calculated.

Processing of missing data

Processing of missing data from safety, pharmacokinetic and pharmacodynamic analyses was not considered in this study.

Research on management

Inspection: in order to ensure the clinical trial participants' safety and rights guaranteed, test record and report the data is accurate, complete and correct, and ensure the test follow the approved plan and the relevant laws and regulations, the bid will be specified with proper qualifications of clinical arbitrator as main contact between the sponsor and the researchers, in the study of the center for inspection. During the study, the investigator should allow sponsor inspectors or representatives to visit the study facility to review documents related to the study (e.g., subject informed consent, drug inventory, and IRB approval documents).

When the examiner visits, the subject's files will be rigorously reviewed to verify that the information recorded in the case report form or eCRF, especially the key information related to safety, matches the subject's original records.

Monitoring activities should follow standard operating procedures to supervise clinical trials and ensure that clinical trials are carried out according to protocol.

Original data verification: This study requires clinical inspectors authorized by Xi 'an Libang Pharmaceutical Co., Ltd. to directly check the original data for data verification.

The data on the patient's case report form or eCRF and the data on their original medical records are checked.

Inspection and inspection: representatives of Xi 'an Libang Pharmaceutical Co., LTD., regulatory department, ethics committee may inspect or inspect the research center, central laboratory and data management and statistics unit.

Personnel training:

The principal investigator will maintain a record of the study training of all study participants (doctors, nurses, and others). The principal investigator will ensure that all such personnel have been properly trained in relation to the study and that any information relevant to conducting the study has been passed on to the relevant personnel.

Data collection and processing:

The clinical trial investigator or clinical study Coordinator (CRC) should complete the case report form or eCRF accurately, timely, completely, and in a standardized manner according to the case Report Form or eCRF completion guidelines. The completed case report form or eCRF must be signed and dated by the investigator.

Researchers must maintain research records and data, including electronic source data and electronic documents. Each data point must be supported by original documentation in the research center. Any records or documents used as the original information (i.e. the subject's original medical documents) shall be kept securely for the sponsor's inspection and inspection by supervisory authorities.

Subject Confidentiality:

All records relating to the identity of the subject will be kept confidential and will not be made public to the extent permitted by relevant laws and/or regulations. The name of the subject will not be provided to the sponsor.

Only subject numbers and initials will be recorded on the medical report form. If a subject's name or other identifying information appears in any other document (e.g. pathology report), this information must be withheld before a copy of the document is provided to the sponsor. Research reports stored by computer must comply with local data protection laws. If the results of the study are published, the individual identities of the subjects will remain confidential. The investigator will maintain a list to verify the subject's identity.

Subject compliance:

In the recruitment selection stage, detailing the purpose of this experiment, experiment with drugs, research approach, the basic situation of the dosing process, the dosage regimen (such as dosage, dosing method, etc.), frequency and the process of clinical observation, biological samples, to test the potential risk, compensation and compensation, etc., make the subjects were informed, voluntary, increase medication compliance;

Before administration, carefully check the subject number, dose and order of administration;

During drug administration, the status of subjects should be closely observed, the feelings of subjects should be communicated at any time, and the subcutaneous injection operation should be carefully controlled.

After the administration, the changes of subjects' reactions and administration sites should be carefully observed, and appropriate medical measures should be taken if necessary.

Follow up will be conducted in strict accordance with the protocol to confirm the safety of the subjects.

Modification of the scheme:

When the modification of the study protocol affects the study purpose, study design, study execution, subject safety, sample size, possible study interest subjects, and study procedure, etc.,

Amendments to the study proposal must be submitted to the ethics committee and approved by the sponsor, investigator and ethics committee.

Deviation or violation of the scheme：

All requirements specified in the research programme must be strictly implemented.

Any intentional or unintentional deviation from or breach of the pilot protocol and GCP principles can be classified as deviation from the protocol or breach of the protocol.

In case of deviation from the plan discovered by the supervisor during the inspection, the researcher or the supervisor should fill in the deviation plan record, and record the time of discovery, the time and process of the event, the reason and corresponding treatment measures in detail. The researcher should sign and report to the ethics committee and the sponsor.

In the data statistics and summary report, the researcher analyzes and reports the impact of the protocol deviation or violation on the final data and conclusions.

When serious program violations occur, an assessment should be made.

If necessary, the sponsor may terminate the study in advance.

Quality control and assurance:

In order to ensure the quality of the trial, the sponsor and the investigator shall discuss and formulate the clinical study plan and study plan together before the trial officially begins.
Research protocol training and GCP training were conducted for the researchers involved in the trial.
The center must manage the investigational drug use in accordance with the SOP, including receipt, storage, distribution, recovery and destruction (if applicable).
In accordance with THE GCP guidelines, necessary steps should be taken during the design and implementation phases of the trial to ensure that the data collected are accurate, consistent, complete and reliable.
All observed results and abnormal findings in clinical studies should be verified and recorded in a timely manner to ensure the reliability of data.
All instruments, equipment, reagents and standard products used in various examination items in clinical research should have strict quality standards and ensure that they are working under normal conditions.
The researchers entered the information required by the protocol into an electronic pathology report form,
The inspectors verify that the information is complete and accurate,
And guide the staff of the test center to make necessary corrections and supplements.
Pharmaceutical supervisory and administrative departments, institutional review board (IRB)/ethics committee (EC), arbitrator of bidders and/or inspectors may be related to clinical research activities and file systematic inspection, to evaluate whether test in accordance with the requirements of the research plan, SOP and relevant laws and regulations, the test data is timely, true, accurate and complete records. Audits should be performed by personnel not directly involved in the clinical study.

Records and data preservation:
The investigator should keep all relevant documents and data necessary for clinical studies completely, including subjects' original medical records, informed consent, case report forms and eCRF, blood collection records, detailed records of drug distribution, etc., and the retention period should be 5 years after the end of the study according to the principles of GCP in China.
However, such information should be retained for a longer period if required by existing regulations or agreements with sponsors.
The sponsor will notify the investigator in writing when these materials are no longer required to be kept.
At least 6 months prior to the expiry date, the investigator should contact the sponsor to discuss the retention or transfer of the research data.
The ownership of the study plan, informed consent, case report form, eCRF and investigator's manual of this clinical study belongs to Xi 'an Libang Pharmaceutical Co., LTD. The investigator shall not provide the study plan, informed consent, case report form, eCRF and investigator's manual to any third party or handle them by himself without the written consent of the sponsor unless required by China Food and Drug Administration.
The sponsor shall retain the clinical trial data up to five years after the trial drug is approved for marketing.

Whole blood and blank plasma samples were collected for the phase I clinical study of long-acting ropivacaine injection

Collection purpose: The purpose of this trial is to meet the needs of pharmacokinetic biological sample assay development for long-acting ropivacaine. Whole blood and blank plasma were collected from healthy subjects to meet the requirements of method development.
Subject selection:

Population and number of subjects: 6 healthy adult subjects.

Subject number: At screening time, each subject will be identified using the screening number. On day -1 of the study, each eligible subject will be assigned a number (e.g. Blank001) based on the screening criteria.

The research process

Screening period (day -14 ~ -2) :

Physical examination, laboratory examination, and other relevant tests (see blank blood collection procedure sheet) will be performed to determine eligibility/exclusion criteria after subjects sign informed consent at -14 to -2 days prior to administration. At screening time, each subject will be identified using a screening number. The investigator will determine whether subjects are eligible to participate in the study based on inclusion/exclusion criteria.

The subjects shall be admitted to the ward of the drug clinical trial Center before 17:30 p.m. on the 1st day. All subjects shall undergo vital signs, physical examination, drug screening test, alcohol breath test and consultation. The female subjects shall have blood pregnancy examination, and shall not bring any food or beverage. A light diet is required during ward stay.

Collection and processing of blank blood biological samples (Day 1) :

About 100 mL of blank whole blood will be collected from each subject, and the subject can leave the drug clinical trial center after completion.

10mL EDTA-K2 vacuum vessels were used to collect about 100 mL of whole blood from each subject, and each tube was gently reversed and mixed 4-5 times.

Whole blood retention sample:

The last 1 tube (about 10mL) of whole blood retention sample from the last 3 subjects in the sequence of screening number was selected, without centrifugation, and stored in the refrigerator at 2-8℃ within 1 hour.

As soon as possible through the professional cold chain company to the detection unit, the transport of 2-8℃ cold chain preservation, real-time monitoring of the temperature during the transport process, and inform the transport personnel not upside down, violent shaking of the sample, so as to avoid cell breakage.

Blank plasma:

The rest of the blood samples were centrifuged at low temperature (4℃) for 10 min at 2000 g centrifugal force.

The upper plasma was extracted and transferred to the labeled cryopreservation tube (specification: 5mL), and the blank plasma samples were stored in the refrigerator of -60℃~-90℃ until transported to the testing unit.

Identification of blank blood biometric samples

Before blood sample collection, the blood collection tube and plasma sample freezing tube should be numbered uniformly, and special labels should be affixed. The sample label is as follows:

This is the hospital project number<br>LB-RSRI-I-AICOMER<br>Subject Number：Blank001<br>plasma1	This is the hospital project number<br>LB-RSRI-I-AICOMER<br>Subject Number：Blank001<br>plasma1

The number of the first hospital item in the label; The second line is the scheme number; The third row is the subject number; The fourth line is the sample type: The label "whole blood" is used for vacuum extraction,The label "plasma" is used for cryopreservation tube, 1 for tube 1, and so on.

Transfer and storage of biological samples

Whole blood and blank plasma are packaged and shipped by a professional biologic sample transport company, Whole blood was transported in the cold chain condition of 2-8℃, and blank plasma was transported in the freezing condition of -60~-90℃ and sent to the analysis and testing unit in time.

The analysis and testing unit stored the blank plasma in the refrigerator at -70±10℃ for future use.

Research flow Chart:

1. Blank blood collection process table

Table 8: whole blood and blank plasma collection test procedures for long-acting ropivacaine injection in human phase I clinical study

**Visit to describe**	**Screening Period (days)**		**blood specimen collection**
Time (day)	**-14-（-2）**	**-1**	**1**
Informed consent	X		
Demographic data	X		
Past and present medical history[1]	X		
Personal history and family history[2]	X		
health checkup[3]	X	X	X
vital signs[4]	X	X	X
Routine blood[5]	X		
Blood biochemical [6]	X		
serological examination [7]	X		
Blood pregnancy test[8]	X	X	
Routine urine[9]	X		
Alcohol breath test and drug abuse screening[10]		X	
routine 12-leads electrocardiogram conventional -lead ecg[11]	X	X	
排标准 Inclusion and Exclusion Criteria	X	X	
入住 check in		X	
血液样本采集 Blood sample collection			X
adverse event	X	X	X
Leave the room			X

Notes：

1) Subjects' past medical history and current medical history should be obtained, including diseases of digestive system, central nervous system, cardiovascular system, kidney and respiratory system, etc.

2) Personal history should include marital history, smoking history, drinking history, drug abuse history and allergy history; Family history.

3) Physical examination includes head, skin, mucous membranes, superficial lymph nodes, neck, chest, abdomen, spine/limbs. Check twice in screening period and once in D1.

4) Vital signs include armpit temperature, supine blood pressure, pulse, respiration, and oxygen saturation.

5) Routine blood examination should include RBC, WBC, HGB, HCT, PLT, EOS, BASO, neutrophil and LYMPH), monocyte count

(MONO). Screening period for examination.

6) Blood biochemical examination should include potassium (K+), sodium (Na+), chlorine (Cl-), calcium (Ca2+), magnesium (Mg2+), phosphorus (P), blood glucose (GLU), creatinine (CREA), UREA (UREA), glutamine transferase (GGT), uric acid (UA), lactate dehydrogenase (LDH), total bilirubin (TBIL)), aspartate aminotransferase (AST), alanine aminotransferase (ALT), alkaline phosphatase (ALP), triglyceride (TG), total cholesterol (CHOL), total protein (TP), albumin (ALB). Check during the screening period.

7) HBsAg, HCV antibody, HIV antibody, syphilis specific antibody.

8) Women must undergo a blood pregnancy test within -1 day of the screening period.

9) Routine urine examination should include glucose (GLU), ketone body (KET), protein (PRO), urinary occult blood (BLD), white blood cell (WBC), bilirubin (BIL), nitrite (NIT), pH, urochologen (UBG), specific gravity (SG); Check once during screening period.

(10) Alcohol breath tests and drug abuse screening (including morphine, methamphetamine, ketamine, dimethylamphetamine and THC) are performed during the screening period.

11) 12-lead electrocardiogram was examined at -1 day during screening period.

2. Single point optimal subcutaneous dose test procedure for long-acting Ropivacaine injection:

Table 9: The optimal subcutaneous dose test flow chart of long-acting ropivacaine injection was investigated

**Visit to describe**	**Screening Period (days)**		**Trial Period (days)**		**follow-up period[22]（days）**
**Time (day)**	**-14-（-2）**	**-1**	**1**	**2[20]**	**3-21**
Informed consent	X				
Demographic data	X				
Past and present medical history[1]	X				
Personal history and Family History[2]	X				
physical examination[3]	X	X		X	
vital signs[4]	X	X	X	X	
Potentially difficult airway[5]	X				
blood routine examination[6]	X			X	
blood biochemistry[7]	X			X	
serological examination [8]	X				
Coagulation function test[9]	X				
Blood pregnancy test[10]	X	X		X	
urine routines[11]	X			X	
Pain test[12]	X				
Alcohol breath test and drug abuse screening[13]		X			
routine 12-leads electrocardiogram [14]	X	X		X	
electrocardiograph monitoring[15]			X[15]	X[15]	
drug combination[16]			X	X	X
adverse event（including SAE）	X	X	X	X	X
Screening to the check-in questionnaire[17]		X			

**Visit to describe**	**Screening Period (days)**		**Trial Period (days)**		**follow-up period[22]（days）**
**Time (day)**	**-14-（-2）**	**-1**	**1**	**2[20]**	**3-21**
Inclusion and Exclusion Criteria	X	X			
check in		X			
The experimental drug was injected subcutaneously			X		
Analgesic range measurement[18]			X		
Local skin irritation at the injection site[19]			X	X	X
out group[21]				X	
Release/use/recovery of experimental drug			X		

Notes：

1) The subject's past medical history and current medical history shall be obtained, including the history of diseases of di gestive system, central nervous system, cardiovascular system, kidney and respiratory system.

2) Personal history should include marital history, smoking history, drinking history, drug use history, drug abuse history a nd allergy history; Family history.

3) Physical examination includes head, skin, mucous membranes, superficial lymph nodes, neck, chest, abdomen, spine/limb s. During the screening period, D2 was checked.

4) Measurement of vital signs: armpit temperature, decubitus blood pressure, pulse, respiration and oxygen saturation were measured during screening, group exit or early exit. Supine blood pressure, pulse, respiration, and oxygen saturation were measured 1h, 2h±15min, 4h, 8h, and 12h± 30min after the first administration, and axillary body temperature was measure d 4h, 12h± 30min after the first administration.

5) The assessment of potentially difficult airway included independent risk factors assessment of mask ventilation difficultie s, assessment of characteristic factors of laryngoscope exposure and intubation difficulties, and relevant physical examinatio n assessment. 6 cm) could be considered as a potentially difficult airway. Evaluate once during the screening period.

6) Routine blood examination should include RBC, WBC, HGB, HCT, PLT, EOS, BASO, neutrophil and LYMPH), mono cyte count (MONO). Screening period and d2 days each examination.

7) Blood biochemical examination should include potassium (K+), sodium (Na+), chlorine (Cl-), calcium (Ca2+), magnesiu m (Mg2+), phosphorus (P), blood glucose (GLU), creatinine (CREA), UREA (UREA), glutamine transferase (GGT), uric a cid (UA), lactate dehydrogenase (LDH), total bilirubin (TBIL)), aspartate aminotransferase (AST), alanine aminotransferase (ALT), alkaline phosphatase (ALP), triglyceride (TG), total cholesterol (CHOL), total protein (TP), albumin (ALB). Once du ring screening period and once on d2 days;

8) Serological examination included HBsAg, HCV antibody, HIV antibody and syphilis specific antibody. Check once durin g screening period.

9) The coagulation function test included prothrombin time (PT), activated partial thrombin time (APTT), thrombin time (T T) and fibrinogen content (FIB). Check once during screening period.

10) Women must have a blood pregnancy test during the screening period and d2 days.

11) Routine urine examination should include glucose (GLU), ketone body (KET), protein (PRO), urinary occult blood (BL D), white blood cell (WBC), bilirubin (BIL), nitrite (NIT), pH, urochologen (UBG), specific gravity (SG); Once during scr eening period and once on d2 days.

12) Pain test: measure the maximum mechanical pressure applied vertically when VAS=4.

13) Alcohol breath test and drug abuse screening (including morphine, methamphetamine, ketamine, methylene dioxy-amphe tamine, THC) should be performed once a day.

14) 12-lead electrocardiogram was examined during the screening period and d2 days.

15) When necessary.

16) Collect drug combination data, including drug dose, route of administration, administration schedule, start date, indicati on, and end date, and record the data from the beginning of the trial until the end of follow-up.

17) After screening, subjects should be asked whether they suffer from acute diseases; Have you taken any alcohol-based p roducts in the 24 hours prior to study administration; Whether you exercise vigorously; Whether to maintain a good living condition; Drink (or consume) theophylline, caffeine, theobromine, and alcohols 48 hours before administration of the study drug.

18) Determination of analgesic range: 15min, 30min, 45min±3min, 1h, 1.5h, 2h, 3h, 4h, 5h, 6h, 7h, 8h, 9h, 10h, 11h, 12h ±5min after the single point of administration, pain points were determined in two directions (when the VAS value of th e test point (n) 1cm away from the point of administration was ≥4, The measurement can be terminated at (n+1) time poi nt), the distance between pain point and drug administration point (R1, R2), analgesic range diameter D = (R1 + R2) wer e measured and recorded.

19) Local skin irritation at the injection site: within 30min before injection, 20min, 40min±5min, 1h, 2h, 6h, 24h±10min a fter injection, the injection site irritation score was observed and recorded before leaving the hospital. Local characteristics of the injection site should be inquired or visited during the follow-up period.

20) When subjects withdraw from the study, they should be checked as far as possible according to the safety evaluation on the second day after administration to complete the withdrawal visit.

21) If the analgesic reaction does not disappear, continue to observe in the phase I experimental ward until the analgesic reaction disappears before leaving the hospital.

22) Follow-up was conducted on days 7±2 and 21±3 after administration, and new local skin irritant reactions, adverse ev ents (including SAE) at the injection site from discharge to day 21, and drug combinations were asked.

3. Table of optimal subcutaneous dose test procedure for long-acting Ropivacaine Injection at single point:

Table 10: Safety and tolerability, pharmacokinetics and pharmacodynamics of a single subcutaneous administration of long-acting ropivacaine injection at different doses in healthy subjects

Visit to describe	Screening Period (days)		Trial Period (days)				Follow-up period[22]（days）
**Time (day)**	**-14-（-2）**	**-1**	**1**	**2**	**3**	**4[21]**	**7-21**
Informed consent	X						
Demographic data	X						
Past and present medical history[1]	X						
Personal history and Family History[2]	X						
physical examination[3]	X	X				X	
vital signs[4]	X	X	X	X	X	X	
Potentially difficult airway[5]	X						
blood routine examination[6]	X					X	
blood biochemistry[7]	X					X	
serological examination[8]	X						
Coagulation function test[9]	X						
Blood pregnancy test[10]	X	X				X	
urine routines[11]	X					X	
Pain test[12]	X						
Alcohol breath test and drug abuse screening[13]		X					
routine 12-leads electrocardiogram [14]	X	X				X	
electrocardiograph monitoring[15]			X[15]	X[15]	X[15]	X[15]	
drug combination[16]			X	X	X	X	X
Adverse events (including SAE)			X	X	X	X	X
Screening to the check-in questionnaire [17]		X					
Inclusion and Exclusion Criteria	X	X					
check in		X					
The experimental drug was injected subcutaneously			X				
Blood pharmacokinetic samples were collected[18]			X	X	X	X	
Intensity of needling pain[19]		X	X	X	X	X	
Local skin irritation at the injection site[20]			X	X	X	X	
Test drug use/recovery/destruction			X				
out group						X	

Notes:

1) The subject's past medical history and current medical history shall be obtained, including the history of diseases of digestive system, central nervous system, cardiovascular system, kidney and respiratory system.

2) Personal history should include marital history, smoking history, drinking history, drug abuse history and allergy history; Family history.

3) Physical examination includes head, skin, mucous membranes, superficial lymph nodes, neck, chest, abdomen, spine/limbs. Twice during screening period and once on day 4.

4) Measurement of vital signs: axillary body temperature, supine blood pressure, pulse, respiration and blood oxygen saturation were measured during the screening period, the second and third days of the trial period, and at the time of group exit or early exit. Supine blood pressure, pulse, respiration and oxygen saturation were measured at 1h before the first day of administration, 1h, 2h(±15min), 4h, 8h and 12h(± 30min) after the end of administration, and axillary body temperature was measured at 4h and 12h(± 30min) after the end of administration.

5) The assessment of potentially difficult airway included independent risk factors assessment of mask ventilation difficulties, assessment of characteristic factors of laryngoscope exposure and intubation difficulties, and relevant physical examination assessment. 6 cm) could be considered as a potentially difficult airway. Evaluate once during the screening period.

6) Routine blood examinations should include RBC, WBC, HGB, HCT, PLT, EOS, BASO, NEUtrophils, LYMPH and LYMPH Nuclear cell count (MONO). The screening period and the fourth day after the end of administration were examined.

7) Blood biochemical examination should include potassium (K+), sodium (Na+), chlorine (Cl-), calcium (Ca2+), magnesium (Mg2+), phosphorus (P), blood glucose (GLU), creatinine (CREA), UREA (UREA), glutamine transferase (GGT), uric acid (UA), lactate dehydrogenase (LDH), total bilirubin (TBIL)), aspartate aminotransferase (AST), alanine aminotransferase (ALT), alkaline phosphatase (ALP), triglyceride (TG), total cholesterol (CHOL), total protein (TP), albumin (ALB). One examination was performed during screening period and one on day 4.

8) Serological examination included HBsAg, HCV antibody, HIV antibody and syphilis specific antibody. Check once during screening period.

9) The coagulation function test included prothrombin time (PT), activated partial thrombin time (APTT), thrombin time (TT) and fibrinogen content (FIB). Check once during screening period.

10) Women must have a blood pregnancy test at screening and on day 4.

11) Routine urine examination should include glucose (GLU), ketone body (KET), protein (PRO), urinary occult blood (BLD), white blood cell (WBC), bilirubin (BIL), nitrite (NIT), pH, urochologen (UBG), specific gravity (SG); The screening period and the fourth day were examined once.

12) Pain test: measure the mechanical pressure value applied vertically when VAS=4.

13) Alcohol breath test and drug abuse screening (including morphine, methamphetamine, ketamine, methylene dioxy-amphetamine, THC) should be performed once a day.

14) 12-lead electrocardiogram was examined once during screening period, once on day -1 and once on day 4.

15) When necessary.

16) Collect drug combination data, including drug dose, route of administration, administration schedule, start date, indication, and end date, and record the data from the beginning of the trial until the end of follow-up.

17) After screening, subjects should be asked whether they suffer from acute diseases; Have you taken any alcohol-based products in the 24 hours prior to study administration; Whether you exercise vigorously; Whether to maintain a good living condition; Drink (or consume) theophylline, caffeine, theobromine, and alcohols 48 hours before administration of the study drug.

18) Collection of blood pharmacokinetic samples: Blood pharmacokinetic samples were collected at 0.5h, 1h, 1.5h, 2h(±2min), 4h, 6h, 8h, 10h, 12h, 21h, 24h, 27h, 30h, 33h, 36h, 48h and 72h(±5min) after 15min before administration.

19) Acupuncture pain intensity was measured before and after administration at 15min, 1h, 4h, 8h, 10h, 12h, 14h, 21h, 24h, 27h, 30h, 33h, 36h, 48h and 72h (the time window of measurement point from 15min to 72h was ±15min).

20) Local skin irritation at the injection site: scores were scored before and after administration 20min, 40min, 1h, 2h, 6h, 24h, 48h and 72h (the time window from 20min to 1h observation point was ±3min; The observation time window from 2h to 72h is ±5min).

21) When subjects withdraw from the study, they should be checked as far as possible according to the safety evaluation on the fourth day after the end of drug administration to complete the withdrawal visit.

22) Telephone follow-up was conducted on days 7±2 and 21±3 after administration, and new local skin irritant reactions, adverse events (including SAE) at the injection site occurred from discharge to day 21, and the situation of combined medication were asked.
