# Supplementary material for: A randomized trial: The safety, pharmacokinetics and preliminary pharmacodynamics of ropivacaine oil delivery depot in healthy subjects
Source: PLoS One. 2023 Sep 19;18(9):e0291793. doi: 10.1371/journal.pone.0291793 (PMC10508611; doi:10.1371/journal.pone.0291793)
Supplement: S2 File — (PDF) [file pone.0291793.s003.pdf]

临床研究方案

随机、单盲、阳性对照观察长效罗哌卡因注射液单次给药在健康受试者中安全性、耐受性、药代动力学及药效学的Ⅰ期临床研究

严禁复制

方案摘要

**题　　目**	随机、单盲、阳性对照观察长效罗哌卡因注射液单次给药在健康受试者中安全性、耐受性、药代动力学及药效学的I期临床研究
**研究药物名称**	长效罗哌卡因注射液
**临床批件号**	/
**注册分类**	化学药品第 2.2 类
**方案编号**	LB-RSRI-I-AICOMER
**临床试验分期**	I 期
**研究单位**	首都医科大学附属北京佑安医院
**主要研究者**	/
**申办者**	/
**试验目的**	**主要目的：**<br>1. 考察长效罗哌卡因注射液不同剂量单次皮下给药在健康受试者中的安全性和耐受性；<br>2. 考察长效罗哌卡因注射液单次皮下给药在健康受试者中的药代动力学。<br>3. 确定长效罗哌卡因注射液单点腹部皮下推荐给药剂量。<br>**次要目的：**<br>1. 考察长效罗哌卡因注射液单次皮下给药在健康受试者中的药效。<br>2. 考察镇痛起效时间、持续时间、镇痛强度，确定最佳给药间距。
**研究设计**	随机、单盲、阳性药物对照、单次皮下给药、剂量递增临床研究（基于临床腹腔镜手术微创特点）
**试验药物**	试验药：长效罗哌卡因注射液　规格：200mg/10mL<br>生产商：西安力邦制药有限公司<br>有效期：暂定 24 个月<br>储存条件：2~8°C避光贮藏。（不可冷冻）<br>对照药：盐酸罗哌卡因注射液（耐乐品 Naropin） 规格：100mg/10mL<br>生产商：AstraZeneca AB<br>有效期：36 个月<br>储存条件：30°C 以下室温贮藏，避免冻结。
**计划受试者例数**	本研究计划入组健康受试者 50 例，男女比例不限。<br>（1）其中 6 例健康受试者用于试验所需全血及空白血浆样本的采集。<br>（2）其中 3 例健康受试者用于考察长效罗哌卡因注射液单点皮下最佳给药剂量探索试验。<br>（3）其中 41 例健康受试者用于考察长效罗哌卡因注射液不同剂量单次皮下给药

严禁复制

	在健康受试者中的安全性和耐受性、药代动力学和药效学。
**入选标准**	1) 充分了解试验目的和要求，自愿参加并签署经伦理委员会批准的知情同意书者；<br>2) 年龄为 18-50 周岁（包括 18 周岁和 50 周岁）的中国成年男性和女性，男女均有；<br>3) 体重指数（BMI）在 19.0~26.0 kg/m^2（包括临界值）；BMI=体重（kg）/身高 ^{2}（m^2）；
**排除标准**	1) 对本试验药物或试验药物中任何成分或同类药物过敏者，或属于过敏性体质者；<br>2) 生命体征检查、体格检查、临床实验室检查（血常规、尿常规、血生化、血清学检查等）、12 导联心电图，结果经研究者判断异常且有临床意义者；<br>3) 有肝脏、肾脏、呼吸、血液或淋巴、内分泌、免疫（包括 HIV 检测阳性或其它免疫缺陷疾病等）、精神、胃肠道系统等慢性疾病史或严重疾病史，或有可能影响药物吸收、分布、代谢、排泄的手术或疾病者；<br>4) 经评估有潜在困难气道风险者；既往及当前有支气管哮喘、慢性阻塞性肺病、睡眠呼吸暂停综合征等呼吸系统疾病者；<br>5) 既往及当前有体位性低血压、心律失常、高血压、癫痫等心脑血管疾病者；<br>6) 具有长 QT 综合征或其家族史（祖父母、父母和兄弟姐妹），或 QTc 间期＞450 ms 者；室内传导阻滞或左右束支传导阻滞和/或 QRS＞120ms；室性异位搏动频发（筛选期任意一次 10s ECG 发生室性早搏≥1 个）；或静息心率异常（＞100 bpm）者；<br>7) 有麻醉意外史者；<br>8) 卟啉症患者；<br>9) 不能耐受静脉穿刺采血者；<br>10) 疤痕体质者；<br>11) 营养不良或低血容量性休克病史者；<br>12) 筛选前 2 周内使用过任何处方药、非处方药、保健品、中草药或中成药者；<br>13) 经常使用镇静、安眠药或其他成瘾性药物者或药物滥用筛查结果阳性者；<br>14) 在整个研究期及研究结束后 3 个月内有生育、捐精、捐卵计划或不同意在试验期间及研究结束后 3 个月采取有效的非药物避孕措施者（包括男性）；<br>15) 育龄期女性血妊娠试验检查结果高于正常值范围或处于哺乳期的女性；<br>16) 既往长期饮用过量茶、咖啡和/或含咖啡因的饮料（1 天 8 杯以上，1 杯=250mL）者，或试验期间不能放弃饮用茶、咖啡等饮料者；<br>17) 最近 3 个月内吸烟超过 5 支/日，或在整个研究期间不能放弃吸烟者；<br>18) 筛选前 12 个月内有酗酒史者（即男性每周饮酒超过 28 个标准单位，女性每周饮酒超过 21 个标准单位；1 单位相当于啤酒 285mL，或烈酒 25 mL，或葡萄酒 150 mL），或筛选前 6 个月内经常饮酒（每周饮酒超过 14 个标准

	单位），或酒精呼气检测为阳性者，或整个研究期间不能放弃饮酒者；<br>19) 筛选前 3 个月内献过血或失血≥400mL 者，或计划在研究期间及研究结束后 3 个月内参与献血者；<br>20) 筛选前 3 个月内参加过其它药物临床试验，或计划在研究期间参加其它药物临床研究者；<br>21) 预期参加研究期间需要手术或住院者；<br>22) 研究者认为痛觉异常，不适宜参加本试验者；<br>23) 研究者认为不宜参加本研究的其它情况。
**起始剂量和最大剂量的确定**	参照原国家食品药品监督管理总局《健康成年志愿者首次临床试验药物最大推荐起始剂量的估算指导原则》2012-05-15及FDA等相关文献。<br>**起始剂量的确定**<br>本试验以动物毒理学试验的未见明显毒性反应剂量（No Observed Adverse Effect Level，NOAEL，mg/kg）为基础，使用人体等效剂量（Human Equivalent Dose，HED，mg/kg）推导临床试验的最大推荐起始剂量（Maximum Recommended Starting Dose，MRSD，mg/kg）。<br>本品临床前动物毒性试验，比格犬一般药理和急性毒试验中，未见明显毒性反应剂量（NOAEL）为60 mg/kg，以下计算中，成人体重以60kg计，安全系数（safe factor，SF）取10。<br>1）根据体表面积换算人体等效剂量（Human Equivalent Dose，HED，）并计算MRSD：<br>结合以下公式及正文表2中的Km数据计算长效罗哌卡因注射液的初始剂量：<br>HED= NOAEL×动物Km/人体Km。<br>HED= 60mg/kg×21.47/36.88=34.93mg/kg<br>MRSD =HED（mg）/ SF<br>MRSD=34.93mg/kg×1/10=3.49mg/kg<br>成人体重以60kg计，3.49mg/kg×60kg=209.40mg，即起始剂量为209.40mg。<br>2）使用mg/kg换算人体等效剂量（HED）并计算MRSD：<br>HED（mg/kg）= NOAEL（mg/kg）=60mg/kg<br>MRSD=60mg/kg×1/10=6mg/kg<br>成人体重以60kg计，6mg/kg×60kg=360mg，即起始剂量为360mg<br>3）根据相关文献计算人体等效剂量（HED）并计算MRSD<br>HED（mg/kg）= NOAEL×Rab=60mg/kg×0.541=32.46mg/kg<br>MRSD =32.46mg/kg×1/10=3.25mg/kg<br>成人体重以60kg计，3.25mg/kg×60kg=195.00mg，即起始剂量可以确定为195.00mg。

本品临床前镇痛试验结果显示，大鼠3.6mg/只剂量组药效作用不明显，与溶剂组和模型组比较痛阈值略高但无显著性差异（$p>0.05$），HED（按大鼠3.6mg/0.25kg，成人60kg标准体重计算） ＝Da×Rab×60kg=（3.6mg/0.25kg）×0.162×60kg=140.00mg，即大鼠3.6mg/只的人体等效剂量为140.00mg/人，该剂量下镇痛药效作用不明显。

已上市盐酸罗哌卡因注射液（耐乐品）最高单次给药剂量为250mg曾被使用过，而且能够很好耐受；急性疼痛控制（区域阻滞）的推荐剂量为2-200mg。

综上，为了充分保障长效罗哌卡因注射液首次用于人体临床研究的安全性，同时兼顾本品制剂特点、皮下注射的给药方式和产生有效数据等各方面因素，我们将I期耐受性临床试验中健康受试者的起始注射剂量设定为150mg/次。

有效剂量的估算

根据长效罗哌卡因注射液临床前 SD 大鼠药效学试验结果，本品给药剂量大于等于 0.4mL（8mg）时，在给药后各时间点，对大鼠热刺激痛阈最高且无显著差异，估算拟用于人体的有效剂量约为 5.2 mg/kg，以成人 60kg 体重计算，临床有效剂量约为 300mg/次。

临床前药代动力学试验结果显示，耐乐品对照组与长效罗哌卡因试验组同剂量（10mg/kg）单次皮下注射药代动力学参数 Cmax 的比值为 4.76（2.76μg/mL / 0.58μg/mL）。

临床前犬急性毒性试验结果显示，犬单次皮下注射长效罗哌卡因注射液无明显不良反应的剂量为 60 mg/kg，换算成人的剂量 32 mg/kg。以成人 60kg 体重计算，HED 为 1920mg/人。

已上市盐酸罗哌卡因注射液（耐乐品）说明书中，手术麻醉中累计剂量达到800mg 时，对成人来说都可很好耐受。

最大剂量的确定

根据未观测到的不良反应剂量（NOAEL）计算人体最大剂量

临床前大鼠急性毒性结果显示，SD 大鼠单次皮下注射长效罗哌卡因注射液的最大耐受剂量为 150 mg/kg，换算至人体的剂量 24.3 mg/kg，以成人 60kg 体重计算，最大剂量为 1458mg/次。

临床前犬急性毒性试验结果显示，犬单次皮下注射长效罗哌卡因注射液无明显不良反应的剂量为 60 mg/kg，换算成人的剂量 32 mg/kg。以成人 60kg 体重计算，最大剂量为 1920mg/次，取 1/5 至 1/2 做最大耐受量估算，换算为人体最大剂量范围为 360mg/次至 900mg/次。

已上市盐酸罗哌卡因注射液（耐乐品）说明书中，盐酸罗哌卡因注射液应用于硬膜外阻滞，最高 250mg 剂量呈现良好耐受；单次手术麻醉中累计剂量达到

<table>
<tr><td></td><td>800mg 时，对成人来说都可很好耐受。
临床前药效学试验结果显示，大鼠给予盐酸罗哌卡因皮下注射 12mg/只时，有 50%动物死亡，50%动物出现全身麻醉反应；但试药 12mg/只组均未出现上述现象，且在相同给药容量时，试药可明显延长镇痛时间。提示：用药部位影响药效剂量范围，且大鼠 12mg/只的人体等效剂量 HED466.80mg 为重要关注剂量。
本试验药物为皮下注射给药，规格为 10 mL:200 mg，随着剂量的进一步增加，疗效可能并不呈明显的线性关系，因此确定将 400 mg，相当于 20mL 试验制剂作为健康受试者的最大剂量，用于观察长效罗哌卡因注射液的安全性和耐受性。</td></tr>
<tr><td>给药剂量的确定和递增方法</td><td>（1）长效罗哌卡因注射液单点皮下最佳给药剂量探索试验，确定单点最佳给药剂量
研究初步设定单点给药剂量包括：0.6mL（12mg）、0.8mL（16mg）、1.0mL（20mg）、1.2mL（24mg）、1.5mL（30mg）五个剂量，总计 5.1mL（102mg）。3 例健康受试者，每位受试者单次不同给药点给予五种不同剂量药物。

（2）长效罗哌卡因注射液不同剂量单次皮下给药安全性、耐受性和药代动力学试验
根据临床前动物试验结果，确定将 150 mg 作为健康受试者的起始剂量。从最低起始剂量到最大剂量分为五个队列，剂量递增方案见下表

健康受试者单次给药试验给药剂量递增方案
<table>
<tr><th rowspan="2">队列</th><th rowspan="2">剂量（mg）</th><th colspan="2">健康受试者例数（n）</th></tr>
<tr><th>试验组
长效罗哌卡因注射液</th><th>对照组
盐酸罗哌卡因注射液</th></tr>
<tr><td>A</td><td>150</td><td>6</td><td>3</td></tr>
<tr><td>B</td><td>230</td><td>8</td><td>--</td></tr>
<tr><td>C</td><td>300</td><td>8</td><td>--</td></tr>
<tr><td>D</td><td>350</td><td>8</td><td>--</td></tr>
<tr><td>E</td><td>400</td><td>8</td><td>--</td></tr>
<tr><td colspan="2">共计</td><td>38</td><td>3</td></tr>
</table>
A 队列设阳性对照药对照，其他剂量组不设阳性对照组。研究从最小剂量开始给药，每位受试者只接受一个剂量给药。队列 A 分 3 批受试者进行，第一批试验组和阳性对照组各 1 例；第二批试验组 2 例，阳性对照药组 1 例；第三批试验组 3 例，阳性对照药组 1 例。队列 B 至队列 E 只设试验组。同一时间内只进行 1 个剂量队列的研究，分两批受试者进行，第一批 3 例，第二批 5 例。在给药后第 7 天和第 21 天进行安全性评估，并根据评估结果决定是否进行下一队</td></tr>
</table>

严禁复制

	列/批次试验。若在最大剂量队列（400mg）中仍未观察到明显不良反应，则不再继续进行剂量递增。
**给药方案**	（1）考察长效罗哌卡因注射液单点皮下最佳给药剂量探索试验给药方案（**试验 1）：**<br>受试者腹部分六区，从剑突部位开始顺时针方向依次标注为 I、II、III、IV、V、VI。<br>试验药五个剂量：0.6mL（12mg）、0.8mL（16mg）、1.0mL（20mg）、1.2mL（24mg）、1.5mL（30mg）。<br>3 例健康受试者，每位受试者腹部六区需随机化确定起始给药区，每个区域内任意选取一个点作为注射点（**任意两点间的距离不低于 5cm**），并顺时针依次标注为 1、2、3、4、5，第 6 个点作为疼痛对照点（对照点疼痛仅测基线值），考察 5 种不同剂量单点给药部位的局部皮肤刺激性，镇痛范围，确定单点最佳给药剂量及其镇痛范围。<br>测量不同注射点周围区域对刺痛的反应，以注射点为圆心，隔 1cm 距离放射状散开进行疼痛点测量（若 VAS 值≥4 时，则返回 0.5cm 处重新测量），测得镇痛范围两个方向的半径。<br>确定**最佳单点给药剂量（L）**和该给药剂量的**镇痛范围的平均直径(D)**。<br>（2）考察长效罗哌卡因注射液不同剂量单次皮下给药在健康受试者中的安全性和耐受性及药代动力学试验给药方案（**试验 2**）：<br>由试验 1 结果获得单点推荐给药剂量为 1.2mL，其镇痛范围的平均直径为 40mm。<br>受试者按筛选号顺序分组，A 队列，分为试验组和对照组，其他队列仅设试验组。每个受试者只接受一个剂量给药，在确定低剂量安全后，再进行高剂量的试验。<br>受试者腹部分四区，从剑突部位开始顺时针方向依次标注为I、II、III、IV。受试者给药当天按受试者所属的剂量队列，经腹部单次多点皮下注射相应剂量的试验药物。<br>同一队列每位受试者腹部四区随机化确定起始给药区域，顺时针依次标注为1、2、3，第4个区域作为疼痛对照区域；每个区域内选取1个点为圆心（任意两圆心间距离不低于80mm），标记出4个直径约为40mm的圆，其中1、2、3区域，沿标记的圆平均分布，皮下注射给予试验药，每针注射量为1.2mL。而第4个区域为空白对照，用于痛觉对照。<br>按照每个队列设计的给药剂量，注射点在给药圆周上平均分布，每次给药量直至达到每队列设计的总给药剂量。

	按1.2mL/针计算每个队列的注射点数量，即每队列注射点数量=该队列剂量/1.2，取整数确定第1、2个圆周上的注射点数量，剩余剂量分配在第3个圆上；每个区域的注射点需在该区域圆周上平均分布。
**试验周期**	本研究分为筛选期、试验期和随访期三个阶段，筛选期为给药前-14 天至给药前-1 天；试验期：试验 1 试验期为给药第 1-2 天，试验 2 试验期为给药第 1 天至给药后第 4 天；随访期在给药后（试验 1 为第 3 天，试验 2 为第 5 天）至第 21 天进行，在第 7 天和第 21 天时各进行 1 次随访。
**试验终止标准**	1. 若在试验进行中出现任何一例与研究药物相关的严重不良事件（SAE），研究者与申办者一起充分讨论分析原因，并判断对后续试验的影响后，再决定是否继续试验；<br>2. 参照《常见不良事件评价标准（CTCAE）》（V5.0，2017年11月27日公布），若试验队列中≥1/2受试者出现≥Ⅱ级不良事件或≥1/3的受试者出现≥III级不良事件（不良事件需经研究者判断可能与试验药物相关），则由研究者与申办方讨论，是否终止剂量递增；<br>3. 若在最大剂量队列（400mg）中仍未观察到明显不良反应，则不再继续进行剂量递增。<br>4. 申办方要求全面终止试验；<br>5. 国家药品监督管理局或伦理委员会因某种原因勒令终止试验。
**观察指标**	**1. 主要指标**<br>（1）安全性和耐受性试验观察指标：包括临床症状与体征、实验室检查、注射部位局部皮肤刺激性反应评估、不良事件（包括全身不良反应及注射部位外观、注射部位疼痛或压痛、注射部位红斑或发红、注射部位硬结或肿胀、注射部位瘙痒等）；<br>（2）单点皮下最佳给药剂量探索试验观察指标包括：注射部位皮肤刺激性评分，镇痛范围的平均直径（试验 1）；<br>（3）药代动力学试验检测：血浆中药物的浓度、药代动力学参数。<br>**2. 次要指标**<br>药效学试验观察指标：观测并比较试验组与阳性对照组两种药物的镇痛作用持续时间、针刺疼痛强度和镇痛起效时间。<br>（1） 镇痛持续时间；<br>（2） 镇痛起效时间；<br>（3） 针刺疼痛强度；<br>（4） 注射点的镇痛范围（试验 1）；<br>（5） 同一圆周上相邻两个给药点之间的直线距离。

严禁复制

**药代动力学**	试验 2 进行药代动力学考察<br>考察单次给药的血药浓度-时间数据和药代动力学参数。<br>结合罗哌卡因注射液的特点，参照试验药 Beagle 犬临床药代动力学试验报告，初步拟定健康受试者的血样采集时间点为：<br>给药前 15min 内，给药结束后 0.5h、1h、1.5h、2h、4h、6h、8h、10h、12h、14h、21h、24h、27h、30h、33h、36h、48h 和 72h。<br>采用 LC-MS/MS 测定人血浆中罗哌卡因的浓度。根据试验中测得的各受试者的血药浓度-时间数据，绘制各受试者的药-时曲线及平均药-时曲线，并计算药代动力学参数。
**药效学**	（1）镇痛维持时间、起效时间和针刺疼痛强度的测量：给药前和给药结束后 15min、1h、4h、8h、10h、12h、14h、18h、21h、24h、27h、30h、33h、36h、48h、72h 测量针刺疼痛强度。<br>（2）镇痛范围测量：于单点给药结束后 15min、30min、45min,，1h、1.5h、2h、3h、4h、5h、6h、7h、8h、9h、10h、11h、12h，沿两个方向测量镇痛范围（试验 1）。
**统计分析**	单点皮下最佳给药剂量试验：描述性统计分析<br>安全性和耐受性试验：描述性统计分析<br>药代动力学试验：计算各受试者血药浓度-时间数据，绘制浓度-时间曲线，并绘制平均血药浓度-时间曲线。同时计算单次给药药代动力学参数。<br>药效学试验：分析统计用药前后各项指标的水平变化；各组之间采用与基线差值的（将基线值作为协变量）最小二乘均数和 95%可信区间进行比较。
**研究方案修订**	本研究将根据已经获得的临床试验数据进行必要的方案修订，修订后的研究方案，必须得到申办者、研究者和伦理委员会的同意方能执行。

试验流程表 1：

考察长效罗哌卡因注射液单点皮下最佳给药剂量试验流程表

访视描述	筛选期（天）		试验期（天）		随访期[22]（天）
时间（天）	-14-（-2）	-1	1	2[20]	3-21
知情同意	X				
人口学资料	X				
既往史和现病史[1]	X				
个人史及家族史[2]	X				
体格检查[3]	X	X		X	
生命体征[4]	X	X	X	X	
潜在困难气道[5]	X				
血常规[6]	X			X	
血生化[7]	X			X	
血清学检查[8]	X				
凝血功能检查[9]	X				
血妊娠试验[10]	X	X		X	
尿常规[11]	X			X	
痛觉测试[12]	X				
酒精呼气试验及药物滥用筛查[13]		X			
12 导联心电图[14]	X	X		X	
心电监护[15]			X[15]	X[15]	
合并用药[16]			X	X	X
不良事件（包括 SAE）	X	X	X	X	X
筛选至入住问卷调查[17]		X			
入/排标准	X	X			
入住		X			
皮下注射试验药物			X		
镇痛范围测定[18]			X		
注射部位局部皮肤刺激性反应[19]			X	X	X
出组[21]				X	
试验药物发放/使用/回收			X		

备注：

1)　须获取受试者的既往病史及现病史情况，包括：消化系统、中枢神经系统、心血管系统、肾脏、呼吸系统疾病史。

2)　个人史应包含婚姻史、吸烟史、饮酒史、药物使用史、药物滥用史及过敏史；家族史。

3)　体格检查包括头部、皮肤、粘膜、浅表淋巴结、颈部、胸部、腹部、脊柱/四肢。在筛选期、d2 进行检查。

4)　生命体征测量：筛选期、出组或提前退出时测量腋下体温、卧位血压、脉搏、呼吸、血氧饱和度。给药当天于第一次给药前 1h 内，第一次给药后 1h、2h±15min、4h、8h、12h±30 min 测量卧位血压、脉搏、呼吸、血氧饱和度，于第一次给药前 1h 内，第一次给药后 4h、12h±30 min 测量腋下体温。

5）　潜在困难气道评估内容包括：面罩通气困难的独立危险因素评估，喉镜显露和插管困难的特征因素评估及相关体格检

查评估，当出现 Mallampati 分级III或IV级、下颌前伸能力受限、甲颏距离过短(<6 cm)等表现可评判为潜在困难气道。在筛选期评估 1 次。

6) 血常规检查内容应包括红细胞计数（RBC）、白细胞计数（WBC）、血红蛋白（HGB）、红细胞压积（HCT）、血小板计数（PLT）、嗜酸性细胞计数（EOS）、嗜碱性细胞计数（BASO）、中性粒细胞计数（NEUT）、淋巴细胞计数(LYMPH)、单核细胞计数（MONO）。筛选期和 d2 天各检查一次。

7) 血生化检查内容应包括钾（K+）、钠（Na+）、氯（Cl-）、钙（Ca^{2+}）、镁（Mg^{2+}）、磷（P）、血糖（GLU）、肌酐（CREA）、尿素（UREA）、谷氨酰胺转肽酶（GGT）、尿酸（UA）、乳酸脱氢酶（LDH）、总胆红素（TBIL）、天门冬氨酸氨基转移酶（AST）、丙氨酸氨基转移酶（ALT）、碱性磷酸酶（ALP）、甘油三酯（TG）、总胆固醇（CHOL）、总蛋白（TP）、白蛋白（ALB）。在筛选期和 d2 天各检查 1 次；

8) 血清学检查包括 HBsAg、HCV 抗体、HIV 抗体、梅毒特异性抗体。在筛选期检查 1 次。

9) 凝血功能检查内容包括凝血酶原时间(PT)、活化的部分凝血活酶时间(APTT)、凝血酶时间(TT)、纤维蛋白原含量(FIB)。在筛选期检查 1 次。

10）女性必须在筛选期和 d2 天进行血妊娠试验检查。

11) 尿常规检查内容应包括葡萄糖（GLU）、酮体（KET）、蛋白质（PRO）、尿潜血（BLD）、白细胞（WBC）、胆红素（BIL）、亚硝酸盐（NIT）、酸碱度（pH）、尿胆原（UBG）、比重（SG）；在筛选期和 d2 天各检查 1 次。

12) 痛觉测试：测量 VAS=4 时，垂直施加的最大机械压力值。

13)酒精呼气试验及药物滥用筛查（包括吗啡、甲基安非他明、氯胺酮、二亚甲基双氧安非他明、四氢大麻酚酸）在-1 天检查 1 次。

14) 12 导联心电图在筛选期、d2 天进行检查。

15） 必要时。

16) 收集合并用药的数据，包括药物剂量、给药途径、给药时间表、开始日期、适应症、结束日期，且须从试验开始一直记录直至随访结束。

17) 筛选至入住问卷调查应向受试者询问的事项包括：是否患急性疾病；是否在研究用药前 24 小时内服用过任何含酒精的制品；是否剧烈运动；是否保持良好的生活状态；在研究药物给药前 48 小时饮用（或食用）茶碱、咖啡因、可可碱、醇类。

18) 镇痛范围测定：于单点给药后 15min、30min、45min±3min，1h、1.5h、2h、3h、4h、5h、6h、7h、8h、9h、10h、11h、12h ±5min，沿两个方向确定疼痛点（当距离给药点为 1cm 的测定点（n）的 VAS 值≥4 时，测定至（n+1）时间点时可终止测量），测量并记录疼痛点和给药点之间的距离（r1，r2），镇痛范围直径 D =（r1+r2）；

19）注射部位局部皮肤刺激性反应：于注射前 30min 内，注射后 20min、40min±5min、1h、2h、6h、24h±10min，离院前观察并记录注射部位刺激性评分，随访期需问询或访视观察注射部位局部特征。

20）受试者退出研究时，应尽可能按给药后第 2 天的安全性评价进行检查，完成退出研究访视。

21）若镇痛反应未消失，需继续在 I 期试验病房观察至镇痛反应消失，方可离院。

22）给药后第 7±2、21±3 天进行随访，需询问出院后至第 21 天新发的注射部位局部皮肤刺激性反应、不良事件（包括 SAE）及合并用药情况。

试验流程表 2

长效罗哌卡因注射液不同剂量单次皮下给药在健康受试者中的
安全性和耐受性和药代动力学研究试验流程表

**访视描述**	**筛选期（天）**		**试验期（天）**				**随访期[22]（天）**
**时间（天）**	**-14-（-2）**	**-1**	**1**	**2**	**3**	**4[21]**	**7-21**
知情同意	X						
人口学资料	X						
既往史和现病史 1	X						
个人史及家族史 2	X						
体格检查 3	X	X				X	
生命体征 4	X	X	X	X	X	X	
潜在困难气道 5	X						
血常规 6	X					X	
血生化 7	X					X	
血清学检查 8	X						
凝血功能检查 9	X						
血妊娠试验 10	X	X				X	
尿常规 11	X					X	
痛觉测试 12	X						
酒精呼气试验及药物滥用筛查 13		X					
12 导联心电图 14	X	X				X	
心电监护 15			X[15]	X[15]	X[15]	X[15]	
合并用药 16			X	X	X	X	X
不良事件（包括 SAE）			X	X	X	X	X
筛选至入住问卷调查 17		X					
入/排标准	X	X					
入住		X					
皮下注射试验药物			X				
血液药代样本采集 18			X	X	X	X	
针刺疼痛强度 19		X	X	X	X	X	
注射部位局部皮肤刺激性反应 20			X	X	X	X	
试验药物使用/回收/销毁			X				
出组						X	

备注：

1) 须获取受试者的既往病史及现病史情况，包括：消化系统、中枢神经系统、心血管系统、肾脏、呼吸系统疾病史。

2) 个人史应包含婚姻史、吸烟史、饮酒史、药物滥用史及过敏史；家族史。

3) 体格检查包括头部、皮肤、粘膜、浅表淋巴结、颈部、胸部、腹部、脊柱/四肢。在筛选期检查 2 次，第 4 天检查 1 次。

4) 生命体征测量：筛选期、试验期第 2 天、第 3 天、出组或提前退出时测量腋下体温、卧位血压、脉搏、呼吸、血氧饱和度。给药当天（第 1 天）于第给药前 1h 内，给药结束后 1h、2h(±15min)、4h、8h、12h(±30 min)测量卧位血压、脉搏、

呼吸、血氧饱和度，于给药前 1h 内，给药结束后 4h、12h(±30 min)测量腋下体温。

5) 潜在困难气道评估内容包括：面罩通气困难的独立危险因素评估，喉镜显露和插管困难的特征因素评估及相关体格检查评估，当出现 Mallampati 分级Ⅲ或Ⅳ级、下颌前伸能力受限、甲颏距离过短(<6 cm)等表现可评判为潜在困难气道。在筛选期评估 1 次。

6）血常规检查内容应包括红细胞计数（RBC）、白细胞计数（WBC）、血红蛋白（HGB）、红细胞压积（HCT）、血小板计数（PLT）、嗜酸性细胞计数（EOS）、嗜碱性细胞计数（BASO）、中性粒细胞计数（NEUT）、淋巴细胞计数(LYMPH)、单核细胞计数（MONO）。筛选期、给药结束后第 4 天各检查一次。

7) 血生化检查内容应包括钾（K+）、钠（Na+）、氯（Cl-）、钙（Ca^{2+}）、镁（Mg^{2+}）、磷（P）、血糖（GLU）、肌酐（CREA）、尿素（UREA）、谷氨酰胺转肽酶（GGT）、尿酸（UA）、乳酸脱氢酶（LDH）、总胆红素（TBIL）、天门冬氨酸氨基转移酶（AST）、丙氨酸氨基转移酶（ALT）、碱性磷酸酶（ALP）、甘油三酯（TG）、总胆固醇（CHOL）、总蛋白（TP）、白蛋白（ALB）。在筛选期和第 4 天各检查 1 次；

8) 血清学检查包括 HBsAg、HCV 抗体、HIV 抗体、梅毒特异性抗体。在筛选期检查 1 次。

9) 凝血功能检查内容包括凝血酶原时间(PT)、活化的部分凝血活酶时间(APTT)、凝血酶时间(TT)、纤维蛋白原含量(FIB)。在筛选期检查 1 次。

10）女性必须在筛选期和第 4 天进行血妊娠试验检查。

11) 尿常规检查内容应包括葡萄糖（GLU）、酮体（KET）、蛋白质（PRO）、尿潜血（BLD）、白细胞（WBC）、胆红素（BIL）、亚硝酸盐（NIT）、酸碱度（pH）、尿胆原（UBG）、比重（SG）；筛选期和第 4 天各检查 1 次。

12) 痛觉测试：测量 VAS=4 时，垂直施加的机械压力值。

13）酒精呼气试验及药物滥用筛查（包括吗啡、甲基安非他明、氯胺酮、二亚甲基双氧安非他明、四氢大麻酚酸）在-1 天检查 1 次。

14) 12 导联心电图在筛选期、-1 天、第 4 天各检查 1 次。

15）必要时。

16) 收集合并用药的数据，包括药物剂量、给药途径、给药时间表、开始日期、适应症、结束日期，且须从试验开始一直记录直至随访结束。

17) 筛选至入住问卷调查应向受试者询问的事项包括：是否患急性疾病；是否在研究用药前 24 小时内服用过任何含酒精的制品；是否剧烈运动；是否保持良好的生活状态;在研究药物给药前 48 小时饮用（或食用）茶碱、咖啡因、可可碱、醇类。

18) 血液药代样本采集：于给药前 15min 内，给药结束后 0.5h、1h、1.5h、2h(±2min)、4h、6h、8h、10h、12h、14h、21h、24h、27h、30h、33h、36h、48h 和 72h(±5min)进行血液药代样本采集。

19) 针刺疼痛强度测量：于给药前和给药结束后 15min、1h、4h、8h、10h、12h、14h、18h、21h、24h、27h、30h、33h、36h、48h、72h 进行测量（15min 至 72h 测量点时间窗为±15min）。

20) 注射部位局部皮肤刺激性反应：于给药前和给药结束后 20min，40min，1h，2h，6h，24h、48h、72h 进行评分（20min 至 1h 观察点时间窗为±3min；2h 至 72h 观察点时间窗为±5min）。

21) 受试者退出研究时，应尽可能按给药结束后第 4 天的安全性评价进行检查，完成退出研究访视。

22) 给药后第 7±2、21±3 天进行电话随访，需询问给出院后至第 21 天新发生的注射部位局部皮肤刺激性反应、不良事件（包括 SAE）及合并用药情况。

1 概述

1.1 药物简介

1.1.1 药物名称和性状

通用名：长效罗哌卡因注射液

商品名称：暂无

英文名称：Ropivacaine Sustained-release Injection

汉语拼音：Changxiao LuopaiKayin Zhusheye

主要成份及其化学名称：本品为淡黄色无菌澄明油状溶液，主要成分为罗哌卡因，化学名：(-)-(S)-N-(2,6-二甲基苯基)-1-正丙基哌啶-2-甲酰胺 ((S)-N-(2,6-Dimethylphenyl)-1-propyl-piperidine-2-carboxamide)。

1.1.2 主要成分化学结构式、分子式、分子量、CAS 号

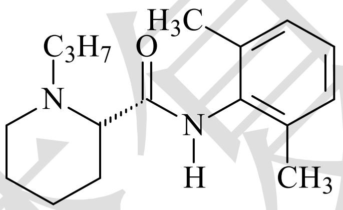

分子式：$C_{17}H_{26}N_2O$

分子量：274.4

CAS：84057-95-4

1.1.3 主要成分理化性质

罗哌卡因为白色或类白色结晶或结晶性粉末，熔点为 152℃~156℃。在甲基异丁基甲酮、乙醇中溶解，在水中几乎不溶，对热、光均稳定。解离常数 pKa 为 8.1，分配系数为 2.9。

1.1.4 处方组成

成分	组成比例
罗哌卡因游离碱 mg/mL	20
苯甲醇	4%
苯甲酸苄酯	30%
Ve	0.5%
大豆油	加至 100%

1.1.5 适应症

严禁复制

本品为油溶性注射液，可用于外科术后镇痛——手术切口周围神经阻滞。

1. 1. 6 规格

10mL:200mg。

1. 1. 7 拟定用法

长效罗哌卡因注射液为术后单次皮下给药。

1.2 药品类别

长效罗哌卡因注射液是罗哌卡因游离碱的油溶性注射液，本品为国内外均未上市新制剂品种，是一种具有全新配方和给药途径的药物，具有自主知识产权。按照化学药品注册分类方案，本品为境内外均未上市的改良型新药，属于化学药品注册分类 2.2 类。

1.3 药理作用及特点[1]

罗哌卡因是第一个纯左旋体酰胺类局麻药，有麻醉和镇痛双重效应，其感觉和运动阻滞明显分离。大剂量可产生外科麻醉，小剂量时则产生感觉阻滞(镇痛)仅伴有局限的非进行性运动神经阻滞。

罗哌卡因通过阻断钠离子流入神经纤维细胞膜内，对沿神经纤维的冲动传导产生可逆性的阻滞。

局麻药可能对如脑细胞和心肌细胞等易兴奋的细胞膜产生类似作用，如果过量药物快速地进入体循环，中枢神经系统和心血管系统将出现中毒症状和体征。

健康志愿者静脉注射罗哌卡因后耐受良好，临床经验提示此药有一个良好的安全范围。根据副交感神经阻滞程度，硬膜外使用此药可出现间接的心血管效应(低血压、心动过缓)。

长效罗哌卡因注射液为油性注射液，采用皮下注射，有明显的缓释效果，可明显延长单次给药的镇痛时间。

2 研究背景

普通罗哌卡因（ropivacaine）注射液是阿斯利康制药公司 1992 年开发的一类新的长效酰胺类局部麻醉药，它主要通过阻断钠离子流入神经纤维细胞膜内对沿神经纤维的冲动产生可逆性阻滞而发挥作用，作用机理同其它酰胺类的局部麻醉药相同，该药物于 1996 年[2]在荷兰首次上市，1998 年我国批准进口，目前已过专利有效期，国内有多家生产单位，主要的给药剂型是罗哌卡因盐（甲磺酸、盐酸）的普通水溶液。

普通罗哌卡因盐的水溶液已经成为临床局部麻醉一线用药，在术后镇痛方面，罗哌卡因

盐的水溶液主要通过局部神经阻滞与浸润麻醉的方式广泛的用于临床妇产科、骨科、普通外科等众多科室，镇痛效果良好、相对不良反应少。

目前临床资料表明，在安全的给药剂量范围内，罗哌卡因术后手术部位局部神经阻滞镇痛有效持续时间仅为 8-24 小时，而临床患者术后疼痛高峰期为术后的前 3 天，普通罗哌卡因水溶液单次注射药效远远不能满足患者的镇痛要求。

本次开展 I 期试验的新药为长效罗哌卡因注射液，是罗哌卡因游离碱的油溶性注射液，具有载药量大和释放缓慢的特点，本试验制剂与罗哌卡因盐的水溶液动物实验药效结果相比较，动物局部给药量增加 3.4 倍左右时，药效增加 3-6 倍左右，可覆盖临床术后疼痛高峰期。

3 临床前研究主要结果

3.1 药效学

3.1.1 对大鼠术后疼痛的缓解作用（300mg:10mL）[3]

SD 大鼠，雌雄各半，体重 250~300g，以 Brennan 法建立术后痛动物模型，在手术侧后肢腓神经处单次注射给药，分为 12、6.4、3.6mg 三个剂量组，给药体积均为 0.4ml/只 (10ml:300mg、10ml:160mg、10ml:90mg 浓度)，采用热刺激和机械压力刺激两种方法评价本品的镇痛效果，并与 0.4ml 的 100mg:10ml 浓度的盐酸罗哌卡因注射液、空白溶剂组对比。

在感觉神经阻滞方面，热刺激法试验结果显示，给药前大鼠的痛阈在 9.8~10.2s 之间，各组比较无显著性差异。长效罗哌卡因注射液 12mg 剂量组药效持续时间可达 10h，6.4mg 剂量组药效持续时间为 8h，而 3.6mg 剂量组药效作用不明显，盐酸罗哌卡因注射液组药效持续时间仅为 2h，溶剂则无镇痛效果。机械压力刺激法结果显示，给药前大鼠痛阈在 16.4~17.4g 之间，各组之间比较无显著性差异。长效罗哌卡因注射液 12mg 剂量组在给药后 10h 时痛阈值明显高于模型组($p< 0.01$)；6.4mg 剂量组药效可持续 8h 以上；3.6mg 剂量组、溶剂组与模型组相比较痛阈值略高但无显著性差异($p>0.05$)，盐酸罗哌卡因注射液药效仅持续 2h 左右。

在运动神经阻滞方面，采用评分法进行评估，模型组给药前后不同时间点的评分均为 0，运动功能正常，溶剂组和长效罗哌卡因注射液 3.6mg 剂量组的评分与模型组比较，运动功能障碍可持续到 10h，长效罗哌卡因注射液 6.4mg 剂量组和 12mg 剂量组与模型组相比较运动功能障碍可持续 12h 以上。

因此，长效罗哌卡因注射液与盐酸罗哌卡因注射液相比较，给药剂量增加3.4倍条件下，则相应的感觉神经阻滞时间延长5倍，具有一定的缓释作用，运动神经阻滞较感觉神经阻滞相

应的延长。

3. 1. 2 对大鼠疼痛的缓解作用[4]

根据临床批件建议，研究了不同处方的长效罗哌卡因注射液对正常大鼠和术后大鼠胫神经的阻滞作用，并与盐酸罗哌卡因注射液(100mg:10ml)比较。

3.1.2.1 对正常大鼠疼痛的缓解作用(200mg:10ml)

采用热刺痛仪选择痛域在10±1 s的雌性SD大鼠64只，随机分为8组，评价了相同给药容量和相同给药量时的镇痛作用时间。如下表给药。

分组	1	2	3	4	5	6	7	8
组别	试验组	试验组	试验组	对照组	试验组	试验组	试验组	对照组
规格	300mg:10ml	230mg:10ml	200mg:10ml	100mg:10ml	300mg:10ml	230mg:10ml	200mg:10ml	100mg:10ml
给药体积	0.4ml	0.4ml	0.4ml	0.4ml	0.4ml	0.5ml	0.6ml	1.4ml
剂量	12mg/只	9.2mg/只	8mg/只	4mg/只	12mg/只	11.5mg/只	12mg/只	12.3mg/只

采用热痛法和运动功能评分法评价其对胫神经的阻滞作用。结果表明，相同给药容量和相同给药量时，长效罗哌卡因镇痛时间均较盐酸罗哌卡因长。相同给药体积(0.4ml)时，盐酸罗哌卡因镇痛时长为2h，长效罗哌卡因镇痛时长在6~10h，其中300mg：10mlK(300mg:10ml)、200mg:10mlK6(230mg:10ml)的镇痛时长10h，200mg:10mlK7(200mg:10ml)的镇痛时长6h；相同给药剂量(约12mg/只)时，盐酸罗哌卡因镇痛时长4h，此时有37.5%的动物出现死亡，37.5%的动物出现全身麻醉反应，长效罗哌卡因300mg：10ml(300mg:10ml)、200mg:10ml(230mg:10ml、200mg:10ml)镇痛时长均为10h。各组配方对大鼠运动神经的阻滞作用与镇痛作用一致。

3.1.2.2 对大鼠术后疼痛的缓解作用(规格：200mg:10ml)[5]

在对正常大鼠疼痛的缓解研究基础上，再次研究了不同处方，在相同给药容量和相同给药剂量对大鼠术后的镇痛效果，据此对本品的处方进行了改进，初步确定降低本品处方中苯甲醇的量，规格确定为200mg:10ml。

选用 SD 雄性大鼠，体重 250~300g，以 Brennan 法建立术后痛动物模型，在手术侧后肢腓神经处单次注射给药，每组 8 只。分组和剂量如下表：

分组	1	2	3	4	5	6	7	8	9
组别	试验组	试验组	试验组	对照组	试验组	试验组	试验组	对照组	试验组
规格	300mg:10ml	230mg:10ml	200mg:10ml	100mg:10ml	300mg:10ml	230mg:10ml	200mg:10ml	100mg:10ml	200mg:10ml

给药体积	0.4ml	0.4ml	0.4ml	0.4ml	0.4ml	0.5ml	0.6ml	1.4ml	0.8ml
剂量	12mg/只	9.2mg/只	8mg/只	4mg/只	12mg/只	11.5mg/只	12mg/只	12.3mg/只	16mg/只

注：实验组用药为本品不同规格的油剂；对照组用药为 100mg:10ml 盐酸罗哌卡因注射液；剂量以碱基计。

采用热刺激和机械压力刺激两种方法评价本品在给药 0~10h 的镇痛效果，并与 100mg:10ml 的盐酸罗哌卡因注射液对比。

结果表明，给药体积相同(0.4ml)时，盐酸罗哌卡因注射液镇痛时长2h，长效罗哌卡因镇痛时长在4~8h范围中，其中300mg：10ml(300mg:10ml)、200mg:10ml(230mg:10ml)镇痛时长8h，200mg:10ml(200mg:10ml)镇痛时长4h。给药剂量相同(均约为12mg/只)时，盐酸罗哌卡因注射液组镇痛时长4h，此时有50%的动物出现死亡，50%的动物出现全身麻醉反应，在2h内均已恢复；长效罗哌卡因300mg：10ml(300mg:10ml)、200mg:10ml(230mg:10ml)、200mg:10ml(200mg:10ml)镇痛时长均为10h；各组配方对大鼠运动神经的阻滞作用与镇痛作用一致。另外还考察了200mg:10ml(200mg:10ml)在不同给药体积(0.4ml、0.6ml、0.8ml)的镇痛作用，0.6ml、0.8ml给药量镇痛时间较0.4ml明显延长。可见，相同给药容量和相同给药量时，长效罗哌卡因镇痛时间均较盐酸罗哌卡因长。各处方对大鼠运动神经的阻滞作用与镇痛作用一致。

3.1.2.3 本品对麻醉家兔神经传导的阻滞作用(规格：300mg:10ml)[6]

新西兰家兔 30 只，2.5±0.5Kg，随机分成 5 组，每组 6 只，分别为模型组(0.9%生理盐水)、溶剂对照组(空白溶剂)、甲磺酸罗哌卡因注射液组 1.2 mg/只(相当于罗哌卡因游离碱 0.89)、长效罗哌卡因注射液 1 组(0.89mg/只)、长效罗哌卡因注射液 2 组(3mg/只)。采用 5%异氟烷(异氟烷:氧)通过异氟烷雾化器将家兔迅速麻醉后，用 2%的异氟烷维持麻醉状态，暴露坐骨神经，用三根针灸针钩住，用缝合线固定在肌肉上，针和针之间距离保持在 1cm 左右，作为刺激电极，并插入参比电极和接受电极。测定空白值后，直接滴注 0.1ml 药液，将兔臀部抬高保持浸润状态 10min，缝合伤口。在近心端和远心端分别给予不同的电压刺激，待产生的波形稳定后，记录刺激电压、电信号从刺激电极到接收电极所需时间、以及电信号的波峰值。分别测定给药前以及给药后 5min、30min、1h、2h、4h、6h、8h、10h、12h、16h、20h、24h 的神经传导速度。

结果表明，阳性对照组给药后 5min 神经传导几乎完全抑制，药效持续 2~3h；试验组 1(0.89mg/只)、生理盐水和空白溶剂组均无效；试验组 2(3mg/只)药效起效稍慢，但能有效地

抑制神经冲动传导，药效持续时间为 12~16h。

结论：采用等体积的给药方式，长效罗哌卡因注射液与甲磺酸罗哌卡因注射液相比较，在给药剂量增加 3.37 倍的条件下，则相应的感觉神经阻滞时间延长 6~7 倍，具有很好的缓释作用。

3.1.2.4 本品和罗哌卡因水溶液在神经源疼痛模型上镇痛作用维持时间的比较(规格：300mg:10ml)[7]

采用 Wistar 大鼠，24 只，雄性，3~4w，L5/L6 脊神经结扎，建立大鼠神经源性疼痛模型，并且硬膜外插管。分为对照组、试验组、阴性组、空白溶剂组共 4 组，分别单次硬膜外给予 100μl 体积的甲磺酸罗哌卡因注射液(2.7mg/ml(相当于罗哌卡因游离碱 2mg/ml))、长效罗哌卡因注射液(300mg:10ml)、生理盐水、空白溶剂，于给药前后考察镇痛作用及持续时间。结果表明，生理盐水和空白溶剂对大鼠神经源性疼痛模型均不产生镇痛作用；甲磺酸罗哌卡因注射液和长效罗哌卡因注射液组均可以产生镇痛作用，效果显著，两药生物效应半衰期分别为 3.1±0.4h 和 8.3±1.1h($p<0.05$)，长效罗哌卡因注射液组的生物效应半衰期相比甲磺酸罗哌卡因注射液组延长了 1.7 倍。说明本品可以明显延长药物的作用时间。

3.1.2.5 本品对硬膜外麻醉持续时间和潜伏期的影响(规格：300mg:10ml)[8]

采用 Wistar 大鼠考察了长效罗哌卡因注射液对硬膜外麻醉持续时间和潜伏期的影响。Wistar 大鼠 24 只，雄性，3~4w，L5/L6 处硬膜外插管。分为阳性对照组 1、2、3，试验组 1、2、3 和阴性组共 7 组，分别单次硬膜外给予 60μl 的甲磺酸罗哌卡因注射液(1mg/ml(罗哌卡因碱基计))、甲磺酸罗哌卡因注射液(3mg/ml(罗哌卡因碱基计))、甲磺酸罗哌卡因注射液(10mg/ml(罗哌卡因碱基计))、长效罗哌卡因注射液(1mg/ml)、长效罗哌卡因注射液(3mg/ml)、长效罗哌卡因注射液(10mg/ml)、生理盐水。于给药前后考察镇痛作用及持续时间。结果表明，对照组甲磺酸罗哌卡因注射液(60、180 和 600μg)，双下肢瘫痪时间分别为 4.6、14.6 和 29.5min；长效罗哌卡因组(60、180 和 600μg)，下肢瘫痪持续时间分别为 15.4、53.3 和 237.2min；在扣除空白制剂作用时间后，试验组与阳性对照组相比较延长 3.3、3.6 和 8.1 倍；生理盐水不产生任何下肢瘫痪；空白溶剂组产生短暂的上下肢瘫痪，约 3.5min。结论：长效罗哌卡因注射液能够显著延长硬膜外麻醉作用，但对麻醉起效时间无明显影响。

3.1.2.6 皮下注射给予巴马小型猪对伤口愈合的影响试验(规格：300mg:10ml)[9]

巴马小型猪 12 只，2~3 月龄，8.28~10.95kg，普通级，雌雄各半。给药前，在动物双侧背部去除被毛，面积大约 40cm×20cm，将动物用戊巴比妥钠(30 mg/kg)静脉注射麻醉，备皮

处 75%酒精消毒，切割大小约 2cm×2cm 创面，背中心线两侧各 4 个，共 8 个创面，创面之间距离不得少于 8cm，用纱布止血。

用1ml注射器吸取供试品或对照品，距离创面边缘1cm处进针，向创面周围皮下给药，多点注射，每个注射点体积为0.5ml，各个注射点之间距离不得少于3cm。给药剂量和方法如下表：

动物号	注射区域	供试品/对照品	给药浓度(mg/ml)*	给药体积(ml/区域)
13-7301 ~ 13-7312	1	长效罗哌卡因注射液	30	2
	2	长效罗哌卡因注射液空白溶剂	0	2
	3	长效罗哌卡因注射液	30	1
	4	长效罗哌卡因注射液	30	0.5
	5	耐乐品	8.3	2
	6	氯化钠注射液	0	2
	7	耐乐品	8.3	1
	8	耐乐品	8.3	0.5

*：对照品及供试品剂量均以罗哌卡因计算，10mg盐酸罗哌卡因含有8.83 mg罗哌卡因

给药结束后，给小型猪裹上无菌纱布以保护创面，并且每天更换，持续 7 天。试验期间，对所有动物进行临床观察、给药局部观察及创面大小观察，于 D3(动物号：13-7301~13-7304)、D7(动物号：13-7305~13-7308)和 D14(动物号：13-7309~13-7312)对动物实施安乐死。保留创面周围和创面内部组织进行组织学检查。

临床观察结果：1 只动物(13-7309 号)于 D6~D8 及 D12 可见稀便，另 1 只动物(13-7312)于 D3~D8 可见稀便，这两只动物于 D11 可见软便。

给药局部观察结果：0.5ml长效罗哌卡因注射液给药局部：1只动物(1/12)D2可见红斑，D3~D7可见溃疡；2ml长效罗哌卡因注射液给药局部：2只动物(2/12)分别于D3~D10和D4~D10可见肿胀；2ml耐乐品给药局部：1只动物(1/12)D2可见红斑，D3~D8可见肿胀。其它给药局部未见异常反应。

创面大小观察结果：创口面积随时间延长逐渐减小，所有动物创口面积未见与供试品相关的异常改变。

病理学检查结果：

D3：各创面大体观察均未见异常；显微镜下观察发现，各创面边缘及中间部分均可见轻度至中度的炎症反应，且创面局部还可见轻微的表皮细胞再生。

D7：各创面大体观察均可见肉芽组织；显微镜下观察发现，各创面边缘及中间部分仍可见轻微至轻度的炎症反应，但已可见轻微至中度的肉芽组织形成，且创面局部还可见轻微至

中度的的表皮细胞再生。

D14：各创面大体观察均可见肉芽组织，且大部分可见不同程度的愈合；显微镜下观察发现，大部分创面边缘已愈合，完全由再生的表皮细胞所覆盖，但创面中间部分仍可见创口；此外，还可见轻微的炎症反应及肉芽组织形成，以及轻度至中度的瘢痕组织形成。

结论：在本试验条件下，长效罗哌卡因注射液、长效罗哌卡因注射液溶剂和耐乐品单次皮下注射给予小型猪，对伤口愈合无影响。

3.1.2.7 长效罗哌卡因注射液申报处方和改进处方对小型猪药效学及刺激性研究

3.1.2.7.1 长效罗哌卡因注射液申报处方(300mg:10ml)和改进处方2(200mg:10ml)对小型猪药效学及刺激性研究[10]

分别研究了长效罗哌卡因注射液申报处方(300mg:10ml)和改进处方 2 (200mg:10ml)给正常云南小型猪单次给予不同体积(0.6、0.8、1.0、1.2、1.4、1.6ml)，及其术后单次给予 1.0ml 的镇痛作用和注射部位的刺激性。

(1)对正常小型猪的镇痛作用和注射部位刺激性

云南雄性小型猪 4 只，7.1~8kg，背部和腹部备皮，脱毛膏脱净。背部和腹部皮肤间隔 6cm 以上画 6 个点，采用交叉方法，每个点单次皮下分别给予申报处方和改进处方 2 的药物 0.6、0.8、1、1.2、1.4、1.6ml，于 0、0.5、1、2、4、6、8、10、12、14、16、18、20、24h 分别观察注射部位的外观、触感、颜色、反应等，以及镇痛范围和镇痛时间。镇痛范围以给药点为圆心，隔 1~2mm 距离放射状散开进行测痛，得镇痛范围。

(2)对术后小型猪的镇痛作用和注射部位刺激性

云南雄性小型猪 6 只，7.1~8kg，背部和腹部备皮，脱毛膏脱净。每头猪的侧腹部皮肤消毒后用利多卡因表面涂抹麻醉，手术刀划开一长度为 1cm 的切口，深度到肌层，按压止血，用线缝合后消毒。对侧同样作一切口缝合作为对照。用注射器在伤口中心 4cm 的地方进针，分别给予受试药物 1.0ml。于 0、0.5、1、2、4、6、8、10、12、14、15、18、21、24、27、30h 分别观察注射部位的外观、触感、颜色、反应等，以及镇痛范围和镇痛时间。镇痛范围以给药点为圆心，隔 1~2mm 距离放射状散开进行测痛，得镇痛范围。

(3)结论

从申报处方和改进处方 2 的镇痛范围和镇痛时间来看，申报处方的镇痛范围大于改进处方 2，申报处方的各个容量的剂量组的镇痛范围在初期(10 小时以内)，镇痛范围基本都比改进处方高 15%左右；给药容量 1ml 以内，改进处方 2 对申报处方之间的镇痛时间基本没有差

异，但随着容量的提高，镇痛时间改进处方 2 对申报处方要缩短 20%左右。申报处方和改进处方 2 对于猪的背部皮肤注射后，都没有可见的红斑，肿块形成。

申报处方对手术创口的相对疼痛强度比改进处方 2 的相对疼痛强度要大一些(0.2 个相对单位)；在镇痛范围上，申报处方对手术创口的镇痛范围随着时间的延长也逐渐大于改进处方 2，且有效作用时间较长(24h/17h)，对手术创口的镇痛作用较好，对皮肤基本无刺激作用，对于手术创面的愈合过程无肉眼可见的影响。

腹部的注射肿块状可能和猪腹部皮肤较松弛，皮下结缔组织较多，药液不易散开吸收，所以提示注射时应避免结缔组织较多的区域，注射时注意使药液均匀分散，避免局部堆积结块。

3.1.2.7.2 **长效罗哌卡因注射液不同改进处方对小型猪药效学及刺激性研究**(200mg:10ml、230mg:10ml) [11]

分别研究了长效罗哌卡因注射液改进处方 1(230mg:10ml)和改进处方 2 (200mg:10ml)给正常云南小型猪单次给予不同体积(0.6、0.8、1.0、1.2、1.4、1.6ml)，及其术后缝合伤口和缺损皮肤单次给予 1.0ml 的镇痛作用和注射部位的刺激性。

(1)对正常小型猪的镇痛作用和注射部位刺激性

云南雄性小型猪 6 只，7.1~8kg，背部备皮，脱毛膏脱净。背部皮肤间隔 6cm 以上画 6 个点，采用交叉方法，每个点单次皮下分别给予申报处方和改进处方 2 的药物 0.6、0.8、1、1.2、1.4、1.6ml，于 0、0.5、1、2、4、6、8、10、12、14、16、18、20、24h 分别观察注射部位的外观、触感、颜色、反应等，以及镇痛范围和镇痛时间。镇痛范围以给药点为圆心，隔 1~2mm 距离放射状散开进行测痛，得镇痛范围。

(2)对术后小型猪的镇痛作用和注射部位刺激性

云南雄性小型猪 6 只，7.1~8kg，腹部备皮，脱毛膏脱净。每头猪的侧腹部皮肤消毒后用利多卡因表面涂抹麻醉，手术刀划开一长度为 1cm 的切口，深度到肌层，按压止血，用线缝合后消毒。对侧同样作一切口缝合作为对照；用注射器在伤口中心 4cm 的地方进针，给予受试药物 1.0ml，在切口两侧做皮下浸润注射。

另在切口旁 5cm 处，用手术刀切开 2 个直径为 0.5cm 的皮肤缺损，深度到肌层，按压止血，对侧同样作一切口作为对照。一缺损部位用注射器在距中心 4cm 的地方进针，给予受试药物 1ml；另以缺损部位用注射器在伤口中心处滴加 100μl 的受试药物，尽量涂抹均匀，直径

严禁复制

不超过 2cm。

分别于 0、2、4、6、8、10、12、14、16、18、20、24、26h 分别观察注射部位的外观、触感、颜色、反应等，以及镇痛范围和镇痛时间。镇痛范围以给药点为圆心，隔 1~2mm 距离放射状散开进行测痛，得镇痛范围。

(3)结论

从改进处方 1 和改进处方 2 的镇痛范围和镇痛时间来看，改进处方 1 的镇痛范围大于改进处方 2，改进处方 1 的各个容量的剂量组的镇痛范围在初期(18h 以内)，镇痛范围基本都比改进处方 2 高 20%左右；镇痛时间改进处方 2 对改进处方 1 要缩短 24%左右。改进处方 1 和改进处方 2 对于猪的背部皮肤注射后，都没有可见的红斑形成。

改进处方 1 对手术创口的相对疼痛强度比改进处方 2 的相对疼痛强度要大一些；在镇痛范围上，改进处方 1 对手术创口的镇痛范围随着时间的延长也逐渐大于改进处方 2，且有效作用时间较长(22h/14h)，对手术创口的镇痛作用较好，对皮肤基本无刺激作用，对于手术创面的愈合过程无肉眼可见的影响。

改进处方 1 和改进处方 2 在猪的背部皮肤注射后，局部会形成硬皮样状态，对外界的刺激麻木，但不能很好的分辨对疼痛的耐受和这种硬皮样变化有什么区别联系，硬皮样状态可能和皮下药液积存导致皮肤紧绷有关，随药液的吸收，硬皮样状态会逐渐缓解，部分区域在 24~48 消失后有硬块样触摸感，这个和猪背部的生理结构可能有一定关系，靠近臀部的皮肤不易显现这种硬皮样状态，应该和皮肤的松弛度有关，另外改进处方 2 的硬块样触摸感要小于改进处方 1，可能和两者成分的不同有一定关系。

皮肤缺损处涂抹改进处方 1 和改进处方 2，两小时后涂抹处伤口血液已凝固，自然风干，伤口没有明显红肿，自然结痂，和空白组相比无明显差异。24h 后伤口结痂，伤口没有明显红肿，和空白组相比无明显差异，表明表面涂抹样品对动物皮肤缺损愈合没有影响。

3.2 一般药理及毒理学研究

3.2.1 单次皮下注射给药对清醒无束缚 Beagle 犬的心血管及呼吸系统功能的影响(规格：300mg:10ml)[12]

研究了长效罗哌卡因注射液单次皮下注射给药后对清醒无束缚Beagle犬的心血管及呼吸系统功能的影响。8 只Beagle犬(雌雄各半，普通级)，采用双拉丁方实验设计，设试验组 15、30、60mg/kg三个剂量组，一个溶媒组(长效罗哌卡因注射液空白溶剂)及一个阴性对照组(氯化

钠注射液)，单次皮下注射给药。给药轮次间隔 5 日。

轮次	动物号							
	F1	F2	F3	F4	M1	M2	M3	M4
1	高剂量	中剂量	低剂量	溶媒	高剂量	中剂量	低剂量	阴性
2	中剂量	低剂量	溶媒	阴性	中剂量	低剂量	溶媒	高剂量
3	低剂量	溶媒	阴性	高剂量	低剂量	溶媒	阴性	中剂量
4	溶媒	阴性	高剂量	中剂量	溶媒	阴性	高剂量	低剂量
5	阴性	高剂量	中剂量	低剂量	阴性	高剂量	中剂量	溶媒

于给药前 1h内，给药后 1h(±10 min)、2h(±10 min)、3h(±10 min)、4h(±30 min)、5h(±30 min)、6h(±45 min)、10h(±45 min)、14h(±45 min)、18h(±1h)、24h(±1h)、48h(±1h)时间点，采集动物心电、血压、呼吸及体温各项指标的数据。对心电、血压、呼吸及体温等的各指标数据进行统计学分析和评估。结果表明，长效罗哌卡因注射液各剂量组及长效罗哌卡因注射液空白溶剂组动物心电指标(心率、RR间期、PR间期、QRS时限、QRS电压、QT间期、QTcF间期、T_{p-e}间期、ST段电压、T波高度、P波高度、P波宽度)、血压指标(收缩压、舒张压、平均动脉压、脉压差)、呼吸功能指标(呼吸频率、潮气量)及体温指标，与阴性相同时间点各指标比较均无统计学差异，也无趋势性改变。**结论：**在本试验条件下，长效罗哌卡因注射液皮下注射给药在 15、30、60 mg/kg剂量下对清醒Beagle犬心血管、呼吸系统功能及体温无明显影响。

3.2.2 单次皮下注射给药对SD大鼠中枢神经系统功能的影响(规格：300mg:10ml)[13]

评价了长效罗哌卡因注射液单次皮下注射给药对 SD 大鼠中枢神经系统功能的影响。方法：试验用 SD 大鼠 50 只(25 只/性别)，按照性别根据体重进行区段随机分组，经电脑随机过程产生随机号，按随机号排序后分配到 5 个组，每组 10 只(5 只/性别)。单次颈背部皮下注射给药，单点或多点注射，每点不超过 1ml，给药时每组取一只动物按轮次依次给药，给药顺序为 A、B、C、D、E。分别给予阴性对照品(氯化钠注射液)、长效罗哌卡因注射液空白溶剂及 30、60、120mg/kg 剂量供试品(长效罗哌卡因注射液)，给药容量分别为 4、4、1、2、4ml/kg。盲法设计，给药信息对参与打分的技术人员保密，于给药前一天，药后 1 h、24h 由两名独立观察并评分的实验观察者，采用功能组合观察法对动物进行观察并记录结果。功能组合观察法内容包括：笼内观察、手抓观察、开放环境观察、刺激性反应观察，对前肢爪抓力、后肢

张开幅度、体温进行测定。两名独立实验观察者的数据求均值后进行统计分析。

结果：与阴性对照组相比，药后 1 h、24 h 溶剂组及供试品各剂量组动物笼内观察，手抓观察，开放环境观察，刺激性反应，及前肢爪抓力、后肢张开幅度、体温测定均未见明显差异。

结论：在本试验条件下，长效罗哌卡因注射液在 30、60、120mg/kg 剂量下单次皮下注射给药对 SD 大鼠中枢神经系统功能无明显影响。

3.3 毒理学研究

3.3.1 毒理学文献[14]

3.3.1.1 急性毒性

只有在高剂量或意外将药物注入血管内而使药物血浆浓度骤然上升或者在药物过量的情况下，罗哌卡因会造成急性毒性反应(参见盐酸罗哌卡因注射液（耐乐品）说明书“药理作用”和“药物过量”)。曾有一例患者因作臂丛神经阻断时，无意中将200mg药物注入血管内后，发生惊厥。

3.3.1.2 生殖毒性

生殖毒性接受试验的两代大鼠未见生育力及一般生殖行为受药物的影响。施用最高剂量的盐酸罗哌卡因后，因其对孕母的毒性作用，产后三天内幼仔的死亡数增多，列于新生仔死亡的第二位原因。对大鼠和兔所进行的致畸试验未见罗哌卡因对器官发生以及胎儿早期发育有任何不利影响，以最大可耐受剂量对围产期及产后的大鼠进行研究，未见其对胎儿后期发育、分娩、哺乳、新生儿生存力及子代的生长有任何影响。

妊娠关于孕妇使用罗哌卡因后对胎儿生长的影响尚无临床试验，建议慎用。分娩时使用罗哌卡因作为产科麻醉或镇痛已有充分的实验报告，未见任何副作用。哺乳在人乳中罗哌卡因或其代谢物的分泌状况未曾研究。根据大鼠实验中乳汁/血浆浓度的比值，估计幼鼠日摄入量为其母鼠剂量的 4%。假设人类乳汁/血浆浓度比值与大鼠相同，则母乳哺育的婴儿所摄入罗哌卡因的量较妊娠时在孕妇子宫中接受的剂量要低得多。

3.3.1.3 致突变

文献表明，在小鼠淋巴瘤试验中，发现罗哌卡因具有弱的致突变活性，但是在其他的动物试验中没有发现，可能是体外试验(小鼠淋巴瘤试验)与在体实验的差异所致。

严禁复制

3.3.2 毒性试验

3.3.2.1 单次皮下注射大鼠急性毒性和体内代谢试验(规格：300mg:10ml)[15]

评价了长效罗哌卡因注射液单次皮下注射给予SD大鼠后的急性毒性反应及其在体内的代谢情况。204只大鼠分为5个毒理研究组(1~5组)和3个毒代研究组(6~8组)，1~5组每组雌雄各15只，6~8组每组雌雄各9只。第1组给予生理盐水10ml/kg，第2组给予长效罗哌卡因注射液溶剂10ml/kg，3组和6组，4组和7组，5组和8组给予长效罗哌卡因注射液，给药剂量分别为75、150、300mg/kg，单次给药，采用颈背部皮下注射。1~5组每组前5只动物/性别于D3实施安乐死，剩余动物于D15实施安乐死，6~8组动物在毒代血样采集完成后实施安乐死。

D1~D3，毒理研究组中共有12只动物死亡(1只雄性和11只雌性)，2组的2只雌性，5组的1只雄性和9只雌性。其中，5组有5只动物于药后3h内死亡，1只与药后8h死亡，2组和5组的其他动物在药后1天或2天死亡。D1~D4，毒代研究组中第8组共有13只动物死亡，包括6只雄性和7只雌性。

与给药相关的死亡动物的临床表现有：呼吸急促，弓背，俯卧，活动减少，全身颤动，前肢和/或后肢无力，眼、鼻分泌物，昏睡和被毛蓬松，组织病理学检查未见明显的致死性病变。

试验期间：2~5组存活动物与药物相关的临床表现主要有：呼吸急促、兴奋/烦躁、弓背、俯卧、活动减少、颤动、前肢和/或后肢无力、眼分泌物、鼻分泌物、昏睡、被毛蓬松、以及给药局部的破溃和结痂等，以上的一些临床表现随着观察的延长消失。

试验期间，与阴性对照组相比，5组雄性动物的体重于D4，D7，D11降低，2组雌性动物体重于D4降低。2、5组雄性和2组雌性D1~D4的食量降低。

血细胞计数：D3和D15，2~5组雌性和/或雄性动物的Neut、Mono升高，Lymph降低，认为可能是供试品引起的继发性反应。血液生化：2~5组雌性和/或雄性动物的Alb和/或A/G降低，D15恢复。另外，5组雌性动物D3的ALT、AST、TBIL、BUN、CREA、TG升高。

雌、雄大鼠给药(75、150、300 mg/kg)后，体内罗哌卡因的暴露量与剂量呈正相关。在75~300 mg/kg剂量范围内，雌性大鼠和雄性大鼠体内罗哌卡因的平均Cmax比率在1.21~1.38之间，平均AUC(0~24h)比率在1.17~1.35之间，罗哌卡因在不同性别动物体内的暴露量基本无差异。

D3：2~5组多数动物注射局部可见肿胀、暗红色区域和/或透明/淡黄色液体，镜检可见轻微至中度的急性/亚急性炎症，组织学特点为大片坏死伴炎细胞浸润，伴或不伴出血。D15：

2~5组多数动物注射局部可见暗红色区域及透明/淡黄色液体，镜检可见轻微至中度的慢性炎症，组织学特点为肉芽组织形成或纤维化，伴或不伴组织坏死，为一种修复过程。以上改变认为与长效罗哌卡因注射液溶剂相关。另外，D3：5组部分安乐死动物肝脏可见轻微或轻度的小叶中心性肝细胞肥大，肾脏可见轻微或轻度的嗜碱性肾小管，认为此病变可能与供试品相关，但程度轻微且可恢复。

在本试验条件下，长效罗哌卡因注射液以 75，150和300mg/kg 的剂量单次皮下注射给予SD大鼠，溶剂对照组(10ml/kg)和供试品300 mg/kg剂量组均有动物死亡，由于溶剂对照组和供试品高剂量组给药容量相同，因此认为溶剂对照组动物死亡可能与溶剂相关，供试品高剂量组动物死亡可能与供试品和溶剂共同作用相关。动物最大耐受剂量为150mg/kg，在该剂量下，药物的AUC(0~24h)为21.09h·μg/ml，Cmax为1582.14ng/ml。未观察到明显的毒性反应剂量(NOAEL)为75mg/kg，在该剂量下，药物的AUC(0~24h)为11.65 h·μg/ml，Cmax为1237.13 ng/ml。

3.3.2.2 **空白溶剂单次皮下注射给予SD大鼠后的急性毒性反应(规格：300mg:10ml)**[16]

90只SD大鼠，共分为3组(15只/性别/组)，第1组给予生理盐水5ml/kg，第2组、3组给予长效罗哌卡因注射液溶剂，给药容量分别为2.5、5ml/kg，单次给药，采用颈背部皮下注射。1~3组每组前5只动物/性别于D3实施安乐死，剩余动物于D15实施安乐死。

试验期间，各组动物均未出现死亡或濒死现象。

试验期间，给药组动物的注射局部于D2~D15可见与给药相关的异常表现，包括破溃和结痂：2组(3/10雄性、4/10雌性)和3组(2/10雄性4/10雌性)。

试验期间，各组动物的体重、食量、血细胞计数、血凝、血生化未见与药物相关的毒理学意义的改变。

D3和D15，各组安乐死动物的脏器重量均未见与药物相关的改变，大体解剖观察和显微镜下观察均未见系统性的病理改变，2~3组多数动物注射局部皮下可见淡黄色或透明油状液体，D3镜下观察可见轻微到中度的皮下亚急性炎症，D15镜下观察可见轻微到中度的皮下慢性炎症，伴或不伴肉芽组织形成/纤维化，有恢复趋势。

在本试验条件下，长效罗哌卡因注射液溶剂单次皮下注射给予SD大鼠，动物耐受容量高达5ml/kg，此剂量下除注射局部有异常表现外，未观察到系统毒性，因此认为最大的给药耐受容量≥5ml/kg。

3.3.2.3 **单次皮下注射给予Beagle犬后的急性毒性反应及其在体内的代谢(规格：**

300mg:10ml)[17]

50只Beagle犬，共分为5组(5只/性别/组)，第1组给予氯化钠注射液2ml/kg，第2组给予长效罗哌卡因注射液溶剂2ml/kg，第3、4、5组给予长效罗哌卡因注射液，给药剂量分别为15、30、60mg/kg，单次给药，采用颈背部皮下注射。1~5组每组前2只动物/性别于D3实施安乐死，剩余动物于D15实施安乐死。

试验期间，各组动物均未出现死亡或濒死现象。

试验期间，2~5 动物的注射局部于D2~D15可见与给药相关的异常表现，包括肿胀(1/10，1/10，0/10，1/10)、红斑(0/10，0/10，1/10，0/10)、硬结(0/10，0/10，1/10，1/10)、破溃(1/10，1/10，0/10，2/10)和结痂(2/10，2/10，1/10，2/10)，各组动物给药局部的异常改变及发生率基本一致。

试验期间，各组动物的体重、体温、心电图参数、血细胞计数、血凝、血生化未见与药物相关的毒理学意义的改变。

在15~60 mg/kg剂量范围内，平均Cmax及AUC(0~48h)值随剂量增加而增大，雄犬和雌犬体内罗哌卡因的平均Cmax比率在1.03~1.35 之间，平均AUC(0~48h)比率在1.04~1.27 之间，罗哌卡因在不同性别动物体内的暴露量基本无差异。

D3和D15，各组安乐死动物的脏器重量均未见与药物相关的改变，大体解剖观察和显微镜下观察均未见系统性的病理改变。D3：2、3、5组部分动物注射局部可见暗红色区域，镜下观察可见轻微到中度急性皮下炎症，其特点为坏死为主，伴有出血和炎细胞浸润；D15：2~5组多数动物注射局部可见暗红色/黄色区域或白色/黄色结节，镜下观察可见轻微到中度慢性皮下炎症，伴有肉芽组织形成或纤维化，伴有残留的坏死组织。慢性炎症伴肉芽组织形成或纤维化是一种修复过程，提示急性炎症是可恢复的。

在本试验条件下，长效罗哌卡因注射液单次皮下注射给予Beagle犬，动物耐受剂量高达60mg/kg，此剂量下未观察到系统毒性。给予长效罗哌卡因注射液溶剂和长效罗哌卡因注射液动物均可见到注射局部的异常表现，认为与溶剂相关。本试验研究结果认为未观察到明显的毒性反应剂量(NOAEL)≥60mg/kg。在该剂量下，药物的$AUC_{(0\sim48h)}$为66.10μg.h/ml，Cmax为2.09μg/ml。

3.4 非临床药代动力学

3. 4. 1 药代动力学文献资料

3.4.1.1 吸收与分布

文献报道，罗哌卡因的 pKa 为 8.1，分布率为 141(25℃n-辛醇/磷酸盐缓冲液 pH7.4)。 罗哌卡因的血浆浓度取决于剂量、用药途径和注射部位的血管分布。罗哌卡因符合线性药代动力学，最大血浆浓度和剂量成正比。罗哌卡因从硬膜外的吸收是完全的，呈双相性，快相半衰期为 14 分钟，慢相终末半衰期约为 4 小时。因缓慢吸收是清除罗哌卡因的限速因子，所以硬膜外用药比静脉用药清除半衰期要长。罗哌卡因总血浆清除率 440ml/min。游离血浆清除率为 8L/min。肾清除率为 1ml/min，稳态分布容积为 47L，终末半衰期为 1.8h。罗哌卡因经肝脏中间代谢率为 0.4。罗哌卡因在血浆中主要和α1-酸糖蛋白结合，非蛋白结合率约 6%。 当连续硬膜外注射时，可观察到罗哌卡因总的血浆浓度的增加和手术后α1-酸糖蛋白浓度的增加有关，未结合的(药理学活性)浓度的变化比总血浆浓度的变化要小得多。

3.4.1.2 代谢与排泄

罗哌卡因结构中的酰胺键因其周围具有大芳香环和 R 上正丙取代基的空间保护而不易降解，生物转化作用主要体现于肝脏混合功能氧化酶对哌啶环到 PPX 的脱烃作用，继而通过与布比卡因代谢产物 PPX 相同的途径代谢，其体内主要经肝脏代谢，相应的代谢产物为 3-羟基罗哌卡因、4-羟基罗哌卡因、PPX，约有 1% 以原形、37%以 3-羟基罗哌卡因结合物形式经泌尿系统排泄，尿液中排出的 4-羟基罗哌卡因，N-去烷基代谢物和 4-羟基罗哌卡因代谢物约为 1~3%。结合的和非结合的 3-羟基罗哌卡因在血浆中仅显示可测知的浓度，3-羟基罗哌卡因和 4-羟基罗哌卡因均有一定的局麻作用，但作用较弱。人体研究结果显示，静脉注射罗哌卡因 50mg，连续测定 96h，约有 86%经尿排出，8%粪便排出，尿液中主要成分为 3-羟基-罗哌卡因(37%)、PPX(3%)、罗哌卡因(1%)、4-羟基-罗哌卡因(0.4%)以及 3-羟基-PPX(2%)，给药后的前 24h 有 80%经尿液排泄。罗哌卡因在体内没有消旋作用的证据。

3.4.2 非临床药代动力学试验资料(规格：300mg:10ml)[18]

研究了 Beagle 犬皮下注射长效罗哌卡因注射液的药代动力学，并与已上市药物盐酸罗哌卡因注射液进行了比较。实验研究采用了交叉试验设计，动物选用 Beagle 犬 12 只，雌雄各半，按性别随机分为 2 组。给药方式为单次皮下注射，剂量均为 10mg/kg。结果表明，皮下注射供试品后，于药后 0.083h 即检测到药物，Tmax 在 8~12h 之间，大部分动物药后 48h 基本检测不到药物；犬皮下注射对照品后，于药后 0.083h 即检测到药物，Tmax 在 0.083~0.25h 之间，大部分动物药后 24h 基本检测不到药物。供试品组与对照品组比较，供试品组平均 C_{max} 明显降低，平均 T_{max}、MRT_{last} 明显延迟，显示供试品在血浆中持续的时间长于对照品，结果

如下表显示：

组别		$t_{1/2}$ h	T_{max} h	C_{max} μg/ml	AUC_{last} h·μg/ml	AUC_{inf} h·μg/ml	Vd L/kg	CL L/h/kg	MRT_{last} h
供试品组	Mean	4.54	11.00	0.58	9.02	9.21	7.98	1.22	10.62
	SD	0.85	1.60	0.25	3.64	3.60	3.07	0.40	1.20
对照品组	Mean	3.06	0.22	2.76	7.93	8.26	5.69	1.29	3.87
	SD	0.96	0.07	1.09	2.09	2.15	2.39	0.34	1.17

3.5 非临床皮肤刺激、致敏、溶血等安全性试验资料

3.5.1 200mg:10ml前的300mg:10ml规格(规格：300mg:10ml)

3.5.1.1 致敏试验[19]

通过豚鼠反复接受供试品后，观察是否出现速发型过敏反应。试验动物豚鼠30只分为5组，分别设定为阴性组，人血白蛋白阳性组(30mg/kg致敏，60mg/kg激发)，予低剂量长效罗哌卡因注射液组(6mg/kg致敏，12mg/kg激发)、高剂量长效罗哌卡因注射液组(30mg/kg致敏，60mg/kg激发)以及相应的长效罗哌卡因注射液溶剂组，激发后结果显示长效罗哌卡因注射液溶剂、长效罗哌卡因注射液低和高剂量组所有动物未见过敏反应症状，过敏反应均为阴性。因此认为6或30mg/kg长效罗哌卡因注射液隔日一次皮下致敏连续3次后，12或60mg/kg长效罗哌卡因注射液单次腹腔注射激发给予豚鼠，未出现全身主动过敏性反应。

3.5.1.2 溶血试验[20]

长效罗哌卡因注射液，按不同体积(0.1、0.2、0.3、0.4 和0.5ml)依次加入含有不同体积的氯化钠注射液(补足容量至2.5ml)和2.5ml的2 %红细胞混悬液的玻璃管中(总体积为5ml)，置于37℃±0.5℃条件下孵育3小时。同时以2.5ml的长效罗哌卡因注射液溶剂、耐乐品、氯化钠注射液和灭菌注射用水分别作为溶媒对照、市售制剂对照、阴性对照和阳性对照。观察结果显示，阳性对照试管中出现红细胞部分溶血而阴性对照管中无溶血和凝聚，长效罗哌卡因注射液和长效罗哌卡因注射液溶剂管中分别可见部分溶血和完全溶血。

因此，可认为长效罗哌卡因注射液溶血原因是由于其溶剂所致，其主药罗哌卡因游离碱无此项作用。

3.5.1.3 刺激性试验[21]

评价长效罗哌卡因注射液单次皮下注射给予家兔的局部刺激作用，以及停药 14 天后刺激的恢复情况，12 只家兔根据体重随机分为 2 组，1 组动物左右后肢分别给予长效罗哌卡因注

射液溶剂和长效罗哌卡因注射液，2 组动物左右后肢分别给予氯化钠注射液和耐乐品，单次给药，两点注射，注射体积分别为 1ml 和 0.5ml。

长效罗哌卡因注射液注射局部：D1~D3，注射局部观察发现，1/6 动物于 0.5 ml 注射局部可见轻微红斑；大体观察未见异常；显微镜下观察发现，2/3 的动物右后肢注射局部可见轻微的急性/亚急性皮下炎症；恢复期结束(D15)，2/3 的动物右后肢注射局部可见轻微或轻度肉芽组织形成/纤维化，1/3 的动物右后肢注射局部可见轻微的亚急性皮下炎症。

长效罗哌卡因注射液溶剂注射局部：D1~D3，注射局部观察发现，1/6 动物于 1ml 注射局部可见溃疡，1/6 动物于 1ml 注射局部可见结痂；大体观察发现，2/3 的动物注射局部可见鲜红色或暗红色区域；显微镜下观察发现，3/3 的动物左后肢注射局部可见轻微或轻度的急性/亚急性皮下炎症，特点为皮下组织的单个核细胞浸润和出血，伴或不伴明显的坏死；恢复期结束(D15)，2/3 的动物左后肢注射局部可见皮下肉芽组织形成/纤维化，程度为轻微或轻度，1/3 的动物左后肢注射局部可见单个核细胞浸润，程度轻微。

因此，长效罗哌卡因注射液单次皮下注射给予新西兰兔，对给药局部有轻微刺激性，可能与溶剂相关，停药后刺激性反应可恢复。

3.5.2改进处方(200mg:10ml)

根据临床批件建议，以进一步减小对注射部位的刺激性为目标，对本品的处方进行了进一步优化。

3.5.2.1 不同处方的刺激性考察试验[22-24]

新西兰白兔，普通级，体重 3~4 Kg，脊柱两侧剃毛(约 10cm×7cm)，标记，两个注射点间距离不少于 5cm。24h 后，单次皮下注射给药，每个点注射 1.0ml。给药当日 2h、6h 及日后每日上午和下午分别观察一次，共 14 天。根据评分标准对局部组织进行评分；药后第 3、7 日分别处死两只动物进行病理检查，按照病理评分标准对刺激性进行评分。不同处方如下表。

处方	300mg：10mlK	200mg:10ml								
		K1	K2	K3	K4	K5	K6	K7	K8	K9
载药量 mg/ml	33.04	30.64	28.24	25.84	24.44	18.64	28.88	24.72	16.40	2.00
苯甲醇	8%	8%	8%	8%	8%	8%	6%	4%	0%	0%
苯甲酸苄酯	30%	25%	20%	15%	10%	0	30%	30%	30%	0%
大豆油	62%	67%	72%	77%	82%	92%	64%	66%	70%	100%

6 只动物随机分为 3 组，先将申报处方 K,改进处方 K4、K8、K9 四个处方顺时针自背部右前开始，每个点注射 1.0ml 后，参照上述时间考察。结果显示处方 K8 连续观察 3 天、7 天、

14 天的评分为 0，评分明显低于 300mg：10mlK。处方 K9 连续观察 3 天时的评分 0.1±0.3，连续观察 7 天、14 时的评分为 0，评分明显低于 300mg：10mlK。处方 K、K4 在连续观察 3 天、7 天、14 的评分相差不明显。同时病理结果显示。处方 K8、K9 的评分最低，明显低于 300mg：10mlK 的评分。K4 的评分高于 K8、K9，也低于 300mg：10mlK。

结论：配方 K8(含苯甲酸苄酯和大豆油)和配方 K9(含大豆油)无明显刺激性，说明苯甲酸苄酯和大豆油不是主要的刺激性物质，配方 K4 刺激性略轻于配方 K。

在上述试验的基础上，8 只动物随机分为 2 组，脊柱两侧剃毛(约 15cm×7cm)，背部划分为右前、右中、右后、左后、左中、左前六个区域标记。24h 后，单次皮下注射给药，每只兔子自前、中、后交替给药注射 K、K2、K5、K6、K7 各 1.0ml，及 K0.86ml，两个注射点间距离不少于 3cm，盐水组另取部位给药 1.0ml。于给药当日 2h、6h 及日后每日观察一次，共 7 天。根据评分标准对局部组织进行评分；给药后第 3、7 日分别处死四只动物进行病理检查，按照病理评分标准对刺激性进行评分。处方 K、K2、K5、K6、K7、K 给药 0.86ml 皮下注射后，处方 K7 刺激性评分最小，明显小于 300mg：10mlK。结论：配方 K7 刺激刺激性显著小；配方 K2、K5、K6 和 K 刺激性均高于 K7，可见苯甲醇是产生刺激性的主要因素。

上述两个不同辅料处方刺激性试验结果表明，苯甲醇是刺激性来源的主要因素，但也是影响载药量的主要因素。因此，继续考察 K7 载药量与刺激性的关系。鉴于之前给药量为 1.0ml/只，有一定刺激性，本次实验拟降低药量，试图找到无明显刺激的最大药量。设计了如下不同处方：

处方	K	K6	K7	K10
预测载药量 mg/ml	33.04	28.88	24.72	
苯甲醇	8%	6%	4%	5%
苯甲酸苄酯	30%	30%	30%	30%
大豆油	62%	64%	66%	65%

16 只新西兰白兔，背部剃毛，背部划分为右前、右后、左前、左后四个区域，分为 4 组。24h 后，第 1 组每只动物分别在 4 个部位皮下注射 0.8ml 的 K、K6、K7、K10；第 2 组每只动物分别在 4 个部位皮下注射 0.6ml 的 K、K6、K7、K10；第 3 组每只动物分别在 4 个部位皮下注射 0.4ml 的 K、K6、K7、K10；第 4 组每只动物分别在 4 个部位皮下注射 0.2ml 的 K、K6、K7、K10；每只动物同时另取部位皮下注射生理盐水作为对照。给药当日 2h、6h 及日后每日观察一次，共 7 天。根据评分标准对局部组织进行评分，并于第 7 日全部处死进行病理检查，按照病理评分标准对刺激性进行评分。结果表明，各处方给药量 0.8ml 时有明显刺

激性，而随着给药量的减小，在 0.2~0.6ml 时刺激性明显降低。300mg：10mlK 的刺激性大于 200mg:10mlK6、K10 和 K7。而处方 K7 刺激性评分最低，在 0.2、0.4 和 0.6ml 评分几乎为 0，在 0.8ml 时评分仅为 0.1±0.2，低于其他处方；病理结果显示各个配方随给药量减小，刺激性减轻，配方 K7 尤为明显，在 0.2、0.4ml 评分分别为 0.8±0.5 和 1.0±0.0。结论：改进处方 K7 的刺激性最小，申报处方 K 的刺激性大，K6、K10 的刺激性介于 K7 与 K 之间。

综上所述，处方中的主要刺激性物质为苯甲醇，处方 K7，即苯甲醇量为 4%时，刺激性最小，且能达到有效剂量。

3.5.2.2 改进处方(200mg:10ml)制剂安全性评价

上述的刺激性试验结果表明，降低处方中主要产生刺激性的因素——苯甲醇用量，可明显的降低本品的刺激性，但同时降低了罗哌卡因的溶解度，导致载药量降低。因此，结合药效学试验研究，确定本品的规格为 200mg:10ml，理论上推测其刺激性要明显好于前期申报的处方规格 300mg:10ml。其制剂安全性评价(刺激、溶血和过敏试验)将在随后按照 GLP 要求进行。

3.6 临床前研究的综合评价

罗哌卡因（ropivacaine）是阿斯利康制药公司 1992 年开发的一类新的长效酰胺类局部麻醉药，它主要通过阻断钠离子流入神经纤维细胞膜内对沿神经纤维的冲动产生可逆性阻滞而发挥作用，作用机理同其它酰胺类的局部麻醉药相同，该药物于 1996 年在荷兰首次上市，1998 年我国批准进口，目前已过专利有效期，国内有多家生产单位，主要的给药剂型是罗哌卡因盐（甲磺酸、盐酸）的普通水溶液。

普通罗哌卡因盐的水溶液已经成为临床局部麻醉一线用药，在术后镇痛方面，罗哌卡因盐的水溶液主要通过局部神经阻滞与浸润麻醉的方式广泛的用于临床妇产科、骨科、普通外科等众多科室，镇痛效果良好、相对不良反应少。

在有效性方面，目前临床资料表明，在安全的给药剂量范围内，罗哌卡因术后手术部位局部神经阻滞镇痛有效持续时间仅为 8-24 小时，而临床患者术后疼痛高峰期为术后的前 3 天，普通罗哌卡因水溶液单次注射药效远远不能满足患者的镇痛要求，长效罗哌卡因注射液与罗哌卡因盐的水溶液动物实验药效结果相比较，动物局部给药量增加 3.4 倍左右时，药效增加 3-6 倍左右，可覆盖临床术后疼痛高峰期。因此，西安力邦制药有限公司研发的长效罗哌卡因注射液具有重要的临床意义。

在安全性方面，长效罗哌卡因注射液主药采用了罗哌卡因的游离碱形式，溶媒系统包括苯甲醇、苯甲酸苄酯、维生素 E 和大豆油，主药和辅料均有临床注射历史，因此其遗传、生殖毒性无问题，由于长效罗哌卡因注射液临床设计剂量无论是主药还是辅料均超出了目前临床使用剂量，因此安全性评价方面给与重点关注；急性毒性方面采用了大鼠和犬两种属动物进行评价同时考察了制剂的血液、生化、主要脏器的病理变化等指标，并同时进行了 TK 试验实验结果显示在目前设计的给药剂量条件下，制剂安全性很好。刺激性、伤口愈合性表明长效罗哌卡因注射液有可恢复的局部刺激性并对伤口愈合不产生影响，过敏反应无影响，PK 实验结果显示长效罗哌卡因注射液与普通制剂等剂量给药后还可大幅降低 T_{max} 并延长体内的 MRT_{last}，降低了罗哌卡因盐的水溶液因快速释放所引起的心血管毒性和神经毒性。总之，长效罗哌卡因注射液与普通制剂相比较，安全性有很好的保证，完全可以满足临床要求。

4 研究目的

4.1 主要目的

1) 考察长效罗哌卡因注射液不同剂量单次皮下给药在健康受试者中的安全性和耐受性；
2) 考察长效罗哌卡因注射液单次皮下给药在健康受试者中的药代动力学。
3) 确定长效罗哌卡因注射液单点腹部皮下推荐给药剂量。

4.2 次要目的

1） 考察长效罗哌卡因注射液单次皮下给药在健康受试者中的药效。
2） 考察镇痛起效时间、持续时间、镇痛强度，确定最佳给药间距。

5 受试人群

5.1 入选标准

1) 充分了解试验目的和要求，自愿参加并签署经伦理委员会批准的知情同意书者；
2) 年龄为 18-50 周岁（包括 18 周岁和 50 周岁）的中国成年男性和女性，男女均有；
3) 体重指数（BMI）在 19.0~26.0 kg/m^2（包括临界值）；BMI=体重（kg）/身高^2（m^2）；

5.2 排除标准

1) 对本试验药物或试验药物中任何成分或同类药物过敏者，或属于过敏性体质者；
2) 生命体征检查、体格检查、临床实验室检查（血常规、尿常规、血生化、血清学检查等）、12 导联心电图检查，结果经研究者判断异常且有临床意义者；
3) 有肝脏、肾脏、呼吸、血液或淋巴、内分泌、免疫（包括 HIV 检测阳性或其它免疫缺陷疾病等）、精神、胃肠道系统等慢性疾病史或严重疾病史，或有可能影响药物

吸收、分布、代谢、排泄的手术或疾病者；

4) 经评估有潜在困难气道风险者；既往及当前有支气管哮喘、慢性阻塞性肺病、睡眠呼吸暂停综合征等呼吸系统疾病者；
5) 既往及当前有体位性低血压、心律失常、高血压、癫痫等心脑血管疾病者；
6) 具有长 QT 综合征或其家族史（祖父母、父母和兄弟姐妹），或 QTc 间期＞450 ms 者；室内传导阻滞或左右束支传导阻滞和/或 QRS＞120ms；室性异位搏动频发（筛选期任意一次 10s ECG 发生室性早搏≥1 个）；或静息心率异常（＞100 bpm）者；
7) 有麻醉意外史者；
8) 卟啉症患者；
9) 不能耐受静脉穿刺采血者；
10) 疤痕体质者；
11) 营养不良或低血容量性休克病史者；
12) 筛选前 2 周内使用过任何处方药、非处方药、保健品、中草药或中成药者；
13) 经常使用镇静、安眠药或其他成瘾性药物者或药物滥用筛查结果阳性者；
14) 在整个研究期及研究结束后 3 个月内有生育、捐精、捐卵计划或不同意在试验期间及研究结束后 3 个月采取有效的非药物避孕措施者（包括男性）；
15) 育龄期女性血妊娠试验检查结果高于正常值范围或处于哺乳期的女性；
16) 既往长期饮用过量茶、咖啡和/或含咖啡因的饮料（1 天 8 杯以上，1 杯=250mL）者，或试验期间不能放弃饮用茶、咖啡等饮料者；
17) 筛选前 3 个月内吸烟超过 5 支/日，或在整个研究期间不能放弃吸烟者；
18) 筛选前 12 个月内有酗酒史者（即男性每周饮酒超过 28 个标准单位，女性每周饮酒超过 21 个标准单位；1 单位相当于啤酒 285mL，或烈酒 25 mL，或葡萄酒 150 mL），或筛选前 6 个月内经常饮酒（每周饮酒超过 14 个标准单位），或酒精呼气检测为阳性者，或整个研究期间不能放弃饮酒者；
19) 筛选前 3 个月内献过血或失血≥400mL 者，或计划在研究期间及研究结束后 3 个月内参与献血者；
20) 筛选前 3 个月内参加过其它药物临床试验，或计划在研究期间参加其它药物临床研究者；

严禁复制

21) 预期参加研究期间需要手术或住院者；

22) 研究者认为其痛觉异常，不适宜参加本试验者；

23) 研究者认为不宜参加本研究的其它情况。

6 试验药物

6.1 药物名称、理化性质、规格和贮藏

6.1.1 长效罗哌卡因注射液

药物名称：长效罗哌卡因注射液

剂型：注射剂

辅料：苯甲醇、苯甲酸苄酯、维生素 E、大豆油

规格：10mL:200mg

贮　藏：2~8℃避光贮藏（不可冷冻）。

有效期：暂定 24 个月

6.1.2 对照药

盐酸罗哌卡因注射液（耐乐品 Naropin）

规　格：100mg/10mL，

生产商：AstraZeneca AB

贮　藏：30℃以下室温贮藏，避免冻结。

有效期：36 个月

6.1.3 药物的生产与贮藏

临床研究用药物和对照药应符合 GCP 要求，在具有符合 GMP 要求的条件下生产，必须具有药品检验报告。

本项研究试验药物必须保存在安全并上锁的地方，未授权人员不能接触。

试验药必须在限定的温度范围内（2~8℃），避光贮藏，不可冷冻；对照药物 30℃以下室温贮藏，避免冻结。必须每日监测试验药物和对照药物的贮藏温度，至少采用有刻度的/经校准的温度计来监测和记录每日温度（最好有连续温度监控）。

贮藏温度发生任何偏差，一经确定应立即向申办者联系人或临床监查员报告。暴露于偏差温度后的试验药物或冻结的试验药物，在获得申办者批准之前不能使用。

严禁复制

6.2 试验药物的包装、标签、编号

标签的准备：本试验药物包装在事先贴好标签的药盒中供受试者使用。标签将同时贴在药品出厂时的最小包装盒上。每只药盒标签上都有药物名称、药物编号、规格、用法、贮藏、有效期及生产单位，并注明“仅供临床研究用”。

药品包装盒标签：

**长效罗哌卡因注射液**<br>（仅用于临床研究，请按临床试验方案使用）<br>1 支/盒<br>受试制剂-T<br>药品编号：T#***<br>临床试验方案编号：<br>LB-RSRI-I-AICOMER<br>【规 格】10ml:200mg<br>【产品批号】CRxxxxxxxx<br>【生产日期】xxxx.xx.xx<br>【有效期】暂定 24 个月	【用法用量】皮下注射。<br>【贮藏】2～8℃避光贮藏。（不可冷冻）<br>**申办方：西安力邦制药有限公司**

注：药品标签实际尺寸正面 40㎜×26㎜ ，背面 40㎜×26㎜　字号小六，行距固定值 9 磅

药品标签：

**长效罗哌卡因注射液**<br>（仅用于临床研究，请按照临床试验方案使用）<br>受试制剂-T　　　　药品编号：T#***<br>临床试验方案编号：LB-RSRI-I-AICOMER<br>【规　　格】10ml:200mg　　【产品批号】XXXXXX<br>【生产日期】xxxx.xx.xx　　【有效期】暂定 24 个月<br>**申办方：西安力邦制药有限公司**

注：药品内标签实际尺寸为 56㎜×27㎜　字号小六号，行距固定值 9 磅

**盐酸罗哌卡因注射液（耐乐品）**<br>（仅用于临床研究，请按照临床试验方案使用）<br>对照制剂-Z　　　　药品编号：Z#***<br>临床试验方案编号：LB-RSRI-I-AICOMER<br>【规　　格】10ml:100mg　　【产品批号】XXXX<br>【生产日期】xxxx-xx　　【有效期至】xxxx-xx<br>**申办方：西安力邦制药有限公司**

注：药品内标签实际尺寸为 56㎜×27㎜　字号小六号，行距固定值 9 磅

药物编号：药物编号形式为T#***或Z#***，其中，T表示试验药，Z表示对照药；#为X或Y，X表示试验1，Y表示试验2；***为3位数字序号，从001开始编写至所需号码。

考虑100%的备用率，试验1需要试验药30支，药物编号为TX001至TX030；试验2需要对照药12支，药物编号为ZY001至ZY012，需要试验药248支，药物编号为TY001至TY248。

6.3 药物运送、分发和清点

应根据西安力邦制药有限公司的标准操作程序将试验药物运抵研究中心。试验药物将按药物标签说明贮藏。

试验药物应由研究者负责分发和清点。试验药物仅用于该临床试验的受试者，其剂量与用法遵照试验方案，且剩余的完整包装试验药物需退回申办者，不得转交给任何非临床试验参加者。

研究者应指定相应人员负责试验药物的贮藏、发放、回收、记录工作。经过适当的间隔，或直到研究结束后，所有未使用的试验药物以及空的容器都必须返还给西安力邦制药有限公司。

必须备有库存记录以便临床监查员检查。提供的试验药物，包括已使用的或空的药盒需经过临床监查员的核对。

7 研究设计

7.1 总体设计

本研究是一项在健康受试者中观察长效罗哌卡因注射液单次皮下给药的安全性、耐受性、药代动力学及药效学的随机、单盲、阳性药物对照、剂量递增研究。

7.2 受试者样本量

根据《化学药物临床药代动力学研究技术指导原则》[31]本研究计划入组健康受试者 50 例，男女比例不限。

其中 6 例健康受试者用于试验所需全血及空白血浆样本的采集（附件 1）。

其中 3 例健康受试者用于考察长效罗哌卡因注射液单点皮下最佳给药剂量试验，确定单点最佳给药剂量。研究初步设定 0.6mL（12mg）、0.8mL（16mg）、1.0mL（20mg）、1.2mL（24mg）、1.5mL（30mg）五个剂量，总计 5.1mL（102mg），3 例健康受试者，每位受试者单次不同给药点给予五种不同剂量药物。单点分别腹部皮下给予不同剂量试验药，考察不同剂量单次给药部位的局部刺激性，确定单点最佳给药剂量。

其余 41 例健康受试者用于考察长效罗哌卡因注射液不同剂量单次皮下给药在健康受试者中的安全性和耐受性、药代动力学和药效学试验。

表 1　健康受试者单次给药试验给药剂量递增方案

队列	剂量（mg）	健康受试者例数（n）	
		试验组<br>长效罗哌卡因注射液	对照组<br>盐酸罗哌卡因注射液
A	150	6	3
B	230	8	--
C	300	8	--
D	350	8	--
E	400	8	--
共计		38	3

如上表，研究初步设定 150 mg、230 mg、300mg、350 mg、400 mg 五个剂量队列。150mg

严禁复制

组随机分为试验组和对照组，分 3 批进行给药，每批受试者中试验组（长效罗哌卡因注射液）和对照组（盐酸罗哌卡因注射液）例数分别为 1+1 例、2+1 例、3+1 例，共计 9 例，即第一批试验组和阳性对照组各 1 例，第二批试验组 2 例，阳性对照组 1 例，第三批试验组 3 例，阳性对照组 1 例；230mg 至 400mg 组每队列仅设试验组（长效罗哌卡因注射液），每组受试者各 8 例，不设对照组，每组分两批进行试验，第一批 3 例受试者，第二批 5 例受试者。

7.3 起始剂量的确定

已上市盐酸罗哌卡因注射液说明书中[14]，临床推荐外科手术麻醉末梢神经区域阻滞和浸润麻醉给药剂量为 7.5~225mg；急性疼痛控制末梢神经区域阻滞和浸润麻醉临床常用推荐剂量为 2~200mg。

长效罗哌卡因注射液的临床前安全药理学研究、单次给药毒性研究、过敏性、溶血性和局部刺激性研究、遗传毒性研究以及生殖毒性研究结果如下：

长效罗哌卡因注射液在 30、60、120mg/kg 剂量下单次皮下注射给药对 SD 大鼠中枢神经系统功能无明显影响[13]。

长效罗哌卡因注射液以 75，150 和 300mg/kg 的剂量单次皮下注射给予 SD 大鼠，试验药物动物最大耐受剂量为 150mg/kg[15]。

长效罗哌卡因注射液单次皮下注射给予 Beagle 犬，动物耐受剂量高达 60mg/kg[17]，此剂量下未观察到系统毒性。本试验研究结果认为未观察到明显的毒性反应剂量(NOAEL)≥60mg/kg。

长效罗哌卡因注射液在 30、60、120mg/kg 剂量下单次皮下注射给药，对 SD 大鼠中枢神经系统功能无明显影响[13]。皮下注射 15、30、60 mg/kg 剂量下给药，对清醒 Beagle 犬心血管、呼吸系统功能及体温无明显影响[12]。

单次给药毒性研究结果显示，SD 大鼠单次皮下注射长效罗哌卡因注射液的最大耐受剂量大于 150 mg/kg[15]，Beagle 犬单次皮下注射长效罗哌卡因注射液，无明显不良反应的剂量均为 60 mg/kg[17]，故长效罗哌卡因注射液单次给药显示出了良好的耐受性。

本试验以动物毒理学试验的未见明显毒性反应剂量（No Observed Adverse Effect Level，NOAEL，mg/kg）为基础，使用人体等效剂量（Human Equivalent Dose，HED，mg/kg）推导临床试验的最大推荐起始剂量[25-27]（Maximum Recommended Starting Dose，MRSD，mg/kg）。

本品临床前动物毒性试验，比格犬一般药理和急性毒试验中，未见明显毒性反应剂量

（NOAEL）为 60 mg/kg，以下计算中，成人体重以 60kg 计，安全系数（safe factor，SF）取 10。

1）根据体表面积换算 HED 并计算 MRSD：

结合以下公式及表 2 中的 Km 数据计算长效罗哌卡因注射液的初始剂量：

HED= NOAEL×动物 Km/人体 Km。

HED =60mg/kg×21.47/36.88=34.93mg/kg

MRSD =HED（mg）×SF。

MRSD=34.93mg/kg×1/10=3.49mg/kg

成人体重以 60kg 计，3.49mg/kg×60kg=209.40mg，即起始剂量为 209.40mg。

表 2.根据体重计算 Km 实例值

种属	参考体重（kg）	体表面积（m²）	Km
人	60	1.6268	36.88
儿童	20	0.80	26.47
小鼠	0.020	0.006086	3.29
仓鼠	0.080	0.01602	4.99
大鼠	0.150	0.02484	6.04
大鼠	0.300	0.04029	7.45
白鼬	0.300	0.04029	7.45
豚鼠	0.400	0.04925	8.12
兔	1.8	0.14073	12.79
犬	10	0.46580	21.47
灵长类			
猴	3	0.20102	14.92
微型猪	20	0.7557	26.47
小型猪	40	1.2259	32.63

2）使用 mg/kg 换算 HED 并计算 MRSD：

HED（mg/kg）= NOAEL（mg/kg）=60mg/kg

MRSD=60mg/kg×1/10=6mg/kg

成人体重以 60kg 计，6mg/kg×60kg=360mg，即起始剂量为 360mg

3）根据相关文献[27]计算 HED 并计算 MRSD：

HED（mg/kg）= NOAEL×R_{ab}=60mg/kg×0.541=32.46mg/kg

MRSD=32.46mg/kg×1/10=3.25mg/kg

成人体重以 60kg 计，3.25mg/kg×60kg=195.00mg，即起始剂量为 195.00mg。

本品临床前镇痛试验结果显示，大鼠 3.6mg/只剂量组药效作用不明显，与溶剂组和模型组比较痛阈值略高但无显著性差异（$p>0.05$），HED（按大鼠 3.6mg/0.25kg，成人 60kg 标准体重计算） = Da×Rab×60kg=（3.6mg/0.25kg）×0.162×60kg=140.00mg，即大鼠 3.6mg/只的人体等效剂量为 140.00mg/人，该剂量下镇痛药效作用不明显。

已上市盐酸罗哌卡因注射液（耐乐品）最高单次给药剂量为 250mg，曾被使用过，而且能够很好的耐受。

耐乐品说明书中，急性疼痛控制（区域阻滞）的推荐剂量为 2-200mg。

综上，为了充分保障长效罗哌卡因注射液首次用于人体临床研究的安全性，同时兼顾本品制剂特点、皮下注射的给药方式和产生有效数据的各方面因素，我们将 I 期耐受性临床试验，健康受试者的起始注射剂量设定为 150mg/次。

7.4 以动物最低有效剂量换算人体起效剂量

参照黄继汉等人《药理试验中动物间和动物与人体间的等效剂量换算》[27]中人和动物剂量折算表计算人体起效剂量。

根据长效罗哌卡因注射液临床前 SD 大鼠药效学试验结果[3-5]，本品给药剂量大于等于 0.4ml（8mg）时，在给药后各时间点，对大鼠热刺激痛阈最高且无显著差异，估算拟用于人体的有效剂量约为：换算至人的剂量 5.2 mg/kg（换算系数 R_{ab}=0.162），以成人 60kg 体重计算，临床有效剂量约为 300mg/次。

临床前药代动力学试验结果显示，耐乐品对照组与长效罗哌卡因试验组同剂量（10mg/kg）单次皮下注射药代动力学参数 Cmax 的比值为 4.76（2.76μg/mL/ 0.58μg/mL）。

临床前犬急性毒性试验结果显示，犬单次皮下注射长效罗哌卡因注射液无明显不良反应的剂量为 60 mg/kg，换算成人的剂量 32 mg/kg。以成人 60kg 体重计算，HED 为 1920mg/人。

已上市盐酸罗哌卡因注射液（耐乐品）说明书中，手术麻醉中累计剂量达到 800mg 时，对成人来说都可很好耐受。

7.5 最大剂量的确定

根据未观测到的不良反应剂量（NOAEL）计算人体最大剂量

临床前大鼠急性毒性结果显示，SD 大鼠单次皮下注射长效罗哌卡因注射液的最大耐受剂量为 150 mg/kg，换算至人体的剂量 24.3 mg/kg（换算系数[27] R_{ab}=0.162），以成人 60kg 体重计算，最大剂量为 1458mg/次。

严禁复制

临床前犬急性毒性试验结果显示，犬单次皮下注射长效罗哌卡因注射液无明显不良反应的剂量为 60 mg/kg，换算成人的剂量 32 mg/kg（换算系数[27] R_{ab}=0.541）。以成人 60kg 体重计算，最大剂量为 1920mg/次，取 1/5 至 1/2 做最大耐受量估算，换算为人体最大剂量范围为 360mg/次至 900mg/次。

已上市盐酸罗哌卡因注射液（耐乐品）说明书中，盐酸罗哌卡因注射液应用于硬膜外阻滞，单次最高使用剂量 250mg 呈现良好耐受；单次手术麻醉中累计剂量达到 800mg 时，对成人来说都可很好耐受。

临床前药效学试验结果显示，大鼠给予盐酸罗哌卡因皮下注射 12mg/只时，有 50%动物死亡，50%动物出现全身麻醉反应；但试药 12mg/只组均未出现上述现象，且在相同给药容量时，试药可明显延长镇痛时间。提示：用药部位影响药效剂量范围，且大鼠 12mg/只的人体等效剂量 HED= 466.80mg 为重要关注剂量。

从以上的最大耐受剂量数据，结合最低有效剂量结果，可以看出，长效罗哌卡因注射液具有很宽的安全剂量范围。考虑到本制剂为皮下注射给药，规格为 10mL:200 mg，而皮下给药每次注射量一般仅为 1 mL-2mL。

超过一定剂量范围时，随着剂量的进一步增加，疗效可能并不呈线性关系，因此，确定将 400 mg，相当于 20mL 试验制剂作为健康受试者的最大给药剂量，用于观察长效罗哌卡因注射液的安全性和耐受性。

7.6 剂量的递增

参照针对小分子药物剂量递增设计方法-单剂量推升法，从最低起始剂量（150 mg）到最高剂量（400 mg），考虑动物药效学剂量约为 300mg 左右，将剂量按照等量递增方法，将健康受试者分为 5 个队列：队列 A（150mg）、队列 B（230mg）、队列 C（300mg）、队列 D（350mg）、队列 E（400mg）。队列 A 随机分为试验药给药组和对照药给药组，分 3 批进行试验，每批试验药给药组和对照药给药组受试者例数分别为 1+1 例、2+1 例、3+1 例。队列 B 至队列 E 仅有试验药给药组，分两批进行试验，第一批 3 例受试者，第二批 5 例受试者，见图 1。

图 1：健康受试者单次给药试验剂量递增方案

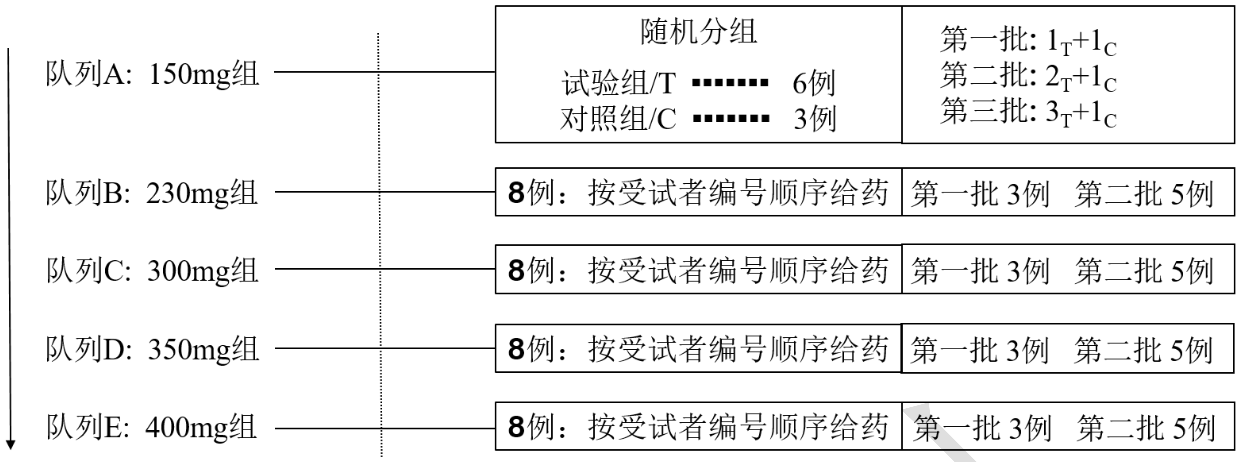

研究从最小剂量开始给药，每位受试者只接受一个剂量给药。同一时间内仅进行 1 个剂量队列的研究，在给药后第 7 天和第 21 天进行安全性评估。

各剂量组按剂量由低到高递增进行，在前一个剂量组给药结束，研究者需全面审查前一剂量组受试者的全部安全性数据，包括临床观察、体格检查、生命体征、心电图、实验室检查报告等，如给药后 7 天（±2 天）未显示不能接受的不良反应时，根据评估结果，决定是否进行下一队列的试验。若试验队列中有≥1/2 受试者出现≥II级不良事件或≥1/3 的受试者出现≥III级不良事件，由研究者与申办方讨论，是否终止剂量递增[28]。

如在递增至设定的最大剂量队列（400mg）时，无受试者、临床试验研究者、或临床不能接受的任何不良反应发生，则可结束耐受性试验。如尚未达到设定的最大剂量时，已出现不能接受的不良反应时，则应终止耐受性试验，此前一组剂量为最大耐受剂量。

7.7 给药方案及试验周期

本品单次经皮下注射给药，注射部位为腹部皮下。本品不可静脉或肌肉注射。

给药当天按受试者所属的剂量队列，经腹部单次多点皮下注射相应剂量的试验药物。

7.7.1 长效罗哌卡因注射液单点皮下最佳给药剂量探索试验方法：

3 例健康受试者，每位受试者腹部六分区从剑突部位开始顺时针方向依次标注为 I、II、III、IV、V、VI。随机化确定起始给药区域并顺时针依次标注为 1、2、3、4、5，皮下单次注射给予试验药 0.6mL（12mg）、0.8mL（16mg）、1.0mL（20mg）、1.2mL（24mg）、1.5mL（30mg）五个剂量，总计 5.1mL（102mg）；第 6 个点作为空白对照点。考察不同剂量腹部皮下注射试验药后，给药部位的局部皮肤刺激性反应评分，镇痛范围；同时观察受试者不良反应和不同剂量注射区域的局部皮肤刺激性反应，包括临床症状与体征、实验室检查、不良事件（包括

全身不良反应及注射部位外观、注射部位疼痛或压痛、注射部位红斑或发红、注射部位硬结或肿胀、注射部位瘙痒等）。

确定单点最佳给药剂量（L）和该给药剂量的镇痛范围的平均直径（D）。

- 每个注射点仅给予一个剂量（剂量由低到高依次进行）；
- 每个注射点仅给药一次；
- 腹部注射点的选取方法：以通过脐的水平线和左、右髂前上棘至腹中线连线的中点为两条垂直线将腹部分为六个区（图 2），从剑突部位开始按顺时针方向分别标注为 I、II、III、IV、V、VI，每个区域内任意选取一个点为注射点，任意两点间的距离不低于 5cm；

图 2：腹部六分区示意图

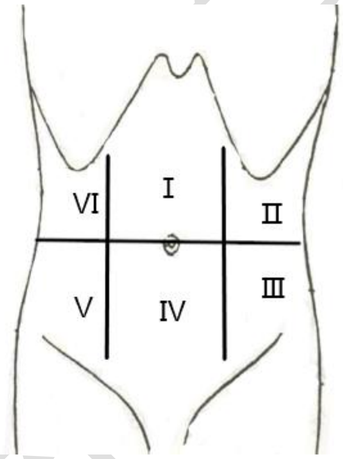

- 观察注射部位局部皮肤刺激性反应：给药后观察注射部位局部皮肤刺激性反应，待评估后，实施下一次给药或终止下一剂量给药（两次注射间隔需≥20min）：

  （1）皮肤刺激性反应≤2 分时，可继续下一剂量注射；

  （2）皮肤刺激性反应≥3 分时，每隔 10min 观察 1 次，直至皮肤刺激性反应≤2 分，记录时间，继续下一剂量注射；

  （3）皮肤刺激性反应≥3 分，且连续观察 2h 未缓解，终止下一剂量注射，观察或随访至反应消失；

- 注射部位局部皮肤刺激性反应评估：试验期间注射部位局部皮肤刺激性反应按皮肤刺激性评分系统[30]进行评估，于每次给药后 20min，40min，1h，2h，6h，24h 进行评分；受试者出组后参照《常见不良事件评价标准（CTCAE）》（V5.0，2017 年 11 月 27 日公布）判定并记录局部皮肤刺激性反应，包括注射部位疼痛或压痛、注射部

位红斑或发红、注射部位硬结或肿胀、注射部位瘙痒等；

皮肤刺激性反应评分表

分值	皮肤反应
0分	未见刺激性
1分	轻微红斑，刚刚能观察到
2分	明显的红斑，肉眼易见，轻微水肿或轻微丘疹反应
3分	红斑和丘疹
4分	明显的水肿
5分	红斑、水肿和丘疹
6分	水泡
7分	强烈反应，分布范围超出测试部位

- 受试者单点给药后需住院观察至少 12 小时。

7.7.2 长效罗哌卡因注射液不同剂量单次皮下给药在健康受试者中的安全性和耐受性及药代动力学试验方法：

每位受试者，依据腹腔镜手术的腹部区域，按腹部四分区法（即通过脐划水平线和垂直线，两线相交将腹部分为四个区，见图 3）标注四个区域，从剑突位置顺时针依次标注为I、II、III、IV，随机确定起始给药区域，并顺时针依次标注给药区域为 1、2、3，最后一个区域为空白对照区域。

图 3：腹部四分区示意图

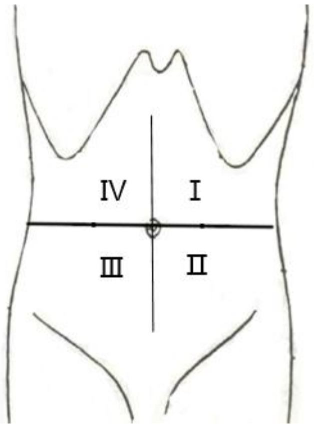

- 每个区域内任选一点为圆心（任意两圆心间距离不低于 80mm），标记直径为 40mm 的圆。

严禁复制

- 按 1.2mL/针 计算每个队列的注射点数量，即每队列注射点数量=该队列剂量/1.2，每个圆周上注射点数量=每队列注射点数量/3，取整数确定第 1、2 个圆周上的注射点数量，剩余剂量分配在第 3 个圆上；
- 每个圆周上的注射点需在该圆周上平均分布；
- 标记注射点为 1a，1b，1c……；2a，2b，2c……；3a，3b，3c……（每个圆周上的注射点序号需从该圆的正上方，即头部方向顺时针进行排序）；
- 标记痛觉测试区域：在圆 1 或 2 上标记圆心(1/2)、距离圆心 1cm（A）、3cm(B)、4cm(C)的点为痛觉测试点；以每个针刺测量点为圆心，标记直径约为 1cm 的圆作为针刺痛觉测量区域；痛觉测试区域依次记录为圆心 1，圆心 2；1aA，1bA，1cA……；2aB，2bB，2cB……；3aC，3bC，3cC……；
- 仅在圆 1 和圆 2 上进行针刺疼痛强度测定；
- 皮下注射给予试验药品（受试制剂或对照制剂），皮下注射位置需对映于圆周上；
- 观察注射部位局部皮肤刺激性反应：注射部位局部皮肤刺激性反应评估按皮肤刺激性评分系统[30]进行评估，于给药前和给药结束后 20min，40min，1h，2h，6h，24h、48h、72h 进行评分，详见 7.7.1 皮肤刺激性反应评分表；受试者出组后参照《常见不良事件评价标准（CTCAE）》（V5.0，2017 年 11 月 27 日公布）判定并记录局部皮肤刺激性反应，包括注射部位疼痛或压痛、注射部位红斑或发红、注射部位硬结或肿胀、注射部位瘙痒等；
- 测量镇痛维持时间、起效时间和针刺疼痛强度，详见 7.14.1；
- 测量并记录同一圆周上相邻两个注射给药点之间的直线距离；
- 给药研究者和痛觉测量研究者不可为同一人；
- 受试者在给药后需住院观察 72 小时，方能出院。

7.7.3 皮下注射方法及注意事项：

7.7.3.1 注射方法

① **皮肤消毒** 腹部分区前进行腹部消毒，注射给药。

② **进针** 捏起皮肤成褶皱，在褶皱最高点垂直、迅速进针，固定针头。

③ **回吸** 防止血管内注射。

④ **注射** 缓慢匀速推注药液，每个剂量于 25min 内注射完毕，拔针，轻轻按压。

⑤ **临床观察** 观察渗出情况，并记录。

7.7.3.2 注意事项：

① 受试者需平卧给药；

② 注射后禁止热敷、按摩；

③ 注射后不可用力按压。

7.8 研究步骤

7.8.1 筛选

对参与试验者，研究者应详细向其阐明试验的获益和风险。

在进行筛选评估前，受试者应知情并签署知情同意书。在签署知情同意书后，每位受试者将接受筛选检查，经各项检查符合入组标准后，方可入组。

7.8.2 入组

经筛查符合入组标准的受试者入组本项研究。

7.8.3 随机化

7.8.3.1 长效罗哌卡因注射液单点皮下最佳给药剂量试验（试验1）。

在受试者腹部按7.7.1腹部注射点选取方法选取6个区域，由剑突部位开始按照顺时针标注为I、II、III、IV、V、VI，随机确定第一个给药区域，在第一个给药区域标注第一个注射点，顺时针依次标注并给药（剂量由小到大），随机后的最后一个区域为空白对照区域。

7.8.3.2 长效罗哌卡因注射液不同剂量单次皮下给药在健康受试者中的安全性和耐受性、药代动力学试验（试验2）

每位受试者，按腹部四分区法（即通过脐划水平线和垂直线，两线相交将腹部分为四个区）标注四个区域，从剑突位置顺时针依次标注为I、II、III、IV，随机确定起始给药区域，并顺时针依次标注为圆1、2、3，最后一个区域圆4为空白对照区域。

受试者在签署知情同意书后将被分配一个S+3位数的受试者筛选号，从001开始依次递增；每个剂量队列合格的受试者在入组时将被分配一个4位数的受试者编号，各剂量队列的受试者编号分配信息见下表：

表3 受试者编号表

队列	受试者编号	剂量水平（mg）
A	1001～1009	150
B	2001～2008	230
C	3001～3008	300
D	4001～4008	350

严禁复制

E	5001～5008	400

各剂量组的受试者编号将根据受试者的入组先后顺序由小往大进行分配，受试者编号不能被遗漏。A队列150mg组受试者先随机分为试验组和对照组，再分3批次进行试验，第1批试验组和阳性对照组各1例；第2批试验组2例，阳性对照药组1例；第3批试验组3例，阳性对照药组1例。随机化统计师采用SAS软件分批次产生A队列1001-1009号受试者的随机分配表，按照随机分配表给予试验组和对照组药物。

注意：（1）队列A随机分组需遵循盲法设计，受试者不可知道自己的分组情况；（2）给药研究者和痛觉测量研究者不可为同一人。

7.8.4 给药

受试者入住临床试验病房后，于次日（即给药日）早晨接受一次生命体征检查（包括测量腋下体温、脉搏、卧位血压等）后，再实施皮下注射给药。

7.8.4.1 长效罗哌卡因注射液单点皮下最佳给药剂量试验（试验1）

给药方法：按受试者腹部依次标注的点1，2，3，4，5顺序分别皮下注射试验药0.6mL（12mg）、0.8mL（16mg）、1.0mL（20mg）、1.2mL（24mg）、1.5mL（30mg）。

7.8.4.2长效罗哌卡因注射液不同剂量单次皮下给药在健康受试者中的安全性和耐受性、药代动力学试验（试验2）

同一队列每位受试者，依据腹腔镜手术的腹部区域，按腹部四分区法，标注四个区域，随机确定起始给药区域，按7.7.2方法标记注射点，按注射点标记序号，依次进行皮下注射操作。

按照每个队列设计的给药剂量，给药点在直径40mm的圆1，2，3的圆周上平均分布，每次给药量直至达到每队列设计的总给药剂量。

如出现每次给药点数量在三个圆周不能平均分布的情况，第三个圆周减少或增加一个给药点，但第三个圆给药点依旧平均分布（即给药剂量平均分布于圆周上）。

7.8.5 饮食

试验期间受试者应进清淡平衡膳食，同时避免剧烈运动，并禁止饮用（或食用）含茶碱、咖啡因、可可碱和醇类等。受试者入住 I 期病房期间，除统一提供的标准餐外，不能再额外进餐。

受试者入住I期临床试验病房后将进食统一清淡平衡膳食，进餐时间遵循试验病房常规时间，每餐剩余饮食量应进行记录。

严禁复制

7.9 研究流程

为确保长效罗哌卡因注射液首次在人体中应用的安全性，现制定如下研究流程。药代动力学研究见本研究方案 7.13 节。

7.9.1 筛选期（-14 天至-2 天）

- 知情同意书；
- 人口学资料：包括出生日期、性别、民族等；
- 既往史和现病史：须获取受试者的既往病史及现病史情况，包括：消化系统、中枢神经系统、心血管系统、肾脏、呼吸系统疾病史；
- 个人史和家族史：个人史应包含婚姻史、吸烟史、饮酒史、药物使用史、药物滥用史及过敏史，家族史；
- 体格检查：包括头部、皮肤、粘膜、浅表淋巴结、颈部、胸部、腹部、脊柱/四肢等检查；
- 生命体征：包括腋下体温、卧位血压、脉搏、呼吸、血氧饱和度；
- 潜在困难气道评估：面罩通气困难的独立危险因素评估，喉镜显露和插管困难的特征因素评估及相关体格检查评估，当出现Mallampati分级III或IV级、下颌前伸能力受限、甲颏距离过短(<6 cm)等表现可评判为潜在困难气道；
- 实验室检查：

a. 血常规：应包括红细胞计数（RBC）、白细胞计数（WBC）、血红蛋白（HGB）、红细胞压积（HCT）、血小板计数（PLT）、嗜酸性细胞计数（EOS）、嗜碱性细胞计数（BASO）、中性粒细胞计数（NEUT）淋巴细胞计数(LYMPH)、单核细胞计数（MONO）；

b. 血生化（电解质、肝肾功能）：应包括钾（K^+）、钠（Na^+）、氯（Cl^-）、钙（Ca^{2+}）、镁（Mg^{2+}）、磷（P）、血糖（Glu）、肌酐（CREA）、尿素（UREA）、谷氨酰胺转肽酶（GGT）、尿酸（UA）、乳酸脱氢酶（LDH）、总胆红素（TBIL）、天门冬氨酸氨基转移酶（AST）、丙氨酸氨基转移酶（ALT）、碱性磷酸酶（ALP）、血淀粉酶（AMY）、甘油三酯（TG）、总胆固醇（CHOL）、总蛋白（TP）、白蛋白（ALB）；

c. 血清学检查：包括HBsAg、HCV抗体、HIV抗体和梅毒特异性抗体检测；

d. 血妊娠试验：如结果高于正常值范围，受试者将没有资格参加研究；

e. 尿常规：应包括葡萄糖（GLU）、酮体（KET）、蛋白质（PRO）、尿潜血（BLD）

严禁复制

、白细胞（WBC）、胆红素（BIL）、亚硝酸盐（NIT）、酸碱度（pH）、尿胆原（UBG）、比重（SG）；

- f. 凝血功能：凝血酶原时间（PT）、活化的部分凝血活酶时间（APTT）、凝血酶时间（TT）、纤维蛋白原含量（FIB）；
- 12导联心电图；
- 痛觉测试：测量VAS=4时，垂直施加的最大机械压力值；
- 不良事件（包括SAE）：如果发生任何自觉症状、新的或恶化的症状，受试者均应被马上给予适当的医疗帮助。相关不良事件均参考本方案中的“9.1可能出现的不良事件及抢救措施”一节来处理，如符合定义，还需按不良事件或SAE报告。记录试验期间不良事件的最严重级别。所有给药后发现的妊娠及其结局（自然流产、选择性终止妊娠、异位妊娠、正常分娩或先天畸形等）均应书面报告。男性受试者给药后其配偶发生妊娠需要报告。妊娠本身不作为不良事件报告。若妊娠过程/结局符合SAE的标准，则研究者应按SAE报告程序报告。即使受试者退出研究，也应随访和记录妊娠结局。
- 根据入/排标准，判断是否可入组；

7.9.2入住（-1天）

受试者于给药前一天下午入住I期病房。从入住开始提供统一清淡平衡餐。

- 生命体征：包括腋下体温、卧位血压、脉搏、呼吸、血氧饱和度；
- 体格检查：包括头部、皮肤、粘膜、浅表淋巴结、颈部、胸部、腹部、脊柱/四肢 等检查；
- 血妊娠试验：如结果为阳性，受试者将没有资格参加研究；
- 酒精呼气检查及药物滥用筛查：药物滥用筛查包含吗啡、甲基安非他明、氯胺酮、二亚甲基双氧安非他明、四氢大麻酚酸；
- 12导联心电图；
- 筛选至入住问卷调查：应向受试者询问的事项包括：是否在研究用药前2天饮酒或饮用/食用西柚汁；在研究药物给药前2天饮用（或食用）含有任何咖啡因的食物；是否使用过任何药物或保健品；是否吸食烟草；是否输血、献血等入/排标准中关注的信息；

严禁复制

- 不良事件：同7.9.12一节中的“不良事件”；
- 根据入/排标准，判断是否可入组；
- 入组受试者入住 I 期临床试验病房。
- 标记注射点（仅试验2）：每位受试者，腹部按7.7.2描述方法进行注射点标记；
- 针刺疼痛强度基线值测量（仅试验2）。

7.9.3 试验期给药和观察

受试者继续入住 I 期病房，试验 1 受试者给药后第 2 天完成出组检查后离院；试验 2 受试者于给药后第 4 天完成出组检查后离院，并接受相关观察。

7.9.3.1 考察长效罗哌卡因注射液单点皮下最佳给药剂量试验（试验 1）

7.9.3.1.1 单点皮下最佳给药剂量试验（Day 1）

- 生命体征：于第一次给药前 1h 内，第一次给药后 1h、2h±15min、4h、8h、12 h±30 min 测量卧位血压、脉搏、呼吸、血氧饱和度各一次，共 6 次；于第一次给药前 1h 内，第一次给药后 4h、12h±30 min 测量腋下体温各一次，共 3 次；
- 标记注射点：每位受试者腹部按 7.7.1 描述方法选取 6 个注射区域并标记；
- 上午 8:00±1h 皮下注射试验药物；
- 注射部位局部皮肤刺激性反应：于注射前 30min 内，注射后 20min、40min±5min、1h、2h、6h±10min，按 7.7.1 皮肤刺激性反应评分表进行评分；
- 镇痛范围测定：于单点给药后 15min、30min、45min±3min、1h、1.5h、2h、3h、4h、5h、6h、7h、8h、9h、10h、11h、12h ±5min，沿两个方向确定疼痛点（当距离给药点为 1cm 的测定点（n）的 VAS 值≥4 时，测定至（n+1）时间点时可终止测量），测量并记录疼痛点和给药点之间的距离（r_1，r_2），镇痛范围直径 D =（r_1+r_2）；
- 记录试验药物的发放/使用/回收；
- 心电监护：必要时；
- 合并用药：同 7.12 一节中的“合并用药”；
- 不良事件（包括 SAE）：如果发生任何自觉症状、新的或恶化的症状，受试者均应被马上给予适当的医疗帮助。相关不良事件均参考本方案中的“可能出现的不良事件及抢救措施”一节来处理，如符合定义，还需按不良事件或 SAE 报告。记录试验期间不良事件的最严重级别。所有给药后发现的妊娠及其结局（自然流产、选择性终止妊娠、异位妊娠、正常分娩或

严禁复制

先天畸形等）均应书面报告。男性受试者给药后其配偶发生妊娠需要报告。妊娠本身不作为不良事件报告。若妊娠过程/结局符合 SAE 的标准，则研究者应按 SAE 报告程序报告。即使受试者退出研究，也应随访和记录妊娠结局。

7.9.3.1.2 单点皮下最佳给药剂量试验(Day 2)

- 体格检查：各系统体格检查（询问受试者用药后的主观症状并作体检，各个系统出现的临床症状和体征）；
- 生命体征：包括腋下体温、卧位血压、脉搏、呼吸、血氧饱和度；
- 实验室检查：
  - a. 血常规：同7.9.1一节中的“血常规”；
  - b. 血生化：同7.9.1一节中的“血生化（电解质、肝肾功能）”；
  - c. 血妊娠试验：同7.9.1一节中的“血妊娠试验”；
  - d. 尿常规：同7.9.1一节中的“尿常规”；
- 心电监护：必要时；
- 12导联心电图；
- 合并用药：同7.12一节中的“合并用药”；
- 不良事件（包括SAE）：同9.1一节中的“不良事件（包括SAE）”；
- 注射部位局部皮肤刺激性反应：于给药后24h和离院前按7.7.1皮肤刺激性反应评分表对注射部位局部皮肤刺激性反应进行评分；
- 若镇痛反应未消失，需继续在 I 期试验病房观察至镇痛反应消失，方可离院。

7.9.3.2 长效罗哌卡因注射液不同剂量单次皮下给药在健康受试者中的安全性和耐受性、药代动力学试验（试验 2）

7.9.3.2.1 安全和耐受性试验（Day 1）

- 生命体征：卧位血压、脉搏、呼吸、血氧饱和度，检测时间点为给药前1h内、给药后1h、2h±15min、4h、8h、12h±30 min各一次，共6次；腋下体温检测时间点为给药前1h内，给药后4h、12h±30 min各一次，共3次；
- 上午9:00±1h 皮下注射试验药物；

严禁复制

- 药代动力学血液采集：静脉取全血约4mL，采血时间点为给药前15min内、给药结束后0.5h、1h、1.5h、2h，4h、6h、8h、10h、12h、14h各一次，共11次；
- 针刺疼痛强度测量：给药前和给药结束后15min、1h、4h、8h、10h、12h、14h [29]测量针刺疼痛强度；
- 注射部位局部皮肤刺激性反应：于给药前，给药结束后20min、40min、1h、2h、6h观察并记录注射部位刺激性评分；
- 测量并记录同一圆周上相邻两个注射给药点之间的直线距离；
- 记录试验药物的使用/回收/销毁；
- 心电监护：必要时；
- 合并用药：同7.12一节中的“合并用药”；
- 不良事件（包括SAE）：同9.1一节中的“不良事件（包括SAE）”。

7.9.3.2.2 安全和耐受性试验（Day 2）

- 生命体征：包括腋下体温、卧位血压、脉搏、呼吸、血氧饱和度；
- 药代动力学血液采集：静脉取全血约4mL，采血时间点为给药结束后21h、24h、27h、30h、33h、36h各一次，共6次；
- 针刺疼痛强度测量：给药结束后18h、21h、24h、27h、30h、33h、36h测量针刺疼痛强度。
- 注射部位局部皮肤刺激性反应：于给药结束后24h ±5min，对注射部位局部皮肤刺激性反应进行评分；
- 心电监护：必要时；
- 合并用药：同 7.12 一节中的“合并用药”；
- 不良事件（包括SAE）：同9.1一节中的“不良事件（包括SAE）”。

7.9.3.2.3 安全和耐受性试验（Day 3）

- 生命体征：包括腋下体温、卧位血压、脉搏、呼吸、血氧饱和度；
- 药代动力学血液采集：静脉取全血约4mL，采血时间点为给药结束后48h ±5min；
- 针刺疼痛强度测量：给药结束后48h测量针刺疼痛强度；

严禁复制

- 注射部位局部皮肤刺激性反应：48h±5min对注射部位局部皮肤刺激性反应进行评分；
- 心电监护：必要时；
- 合并用药：同 7.12 一节中的“合并用药”；
- 不良事件（包括SAE）：同9.1一节中的“不良事件（包括SAE）”。

7.9.3.2.4 安全和耐受性试验（Day 4）：

- 体格检查：各系统体格检查（询问受试者用药后的主观症状并作体检，各个系统出现的临床症状和体征）；
- 生命体征：包括腋下体温、卧位血压、脉搏、呼吸、血氧饱和度；
- 实验室检查：

 a. 血常规；b. 血生化；c. 尿常规；d.血妊娠试验；

- 12导联心电图；
- 药代动力学血液采集：静脉取全血约4mL，采血时间点为给药结束后72h ±5min；
- 针刺疼痛强度测量：给药结束后72h针刺疼痛强度；
- 注射部位局部皮肤刺激性反应：于给药结束后 72h ±5min，按皮肤刺激性反应评分表对注射部位局部皮肤刺激性反应进行评分；
- 心电监护：必要时；
- 合并用药：同 7.12 一节中的“合并用药”；
- 不良事件（包括SAE）：同9.1一节中的“不良事件（包括SAE）”；
- 出组：完成各项检查，离开Ⅰ期试验病房。

7.9.4 随访

如临床症状、体格检查或实验室检查某项指标在试验期结束时（出院时）仍出现有临床意义的异常，则需继续随访直至该指标恢复至基线水平或稳定或失访。

给药后第 7 ±2、21 ±3 天进行 2 次随访，记录出院后至第 21 天新发生的注射部位局部皮肤刺激性反应、不良事件（包括 SAE）及合并用药等情况。

7.10 退出试验

所有签署知情同意书的受试者，均有权随时退出临床试验。但只要使用过药物者，均需参与安全性及耐受性评价。

严禁复制

7.10.1 退出标准

1) 受试者或其法定代理人要求退出研究；
2) 研究者从受试者的最大利益出发判定应终止该例受试者的研究；
3) 研究期间发现受试者怀孕；
4) 研究者认为受试者依从性差，严重违背研究方案；
5) 受试者死亡。

7.10.2 退出程序及处理

对于退出研究的受试者，研究者应当询问其退出原因和是否发生任何不良事件。退出研究的原因和日期应当要记录在原始病历和病例报告表或电子病例报告表上（eCRF）。如果可能，研究者应对退出研究的受试者进行安全性评估（试验 1 评估项目同 d2，试验 2 评估项目同 d4）：

- 体格检查：各系统体格检查（询问受试者用药后的主观症状并作体检，各个系统出现的临床症状和体征）；
- 生命体征：包括腋下体温、卧位血压、呼吸、脉搏和血氧饱和度；
- 实验室检查：
  a. 血常规：同 7.9.1 一节中的“血常规”；
  b. 血生化：同 7.9.1 一节中的“血生化”；
  c. 尿常规：同 7.9.1 一节中的“尿常规”；
- 12 导联心电图；
- 合并用药：同 7.12 一节中的“合并用药”；
- 不良事件（包括 SAE）：同 9.1 一节中的“不良事件（包括 SAE）”；
- 注射部位局部皮肤刺激性反应：按 7.7.1 皮肤刺激性反应评分表对注射部位局部皮肤刺激性反应进行评分。

退出试验时，如有新发的或加重的实验室检查异常值，受试者需接受进一步检查。结果应记录在病例报告表或 eCRF 的相应部分。

终止研究时所有存在的不良事件和 SAE 应当随访至到其缓解基线水平或稳定或失访，除非根据研究者的意见，该状况因受试者本身的疾病而不可能缓解。对这些病例，研究者需在病历上记录。

严禁复制

7.11 终止试验标准

1） 若在试验进行中出现任何一例与研究药物相关的严重不良事件（SAE），需暂停试验，研究者与申办者一起讨论分析原因，并判断对后续试验的影响后，再决定是否继续试验；

2） 参照《常见不良事件评价标准（CTCAE）》（V5.0，2017 年 11 月 27 日公布），若试验队列中有≥1/2 受试者出现≥Ⅱ级不良事件或≥1/3 的受试者出现≥III级不良事件（不良事件需经研究者判断可能与试验药物相关），则由研究者与申办方讨论，是否终止剂量递增；

3） 若在最大剂量队列（400mg）中仍未观察到明显不良反应，则不再继续进行剂量递增。

4） 申办方要求全面终止试验；

5） 国家药品监督管理局或伦理委员会因某种原因勒令终止试验。

7.12 合并用药

7.12.1 试验期间不允许使用的药物

从筛选期到最后的临床观察和实验室检查完成期间，禁止擅自使用任何药品。包括任何处方药、非处方药、中草药、特殊医学用途配方食品、保健品。

7.12.2 试验期间允许使用的药物

紧急情况下，出于对受试者安全和健康的考虑，如果经研究者判断确需合并使用其他药物，可在研究者的指导下使用，并及时告知申办方药物的使用情况。所有的合并用药及治疗都必须记录在病例报告表或eCRF的相应位置上。包括药品名称（化学名和商品名）、剂量、给药方式、目的和用药起止日期。

7.13 药代动力学研究

7.13.1 药物剂量

本试验共设有五个队列，五个剂量组：150 mg、230mg、300mg、350mg、400 mg。

7.13.2 血样采集时间

根据人体药代动力学研究指导原则，结合长效罗哌卡因注射液的特点，初步拟定的健康受试者血样采集时间如表 4 所示。可根据健康受试者低剂量队列实际获得的药时曲线，调整后续队列的采血点。

表 4 药代动力学研究的血样采集时间

采血点序号	采血点	时间窗
1	给药前 15 min 内	/
2	给药结束后 0.5h	±2 min
3	1 h	±2 min
4	1.5 h	±2 min

严禁复制

5	2 h	±2 min
6	4 h	±5 min
7	6 h	±5 min
8	8 h	±5 min
9	10 h	±5 min
10	12 h	±5 min
11	14 h	±5 min
12	21h	±5 min
13	24 h	±5 min
14	27 h	±5 min
15	30 h	±5 min
16	33h	±5 min
17	36 h	±5 min
18	48h	±5 min
19	72 h	±5 min

7.13.3 样本的处理

7.13.3.1 生物样本的标识

在血液样本采集前对采血管及血浆样本冻存管进行统一编号，并粘贴专用标签（如下图所示）。

采血管标识字样

此处为医院项目编号
LB-RSRI-I-AICOMER
- XX
全血　　**min/h

冻存管标识字样

此处为医院项目编号
LB-RSRI-I-AICOMER
- XX
血浆A/B　**min/h

标识字样说明：标签中第一行为医院项目编号；第二行为方案编号；第三行####为受试者编号，XX 为采血点序号；第四行为样本类型：全血则表示用于真空采血管，血浆则表示用于冻存管，A 为检测血浆，B 为备份血浆；**min/h 表示受试者采血点。

7.13.3.2 PK 样本的采集

取静脉全血 4mL 于含 EDTA-K_2 抗凝剂的 4mL 采血管内，采血后立即轻轻颠倒试管 4-5 次，以确保血液与抗凝剂充分混合，避免血样与橡皮塞长时间接触。全血于采集后 1h 内完成离心（4℃，2000g，10min），取上层血浆分装于两个冻存管（规格：2mL）（检测血浆 A 和备份血浆 B）中，血浆样本于 2h（从血样采集开始计算）内转移至超低温冰箱（-60℃～-90℃）中，以供生物检测分析。

7.13.3.3 PK 样本的转运

严禁复制

整个试验的样本采集结束后，血浆将由冷链公司采用-60℃~-90℃的样本运输线运输至检测单位进行血药浓度测定。运输过程温度实时监控，不可颠倒。

7.13.4 血浆中药物浓度的检测方法

采用 LC-MS/MS 法测定人血浆中罗哌卡因游离碱的浓度。

本方法严格遵守有关要求进行充分确证，确证项目包括特异性；基质效应；标准曲线、线性范围、定量下限；批内、批间变异；残留、稀释效应；回收率试验；标准品、内标放置性稳定性试验、母液稳定性试验、全血放置稳定性试验、冻融稳定性试验、重复进样稳定性试验、制备后稳定性试验、室温放置稳定性、长期稳定性等。

7.13.5 药代动力学参数的估算和评价

根据试验中测得的各受试者的血药浓度-时间数据，绘制各受试者的药-时曲线及平均药-时曲线，并计算药代动力学参数（均数和标准差），主要药代动力学参数包括：T_{max}、C_{max}、AUC_{0-t}、$AUC_{0-\infty}$、Vd、Kel、$t_{1/2}$、CL、MRT 等。

7.14 药效学研究

7.14.1 试验设计

（1）镇痛范围测量（试验 1）：

于单点给药后 15min、30min、45min±3min，1h、1.5h、2h、3h、4h、5h、6h、7h、8h、9h、10h、11h、12h ±5min，沿两个方向确定疼痛点（当距离给药点为 1cm 的测定点（n）的 VAS 值≥4 时，测定至（n+1）时间点时可终止测量），测量并记录疼痛点和给药点之间的距离（r1，r2），镇痛范围直径 D =（r1+r2）。

（2）镇痛维持时间、起效时间和针刺疼痛强度的测量（试验 2）：

- 于给药前和给药结束后 15min、1h、4h、8h、10h、12h、14h、18h、21h、24h、27h、30h、33h、36h、48h、72h 测量针刺疼痛强度。
- 痛觉基线值测量：于给药前，采用视觉模拟评分法（visual analogue scale，VAS）测量痛觉测试区域 VAS=4、5、6 的疼痛压力基线值（g），各重复 3 次，分别取平均值，即基线值。
- 针刺疼痛强度测量：

  1）于空白区域施加 VAS=4 的基线值，提醒受试者记住该感觉；

  2）于痛觉测试区域，施加该区域 VAS=4 基线值的压力，并询问受试者与空白区域感受的比较，感觉为“弱于”、“相似”、“强于”；

严禁复制

3）当 “弱于”时，依次给予 VAS=5、6 时的基线压力并重复该询问过程；

4）当施加 VAS=6 压力，受试者感觉为“弱于”时，不再继续增加压力；

5）当“相似”时，记录该次施加的最大压力值；当“强于”时，记录前一次施加的最大压力值。

记录并统计镇痛维持时间、起效时间和针刺疼痛强度。

严禁复制

7.14.2 药效学指标

（1）镇痛起效时间：

镇痛起效时间的评价：给药后测量针刺疼痛感觉，首次出现测量痛感觉阈值大于该受试者基线期痛感觉阈值（即基线期 VAS=4 针刺压力平均值）的时间，记录为镇痛起效时间。

痛感觉阈：人体感受到疼痛时的最小刺激强度[32]。痛阈通常是指一种能够引起人体痛觉的刺激强度，一般分为痛感觉阈和耐痛阈（人体所能忍受的最大的刺激强度）[32]。

（2）镇痛持续时间：

镇痛有效指痛觉测量时人体的痛感觉阈大于基线期痛感觉阈。理论上，镇痛持续时间为镇痛有效开始时间与镇痛有效结束时间的差值。但在本研究实际操作过程中，无法实现实时动态监测，因此本研究对镇痛持续时间的记录分为镇痛持续最小时间和镇痛持续最大时间；镇痛持续时间真实值介于镇痛持续最小时间和镇痛持续最大时间之间。

镇痛持续最小时间= 末次测量到镇痛有效时间-首次测量到镇痛有效时间

镇痛持续最大时间=（末次测量到镇痛有效+1）测量时间-首次测量到镇痛有效时间

（3）针刺疼痛强度：

本研究针刺疼痛强度分为 3 级如下表所述：

三级	测量痛感觉阈值≥基线期 VAS=6 针刺压力值
二级	基线期 VAS=6 针刺压力值＞测量痛感觉阈值≥基线期 VAS=5 针刺压力值
一级	基线期 VAS=5 针刺压力值＞测量痛感觉阈值≥基线期 VAS=4 针刺压力值

（4）注射点的镇痛范围（试验 1）;

（5）同一圆周上相邻两个给药点之间的直线距离。

8 盲法设计

8.1 设盲原则

由于本研究的试验药和对照药的包装和性状均不同，无法做到对研究者实施盲法，因此，本试验采用设盲方法为受试者不知道自己接受哪种药物注射，以避免受试者的主观因素对疗效造成偏倚。

本研究试验 2 队列 A 随机分组需遵循盲法设计，受试者不可知道自己的分组情况，且给药研究者和痛觉测量研究者不可为同一人。

8.2 盲底保存

本研究试验 2 队列 A 中，每位受试者接受试验制剂或对照制剂的顺序将由随机表确定。随机表由统计单位应用 SAS（9.4 或更高版本）按 1:1 区组随机产生，所选择的 block 长度和随机种子数等作为保密数据一起密封于盲底中。

8.3 应急信件

在药物编盲同时，需为临床试验研究中心，提供应急信件。紧急揭盲时根据应急信件的提示，研究者可获取受试者的分组信息。

应急信件内密封有遇紧急情况揭盲的规定、应立即汇报的单位及期联系方式和地址。如拆阅应急信件，需注明拆阅者、拆阅日期、原因，并在电子病历报告表中记录。

8.4 紧急揭盲规定

在发生紧急情况（如严重不良反应）或受试者需要抢救必须知道接收的是何种处理时，经该中心主要研究者同意，由研究人员拆阅应急信件。应急信件一旦被拆阅，该编号受试者将退出试验，研究者应将原因记录在病历报告中，并告知申办方和 CRA。所有应急信件在试验结束后收回，以便试验结束后盲态审核。

9 安全性评价

所有接受过一次试验药物的受试者都将作为安全性分析的有效人群。受试者的体检结果、生命体征、不良事件和实验室检查异常值都会被总结。必须对受试者出现的不良事件进行密切监测。并参考 CTCAE（V5.0）标准对不良事件严重程度进行分级，同时应按照不良事件的严重性、严重程度及与试验药物的关系等方面进行评估。

研究者负责评估所有不良事件与试验药物之间的关系。但是，主要研究者可以授权参加本研究的其他有资格的临床医生来判断，但仍需要对此负责。研究者必须提供一份具有相应资格并接受授权的人员名单。

9.1 不良事件

9.1.1 不良事件的定义

不良事件是指受试者接受试验用药品后出现的所有不良医学事件，可以表现为症状体征、疾病或者实验室检查异常，但并不一定与试验用药品有因果关系。在临床研究中，不良事件可以是发生在接受试验用药品后，任何时间的不良医疗状况。

亚太原刊

AE 的例子包括：

1） 原有慢性疾病或间歇性疾病加重，包括发作频率和/或强度增加。

2） 试验药物给药后发现的或诊断的新的疾病，即使在研究开始之前可能已经存在。

3） 可疑相互作用的体征、症状或临床后遗症。

4） 试验药物或合并用药因药物过量而可疑的体征、症状或临床后遗症。

5） 与治疗给药有时间上相关性的体征、症状。

9.1.2 非预期不良事件

非预期不良事件是指任何特征或严重程度与研究者手册（或对照药说明书）不一致的药物不良事件。对已知、已经记载的不良事件的特征或严重程度的重要信息进行补充也属于非预期不良事件报告的组成部分。例如，比研究者手册中描述的更为特殊或更为严重的事件应视为“非预期的”。

9.1.3 不良事件的观察、记录及报告

受试者接受试验用药品后发生的所有不良事件，都需要完整地记录在受试者的病例报告表或 eCRF 中。

对每起不良事件的描述应包括其起止时间、是否符合严重不良事件、采取的措施（如研究治疗的改变、其他的治疗及后续检查）和转归，并请研究者进行因果关系评价（与研究治疗的关系）。对不良事件，应进行分级，其变化记录在相应的病例报告表或 eCRF 上。记录文件必须有原始资料支持。

9.1.4 发现不良事件的方法

每次随访时，可通过以下方法发现不良事件：

- 由受试者或看护者主动提供的信息
- 每次随访时，对受试者提出开放、非诱导性的问题：您的感觉如何？自从上次随访以来，您是否有任何（其它）的医学上的问题？
- 由研究者、其他医务人员及家属观察到的异常
- 经医学检验和检查发现的异常
- 其他

9.1.5 收集不良事件的时间

本研究中记录不良事件的时间是从受试者接受试验用药品后，直至受试者给药后 21 天。

9.1.6 不良事件严重程度判断标准

参考CTCAE（V5.0）标准判断不良事件严重程度。如果出现未列出的不良事件可参照下

表的标准：

不良事件判定标准

不良事件分级	严重程度描述
Ⅰ级：	轻度，无临床症状或有轻微临床症状；仅有临床或实验室检查异常；不需治疗。
Ⅱ级：	中度，需要微量的、局部的或非侵害性的治疗；与年龄相符的使用工具的日常生活活动（Activities of Daily Living，ADL）受限，使用工具的日常生活指做饭、购物、打电话等。
Ⅲ级：	病情重或有医学上严重的症状但是暂时不会危及生命；导致住院或住院时间延长；导致残疾；日常生活自理（Self care ADL）受限。日常生活自理指：洗澡、穿衣、脱衣、吃饭、去卫生间、吃药等，非卧床不起。
Ⅳ级：	危及生命，需要紧急治疗。
Ⅴ级：	因不良事件致死。

9.1.7 不良事件与试验药物的相关性评价 根据药物与不良事件因果关系判断标准，将不良事件与受试药物应用的相关性分为五级，肯定有关、很可能有关、可能有关、可能无关、肯定无关。将肯定有关、很可能有关、可能有关均列为药物不良反应。将药物不良反应病例数总和作为分子，全部可供不良反应评价的入选病例作为分母，计算不良反应发生率。

①肯定有关：符合所疑药物已知的反应类型，符合用药后合理的时间顺序，减量或停药后不良事件减轻或消失，再次给药后又出现该不良事件。

②很可能有关：符合所疑药物已知的反应类型，符合用药后合理的时间顺序，减量或停药后不良事件减轻或消失，但受试者临床状态或其它原因也可能产生该事件。

③可能有关：符合所疑药物已知的反应类型，符合用药后合理的时间顺序，减量或停药后不良事件减轻或不明显，但受试者的临床状态或其它原因可解释该事件。

④可能无关：不太符合所疑药物已知的反应类型，不太符合用药后合理的时间顺序，受试者的临床状态或其它原因也有可能产生该事件。

⑤肯定无关：不符合所疑药物已知的反应类型，不符合用药后合理的时间顺序，受试者的临床状态或其它原因也可解释该反应，排除临床症状或其它原因后，事件减轻或消失。

不良事件因果关系判断标准

	肯定有关	很可能有关	可能有关	可能无关	肯定无关
与试验药物有合理的时间顺序	+	+	+	—	—
已知的药物不良反应类型	+	+	+	—	—
停药后反应减轻或消失	+	+	±	±	—
再次给药后不良反应复出	+	?	?	?	—
无法用受试者疾病来解释	+	+	±	±	—

说明：⑴“+”表示肯定；“—”表示否定；“±”表示难以肯定或否定；“?”表示情况不明；⑵肯定有关、可能有关、无法

判定可认为是药品所引起的不良反应。

9.1.8 发生不良事件病例的治疗、随访和持续时间

研究者初次报告不良事件后需对该受试者进行密切随访，必要时采取相应的治疗措施，并提供相关信息给申办者。研究过程中的所有不良事件，需按临床试验中心的既定 SOP 进行处理，均应随访至基线水平或稳定或失访。

9.1.9 实验室检测指标异常的判断和处理

实验室检查数值的异常首先需由研究者和基线相比较并判断是否有临床意义；判断为有临床意义且和基线相比有变化的实验室检查数值异常作为不良事件报告；无临床意义的实验室检查数值异常不作为不良事件报告。所有的异常的实验室检查数值/生命体征应准确记录在相关的病例报告表或 eCRF 上。

9.1.10 可能出现的不良事件及抢救措施

9.1.10.1 可能出现的不良事件

根据国内外同类药物的临床经验，罗哌卡因类似物十分常见(≥1/10)的临床不良反应有：恶心、低血压。常见(≥1/100) 的临床不良反应有体温升高，僵直，背痛，心动过缓，心动过速，高血压，感觉异常，头晕，头痛，呕吐，尿潴留。偶见临床不良反应(≥1/1000)有低体温、昏厥、焦虑，中枢神经系统毒性症状(惊厥，癫痫大发作，癫痫发作，头晕，口周感觉异常，舌头麻木，听觉过敏，耳鸣，视觉障碍，构音障碍，肌肉抽搐，震颤)，感觉减退，呼吸困难。罕见临床不良反应(<1/1000)有过敏反应，最严重的情况是过敏性休克，心搏骤停，心律失常。

根据试验药物现有的临床前研究结果，长效罗哌卡因注射液主要是皮下注射，因其溶剂也具有刺激性，临床前药理毒理试验主要观察到的不良反应包括注射部位的肿胀、红斑、硬结、破溃、结痂、轻微的急性/亚急性皮下炎症、轻微或轻度肉芽组织形成/纤维化。停药后刺激性反应可恢复。

主要不良反应如下表：

十分常见(≥1/10)	
全身	恶心
循环系统	低血压
常见(≥1/100)	
全身	体温升高，僵直，背痛
循环系统	心动过缓，心动过速，高血压
中枢神经系统	感觉异常，头晕，头痛
消化系统	呕吐

严禁复制

肾脏及泌尿系统	尿潴留
偶见(≥1/1000)	
全身	低体温
循环系统	昏厥
中枢神经系统	焦虑，中枢神经系统毒性症状(惊厥，癫痫大发作，癫痫发作，头晕，口周感觉异常，舌头麻木，听觉过敏，耳鸣，视觉障碍，构音障碍，肌肉抽搐，震颤)，感觉减退
呼吸系统	呼吸困难
罕见(<1/1000)	
全身	过敏反应，最严重的情况是过敏性休克
循环系统	心搏骤停，心律失常

应对以上不良反应，需及时采取相应措施确保长效罗哌卡因注射液首次在人体 I 期临床研究的用药安全性，制定如下预期不良事件风险管控措施。

9.1.10.2 不良事件的风险管控

罗哌卡因注射液最常见的不良反应包括恶心和低血压以及全身神经系统、循环系统、呼吸系统、肾脏及泌尿系统偶见或罕见的不良反应，针对罗哌卡因可能出现的不良反应，在临床研究中重点从以下几个方面进行风险控制：

（1）首先在筛选入组受试者时，排除对同类药物过敏或属于过敏性体质的人群。

（2）入组用药前应向受试者充分告知该治疗方案可能导致的不良反应，减少受试者的焦虑。

（3）在给药期间和给药结束后 30min 内，需有麻醉经验的临床医生在场，其余在院观察期间，需有经过专业培训的临床医护人员严密监控。

（4）必要时进行心电监护。

（5）针对注射入血风险，严格按照皮下注射的标准方法操作，给药前，应仔细回吸以防止血管内注射，同时控制注射速度，缓慢注射。

（6）针对常见的不良反应呕吐，受试者需在试验期间清淡饮食。

（7）注射给药同时，密切观察病人的生命指征并持续与病人交谈，如出现中毒症状，立即停止注射。

（8）试验药品打开只能使用一次，剩余的液体必须抛弃。

（9）局部麻醉会轻微影响精神状况及共济协调，还会暂时损害运动和灵活性，所以，给

严禁复制

药后限制受试者的自由活动。

（10）对于采取的支持治疗等措施和检查应作为原始文件妥善保存。并严格按照相关 SOP 进行记录和报告。

9.1.10.3 不良反应的处理及抢救[9]

（1）如果出现急性全身毒性反应的现象必须立即停止注射。

（2）如果发生惊厥等中枢神经系统毒性反应，需及时按专业 SOP 进行应急处理。

（3）如果出现循环衰竭等循环系统毒性反应，需及时按专业 SOP 进行应急处理。

（4）如发生心跳停止，为了提高复苏成功率，可能应该延长复苏时间。

（5）如果出现呼吸系统毒性反应，需及时按专业 SOP 进行应急处理。

（6）如针头刺破血管，立即拔针，按压注射部位。

（7）如发生其他情况，研究者需按相应情况做相应应急处理。

9.2 严重不良事件

9.2.1 严重不良事件的定义[33]

严重不良事件（Serious Adverse Event, SAE），指受试者接受试验用药品后出现死亡、危及生命、永久或者严重的残疾或者功能丧失、受试者需要住院治疗或者延长住院时间，以及先天性异常或者出生缺陷等不良医学事件。

死亡：不可逆的昏迷或脑死亡，在死因源于心脏时描述为猝死。死亡和猝死明显不同，因而不得交换使用。

危及生命：在"严重的"定义中，术语"危及生命"指病人在事件/反应当时处于死亡危险，它并不是指假设将来更严重时可能引起死亡的事件/反应。

住院：导致受试者住院或住院受试者延长住院时间的任何不良事件都被认为是严重的，除非符合以下特例之一：

- 在医院留观不超过 12 小时；
- 入院是预先计划好的（即签署知情同意书前已安排好的手术或择期手术）；
- 入院与不良事件无关（如出于疗养目的的住院）。

残疾：意味着某人从事日常生活的能力严重受损。

9.2.2 可疑且非预期严重不良反应[33]

可疑且非预期严重不良反应简称 SUSAR（suspected unexpected serious adverse reaction）指临床表现的性质和严重程度超出了试验药物研究者手册、已上市药品的说明书或者产品特

严禁复制

性摘要等已有资料信息的可疑并且非预期的严重不良反应。

9.2.3 严重不良事件/妊娠的记录及报告[33]

受试者接受试验用药品后，直至完成用药后 21 天内发生的严重不良事件都应该上报。

严重不良事件报告的程序如下：

- 主要研究者需在获知 SAE 后的 24 小时内，立即填写《严重不良事件报告表》，并报告给申办者；
- 申办方对收到的安全性信息立即进行评估后，将判定为可疑且非预期严重不良反应快速报告给所有参加临床试验的研究者及临床试验机构、伦理委员会，并上报给药品监督管理部门和卫生健康主管部门，同时通报给所有参加临床试验的研究人员（药物警戒、数据管理等相关项目责任人）。

表 6 严重不良事件/可疑且非预期严重不良反应报告联系电话

报告单位	联系电话	传真
**严重不良事件需报告给以下单位：**		
主要研究者	010-83997322	010-63296483
伦理委员会	010-83997028	010-83997028
申办单位	029-81122714	029-81122714
北京海金格医药科技有限公司药物警戒部	010-83692660	010-83699580
北京爱康维健医药科技有限公司	010-52894249	010-52894249
**可疑且非预期严重不良反应还需上报给以下单位：**		
北京市药品监督管理局	010-83979465	010-83560723
陕西省药品监督管理局药品不良反应监测处	029-62288143<br>029-62288041	029-62288004<br>029-62288145
国家药品监督管理局药品注册管理司研究监督处	010-68313344	010-88363228
国家卫生和计划生育委员会医政医管局	010-68792201	010-68792734

申办者可能要求研究人员提供其它的信息以保证及时并准确的完成安全性报告。

研究人员必须采用所有必要的治疗措施解决 SAE。处理 SAE 所需的任何药物都必须记录在该受试者病例报告表或 eCRF 的合并用药中。

每一起 SAE 都应随访至基线水平或稳定或失访，并向指定人员递交更新报告。单纯的实验室检查异常不作为 SAE 报告，除非研究者认为该异常已达到 SAE 的标准。实验室检查异常应记录在“实验室资料”页中，并由临床监查员定期核对。

妊娠：所有给药后出现的妊娠及其结局（自然流产、选择性终止妊娠、异位妊娠、正常分娩或先天畸形等）均应书面报告伦理委员会及申办方。男性受试者给药后其配偶发生妊娠

也需要报告。

妊娠本身不作为不良事件或严重不良事件报告。若妊娠过程/结局符合 SAE 的标准，则研究者应按 SAE 报告程序报告。即使受试者退出研究，也应随访和记录妊娠结局。

如果妊娠发生在完成给药后 21 天内，研究者应知晓妊娠后 24 h 内通知申办方。

10 数据管理和统计分析

10.1 数据管理

10.1.1 CRF 的设计

CRF 根据方案中规定的试验步骤和流程图来设计，初稿形成后，需要项目经理、数据和统计人员、方案撰写人等项目组人员共同审核，符合方案并遵循相关的法律法规，且版本控制过程须进行完整记录。

10.1.2 CRF 填写指南

CRF 填写指南是根据研究方案对于病例报告表的每页表格及各数据点进行具体的填写说明。保证临床试验中心在入选受试者之前获得 CRF 及其填写指南，并对临床试验中心相关工作人员进行方案、CRF 填写和数据提交流程的培训，该过程需存档记录。

10.1.3 CRF 注释

注释 CRF 是对空白 CRF 的标注，记录 CRF 各数据项的位置及其在数据库中的变量名和编码。CRF 中的所有数据项都需要标注。需要 DM 审阅。

10.1.4 数据库的设计

数据库应按照注释 CRF 中的数据集名称、变量名称、变量类型和变量长度来建立，并尽量依从标准数据库的结构与设置。数据库建立完成后，应进行数据库测试，出具数据库测试报告，并由数据管理负责人签署确认。

10.1.5 权限分配

系统管理员根据不同角色，分别创建账户，授予不同权限。

10.1.6 eCRF 填写

研究人员需按照研究方案要求来收集受试者数据，并根据原始资料参照填写指南把信息准确、及时、完整、规范地填写到 eCRF 中。CRF 数据的修改必须遵照标准操作程序，保留修改痕迹。

10.1.7 质疑的发送和解决

数据管理部门 DM 列出详细的数据核查计划，核查计划由程序员和数据管理部门 PM 审阅无异议后签字确认，数据录入到 EDC 后，系统按照数据核查计划书中所建编辑核查（Edit Check）将对数据进行核查，有疑问的数据会自动发出系统质疑；无法设置为系统发出疑问的

严禁复制

数据会通过 EDC 发送人工疑问，录入人员或者研究者对人工质疑和系统质疑进行确认和回答，必要时修改错误数据，直至质疑解决。回答未能解决质疑时，数据管理员和临床监查员可以对该数据点进行一次再质疑，所有的留痕均保存在 EDC 数据库中。

10.1.8 数据的修改和审核

数据录入人员或研究者核实数据后可对数据进行修改，修改的数据需按照系统提示，在系统中填写修改理由。研究者对最终所有数据具有审核权限。

10.1.9 医学编码

临床试验中收集的病史、不良事件、合并药物治疗应使用标准的字典进行编码。一般使用的标准字典为 MedDRA, ATC。编码后的数据集应明确记录编码时所使用的字典和版本。

10.1.10 SAE 一致性比对

把 CRF 中所有 SAE 相关的数据点都与 PV 库中的数据点用程序做比对，不一致的数据需要与 PV 人员沟通，直至数据没有差异为止。

10.1.11 数据审核会

数据库锁定前，准备好数据管理报告初稿和所有的数据列表，由申办方、研究者、数据管理人员和统计分析师在盲态下共同最终审核数据中未解决的问题，并按照临床试验方案进行统计分析人群划分、 核查严重不良事件报告与处理情况记录等，数据审核会后要定稿数据管理报告和人群划分计划等。

10.1.12 数据库锁定与解锁

数据库锁定是临床研究过程中的一个重要里程碑。锁定过程和时间应有明确的文档记录，锁定是取消对数据库进行编辑的权限，并将收回数据编辑权限的日记记录在文档中。

数据库锁定后如有修改，需提出申请，经申办方、研究者、录入人员、临床监查员、数据管理人员讨论并签字确认方可执行，并详细记录需要解锁的原因。

10.1.14 数据保存

在进行临床试验的过程中，把所有收集到的原始数据（如 CRF 和电子数据）存储在安全的地方。这些原始文档是追踪到原始数据的审核路径的一部分，应如同电子审核路径对数据库的任何修改或备份所做记录一样，严格进行保护。

10.1.15 数据保密

数据保密是临床研发过程中必须遵守的基本原则，参与药物研发的机构应建立适当的程序保证数据库的保密性，包括建立及签署保密协议以规范相应人员的行为，以及建立保密系统以防止数据库的泄密。

严禁复制

数据传输过程中应对数据集进行加密后再传输，数据集和密码不可以在同一封邮件中传输。

10.2 统计分析

10.2.1 分析人群

- 安全性分析集（Safety Set，SS）：包括所有至少给予一次试验药物的受试者构成。安全性分析集用于基线特征和安全性分析。
- 药代动力学浓度分析集 (PK Concentration Set, PKCS）：包括所有接受试验药物治疗，且有至少一次血药浓度数据的受试者。药代动力学浓度分析集用于药代动力学浓度数据相关分析。
- 药代动力学参数分析集 (PK Parameter Set, PKPS）：包括所有接受试验药物治疗，且有至少一个药代动力学参数的受试者。药代动力学参数分析集用于药代动力学参数数据相关分析。
- 药效学分析数据集 (PD Set)：包括所有入组至少使用一次试验药物、且具有用药后药效学评价数据的受试者。药效学分析数据集用于药效学数据相关分析。

10.2.2 统计分析方法

10.2.2.1 入组及完成情况

分别计算筛选、入组、脱落和完成试验的受试者例数和百分比，并按照不同剂量组总结进行各分析集的受试者例数和百分比。

10.2.2.2 基线特征分析

基线数据包括：人口统计学特征、实验室检查、生命体征和合并用药等，按照剂量组进行汇总。对于计量资料采用均数、标准差、中位数、最大值、最小值进行描述；对于计数资料，采用频率及百分率进行描述。基线指标的组间比较采用团体 t 检验或 Wilcoxon 秩和检验、Fisher’s 确切概率法。基线分析基于安全性分析集进行。

10.2.2.3 安全性/耐受性分析

安全性/耐药性分析包括不良事件、临床实验室检查结果评估、生命体征。安全性/耐受性分析基于安全性分析数据集。

10.2.2.3.1 不良事件

对任何接受了试验药物治疗的受试者，均需进行安全性评估。评价时间应从受试者接受试验用药品后到研究结束或给药后 21 天内，对于在这期限出现的 AEs 或 SAEs 应进行追踪观

严禁复制

察直到其恢复正常或稳定为止（死亡、失访、撤回知情同意的受试者除外）。

采用 MedDRA 对不良事件进行编码。本试验主要对于治疗中出现的不良事件（TEAE, Treatment Emergent Adverse Event）进行统计分析，试验在筛选期至首次用药前发生的不良事件以清单形式列出，但不纳入不良事件的统计分析。下文中的不良事件均指治疗中出现的不良事件。

分别计算各组所有不良事件、与研究药物有关的不良事件、严重不良事件和导致脱落的不良事件的发生例次、例数和发生率。按照系统器官分类（SOC）和首选术语（PT）两级分类，分别计算两组各类不良事件的发生例次、例数和发生率。分别列出治疗中出现的与研究药物有关的不良事件清单、所有不良事件清单、导致脱落的不良事件清单和严重不良事件清单。

10.2.2.3.2 临床实验室检查结果评估

实验室数据将根据实验室检查的类型进行总结。正常值范围和异常有意义标准将在对实验室数据的总结中作为参考。与基线相比较的变化将采用治疗前-治疗后的交叉表进行分析。

10.2.2.3.3 生命体征

在各指定随访时间点对腋下体温、脉搏及卧位血压（收缩压和舒张压）以及相对于基线的变化进行描述性统计，计算其均数、标准差、中位数、最小和最大值。

10.2.4 药代动力学分析

用试验中测得的各受试者血药浓度(c) -时间(t)数据，绘制 c-t 线性和半对数图。同时，对各时间点的血药浓度进行统计学描述，包括例数、几何均数、变异系数、最小值和最大值等。

计算各受试者的药代动力学参数，包括：Tmax、C_{max}、AUC_{0-t}、$AUC_{0-\infty}$、Vd、Kel、$t_{1/2}$、CL、MRT。对不同剂量组、以及试验组和对照组的药代动力学参数统计学描述，包括例数、几何均数、变异系数、几何变异系数、最小值和最大值等。

10.2.5 药效学分析

药效学指标以均数、标准差、最大值、最小值、中位数进行描述，并与筛选期基础值进行比较，采用配对 t 检验比较组内用药前后差异。各组之间采用与基线差值的（将基线值作为协变量）最小二乘均数和 95%可信区间进行比较。

采用混合效应模型对给药后不同时间点的针刺疼痛强度较给药前的变化进行分析，模型以不同给药区域各时间点较基线的疼痛强度变化值为应变量，给药前的疼痛强度为协变量，分组（试验组、对照组）、给药区域/空白区域、访视点、以及分组与访视点间的交互作用为固定效应，考虑个体因素。根据模型计算给药不同时间点疼痛强度较基线变化值的均数及其

95%可信区间，以及试验组与对照组差值的均数及其 95%可信区间。采用类似的方法对镇痛范围进行统计分析。

采用 Kaplan-Meier 方法对镇痛起效时间和镇痛维持时间进行统计分析，计算镇痛起效时间和镇痛维持时间的中位数、Q1、Q3 及其 95%可信区间。

10.2.6 缺失数据的处理

本试验不考虑对安全性分析、药代动力学分析和药效学分析中的缺失数据进行处理。

11 研究管理

11.1 监查

为了保证临床试验中受试者的安全和权利受到保障，试验记录与报告的数据准确、完整无误，保证试验遵循已批准的方案和有关法规，申办方将指定有适当资质的临床监查员作为申办者与研究者之间的主要联系人，对参加研究的各中心进行监查。

在研究过程中，研究者应允许申办方的监查人员或代表到研究机构检查研究相关文件（如受试者知情同意书、试验药物清点表、伦理委员会的批准文件）。监查人员到访时会对受试者的档案进行严格审阅，以核对记录在病例报告表或 eCRF 中的信息，尤其是安全性有关的关键信息和受试者的原始记录是否吻合。

监查活动应遵循标准操作规程，以督促临床试验的进行和保证临床试验按方案执行。

11.2 原始数据核查

本研究要求西安力邦制药有限公司授权委托的临床监查员可以直接核对原始数据进行数据核查，通过将受试者的病例报告表或 eCRF 上的数据和其原始病历上的数据进行核对实现。

11.3 稽查和视察

西安力邦制药有限公司的代表、法规部门、伦理委员会可以到研究中心、中心实验室以及数据管理与统计单位进行稽查或视察。

11.4 人员培训

主要研究者将保留一份所有研究相关人员（医生、护士和其他人员）的研究培训记录。主要研究者要保证所有这些人员已受到与研究有关的适当的培训，并且与进行研究相关的任何信息已传递给相关人员。

11.5 资料收集和处理

临床试验研究者或临床研究协调员（CRC）应依照病例报告表或 eCRF 填写指南，准确、及时、完整、规范地填写病例报告表或 eCRF。完整的病例报告表或 eCRF 必须有研究者签名

严禁复制

及日期。

研究者必须保存研究记录和数据，包括电子源数据和电子文档。每个数据点都必须在研究中心有原始文档支持。凡被作为原始信息的记录或者文档（即受试者原始医疗文件）需妥善保管以供申办者的稽查及监管部门的视察。

11.6 受试者保密

有关受试者身份的所有记录均予以保密，并且在相关法律和/或法规允许的范围之外，这些资料将不对外公开。受试者的姓名不会提供给申办方。病历报告表中仅记录受试者编号和姓名的缩写。如果受试者的姓名等身份信息出现在任何其它文件（例如病理报告）中，在将文件的副本提供给申办方之前必须将这些信息隐去。采用电脑储存的研究报告必须符合当地有关资料保护的法律。如果研究的结果发表，受试者的个人身份仍将保密。研究者将保留一份名单以便核实受试者的身份信息。

11.7 受试者依从性

在招募筛选阶段，详细介绍本试验的目的、试验用药品的基本情况、研究方案、给药流程、给药方案（如剂量、给药方式等）、临床观察、生物样本采集的频次和流程、参加试验的潜在风险、补偿和赔偿等，使受试者充分知情，自愿参加，提高服药的依从性；在给药前，仔细核对受试者编号，给药剂量和顺序；给药过程中，要密切观察受试者状态，随时沟通受试者感受，并细致控制皮下注射操作；给药结束后，要认真观察受试者反应和给药部位的变化，必要时实施适当医疗措施；严格按照试验方案要求进行随访，以确证受试者的安全。

11.8 方案的修改

当对研究方案的修改影响研究目的、研究设计、研究执行、受试者的安全性、例数、研究的可能利益受试者以及受试者研究的程序等时，必须将研究方案的修正意见提交伦理委员会并得到申办者、研究者和伦理委员会的同意方能执行。

11.9 方案偏离或违背

研究方案中规定的所有要求，必须严格执行。任何有意或无意偏离或违反试验方案和 GCP 原则的行为，均可归类为偏离方案或违反方案。监查员在监查过程中，如果发现偏离方案时应由研究者或监查员填写偏离方案记录，详细记录发现的时间、事件发生的时间及过程、原因及相应的处理措施，由研究者签字，并通报伦理委员会及申办者。在数据统计和总结报告中，研究者对发生的方案偏离或违背对最终数据和结论的影响进行分析和报告。

当发生严重方案违背时，应进行评估。必要时，申办方可提前终止研究。

12 伦理要求和知情同意

12.1 伦理委员会

研究方案和受试者知情同意书在研究开始前必须提交到研究单位伦理委员会审批，伦理委员会应将其批准意见用书面形式送交研究者，研究人员需将伦理委员会批准的文件复印件以及伦理委员会的成员和资格送交给申办方。如有方案及知情同意书等文件的修订，也需要再次经伦理委员会审批。

在研究过程中出现的任何严重不良事件，研究者应报告给伦理委员会。在研究结束时，应书面通知伦理委员会。

12.2 知情同意书

知情同意书应符合最新版本的赫尔辛基宣言的要求并符合中国国家药品监督管理局制定的规则和指南。受试者在被筛选之前应被告知试验药物的性质、研究目的、可能的利益和可能的风险。研究者应将试验的过程和可能出现的问题向受试者解释清楚，受试者应在知情同意书上签名和填写日期以表示同意。特殊情况下允许有第三方见证人时获得受试者的受试者的口头同意，由见证人签名和填写日期。受试者或其合法代表将保存一份已签署的知情同意书，另一份已签署的知情同意书将保存在临床研究者的研究记录中。

13 质量控制和质量保证

为保证试验质量，在试验正式开始前，由申办方和研究者共同讨论、制定临床研究方案和研究计划。对参加试验的有关研究人员进行研究方案培训和 GCP 培训。

试验中心必须按照 SOP 管理研究用药，包括接收、保管、分发、回收及销毁（如适用）。

根据 GCP 指导原则，在试验的设计和实施阶段应采取必要的步骤，以保证所收集的数据准确、一致、完整和可信。临床研究中所有观察到的结果和异常发现，均应及时核实、记录，保证数据的可靠性。临床研究中各种检查项目所使用的仪器、设备、试剂、标准品等，均应有严格的质量标准，并确保其是在正常状态下工作。

研究者将方案所要求的信息输入到电子病理报告表中，由监查员核实其填写是否完整和准确，并指导试验中心的工作人员进行必要的修正和补充。

药品监督管理部门、机构审查委员会（IRB）/伦理委员会（EC）、申办方的监查员和/或稽查人员可能会对临床研究相关活动和文件进行系统性检查，以评价试验是否按照研究方案、

严禁复制

SOP 以及相关法规的要求进行，试验数据是否及时、真实、准确、完整地记录。稽查应由不直接涉及该临床研究的人员执行。

14 论文发表

在研究结果发表或公布之前，申办方保留对初稿进行审阅的权力。从本研究中获得的所有资料和结果以及全部知识产权均属申办方所有，申办方可以以各种形式利用这些资料，例如呈送政府药监部门或向其他研究者公开。与此同时，研究人员出于科学的目的可以单独使用从本研究中获得的资料，但在发表前必须同申办方协商并得到申办方的书面同意。申办方认可研究完成后研究者有权发表研究结果。然而，研究人员必须在投稿前 45 天将文章的草稿或摘要送交申办方，以便在投寄待发表的文章最终版本前获得申办方的核准。申办方将对文章及时进行审查，且不会无理拒绝批准。当申办方和研究者出现意见分歧时，应就欲发表的内容进行讨论，以便达成令双方都满意的解决方式。

15 记录和资料保存

研究者应当使所有临床研究必需的相关文件资料保存完整，包括受试者的原始病历、知情同意书、病例报告表和 eCRF、采血记录、药物分发详细记录等，保存期限按照我国 GCP 原则应至试验结束后 5 年。但若现行法规或与申办者的协议中有要求，这些资料还应保存更长的时间。申办者将以书面形式通知研究者何时这些资料将不再需要保存。最晚在期满前 6 个月，研究者需要联系申办方商定研究资料的继续保存或移交事宜。本临床研究的研究方案、知情同意书、病例报告表和 eCRF、研究者手册的所有权属于西安力邦制药有限公司，除国家食品药品监督管理总局要求外，未经申办方书面同意，研究者不得以任何形式提供给第三方或者自行处理。申办者应保存临床试验资料至试验药物被批准上市后五年。

严禁复制

16 参考文献

[1] Hansen TG. Ropivacaine: A pharmacological review. Expert Rev Neurother. 2004;4:781－91.

[2] Markham A, Faulds D. Ropivacaine[J]. Drugs, 1996, 52(3): 429-449.

[3] 曹永孝、朱银静等，长效罗哌卡因注射液对大鼠术后疼痛的缓解作用，西安交通大学医学部，2013.10.~12.，公司内部资料.

[4] 曹永孝、鹿静等，长效罗哌卡因注射液对正常大鼠疼痛的缓解作用，西安交通大学医学部，2018.2.，公司内部资料.

[5] 曹永孝、鹿静等，长效罗哌卡因注射液对大鼠术后疼痛的缓解作用，西安交通大学医学部，2018.6.，公司内部资料.

[6] 黄晓星、王一飞等，长效罗哌卡因注射液对麻醉家兔神经传导的阻滞作用，上海医药工业研究院，公司内部资料.

[7] 李腾飞、樊惠等，长效罗哌卡因注射液对神经源疼痛模型上镇痛作用维持时间的比较，上海交通大学药学院，2013.3~4，公司内部资料.

[8] 李腾飞、樊惠等，长效罗哌卡因注射液对硬膜外麻醉持续时间和潜伏期的影响，上海交通大学药学院，2013.1~3.，公司内部资料.

[9] 周力强、曹芸吉等，长效罗哌卡因注射液巴马小型猪对伤口愈合的影响试验，昭衍(苏州)新药研究中心有限公司，2013.12. 公司内部资料.

[10] 顾丰华、顾骏等，长效罗哌卡因注射液猪皮肤镇痛药效学总结报告(一)，上海医药工业研究院，2018.8.~11. 公司内部资料.

[11] 顾丰华、顾骏等，长效罗哌卡因注射液猪皮肤镇痛药效学总结报告(二)，上海医药工业研究院，2018.12. 公司内部资料.

[12] 张天竺、操佳佳等，长效罗哌卡因注射液单次皮下注射给药对清醒无束缚 Beagle 犬的心血管及呼吸系统功能的影响，昭衍(苏州)新药研究中心有限公司，2016.8.~9.，公司内部资料.

[13] 张天竺、操佳佳等，长效罗哌卡因注射液单次皮下注射给药对 SD 大鼠中枢神经系统功能的影响，昭衍(苏州)新药研究中心有限公司，2016. 9.，公司内部资料.

[14] 盐酸罗哌卡因注射液(耐乐品)说明书.

[15] 张冬霞、孟小巧等，长效罗哌卡因注射液单次皮下注射给予 SD 大鼠的毒性试验，昭衍(苏州)新药研究中心有限公司，2013.12.~2014.1.，专题号: 13-0227SD，公司内部资料.

[16] 张冬霞、曹静等，长效罗哌卡因注射液溶剂单次皮下注射给予 SD 大鼠的毒性试验，昭衍(苏州)新药研究中心有限公司，2014.1.~5.，专题号: R13-S033-SD，公司内部资料.

[17] 张冬霞、冯瑛等，长效罗哌卡因注射液单次皮下注射给予 Beagle 犬的毒性试验，昭衍(苏州)新药研究中心有限公司，2014.1.~2.，专题号: 13-0234SD，公司内部资料.

[18] 李小川、张伟玮等，长效罗哌卡因注射液皮下注射给予 Beagle 犬的药代动力学试验，昭衍(苏州)新药研究中心有限公司，2013.12.，公司内部资料.

严禁复制

[19] 周力强、张丽等，长效罗哌卡因注射液给予豚鼠的全身主动过敏试验，昭衍(苏州)新药研究中心有限公司，2013.12.~2014.3.，公司内部资料.

[20] 周力强、仲启秀等，长效罗哌卡因注射液对兔红细胞的体外溶血试验，昭衍(苏州)新药研究中心有限公司，2014.1.~3.，公司内部资料.

[21] 周力强、曹芸吉等，长效罗哌卡因注射液皮下注射给予家兔的局部刺激性试验，昭衍(苏州)新药研究中心有限公司，2013.11.~12.，公司内部资料.

[22] 曹永孝、鹿静等，不同处方长效罗哌卡因注射液刺激性试验，西安交通大学医学部，2018.11.，公司内部资料.

[23] 曹永孝、鹿静等，不同处方罗哌卡因注射液刺激性试验，西安交通大学医学部，2017.9.~12.，公司内部资料.

[24] 曹永孝、鹿静等，不同处方罗哌卡因注射液刺激性试验，西安交通大学医学部，2017.9.~12.，公司内部资料.

[25] 国家食品药品监督管理总局. 健康成年志愿者首次临床试验药物最大推荐起始剂量的估算指导原则. 2012-05-15, http://www.nmpa.gov.cn/WS04/CL2196/323902.html.

[26] FDA：Guidance for Industry.Estimating the Maximum Safe Starting Dose in Initial Clinical Trials for Therapeutics in Adult Healthy Volunteers. July 2005. https://www.fda.gov/downloads/Drugs/GuidanceComplianceRegulatoryInformation/Guidances/UCM078932.pdf.

[27] 黄继汉,黄晓晖,陈志扬,郑青山,孙瑞元.药理试验中动物间和动物与人体间的等效剂量换算[J].中国临床药理学与治疗学,2004(09):1069-1072.

[28] 王兴河.药物早期临床试验[M]. 2018 年 8 月.北京科学技术出版社.

[29] Ginosar Y, Haroutounian S, Kagan L, et al. Proliposomal ropivacaine oil: pharmacokinetic and pharmacodynamic data after subcutaneous administration in volunteers[J]. Anesthesia & Analgesia, 2016, 122(5): 1673-1680.

[30] 王庆利，张凤琴，赵德恒. FDA 发布经皮仿制药对皮肤刺激性和过敏性临床试验的设计及评分系统[J]. 中国临床药理学杂志，2004，20（6）：459-461.

[31] 国家药品监督管理局. 化学药物临床药代动力学研究技术指导原则[EB/OL]. 2005-03.

[32] 毕海金，吴国程，陈红云，等. 人体痛阈测定方法及其应用研究进展[J].中国疼痛医学杂志，2015，12（1）：60-62.

[33] 国家药品监督管理局，国家卫生健康委. 药物临床试验质量管理规范（2020 年第 57 号）[EB/OL].2020-04-23.

附件 1：长效罗哌卡因注射液人体I期临床研究所需全血及空白血浆样本的采集

1 采集目的

本试验的目的是为了满足长效罗哌卡因的药代动力学生物样本检测方法开发的需求，通过采集健康受试者全血及空白血浆，以达到方法开发等要求。

2 受试者选择

2.1 受试人群及人数

健康成年受试者 6 名。

2.2 入选标准

（1）充分了解试验目的和要求，自愿参加并签署经伦理委员会批准的知情同意书者；

（2）年龄为 18-50 周岁（包括 18 周岁和 50 周岁）的中国成年男性和女性，男女均有；

（3）体重指数（BMI）在 19.0~26.0 kg/m^2（包括临界值）；BMI=体重（kg）/身高 ^{2}（m^2）；

2.3 排除标准

（1）筛选期体格检查、生命体征、心电图检查、实验室检查（包括血常规、尿常规、血生化等），研究者判断异常有临床意义者；

（2）筛选前 3 个月每日吸烟量大于 5 支者；

（3）筛选前 12 个月内有酗酒史者（即男性每周饮酒超过 28 个标准单位，女性每周饮酒超过 21 个标准单位（1 单位相当于啤酒 285mL，或烈酒 25 mL，或葡萄酒 150 mL），或筛选前 6 个月内经常饮酒（每周饮酒超过 14 个标准单位）者；或酒精呼气检测为阳性者；

（4）既往长期饮用过量（一天 8 杯以上，1 杯=250mL）茶、咖啡或含咖啡因的饮料者；

（5）筛选前三个月内有过失血或献血达 400mL 者，或计划在研究期间或研究结束后一个月内献血者；

（6）采血困难或有晕针晕血史者；

（7）参加试验前四周内曾动手术或有急性病症发作者；

（8）正在参加其他临床试验或在采血前 3 个月内参加过其它药物试验的受试者；

（9）受试者理解、交流和合作能力不够，不能保证按方案进行者；

（10）精神或躯体上的残疾患者；

（11）有心、肝、肾、血液、呼吸道、消化道、神经系统、代谢异常等疾病者；

（12）筛选前 3 个月内用过已知对主要脏器有损害的药物者；

（13）筛选前两周内服用过任何处方药、非处方药、中草药、保健品者；

（14）药物滥用筛查（吗啡、甲基安非他明、氯胺酮、二亚甲基双氧安非他明、四氢大麻酚酸）阳性或筛选前 1 年内有药物滥用史者；

（15）受试者 HBsAg，HIV，HCV 和梅毒抗体检查阳性者；

（16）女性处于哺乳期，或血妊娠检查为阳性者；

（17）研究者认为有不适合参加试验的其他因素。

3 受试者编号

在筛选时，每名受试者将使用筛选号进行识别。试验的第-1 天经入排标准筛选后研究中心将给予每名合格的受试者一个编号（例如 Blank001），按照筛选号从小到大获得受试者编号。

4 研究流程

4.1 筛选期（第-14～-2 天）

在给药前-14天至-2天，受试者签署知情同意书之后，进行体格检查、实验室检查及其他相关检查（见空白血采集流程表），来决定是否符合入选/排除标准。在筛选时，每名受试者将使用筛选号进行识别。研究者根据入选/排除标准判定受试者是否能参与本研究。

受试者于第-1天下午17:30前入住药物临床试验中心病房，所有受试者进行生命体征、体格检查、药筛试验、酒精呼气检查和问诊，女性受试者加查血妊娠，不得自带任何食品或饮料等。入住病房期间需要统一清淡饮食。

4.2 空白血生物样本采集及处理（第 1 天）

每名受试者采集空白全血约100 mL，结束后方可离开药物临床试验中心。

每名受试者使用10个10mL EDTA-K_2真空采血管采集全血约100 mL，每管轻柔颠倒4-5次混匀。

全血留样：选择筛选号顺序的后3例受试者的最后1管（约10mL）全血留样，不离心，1小时内保存于2-8℃冰箱。尽快通过专业冷链公司运至检测单位，运输时2-8℃冷链保存，运输过程温度实时监控，并告知运输人员不可颠倒、剧烈晃动样本，以免细胞破碎。

空白血浆：其余血样，以2000 g离心力进行低温（4℃）离心10分钟。抽取上层血浆，分别转移至贴有标签的冻存管（规格：5mL）中，空白血浆样本存于-60℃~-90℃冰箱冷冻保存至运往检测单位。

5 空白血生物样本标识

在血液样本采集前对采血试管及血浆样本冻存试管进行统一编号，并粘贴专用标签。标签样本如下所示：

此处为医院项目编号<br>LB-RSRI-I-AICOMER<br>受试者编号：Blank001<br>血浆 1	此处为医院项目编号<br>LB-RSRI-I-AICOMER<br>受试者编号：Blank001<br>全血 1

标签中第一行为医院项目编号；第二行为方案编号；第三行为受试者编号；第四行为样本类型：“全血”字样标签用于真空采血管，“血浆”字样标签用于冻存管，1 表示第 1 管，以此类推。

6 生物样本转移及保存

全血和空白血浆采用专业的生物样本运输公司包装并运输，全血以 2-8℃冷链条件运输，空白血浆以-60~-90℃条件的冷冻条件运输及时送往分析检测单位。分析检测单位将空白血浆保存于-70±10℃冰箱内待用。

7 空白血采集流程表

长效罗哌卡因注射液人体I期临床研究
所需全血及空白血浆采集试验流程表

访视描述	筛选期（天）		血样采集
**时间（天）**	**-14-（-2）**	**-1**	**1**
知情同意	X		
人口学资料	X		
既往史和现病史 [1]	X		
个人史及家族史 [2]	X		
体格检查 [3]	X	X	X
生命体征 [4]	X	X	X
血常规 [5]	X		
血生化 [6]	X		
血清学检查 [7]	X		
血妊娠试验 [8]	X	X	
尿常规 [9]	X		
酒精呼气试验及药物滥用筛查 [10]		X	
12 导联心电图 [11]	X	X	
入/排标准	X	X	
入住		X	
血液样本采集			X
不良事件	X	X	X
离开病房			X

严禁复制

备注：

1） 须获取受试者的既往病史及现病史情况，包括：消化系统、中枢神经系统、心血管系统、肾脏、呼吸系统疾病史等。

2） 个人史应包含婚姻史、吸烟史、饮酒史、药物滥用史及过敏史；家族史。

3） 体格检查包括头部、皮肤、粘膜、浅表淋巴结、颈部、胸部、腹部、脊柱/四肢。在筛选期检查 2 次、d1 检查 1 次。

4） 生命体征包括腋下体温、卧位血压、脉搏、呼吸、血氧饱和度。

5） 血常规检查内容应包括红细胞计数（RBC）、白细胞计数（WBC）、血红蛋白（HGB）、红细胞压积（HCT）、血小板计数（PLT）、嗜酸性细胞计数（EOS）、嗜碱性细胞计数（BASO）、中性粒细胞计数（NEUT）、淋巴细胞计数(LYMPH)、单核细胞计数（MONO）。筛选期进行检查。

6） 血生化检查内容应包括钾（K+）、钠（Na+）、氯（Cl-）、钙（Ca^{2+}）、镁（Mg^{2+}）、磷（P）、血糖（GLU）、肌酐（CREA）、尿素（UREA）、谷氨酰胺转肽酶（GGT）、尿酸（UA）、乳酸脱氢酶（LDH）、总胆红素（TBIL）、天门冬氨酸氨基转移酶（AST）、丙氨酸氨基转移酶（ALT）、碱性磷酸酶（ALP）、甘油三酯（TG）、总胆固醇（CHOL）、总蛋白（TP）、白蛋白（ALB）。在筛选期进行检查。

7） HBsAg、HCV 抗体、HIV 抗体、梅毒特异性抗体。

8） 女性必须在筛选期、-1 天内进行血妊娠试验检查。

9） 尿常规检查内容应包括葡萄糖（GLU）、酮体（KET）、蛋白质（PRO）、尿潜血（BLD）、白细胞（WBC）、胆红素（BIL）、亚硝酸盐（NIT）、酸碱度（pH）、尿胆原（UBG）、比重（SG）；筛选期检查 1 次。

10） 酒精呼气试验及药物滥用筛查（包括吗啡、甲基安非他明、氯胺酮、二亚甲基双氧安非他明、四氢大麻酚酸）在筛选期进行筛查。

11） 12 导联心电图在筛选期、-1 天进行检查。

严禁复制
