# Supplementary material for: A randomized trial: The safety, pharmacokinetics and preliminary pharmacodynamics of ropivacaine oil delivery depot in healthy subjects
Source: PLoS One. 2023 Sep 19;18(9):e0291793. doi: 10.1371/journal.pone.0291793 (PMC10508611; doi:10.1371/journal.pone.0291793)
Supplement: Attachment — Submitted filename: PONE-D-22-07472_R1_reviewer.pdf [file pone.0291793.s004.pdf]

PLOS ONE

A randomized trial: the safety, pharmacokinetics and preliminary pharmacodynamics of ropivacaine oily delivery depot in healthy subjects

--Manuscript Draft--

**Manuscript Number:**	PONE-D-22-07472R1
**Article Type:**	Research Article
**Full Title:**	A randomized trial: the safety, pharmacokinetics and preliminary pharmacodynamics of ropivacaine oily delivery depot in healthy subjects
**Short Title:**	The safety and effect of ropivacaine oily delivery depot in healthy subjects
**Corresponding Author:**	Yong-xiao Cao<br>Xi'an Jiaotong University<br>xi'an , CHINA
**Keywords:**	ropivacaine oily delivery depot, safety, pharmacodynamics, pharmacokinetics, healthy subject.
**Abstract:**	Abstract<br>Introduction<br>Ropivacaine oily delivery depot (RODD) can slowly release ropivacaine and block nerves for a long time. The aim of the present work was to investigate the safety, pharmacokinetics, and preliminary pharmacodynamics of RODD in subcutaneous infiltration into healthy subjects.<br>Methods<br>The abdomens of 3 subjects were subcutaneously infiltrated with single-needle RODD containing 12~30 mg of ropivacaine. The irritation, nerve blocking range and optimum dose were investigated. Forty-one subjects were divided into RODD containing ropivacaine 150, 230, 300, 350, 400 mg and ropivacaine hydrochloride injection (RHI) 150 mg groups. Multineedle subcutaneous infiltration of RODD or RHI was performed in the abdomens of the subjects. The primary endpoint was safety or a 400 mg maximum dose. Subjects’ vital signs were observed, their blood was analyzed, their cardiovascular, nervous systems, and irritation were monitored. Second, the ropivacaine in plasma was determined, pharmacokinetic parameters were calculated, and the anesthetic effects of RODD were studied, including RODD onset time, duration and intensity of nerve block.<br>Results<br>RODD 24 mg in a single-needle injected infiltration in 3 subjects was optimal, and the range of nerve block was 42.5±20.8 mm. Multineedle subcutaneous infiltration of RODD in the abdomens of subjects was safe, and all adverse events were no more than grade II. The incidence rate of grade II adverse events, such as pain and abnormal ST and ST-T segment changes on electrocardiography, was approximately 1%. The incidence rate of grade I adverse events, including erythema, papules, hypertriglyceridemia, and hypotension was more than 10%. Erythema and papules were relieved after 24 h and disappeared after 72 h. Other adverse reactions disappeared after 7 days. The curve of ropivacaine concentration-time in plasma presented a double-peak profile. The results showed that ropivacaine was slowly released from RODD. Compared with the 150 mg RHI group, Tmax in the RODD groups was obviously longer. In particular, Tmax in the 400 mg RODD group of 11.8±4.6 h was longer than that in the RHI group of 0.77±0.06 h. The Cmax in the 150 mg RODD group was obviously lower than that in the 150 mg RHI group (0.35±0.09 vs. 0.58±0.13 μg·mL-1). In particular, the Cmax increased by 48% when the dose was increased by 2.6 times in the 400 mg group. Cmax and AUC were positively correlated with doses. Finally, the intensity of nerve block (≥level 2) was correlated with RODD doses. The 400 mg RODD group presented the strongest nerve block. The median of onset time was 0.42 h, and the duration median was 35.7~47.7 h.<br>Conclusions<br>RODD containing 400 mg of ropivacaine is safe when subcutaneously infiltrated into healthy subjects. Meanwhile, Tmax was delayed in the RODD group compared with the RHI group. The median duration of nerve block was more than 35 h. Therefore, RODD has an obvious sustained release effect.

Powered by Editorial Manager® and ProduXion Manager® from Aries Systems Corporation

**Order of Authors:**	Wu-dang Lu
	Dan-ling Zhao
	Mei-xia Wang
	Ya-qi Jiao
	Ping Chi
	Bo Ma
	Min Zhang
	Jian-ping Dong
	Hai-bo Zhang
	Yi Yang
	Ye Tian
	Min-quan Hui
	Bo Yang
	Yong-xiao Cao
**Response to Reviewers:**	Dear Dr. Maria Elisabeth Johanna Zalm,<br>Thank you for your kind consideration for evaluation of our manuscript PONE-D-22-07472, entitled “A randomized trial: the safety, pharmacokinetics and preliminary pharmacodynamics of ropivacaine oily delivery depot in healthy subjects”. Based on your comments and suggestions, we have modified our manuscript and responsed reviewers’ comments point-by-point showed in the following table (attachment 1). Meanwhile, a revised manuscript is also attached. The manuscript has been further polished and the certificate is showed the attachment 2.<br>In addition, the reviewers had confirmed that all the raw data were fully available at the first review of the manuscript. We have reviewed the raw data again. It had been uploaded to a public repository "ResMan" (www.Medresman.org.cn. ‘ResMan’ was associated with the Chinese Clinical Registry (ChiCTR)).<br>Supporting information, acknowledgments and author contributions were added to the manuscript. The attachments were added, including updated CONSORT checklist, chinese original protocol and english brief protocol.<br><br>We hope the revised version will meet your requirements.<br><br>We are looking forward to your reply.<br><br>Best Regards,<br><br>Yong-xiao Cao<br>Department of Pharmacology,<br>School of Basic Medical Sciences,<br>Xi’an Jiaotong University Health Science Center<br>yxy@xjtu.edu.cn
**Additional Information:**	
**Question**	**Response**
**Financial Disclosure**<br><br>Enter a financial disclosure statement that describes the sources of funding for the work included in this submission. Review the submission guidelines for detailed requirements. View published research articles from *PLOS ONE* for specific	The author(s) received no specific funding for this work.

examples.<br><br>This statement is required for submission and **will appear in the published article** if the submission is accepted. Please make sure it is accurate.<br><br>**Unfunded studies**<br>Enter: *The author(s) received no specific funding for this work.*<br><br>**Funded studies**<br>Enter a statement with the following details:<br>• Initials of the authors who received each award<br>• Grant numbers awarded to each author<br>• The full name of each funder<br>• URL of each funder website<br>• Did the sponsors or funders play any role in the study design, data collection and analysis, decision to publish, or preparation of the manuscript?<br>• **NO** - Include this sentence at the end of your statement: *The funders had no role in study design, data collection and analysis, decision to publish, or preparation of the manuscript.*<br>• **YES** - Specify the role(s) played.<br><br>* typeset	
**Competing Interests**<br><br>Use the instructions below to enter a competing interest statement for this submission. On behalf of all authors, disclose any competing interests that could be perceived to bias this work—acknowledging all financial support and any other relevant financial or non-financial competing interests.<br><br>This statement is required for submission and **will appear in the published article** if the submission is accepted. Please make sure it is accurate and that any funding sources listed in your Funding Information later in the submission form are also declared in your Financial Disclosure statement.	The authors have declared that no competing interests exist.

Powered by Editorial Manager® and ProduXion Manager® from Aries Systems Corporation

View published research articles from *PLOS ONE* for specific examples.<br><br>**NO authors have competing interests**<br><br>Enter: *The authors have declared that no competing interests exist.*<br><br>**Authors with competing interests**<br><br>Enter competing interest details beginning with this statement:<br><br>*I have read the journal's policy and the authors of this manuscript have the following competing interests: [insert competing interests here]*<br><br>* typeset	
**Ethics Statement**<br><br>Enter an ethics statement for this submission. This statement is required if the study involved:<br><br>• Human participants<br>• Human specimens or tissue<br>• Vertebrate animals or cephalopods<br>• Vertebrate embryos or tissues<br>• Field research<br><br>Write "N/A" if the submission does not require an ethics statement.<br><br>General guidance is provided below. Consult the submission guidelines for detailed instructions. **Make sure that all information entered here is included in the Methods section of the manuscript.**	The protocol of clinical study was approved by the Ethics Committee of Beijing You'an Hospital affiliated to Capital Medical University, China.<br>Approval number: LL-2019-117-S

**Format for specific study types**<br><br>**Human Subject Research (involving human participants and/or tissue)**<br>• Give the name of the institutional review board or ethics committee that approved the study<br>• Include the approval number and/or a statement indicating approval of this research<br>• Indicate the form of consent obtained (written/oral) or the reason that consent was not obtained (e.g. the data were analyzed anonymously)<br><br>**Animal Research (involving vertebrate animals, embryos or tissues)**<br>• Provide the name of the Institutional Animal Care and Use Committee (IACUC) or other relevant ethics board that reviewed the study protocol, and indicate whether they approved this research or granted a formal waiver of ethical approval<br>• Include an approval number if one was obtained<br>• If the study involved *non-human primates*, add *additional details* about animal welfare and steps taken to ameliorate suffering<br>• If anesthesia, euthanasia, or any kind of animal sacrifice is part of the study, include briefly which substances and/or methods were applied<br><br>**Field Research**<br><br>Include the following details if this study involves the collection of plant, animal, or other materials from a natural setting:<br>• Field permit number<br>• Name of the institution or relevant body that granted permission	
**Data Availability**<br><br>Authors are required to make all data underlying the findings described fully available, without restriction, and from the time of publication. PLOS allows rare exceptions to address legal and ethical concerns. See the PLOS Data Policy and FAQ for detailed information.	Yes - all data are fully available without restriction

A Data Availability Statement describing where the data can be found is required at submission. Your answers to this question constitute the Data Availability Statement and **will be published in the article**, if accepted.<br><br>**Important:** Stating 'data available on request from the author' is not sufficient. If your data are only available upon request, select 'No' for the first question and explain your exceptional situation in the text box.<br><br>Do the authors confirm that all data underlying the findings described in their manuscript are fully available without restriction?	
**Describe where the data may be found in full sentences. If you are copying our sample text, replace any instances of XXX with the appropriate details.**<br><br>• If the data are **held or will be held in a public repository**, include URLs, accession numbers or DOIs. If this information will only be available after acceptance, indicate this by ticking the box below. For example: *All XXX files are available from the XXX database (accession number(s) XXX, XXX.*).<br>• If the data are all contained **within the manuscript and/or Supporting Information files**, enter the following: *All relevant data are within the manuscript and its Supporting Information files.*<br>• If neither of these applies but you are able to provide **details of access elsewhere**, with or without limitations, please do so. For example:<br><br>*Data cannot be shared publicly because of [XXX]. Data are available from the XXX Institutional Data Access / Ethics Committee (contact via XXX) for researchers who meet the criteria for access to confidential data.*<br><br>*The data underlying the results presented in the study are available from (include the name of the third party*	All relevant data are within the manuscript and its Supporting Information files.

and contact information or URL).<br>• This text is appropriate if the data are owned by a third party and authors do not have permission to share the data.<br><br>* typeset	
Additional data availability information:	Tick here if the URLs/accession numbers/DOIs will be available only after acceptance of the manuscript for publication so that we can ensure their inclusion before publication.

Powered by Editorial Manager® and ProduXion Manager® from Aries Systems Corporation

Manuscript-clean-10-27

Click here to access/download;Manuscript;Ropivacaine oily delivery depot-manuscript-clean-10-27.docx

Research article

A randomized trial: the safety, pharmacokinetics and preliminary pharmacodynamics of ropivacaine oily delivery depot in healthy subjects

Wu-dang Lu[1,4], Dan-ling Zhao[2], Mei-xia Wang[3], Ya-qi Jiao[4], Ping Chi[3], Min Zhang[2], Bo Ma[2], Jian-ping Dong[1], Hai-bo Zhang[2], Yi Yang[1], Ye Tian[2], Min-quan Hui[1], Bo Yang[2], Yong-xiao Cao[1*]

1 School of Basic Medical Sciences, Xi’an Jiaotong University Health Science Center, Xi’an, Shaanxi, China,

2 Beijing Aicomer Pharmaceutical Technology Co., Ltd., Beijing, China,

3 Beijing You An Hospital, Capital Medical University, Beijing, China,

4 Xi’an Libang Pharmaceutical Co., Ltd, Xi’an, Shaanxi, China

[*]Corresponding author

Address: 76 Yanta West Road, Xi’an, Shaanxi 710061, P.R. China

Tel/Fax: +86-029-82655140

Email: yxy@xjtu.edu.cn (YXC)

Trial registration

Chictr.org: ChiCTR2200058122;

Chinadrugtrials.org: CTR20192280.

Abstract

Introduction

Ropivacaine oily delivery depot (RODD) can slowly release ropivacaine and block nerves for a long time. The aim of the present work was to investigate the safety, pharmacokinetics, and preliminary pharmacodynamics of RODD in subcutaneous infiltration into healthy subjects.

Methods

The abdomens of 3 subjects were subcutaneously infiltrated with single-needle RODD containing 12~30 mg of ropivacaine. The irritation, nerve blocking range and optimum dose were investigated. Forty-one subjects were divided into RODD containing ropivacaine 150, 230, 300, 350, 400 mg and ropivacaine hydrochloride injection (RHI) 150 mg groups. Multineedle subcutaneous infiltration of RODD or RHI was performed in the abdomens of the subjects. The primary endpoint was safety of a 400 mg maximum dose. Subjects' vital signs were observed, their blood was analyzed, their cardiovascular, nervous systems, and irritation were monitored. Second, the ropivacaine in plasma was determined, pharmacokinetic parameters were calculated, and the anesthetic effects of RODD were studied, including RODD onset time, duration and intensity of nerve block.

Results

RODD 24 mg in a single-needle injected infiltration in 3 subjects was optimal, and the range of nerve block was 42.5±20.8 mm. Multineedle subcutaneous infiltration of RODD in the abdomens of subjects was safe, and all adverse events were no more than

grade II. The incidence rate of grade II adverse events, such as pain and abnormal ST and ST-T segment changes on electrocardiography, was approximately 1%. The incidence rate of grade I adverse events, including erythema, papules, hypertriglyceridemia, and hypotension was more than 10%. Erythema and papules were relieved after 24 h and disappeared after 72 h. Other adverse reactions disappeared after 7 days. The curve of ropivacaine concentration-time in plasma presented a double-peak profile. The results showed that ropivacaine was slowly released from RODD. Compared with the 150 mg RHI group, T_{max} in the RODD groups was obviously longer. In particular, T_{max} in the 400 mg RODD group of 11.8±4.6 h was longer than that in the RHI group of 0.77±0.06 h. The C_{max} in the 150 mg RODD group was obviously lower than that in the 150 mg RHI group (0.35±0.09 vs. 0.58±0.13 μg·mL^{-1}). In particular, the C_{max} increased by 48% when the dose was increased by 2.6 times in the 400 mg group. C_{max} and AUC were positively correlated with doses. Finally, the intensity of nerve block (≥level 2) was correlated with RODD doses. The 400 mg RODD group presented the strongest nerve block. The median of onset time was 0.42 h, and the duration median was 35.7~47.7 h.

Conclusions

RODD containing 400 mg of ropivacaine is safe when subcutaneously infiltrated into healthy subjects. Meanwhile, T_{max} was delayed in the RODD group compared with the RHI group. The median duration of nerve block was more than 35 h. Therefore, RODD has an obvious sustained release effect.

Keywords

ropivacaine oily delivery depot, safety, pharmacodynamics, pharmacokinetics, healthy subject.

Introduction

Ropivacaine is an S-enantiomer amide local anesthetic with high pKa and low lipid solubility. Its nerve block effects are similar those of bupivacaine, but it has lower neurotoxicity, cardiovascular toxicity, and local muscular toxicity [1-4].

At present, ropivacaine hydrochloride injection is widely used in the clinic. Commonly, the injection is infiltrated subcutaneously at the surgical incision or injected directly at the ganglion for preemptive analgesia before operation [5, 6]. During the operation, ropivacaine is combined with opioids for anesthesia maintenance [7, 8]. Moreover, ropivacaine is used in a variety of ways for postoperative analgesia. 1) A mixture of ropivacaine and opioids is administered for persistent nerve block analgesia by an epidural analgesia pump [9]. 2) Ropivacaine solution is infiltrated subcutaneously at the incision, which is suitable for large incision operations with dense cortical nerve distribution, such as in the chest, abdomen and limbs [10-13]. 3) Ropivacaine is sprayed intraperitoneally to reduce pulling pain, and is often used in digestive system surgery, such as stomach, liver, gallbladder, urinary system and gynecological uterus- and ovary-related laparoscopic surgery [14-18]. This postoperative analgesic management can effectively relieve postoperative pain in patients, reduce the amount of prescribed opioids, and promote the postoperative recovery.

After the surgical incision is infiltrated with ropivacaine hydrochloride solution, a nerve block is produced. The time for the first use of other analgesics is less than 8 h, which cannot meet the needs of postoperative analgesia of more than 24 h [19, 20].

Although the increased dose of ropivacaine can prolong the time of nerve block, the increase in plasma drug concentration will significantly increase the incidence of related neurotoxicity and cardiovascular toxicity [21]. Therefore, there is obvious clinical significance in prolonging the postoperative nerve block time of ropivacaine and reducing adverse reactions.

To date, slow-release formulations of ropivacaine have been studied. Ropivacaine liposome gels containing infrared photosensitizers and phospholipids could be retained *in vivo* for 10 days [22]. Poloxamer 407 was used as an excipient to prepare a ropivacaine loaded gel to treat pain after thoracoscopic pneumonectomy. The analgesic effect was comparable to that of continuous infusion of 0.2% ropivacaine for 48 h [23]. Poloxamer 407 and polycaprolactone electrospinning fibers were used as excipients to prepare ropivacaine hydrogels that blocked rat nerves for more than 30 h [24]. However, the evidence of human safety of new excipients is insufficient, and liposomes or hydrogels have the risk of sudden release. Oil solutions commonly used in the clinic can avoid the above problems.

Ropivacaine is insoluble in water and is slowly released into the oil solution. Castor oil and phospholipids were used as carriers to design a ropivacaine suspension formula that could sustainably block nerves for 36 h [25]. We disclosed the dogs' pharmacokinetic data and preliminary toxicity of ropivacaine in soybean oil solution. Compared with ropivacaine hydrochloride injection, the C_{max} value of the ropivacaine oily delivery system was decreased by 80%, and T_{max} was significantly delayed. The dog was given a subcutaneous preparation of 60 $mg \cdot kg^{-1}$. There was no

significant change in the dog's behaviors, blood count, coagulation, blood, biochemistry urine, etc. The oily delivery system of ropivacaine was demonstrated to be safe [26].

Currently, our formula of ropivacaine oily delivery depot (RODD) has been approved by the Center for Durg Evaluation of National Medical Products Administration of China to carry out phase I clinical research. This study simulated the administration method of local infiltration analgesia after abdominal surgery [27], and the safety, pharmacokinetics and preliminary efficacy of RODD in healthy subjects were studied.

Materials and methods

Materials

Ropivacaine was obtained from Libang Pharmaceutical Co., Ltd., China. The purity was 99%. The batch number was C082-190101. Benzyl alcohol and benzyl benzoate were purchased from Hunan Er-kang Pharmaceutical Co., Ltd., China. The batch numbers were 104020190402 and F241C180902, respectively. Soybean oil was produced in Tieling Pharmaceutical Co., Ltd., China. The batch number was 19060203-2-01. Ropivacaine hydrochloride injection (RHI) was manufactured by AstraZeneca AB, Sweden. The specifications are 100 mg:10 mL. The batch number was NBAZ. RODD was composed of 2% ropivacaine, 4% benzyl alcohol, 30% benzyl benzoate and approximately 64% soybean oil, and was commissioned to be produced at the GMP (Good Manufacturing Practice)-certified plant of Jiangsu Taikang Biomedical Co., Ltd, batch number CR20191001. Ropivacaine-D7, a reference substance, was obtained from

USP

RODD preparation

Ropivacaine (200 mg) was added to benzyl alcohol. After the ropivacaine was dissolved, benzyl benzoate and soybean oil were added to the ropivacaine solution up to 10 mL. Then, the mixture was heated and stirred until the solution was clear. After being filtered and sterilized with a filter, the bulk solution, i.e., RODD, was obtained, divided into vials, filled with nitrogen and sealed. The concentration of RODD was 20 $mg \cdot mL^{-1}$, and the stability of RODD was more than 2 years at 2~8°C.

Selection of subjects

The inclusion criteria included an age range of healthy subjects of 18~50 years for men or women, and a body mass index ranging from 19.0 to 26.0 $kg \cdot m^{-2}$.

Exclusion criteria included subjects with abnormal vital signs, clinical/laboratory tests (blood routine, urine routine and blood biochemical, serology, electrocardiogram (ECG)), cerebrovascular, liver, kidney, respiratory, blood, endocrine, immune, mental diseases, gastrointestinal system; porphyria patients, malnutrition or blood disease, scar constitution; 2 weeks taking drugs, in particular, analgesics; long-term drinking of tea and coffee, blood donation within three months, and participated in other drug clinical tests within three months.

A total of 50 healthy subjects were selected, with no restriction on men and women. They were informed about the purpose, procedure, possible benefits and risks of the study and signed the informed consent form.

Ethics statement

The research was designed and conducted in accordance with the current “Good Clinical Practice (GCP) of China”, “The International Council for Harmonization of Technical Requirements for Pharmaceuticals for Human Use (ICH)-GCP”, “Guidelines for Ethical Review of Drug Clinical Trials” and “Declaration of Helsinki”. Clinical trial protocol revision and implementation were approved and supervised by the Hospital Ethics Committee. For the first time, the protocol of the clinical study was approved by the Ethics Committee of Beijing You’an Hospital affiliated with Capital Medical University on September 5, 2019. The study recruited the first subject on December 2, 2019, and enrolled the last participant on June 25, 2021.

The study has completed registration in the drug clinical trial registration and information disclosure platform, which is the legal registration platform for clinical information used by the drug evaluation center of the state medical products administration of China. The clinical trial record number is CTR20192280 in China. At present, the study has completed registration in Chinese Clinical Trial Registry (ChiCTR), and the clinical trial record number is ChiCTR2200058122.

Trial design

Three subjects were single-needle infiltrated subcutaneously with different doses of RODD in the abdomen to explore the optimal dose, anesthesia range, and irritation. The abdomens of forty-one subjects had multiple needles infiltrated subcutaneously with an escalating dose of RODD. The primary endpoint was safety, defined as the occurrence of grade III adverse events, or the attainment of RODD’s maximum (containing 400 mg of ropivacaine). Meanwhile, the pharmacokinetics and preliminary effects were

studied. The grouping and test process is shown in Fig 1.

Fig 1. Grouping and flow chart of healthy subjects' abdominal subcutaneous infiltration RODD.

Study 1: Single-needle subcutaneous infiltration exploration

Three subjects were selected. Fig 2A shows that the abdomen of each subject was divided into six regions from the ensisternum and marked I to VI clockwise. According to the random number table from the computer, any area was selected as the starting point and marked 1~5 clockwise. The infiltration doses of RODD (containing ropivacaine) were 12 mg (0.6 mL), 16 mg (0.8 mL), 20 mg (1.0 mL), 24 mg (1.2 mL) and 30 mg (1.5 mL). Area 6 was used as the blank control. An interval of 1 h between two adjacent doses was used to observe the adverse reactions. This experiment was based on the VAS score standard [28]. The range of nerve block was measured through acupuncture by a Von Frey electronic pain meter (IITC Life Science) before and 0.25, 0.5, 0.75, 1, 1.5, 2, 3, 4, 5, 6, 7, 8, 9, 10, 11, and 12 h after subcutaneous administration.

The test methods are shown in Fig 2B and C. The infiltration points in the abdominal region were taken as the starting point, eight test directions were designed, and two lines (r1 and r2) were randomly selected to test the nerve block effect in 8 directions around the infiltration point that started from the infiltration point. Test points were set along the line from the infiltration point, and the distance between two points was 1 cm. The test steps are shown in Fig 3. The nerve block range (r1+r2) at each

infiltration point was recorded and the area under the block range-time curve was calculated to determine the optimal infiltration dose.

At 0.5 h before administration, and at 0.3, 0.6, 1, 2, 6, 24, and 72 h after administration, the symptoms of infiltration sites were recorded, and the irritation was scored.

Fig 2. Schematic diagram of healthy subjects' abdominal subcutaneous infiltration of RODD. A and D are the distribution designs of single-needle and multineedle infiltration areas under the abdomen skin, which were areas Ⅰ~Ⅵ and Ⅰ~Ⅳ, respectively. B and C are examples of single-needle infiltration points and needle test points; E and F are examples of multineedle infiltration points and acupuncture test points on a circle, respectively.

Fig 3. The evaluative process of analgesia range in single-needle abdominal subcutaneous infiltration with RODD in healthy subjects.

Study 2: Multiple-needle subcutaneous infiltration

The subjects were randomly divided into 150, 230, 300, 350 and 400 mg RODD groups (n=6~8), and the ropivacaine hydrochloride injection (RHI) 150 mg group (n=3), and each subject was subcutaneously infiltrated with RODD or RHI. The abdomen was divided into four areas by drawing horizontal and vertical lines through the navel, as shown in Fig 2D, marked with Ⅰ, Ⅱ, Ⅲ and Ⅳ clockwise.

One area was randomly selected and marked as 1, while the other areas were marked clockwise as 2~4. Areas 1~3 were infiltrated RODD, and area 4 was the blank control for the nerve block test. A point in each region was selected as the center of the circle (the distance between the two centers was more than 8 cm), and the optimal block range and dosage were obtained in Study 1. The number of needles in each dose group was calculated as follows:

Number of needles in the group = total dose of the group/optimal dose.

Number of needles on circle 1 or 2 = the number of needles on each group/3.

The remaining needles were allocated to the third circle. The needles on the circumference should be evenly distributed. The intensity of the nerve block in the abdominal infiltrating area was measured in all subjects at different times after administration. The subjects were hospitalized for 72 h and followed up for 7 days after discharge.

As shown in Fig 2E and F, two infiltration points on a circle were randomly selected, which were connected to the center of the circle and extended outward 2 cm, respectively. The four test points on the straight line are the center of the circle, 1 cm away from the center of the circle (inside the circle), and 1 and 2 cm away from the infiltration point (outside the circle). The test area was within 0.5 cm around the test point. Referring to the VAS method, pain intensity was tested by acupuncture in any two directions of circles 1 and 2 [28], and each point or area was tested once. The evaluative process of nerve block intensity is shown in Fig. 4.

Fig 4. Scoring process of analgesia intensity for subcutaneous multile-needle infiltration of RODD in the abdomens of healthy subjects in Study 2.

Safety assessment

Vital signs such as blood pressure, pulse, respiration and oxygen saturation were measured in the supine position for the subjects 1 h before and 1, 2, 4, 8, 12, 24, 48 and 72 h after administration. Routine blood tests, electrocardiograms, blood biochemistry and urine tests were performed 24 h before and 72 h after administration. The symptoms of infiltration sites were recorded, and irritation was scored at 0.5 h before and 0.3, 0.6, 1, 2, 6, 24 and 72 h after administration. The criteria for local adverse events are shown in Table 1. The safety evaluation was based on the Evaluation Criteria for “Common Adverse Events” published by Chinese Clinical regulatory authorities (V5.0, 2017.11). Routine blood tests included red blood cell count, white blood cell count, hemoglobin, hematocrit, platelet count, eosinophil count, neutrophil count, and lymph cell count, monocyte count, etc. Routine urine examination items included glucose, ketone bodies, protein, urinary occult blood, white blood cells, bilirubin, nitrite, specific gravity, etc. Blood biochemical indices included potassium, sodium, chlorine, calcium, magnesium, phosphorus, blood glucose, creatinine, urea, glutamine transferase, uric acid, lactate dehydrogenase, total bilirubin, aspartic ammonia acid aminotransferase, alanine aminotransferase, alkaline phosphatase, triglyceride, total cholesterol, total protein, albumin and so on.

Pharmacokinetics

The liquid chromatography coupled with tandem mass spectrometry (LC–MS/MS) analysic method of ropivacaine was optimized on the basis of previous reports [29-31]. The liquid chromatography analysis conditions are shown in Table 2, including a column temperature of 20°C, injection volume of 10 μL, flow rate of 0.40 $mL \cdot min^{-1}$, and sample chamber temperature of 2~8°C. Mass spectrometry conditions included API 4000 mass spectrometer, Turbo Ionspray ($APCI^+$) ion source, MRM detection mode, GS1 and 2 of 60 psi, TEM: 500°C, CUR: 30 psi, CAD: 5 psi, ropivacaine ion pair: 275.1/126.1, scanning time 200 ms, DP: 60 V, EP: 10 V, CE: 24 V; CXP: 13 V. The extraction and detection method were verified, and the content of ropivacaine in plasma samples was determined.

At 0.25 h before and 0.5, 1, 1.5, 2, 4, 6, 8, 10, 12, 14, 21, 24, 27, 30, 33, 36, 48 and 72 h after administration, 4 mL of venous blood was drawn into a blood collection tube containing EDTA-K_2 anticoagulant, and centrifuged at 2000 × g for 10 min. The upper layer of plasma was transferred into 2 mL cryopreserved tubes (Axygen, made in USA) and then stored in a refrigerator (-60~-90°C). The plasma was treated with the protein precipitation method before analysis. Specifically, the plasma was thawed at room temperature and blended by vortexing, 50 μL of plasma was transferred into a centrifuge tube, and 160 μL of ropivacaine-D7 acetonitrile solution (internal standard ropivacaine-D7 15 $ng \cdot mL^{-1}$) was added. After 1 min of vortexing, the sample was centrifuged at 12000 rpm for 10 min, and 40 μL supernatant was transferred into the sample tube. Then, 160 μL ultra-pure water was added to the same tube and vortexed for testing. The injection volume was 10 μL for quantitative analysis by LC–MS/MS.

Preliminary pharmacodynamics

At 0.25 h before and 0.25, 1, 4, 8, 10, 12, 14, 18, 21, 24, 27, 30, 33, 36, 48 and 72 h after administration, the analgesia intensity was measured by acupuncture on circles 1 and 2 of the abdomen, and the onset time and effective duration were evaluated. The distance between two adjacent infiltration points was recorded.

Statistical an[illegible]ysis

All data were analyzed by SAS software (version 9.4), and the indicators of each group were compared with baseline. The ropivacaine concentration-time curves were drawn, and pharmacokinetic parameters were calculated (Phoenix Win Nonlin 8.2). Local irritation scores of RODD were evaluated by descriptive statistics, and the frequency and percentage of categorical or ordinal variables were summarized. The mixed effect model was used to analyze the changes in acupuncture pain intensity at different time points before and after administration. In the model, changes in pain intensity at different time points in different administration areas compared with baseline were dependent variables. Pain intensity before administration was a cov[illegible]ble. Administrative area/blank area, grouping and visit point were fixed effects. The onset time and duration of RODD nerve block were compared by median (95%, CI). The analgesic intensity was calculated by percentage, and the range of nerve block and distance adjacent infiltration points were expressed as the mean±SD.

Results

Subject disposition and demographics

A total of 44 healthy subjects, 75% men and 25% women, with an average age of 29.3±7.1 years, were enrolled. Han nationality accounted for 93% and other ethnic groups accounted for 7%. The average height was 168.6±8.4 cm, the average weight was 63.9±9.7 kg, and the average body mass index was 22.3±1.9 kg·m^{-2}. The specific demographic data of healthy subjects are shown in Table 3. In addition, whole blood and blank plasma samples from 6 healthy subjects were collected for the methodology and stability study of the required biological samples.

Optimal dose of single-needle infiltration RODD

After single-needle subcutaneous infiltration of RODD, the block range-time curve is shown in Fig 5A. In the 24 mg and 30 mg RODD groups, the nerve block range (i.e., average diameter, 42.5±20.8 and 46.2±24.3 mm) and the areas under the curve (574±228 and 573±56 h·mm) were significantly higher than those in the 12, 16 and 20 mg groups (Table 4). The anesthesia duration was maintained for more than 12 h. Slight erythema around the injection site was alleviated after 2 h, with an incidence of 28%, and disappeared after approximately 24 h (Fig 5B). The local irritation of RODD at the 24 mg infiltration points was slightly lower than that at the 30 mg infiltration point. It was suggested that the optimal dosage was 24 mg with a block range of 40 mm.

Fig 5. Curves of nerveblock range-time after subcutaneous infiltration 12~30 mg RODD (mean±SD, A). Irritation after subcutaneous infiltration RODD for 2 h on the abdomen of the subject (B), and slight erythema appeared on the skin around some infiltration points (arrows).

Safety of multiple-needle infiltration RODD

The abdomen of the subjects was divided into 4 regions, and a circle with a diameter of 40 mm was marked in every region. The number of infiltration points and drug quantity distribution on the circle are shown in Table 5. The incidences of adverse reactions of subjects in the 150, 230, 300, 350, 400 mg RODD, and 150 mg RHI groups were 83.3% (5/6), 75.0% (6/8), 100.0% (8/8), 62.5% (5/8), 87.5% (7/8) and 100.0% (3/3), respectively. Common grade I adverse events included erythema at the infiltrating site (40.9%, 18/41), papules (15.9%, 7/41), hypertriglyceridemia (25.0%, 11/41), andhypotension (20.4%, 9/41). There was no correlation between the incidence of adverse events and doses. Erythema and papules are shown in Fig 6. Grade II adverse events mainly included ST segment depression and ST segment abnormality (2.3%, 1/41). There were no grade III or above adverse events (Table 6). Most of the erythema and papules in the infiltrating site were relieved 24 h after administration and almost disappeared within 72 h. Blood pressure and ECG returned to normal 24 h after administration.The data suggested that RODD has good safety.

Fig 6. Local skin irritation (arrows) of healthy subjects in Study 2 at 2 h after subcutaneous infiltration RODD. A shows mild erythema around the infiltration point in the 150 mg RODD group. B shows erythema and mild papules around the infiltration site in the 300 mg RODD group, and C shows no abnormalities around the infiltration site in 400 mg RODD group.

Methodological verification

The analysis method of ropivacaine had superior selectivity. The standard curve range was 2.5~2000 ng·mL^{-1}, the correlation coefficient was greater than 0.998, the inter batch precision (CV%) was 0.81%~2.05%, the inter batch accuracy (RE%) was -2.45%~2.06%, and the lowest limit of quantification was 2.5 ng·mL^{-1}. Quality control samples were prepared by adding different doses of ropivacaine to blank plasma, and then the samples were processed according to the sample handling method. The recovery rates of the quality control samples (the quality control of low (7.5 ng·mL^{-1}), medium (150 ng·mL^{-1}) and high (1.5 mg·mL^{-1}) concentrations) were 102.1%±4.5%, 102.8%±1.5% and 103.1%±1.9%, respectively. The average recovery was 102.7%±0.5%. The internal standard recovery was 97.9%±2.2%, and the matrix effects were 99.3%±2.8%, 100.6%±3.2% and 99.3%±2.0%, respectively. The average matrix effect was 99.7%±0.8%. The internal standard matrix effect was 99.3%±3.2%, without hemolysis or hyperlipidemia effects. The in-batch precision (CV %) and accuracy (RE %) of the quality control samples were 1.15%~4.99% and -2.45%~6.93%, respectively, the interbatch precision (CV %) was 2.87%~3.56%, and the interbatch accuracy (RE%) was 1.32%~4.42%. The quality control sample presented good stability at -20°C and -70°C for 2 years, -70°C for repeated freeze- thaw cycles, room temperature and 2~8°C for 3 days, which met the determination requirements.

Pharmacokinetics

The average plasma concentration-time curve of ropivacaine in each group is presented

in Fig 7. The ropivacaine concentration in the RHI group peaked at 0.5 h after administration and then decreased rapidly, suggesting that the release of ropivacaine was rapid. The ropivacaine concentration-time curves in the RODD groups presented double peaks, with the first peak at 1~2 h and the second peak at 10~15 h. After the second peak, the concentration of ropivacaine decreased slowly. The pharmacokinetic parameters are shown in Table 7. Compared with the C_{max} in the RHI group (0.58±0.12 μg·mL^{-1}), the C_{max} of the 150 mg RODD group (0.35±0.09 μg·mL^{-1}) was significantly decreased. The C_{max} values of the 230 and 300 mg RODD groups increased gradually. Moreover, the C_{max} values of the 350 and 400 mg groups were 0.80±0.12 and 0.86±0.24 μg·mL^{-1}, respectively, and the increases were significant. The T_{max} value of the RHI group was 0.77±0.06 h, and the T_{max} of the RODD groups ranged from 3.63±4.98 h to 11.81±4.58 h. The result of average concentration- time semilog of ropivacaine in each group were suggested that ropivacaine was continuously released from the RODD into the blood before the second peak, which offset part of ropivacaine's metabolism. Ropivacaine elimination exhibited linear pharmacokinetic characteristics after complete release, namely, the first-order model. This was the same metabolic pattern as that reported for ropivacaine hydrochloride injection [32].

Fig 7. After multineedle subcutaneous infiltration of RODD in healthy subjects' abdomens, curves of ropivacaine concentration-time in plasma, mean±SD. The time ranges of A and B were 0~ 4 h and 0~72 h, respectively.

Onset, duration, and intensity of nerve block

The onset time of nerve block shortened with increasing RODD dose, and the time of the 400 mg group was the fastest, with a median of 0.42 h in all test areas. The duration of the nerve block was dose-independent, and the median of the 400 mg RODD group was 35.7~47.7 h. When the scores of nerve block intensity were greater than 4 (>level 1), the analgesic rate of 150~400 mg RODD groups were 100%. In the scores of nerve block intensity were greater than and equal to 5 (≥level 2) the analgesic rate of 150, 230, 300, 350, and 400 mg RODD groups were 13.9%, 22.5%, 32.8%, 30.8%, 42.9%, respectively, and that was positively correlated with the dose of RODD. Finally, in the scores of nerve block intensity were greater than and equal to 6 (level 3), the analgesic rate of the RODD groups ranged from 0.99% to 3.6%, a small percentage with no significant dose correlation. Therefore, the highest nerve block intensity was found in the 400 mg RODD group, and the distance between two infiltration points on the circumference was 21.5±1.5 mm (Table 8).

Discussion

An injectable oily delivery depot (ODD) is a classic oily drug delivery system, that is a vehicle for hormone or psychotropic drugs. Currently, there have been clinical studies on the application of ODD in the field of anesthesia such as the formulation of ropivacaine with castor oil and phospholipids. However, the high irritant and sensitization of castor oil arecauses for concern [33, 34]. In contrast, the RODD formulation that we designed for clinical trials, with high concentrations of ropivacaine

has a stronger nerve block analgesic effect. Benzyl alcohol is a rapid analgesic for common injections. Benzyl benzoate has an anti-inflammatory effect, and soybean oil is the main excipient for lipid emulsion for injection [35-37]. Therefore, RODD was judged to have better clinical safety and anesthetic effects.

RODD was allowed for further clinical studies in healthy subjects after evaluation in animal studies. In exploratory studies, there was slight local irritation at a single infiltration dose of RODD 24 mg. Local irritation slightly increased after multineedle subcutaneous administration. In most cases, it was relieved after 24 h and disappeared after 72 h. It was speculated that the local irritation might be related to the overlapping infiltration range of two adjacent needles. The incidence of RODD-related adverse events was mild, including hypotension, abnormal ST-segment electrocardiogram, and hypertriglyceridemia, which returned to normal 72 h after administration. In addition, two subjects in the RHI group had mild erythema in the infiltrated area, and one subject had hypertriglyceridemia, which quickly disappeared. These transient and mild adverse reactions may be caused by ropivacaine, excipients, frequent clinical administration, or topical disinfectants. In addition, no abnormal mental reaction was observed. Therefore, RODD was demonstrated to be safe. The results support the follow-up study of subcutaneous infiltration analgesia in patients.

The preliminary pharmacodynamic assessment of RODD was divided into two parts. The recommended amount of RODD in single-needle administration was 24 mg in the exploratory experiment, the duration of analgesia was approximately 12 h, and the infiltration range was approximately 40 mm. The circle on the abdomen of the

subject was uniformly infiltrated with 6 needles in the 400 mg RODD group, the distance between two adjacent needles was 20 mm, and the subcutaneous infiltration range overlapped each other, suggesting that the anesthesia time of RODD multipoint infiltration was further prolonged. The efficacy of local infiltration of RHI has been reported [38-40]. The analgesic onset time and duration of RHI were approximately 10~45 min and 6~8 h, respectively. Compared with RHI, the analgesic onset time of the 400 mg RODD group was similar, and the duration of analgesia was extended by more than 6 times (vs. 35.7~47.7 h, median), which could meet the needs of postoperative analgesia. Therefore, the analgesic effect of RODD was significantly prolonged, and the potential clinical advantages were obvious.

The concentration-time curves of ropivacaine for RODD were significantly different from those for RHI. The RHI curve rose rapidly after administration, and the curve decreased rapidly after 0.77 h (T_{max}) and was then prolonged at a lower concentration. The first peak (589.4+158.9 ng·mL^{-1}) of the curve in the RODD 400 mg group was reached at 1 h after administration, and the second peak (864.9+237.5 ng·mL^{-1}) was reached at 11.8 h (T_{max}). Then, the curve decreased slowly. The curves of the 150~350 mg RODD group were similar to that of the 400 mg RODD group. This phenomenon has also been found in preclinical animal studies of RODD, including pharmacokinetics in dogs and toxicokinetics in rats [26, 41]. This bimodal phenomenon had not been found with other clinically used oily preparations. The metabolic pathway of ropivacaine in the body is clear; and ropivacaine has no hepato-enteric ciculation [32]. Therefore, we hypothesized that the bimodal ropivacaine-time curve characteristic

of RODD in subcutaneous infiltration was caused by the continuous release of ropivacaine, which might be related to the combinative effect of free ropivacaine and ODD. The ropivacaine outside of RODD releases to produce the first peak. With the metabolism of ODD, then, ropivacaine inside of RODD re-releases to generate the second peak.

Pharmacokinetic studies of RODD showed that the T_{max} of the RHI 150 mg group was lower than 1 h, while that of the RODD 150~400 mg groups lagged 3.6~11.8 h, suggesting that ropivacaine can be released slowly. Compared with the 150 mg RHI group, the C_{max} value in the RODD 150 mg group decreased by 40%, and the value increased by 27%, and 48% when the doses were increased to 350 mg and 400 mg, respectively. This value is much lower than the reported C_{max} of ropivacaine hydrochloride injection at the maximum tolerated dose (2.9±0.5μg·mL^{-1}) [42]. All of the above findings suggest that RODD is safe. Second, the elimination half-life ($t_{1/2}$) of the 150 mg RHI group was 7.6±5.4 h, which was higher than that of the 150 mg RODD group. The reason might be the small number of subjects in the RHI group, or the difference in ropivacaine concentration between RODD and RHI.

At present, the release mechanism in ODD has been further studied. For instance, Kalicharan et al. reported the metabolism of ODD in different tissues, and measured its spatial distribution by magnetic resonance imaging technology to visualize the changes of in ODD *in vivo*. They found that 1 mL ODD was injected into the vastus lateralis and deltoid muscles in healthy subjects, and the ODD could be cleared from the body within a week [43, 44]. This provided support for us to further study the release mode

of ropivacaine from RODD *in vivo*. The mechanism of RODD is still being investigated, and we will report it in the future.

Conclusions

The results of clinical studies showed that RODD at no more than 400 mg shows good safety with slight irritation. RODD can slowly release ropivacaine to produce long-duration analgesia for more than 35 h, which can be used for long-term analgesia in the clinic.

Supporting information

S1 Checklist. CONSORT 2010 checklist.

S2 File. Study protocol (Brief english version).

S3 File. Study protocol (Original chinese version).

Acknowledgments

The authors are grateful to all the subjects who took part in the trial, as well as the team of doctors' team in the Department of Anesthesiology and the Phase I Clinical Research Institution at You An Hospital, Capital Medical University.

Author Contributions

Conceptualization: Wu-dang Lu, Yong-xiao Cao.

Formal analysis: Wu-dang Lu, Mei-xia Wang, Bo Yang.

Investigation: Wu-dang Lu, Dan-ling Zhao, Mei-xia Wang, Ya-qi Jiao, Min Zhang, Hai-bo Zhang, Jian-ping Dong, Yi Yang, Ye Tian, Min-quan Hui, Bo Yang.

Validation: Hai-bo Zhang, Ye Tian.

Methodology: Dan-ling Zhao, Ya-qi Jiao, Ping Chi, Bo Ma, Yong-xiao Cao.

Resources: Dan-ling Zhao, Mei-xia Wang, Ping Chi, Min Zhang, Yi Yang.

Project administration: Wu-dang Lu, Dan-ling Zhao, Mei-xia Wang, Ya-qi Jiao, Ping Chi, Bo Ma, Jian-ping Dong, Min Zhang, Hai-bo Zhang, Yong-xiao Cao.

Writing–original draft: Wu-dang Lu.

Writing–review & editing: Wu-dang Lu, Yi Yang, Yong-xiao Cao.

References

1. Mcclellan KJ, Faulds D. Ropivacaine: an update of its use in regional anaesthesia. Drugs. 2000; 60(5): 1065-93. https://doi.org/10.2165/00003495-200060050-00007 PMID: 11129123
2. Knudsen K, Beckman SM, Blomberg S, Sjövall J, Edvardsson N. Central nervous and cardiovascular effects of i.v. infusions of ropivacaine, bupivacaine and placebo in volunteers. Br J Anaesth. 1997; 78(5): 507-14. https://doi.org/10.1093/bja/78.5.507 PMID: 9175963
3. Yutaka ODA. Ropivacaine: pharmacokinetics and toxicity. The journal of Japan Society for Clinical Anesthesia. 2009; 29(4): 519-27. https://doi.org/10.2199/ jjsca.29.519.
4. Viderman D, Ben-David B, Sarria-Santamera A. Analysis of bupivacaine and ropivacaine-related cardiac arrests in regional anesthesia: a systematic review of

case reports. Rev Esp Anestesiol Reanim. 2021; 68(8): 472-83. https://doi.org/10.1016/ j.redare.2020.10.005 PMID: 34538765

5. Zhou HY, Ou MC, Yang YX, Ruan Y, Pan Y, Li Y. Effect of skin infiltration with ropivacaine on postoperative pain in patients undergoing craniotomy. Springerplus. 2016; 5(1): 1180. https://doi.org/10.1186/s40064-016-2856-3 PMID: 27512639
6. Kang KH, Kim BS, Kang H. The benefits of preincision ropivacaine infiltration for reducing postoperative pain after robotic bilateral axillo-breast approach thyroidectomy: a prospective, randomized, double-blind, placebo-controlled study. Ann Surg Treat Res. 2015; 88(4): 193-9. https://doi.org/10.4174/astr.2015.88.4.193 PMID: 25844353
7. Sun XF, Zhou Q, Zhou M, Cao R, Chen ZL, Tang SJ, et al. The effect of epidural nalbuphine combined with ropivacaine on epidural analgesia during labor: a multicenter, randomized, double-blind, controlled study. The Clinical Journal of Pain. 2021; 37(6): 437-42. https://doi.org/10.1097/AJP.0000000000000928 PMID: 33758132
8. Zhou SQ, Wang J, Du WJ, Song YJ, Xu ZD, Liu ZQ. Optimum interval time of programmed intermittent epidural bolus of ropivacaine 0.08 with sufentanyl 0.3 μg/mL for labor analgesia: a biased-coin up-and-down sequential allocation trial. Chin Med J. 2020; 133(05): 517-22. https://doi.org/10.1097/CM9.0000000000000669 PMID: 32142491
9. Shelke PD, Nale SR, Babriya J, Tiwari S. A comparative study of epidural ropivacaine 0.2% with nalbuphine and ropivacaine 0.2% with fentanyl in unilateral total knee replacement surgeries. Int J Med Anesthesiology. 2020; 3(3): 14-7. https://doi.org/10.33545/26643766.2020.v3.i3a.140.
10. Mattila I, Pätilä T, Rautiainen P, Korpela R, Nikander S, Puntila J, et al. The effect of continuous wound infusion of ropivacaine on postoperative pain after median sternotomy and mediastinal drain in children. Paediatr Anaesth. 2016; 26(7): 727-33. https://doi.org/10.1111/ pan.12919 PMID: 27184591
11. Choromanski DW, Patel PS, Frederick JM, Lemos SE, Chidiac EJ. The effect of continuous interscalene brachial plexus block with 0.125% bupivacaine vs 0.2%

ropivacaine on pain relief, diaphragmatic motility, and ventilatory function. J Clin Anesth. 2015; 27(8): 619-26. https://doi.org/10.1016/j.jclinane.2015.03.006 PMID: 26216250

12. Zhang Y, Liu L, Zheng L, Chen J, Huang L, Wang Q, et al. Comparison of effectiveness of ropivacaine infusion regimens for continuous femoral nerve block for recovery after total knee arthroplasty: a randomized double-blind trial. J Pain Res. 2020; 13: 997-1005. https://doi.org/10.2147/JPR.S247158 PMID: 32494188
13. Li BL, Liu XZ, Cui LH, Zhang WQ, Pang H, Wang MS, et al. Local infiltration analgesia with ropivacaine improves postoperative pain control in ankle fracture patients: a retrospective cohort study. Pain Research and Management. 2020 (Special issue): 1-7. https://doi.org/10.1155/2020/8542849 PMID: 32215137
14. Collins GG, Gadzinski JA, Fitzgerald GD, Sheran J, Wagner S, Edelstein S, et al. Surgical pain control with ropivacaine by atomized delivery (spray): a randomized controlled trial. J Minim Invasive Gynecol. 2016; 23(1): 40-5. https://doi.org/10.1016/j.jmig.2015.07.018 PMID: 26241686
15. Choi SW, Cho SJ, Moon HW, Lee KW, Lee SH, Hong SH, et al. Effect of intercostal nerve block and nephrostomy tract infiltration with ropivacaine on postoperative pain control after tubeless percutaneous nephrolithotomy: a prospective, randomized, and case-controlled trial. Urology. 2018; 114: 49-55. https://doi.org/10.1016/j.urology.2017.12.004 PMID: 29288788
16. Samal D, Sahoo R, Patra K. Multisite ropivacaine for pain relief after laparoscopic cholecystectomy: a placebo controlled study at a tertiary care teaching hospital. Int Surg J. 2018; 5(1): 115-9. http://dx.doi.org/10.18203/2349- 2902.isj20175550
17. Alevizos L, Zavridis P, Kalles V, Albanopoulos K, Menenakos E, Zografos G, et al. Efficacy of incisional infiltration and intraperitoneal instillation of ropivacaine for the management of pain after laparoscopic sleeve gastrectomy: A randomised clinical trial. Eur J Anaesthesiol. 2020; 37(9): 829-33. https://doi.org/10.1097/EJA.0000000000001206 PMID: 32769515
18. Liu DS, Guan F, Wang B, Zhang T. Combined usage with intraperitoneal and incisional ropivacaine reduces pain severity after laparoscopic cholecystectomy.

Int J Clin Exp Med. 2015; 8(12): 22460-8 PMID: 26885228

19. Weinbroum AA. Postoperative hyperalgesia-A clinically applicable narrative review. Pharmacol Res. 2017; 120: 188-205. https://doi.org/10.1016/j.phrs.2017.02.012 PMID: 28365208
20. Sommer M, de Rijke JM, van Kleef M, Kessels AG, Peters ML, Geurts JW, et al. The prevalence of postoperative pain in a sample of 1490 surgical inpatients. Eur J Anaesthesiol. 2008; 25(4): 267-74. https://doi.org/10.1017/S0265021507003031 PMID: 18053314
21. Winkler M, Alt A, Dietzsch S, Kunz SN. Two fatal and four surviving cases after accidental infusion of ropivacaine. Forensic Toxicol. 2021; 39, 506–12. https://doi. org/10.1007/s11419-021-00574-x.
22. Hou Y, Meng X, Zhang S, Sun F, Liu W. Near-infrared triggered ropivacaine liposomal gel for adjustable and prolonged local anaesthesia. Int J Pharm. 2022; 611: 121315. https://doi.org/10.1016/j.ijpharm.2021.121315 PMID: 34826592
23. Jeon JH, Seong YW, Han JE, Cho S, Kim JH, Jheon S, et al. Randomized trial of poloxamer 407-based ropivacaine hydrogel after thoracoscopic pulmonary resection. Ann Thorac Surg. 2021; 114(4): 1189-96. https://doi.org/10.1016/j.athoracsur PMID: 34653384.
24. Chen S, Yao W, Wang H, Wang T, Xiao X, Sun G, Yang J, Guan Y, Zhang Z, Xia Z, Li M, Tao Y, Hei Z. Injectable electrospun fiber-hydrogel composite sequentially releasing clonidine and ropivacaine for prolonged and walking regional analgesia. Theranostics. 2022; 12(11): 4904-21. https://doi.org/10.7150/thno.74845 PMID: 35836801
25. Li TF, Fan H, Wang YX. Epidural sustained release ropivacaine prolongs anti-allodynia and anti-hyperalgesia in developing and established neuropathic pain. Plos One. 2015; 10(1): e0117321. https://doi.org/10.1371/journal.pone. 0117321 PMID: 25617901
26. Hui MQ, Lu WD, Yu WP, Chen T, Gu JL, Cao YX. Pharmacokinetics and toxicity of oily delivery system of ropivacaine via subcutaneous injection in beagle dogs. J. Pharmacol. Toxicol. 2018; 13(1): 37-44. https://doi.org/10.3923/ jpt.2018.37.44.

27. Das NT, Deshpande C. Effects of intraperitoneal local anaesthetics bupivacaine and ropivacaine versus placebo on postoperative pain after laparoscopic cholecystectomy: a randomised double blind study. J Clin Diagn Res. 2017; 11(7): UC08-UC12. https://doi.org/10.7860/JCDR/2017/26162.10188 PMID: 28893013
28. Karcioglu O, Topacoglu H, Dikme O, Dikme O. A systematic review of the pain scales in adults: Which to use? Am J Emerg Med. 2018; 36(4): 707-14. https://doi.org/10.1016/j.ajem.2018.01.008 PMID: 29321111
29. Lamy E, Fall F, Boigne L, Gromov K, Fabresse N, Grassin-Delyle S. Validation according to european and american regulatory agencies guidelines of an LC-MS/MS method for the quantification of free and total ropivacaine in human plasma. Clin Chem Lab Med. 2020; 58(5): 701-8. https://doi.org/10.1515/cclm-2018-1298 PMID: 30939109
30. Breindahl T, Simonsen O, Andreasen K. Column-switching HPLC-MS/MS analysis of ropivacaine in serum, ultrafiltrate and drainage blood for validating the safety of blood reinfusion. J Chromatogr B Analyt Technol Biomed Life Sci. 2010; 878(1): 76-82. https://doi.org/10.1016/j.jchromb.2009.11.028 PMID: 1995 5023
31. Zhou JZ, Chen ML, Cheng XH, Li Y, Mei XG. Determination of ropivacaine in rat plasma with liquid chromatography-tandem mass spectrometry. Journal of International Pharmaceutical Research. 2016; 43(4): 736-9. https://doi.org/10. 13220/j. cnki. jipr.2016.04.028.
32. Hansen TG. Ropivacaine: a pharmacological review. Expert Rev Neurother. 2004; 4(5): 781-91. https://doi.org/10.1586/14737175.4.5.781 PMID: 15853505
33. Di BL, Della TF. Side effects to castor oil. Allergy. 2003; 58(8): 826. https://doi.org/10.1034/j.1398-9995.2003.00221.x PMID: 12859571
34. Final report on the safety assessment of ricinus communis (castor) seed oil, hydrogenated castor oil, glyceryl ricinoleate, glyceryl ricinoleate se, ricinoleic acid, potassium ricinoleate, sodium ricinoleate, zinc ricinoleate, cetyl ricinoleate, ethyl ricinoleate, glycol ricinoleate, isopropyl ricinoleate, methyl ricinoleate, and octyldodecyl ricinoleate. Int J Toxicol. 2007; 26 Suppl 3: 31-77. https://doi.org/10.1080/10915810701663150 PMID: 18080873

35. Deckers N, Ruigrok CA, Verhoeve HP, Lourens N. Comparison of pain response after subcutaneous injection of two maropitant formulations to beagle dogs. Veterinary Record Open. 2018; 5(1): e000262. https://doi.org/10.1136/vetreco-2017-000262 PMID: 29531751
36. Jacob S, VanDaele MA, Brown JN. Treatment of demodex-associated inflammatory skin conditions: a systematic review. Dermatol Ther. 2019; 32(6): e13103. https://doi.org/10.1111/dth.13103 PMID: 31583801
37. Araujo F, Fenton TR, Lukmanji S, Raman M. Olive oil and soybean oil based intravenous lipid emulsions, liver biochemistry and clinical outcomes. Nutrients. 2018;10(6): 658. https://doi.org/10.3390/nu10060658 PMID: 29789518
38. Casati A, Vinciguerra F, Santorsola R, Aldegheri G, Putzu M, Fanelli G. Sciatic nerve block with 0.5% levobupivacaine, 0.75% levobupivacaine or 0.75% ropivacaine: a double-blind, randomized comparison. Eur J Anaesthesiol. 2005; 22(6): 452-6. https://doi.org/10.10 17/s0265021505000773 PMID: 15991509
39. Kuthiala G, Chaudhary G. Ropivacaine: a review of its pharmacology and clinical use. Indian Journal of Anaesthesia. 2017; 55(2): 104-10. https://doi.org/ 10.4103/ 0019-5049.79875 PMID: 21712863
40. Marhofer P, Oismüller C, Faryniak B, Sitzwohl C, Mayer N, Kapral S. Three-in-one blocks with ropivacaine: evaluation of sensory onset time and quality of sensory block. Anesth Analg. 2000; 90(1): 125-8. https://doi.org/10.1097/ 00000539-200001000-00028 PMID: 10624992
41. Lu WD, Hui MQ, Gu JL, Liu L, Wu ML, Yang Y, et al. Pharmacodynamics, toxicology and toxicokinetics of ropivacaine oil delivery depot. BMC Anesthesiol. 2022; 22(1): 113. https://doi.org/10.1186/s12871-022-01653-1. PMID: 35448955
42. Griffiths JD, Le NV, Grant S, Bjorksten A, Hebbard P, Royse C. Symptomatic local anaesthetic toxicity and plasma ropivacaine concentrations after transversus abdominis plane block for Caesarean section. Br J Anaesth. 2013; 110(6): 996-1000. https://doi.org/10.1093/bja/aet015 PMID: 23454825
43. Kalicharan RW, Oussoren C, Schot P, de Rijk E, Vromans H. The contribution of the in-vivo fate of an oil depot to drug absorption. Int J Pharm. 2017; 528(1-2):

595-601. https://doi.org/10.1016/j.ijpharm.2017.06.055 PMID: 28629984

44. Kalicharan RW, Baron P, Oussoren C, Bartels LW, Vromans H. Spatial distribution of oil depots monitored in human muscle using MRI. Int J Pharm. 2016; 505(1-2): 52-60. https://doi.org/10.1016/j.ijpharm.2016.03.064 PMID: 27041126

Table 1. The levels of local adverse events.

Level	Skin reaction
0	No irritation was observed
1	A slight erythema can just be observed
2	Obvious erythema, mild edema or papules were observed
3	Erythema and papules are prominent
4	Edema was significantly
5	Erythema, edema and papules

Table 2. Liquid chromatography tandem-mass spectrometry quantitative liquid phase analysis conditions.

/	Conditions		
Instrument	Agilent 1100 High phase liquid chromatograph (degassing machine G1322A, binary pump G1312A, column temperature chamber G1316A)		
	Automatic sampler (CTC Analytics HTC PAL)		
	Chromatographic column (Hypersil GOLD, 2.1·50 mm, 5 µm)		
Mobile phase	A: Acetonitrile-water-formic acid (10:90:0.2, v/v/v)		
	B: Acetonitrile-water-formic acid (90:10:0.2, v/v/v)		
Gradient elution condition	Time (min)	A%	B%
	0.00	90.0	10.0
	0.50	90.0	10.0
	0.70	5.0	95.0
	2.20	5.0	95.0
	2.21	0.0	100.0
	2.60	0.0	100.0
	2.61	90.0	10.0
	4.00	90.0	10.0

Table 3. Demographics and baseline characteristics (mean±SD).

Group		Study 1: Ropivacaine oily delivery depot	Study 2: Ropivacaine hydrochloride injection	Study 2: Ropivacaine oily delivery depot				
Dose (mg)		12~30	150	150	230	300	350	400
	n	3	3	6	8	8	8	8
Age	Average	28.7±4.1	28.3±2.9	26.7±6.3	28.1±8.1	31.3±10.0	26.3±4.5	33.9±5.1
	Min, Max	25, 33	25, 30	20, 38	19, 43	21, 45	20, 32	28, 43
Sex n (%)	Male	2 (66.7)	3 (100.0)	5 (83.3)	5 (62.5)	6 (75.0)	6 (75.0)	6 (75.0)
	Female	1 (33.3)	0 (0.0)	1 (16.7)	3 (37.5)	2 (25.0)	2 (25.0)	2 (25.0)
Nation n (%)	Ethnic han	2 (66.7)	3 (100.0)	6 (100.0)	8 (100.0)	6 (75.0)	8 (100.0)	7 (87.5)
	other	1 (33.3)	0 (0.0)	0 (0.0)	0 (0.0)	2 (25.0)	0 (0.0)	1 (12.5)
High (cm)	Average	164.3±8.1	171.3±12.2	171.5±6.0	169.9±8.1	166.9±10.1	166.3±7.2	168.2±9.6
	Min, Max	157.0, 173.0	158.0, 182.0	164.0, 179.0	157.5, 183.0	150.0, 183.0	157.5, 179.0	148.0, 179.0
Weight (kg)	Average	61.1±4.4	64.8±9.8	67.4±10.6	65.4±11.1	62.5±10.1	61.2±10.2	63.3±8.8
	Min, Max	57.7, 66.1	54.9, 74.5	55.1, 80.9	50.0, 80.5	47.8, 79.4	47.9, 77.4	50.9, 74.4
BMI (kg/m^2)	Average	22.6±1.3	22.0±0.5	22.8±2.5	22.5±2.1	22.3±2.0	22.0±1.9	22.3±2.1
	Min, Max	21.7, 24.1	21.5, 22.5	19.5, 25.6	20.2, 25.3	20.0, 25.3	19.3, 24.9	19.4, 24.7

BMI, body mass index; SD, standard deviation; Min, minute; Max, maximum.

Table 4. Analgesic range diameter and area under time curve of single-needle abdominal subcutaneous infiltration ropivacaine oily delivery depot (mean±SD, n=3).

Group	Ropivacaine oily delivery depot				
**Dose (mg)**	**12**	**16**	**20**	**24**	**30**
Analgesic range diameter (mm)	31.8±20.8	31.8±20.9	27.4 ±11.6	42.5 ±20.8	46.2±24.3
AUC (h·mm)	304.2 ±229.9	338.4±52.1	286.7 ±29.0	574.2 ±227.6	572.8 ±55.7

AUC, area under time curve; SD, standard deviation.

Table 5. Circumferential distribution of ropivacaine oily delivery depot infiltrated by multiple-needle in the abdomens of the subjects.

Group	Ropivacaine hydrochloride injection	Ropivacaine oily delivery depot				
**Dose (mg)**	**150**	**150**	**230**	**300**	**350**	**400**
**Volum (mL)**	**17**	**7.5**	**11.5**	**15**	**17.5**	**20**
**n**	**3**	**6**	**8**	**8**	**8**	**8**
Circle 1[1], Circle 2[1]	1.2 mL×5	1.2 mL×2	1.2 mL×3	1.2 mL×4	1.2mL×5	1.2mL×6
Circle 3	1.2 mL×4, 0.2 mL×1	1.2 mL×2, 0.3 mL×1	1.2 mL×3, 0.7 mL×1	1.2 mL×4, 0.6 mL×1	1.2 mL×4, 0.7 mL×1	1.2 mL×4, 0.8 mL×1

RODD, ropivacaine oily delivery depot; RHI, ropivacaine hydrochloride injection. 150 mg RHI, n=3; 150 mg RODD, n=6; 230～400 mg RODD, n=8, respectively.
Note[1], RODD or RHI given on circumference 1 and 2, respectively.

Table 6. Incidence of ropivacaine oily delivery depot-related adverse events.

Group	Ropivacaine hydrochloride injection	Ropivacaine oily delivery depot				
**Dose (mg)**	**150**	**150**	**230**	**300**	**350**	**400**
**n**	**3**	**6**	**8**	**8**	**8**	**8**
Erythema at injection site	2	4	2	5	1	3
Papules at the injection site	0	2	0	5	0	0
Hypertriglyceridemia	1	4	1	1	1	3
Hypotension	0	1	4	1	2	1
Abnormal T wave of ECG	0	0	1	2	0	1
Fervescence	0	0	0	1	0	1
ECG ST segment depression	0	0	0	1	0	0
Abnormal ST segment of ECG	0	0	0	0	0	1
Changes in ST-T segment of ECG	0	0	0	0	0	1
Total	3	5	5	7	5	7

ECG, electrocardiogram.

Table 7. Pharmacokinetic parameters for ropivacaine oily delivery depot in healthy subjects (mean±SD).

**Group**	**Ropivacaine hydrochloride injection**	**Ropivacaine oily delivery depot**				
**Dose (mg)**	**150**	**150**	**230**	**300**	**350**	**400**
**n**	**3**	**6**	**8**	**8**	**8**	**8**
C_{max} ($\mu g \cdot mL^{-1}$)	0.58±0.13	0.35±0.09	0.50±0.09	0.74±0.18	0.80±0.12	0.86±0.24
AUC_{0-t} ($h \cdot \mu g \cdot mL^{-1}$)	5.3±0.6	6.2±1.9	8.6±1.9	10.8±2.3	12.3±1.7	15.3±3.7
$AUC_{0-\infty}$ ($h \cdot \mu g \cdot mL^{-1}$)	5.4±6.8	6.2±1.9	8.7±2.0	10.85±2.31	12.4±1.7	15.3±3.7
T_{max} (h)	0.77 ±0.06	8.2±5.9	10.4 ±5.6	3.6± 1.9	8.44± 4.68	11.8 ±4.6
$t_{1/2}$ (h)	7.6 ±5.4	3.4 ±0.96	3.20 ±1.13	3.7± 1.1	2.7 ±0.65	3.6±0.80
Kel (h^{-1})	0.17 ±0.18	0.22± 0.06	0.24 ±0.08	0.20 ±0.06	0.27±0.06	0.20± 0.05
CL ($L \cdot h^{-1}$)	28.3 ±3.5	25.8 ±6.6	27.9 ±7.4	28.6± 5.5	28.8± 4.1	27.8± 7.8

C_{max}, maximum concentration; AUC, area under curve; T_{max}, time of maximum concentration; $t_{1/2}$, half-life; Kel,elimination rate constant; CL, clearance.

Table 8. Nerve block effects of ropivacaine oily delivery depot.

Group		Ropivacaine oily delivery depot				
**Dose (mg)**		**150**	**230**	**300**	**350**	**400**
**n**		**6**	**8**	**8**	**8**	**8**
Onset time (h)[1]	Center of the circle	0.87 (0.43,8.1)	0.5 (0.47,0.55)	0.46 (0.38,0.48)	0.47 (0.42,0.63)	0.42 (0.38,0.42)
	1 cm from the center of the circle	2.4 (0.4,4.2)	0.5 (0.47,0.55)	0.46 (0.38,0.48)	0.47 (0.40,0.63)	0.42 (0.38,0.47)
	3 cm from the center of the circle	0.57 (0.45,4.4)	0.5 (0.46,0.55)	0.47 (0.42,0.52)	0.78 (0.40,4.18)	0.42 (0.38,0.47)
	4 cm from the center of the circle	0.54 (0.45,1.2)	0.53 (0.47,4.1)	0.82 (0.42,4.0)	1.13 (0.42,4.2)	0.42 (0.40,1.0)
Maintenance time (h)[1]	Center of the circle	31.2 (9.02,67.9)	47.5 (22.0,62.0)	52.7 (35.6,71.6)	26.57 (20.9,35.7)	35.73 (26.7,47.9)
	1 cm from the center of the circle	54.7 (13.7,67.9)	47.6 (34.9,68.0)	71.3 (29.7,71.6)	41.54 (19.9,71.6)	35.79 (31.9,47.7)
	3 cm from the center of the circle	37.88 (16.9,71.6)	47.7 (47.6,71.5)	33.8 (23.0,47.6)	43.61 (20.7,67.9)	47.71 (29.8,71.7)
	4 cm from the center of the circle	69.5 (23.1,71.6)	47.7 (35.7,67.9)	47.7 (34.9,67.9)	35.68 (29.6,63.9)	47.43 (26.7,71.7)
Nerve block intensity[1]	Level 1: 5>analgesic intensity score>4	86.1 %	77.5 %	67.2 %	69.2 %	57.1 %
	Level 2: 6>analgesic intensity score ≥5	10.3 %	19.5 %	29.5 %	29.8 %	40.2 %
	Level 3: analgesic intensity score ≥6	3.6 %	3.0 %	3.30 %	0.99 %	2.7 %
Distance between two infiltration points (mm)[2]		40.9±1.3	35.2±2.2	29.6±2.3	24.0±1.9	21.5±1.5

Note [1], Median, 95% , CI; Note [2], mean±SD.

Fig 1. Grouping and flow chart of healthy subjects' abdominal subcutaneous infiltration RODD

Click here to access/download;Figure;Figure-1.png

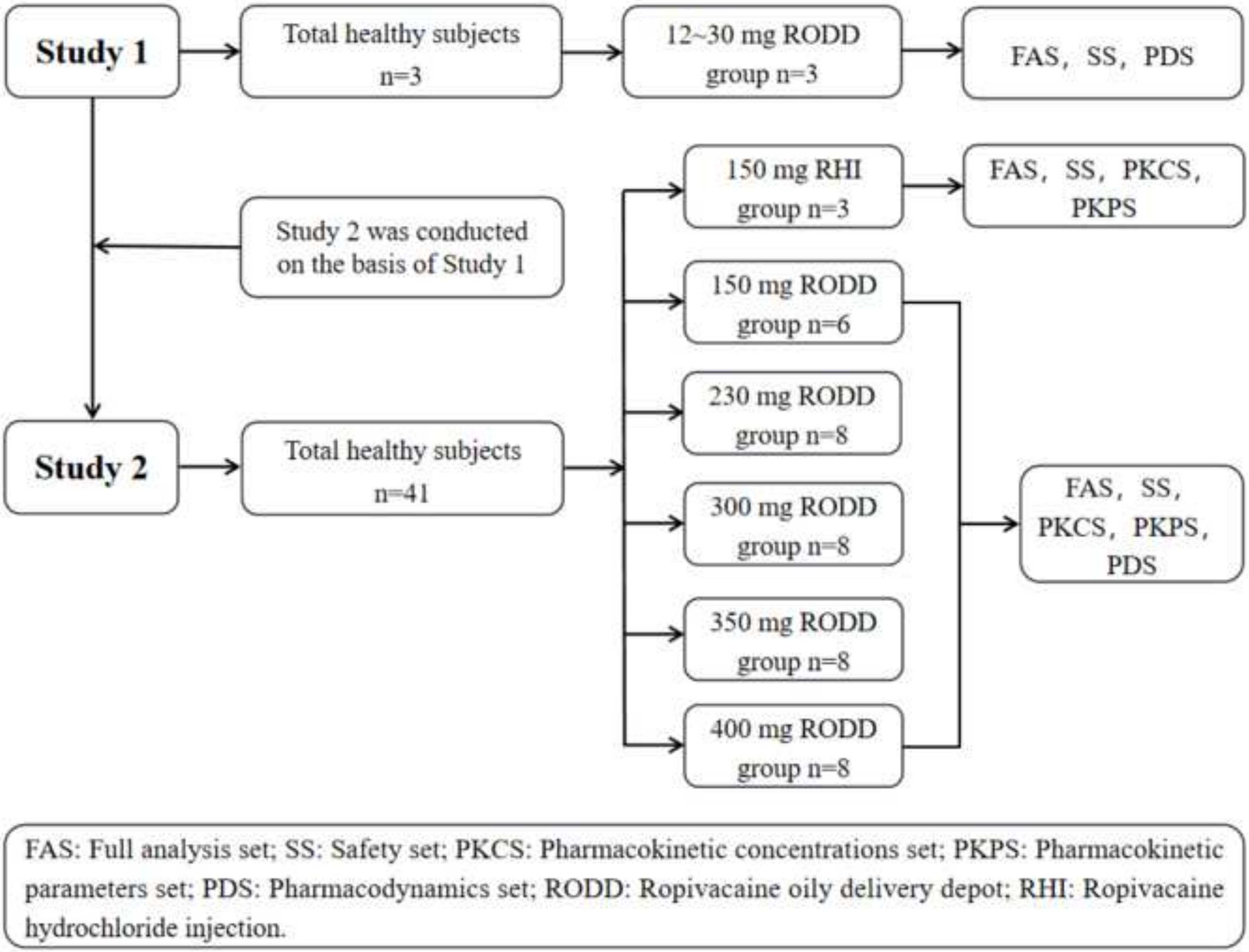

Fig 2. Schematic diagram of healthy subjects' abdominal subcutaneous infiltration of RODD

Click here to access/download;Figure;Figure-2.png

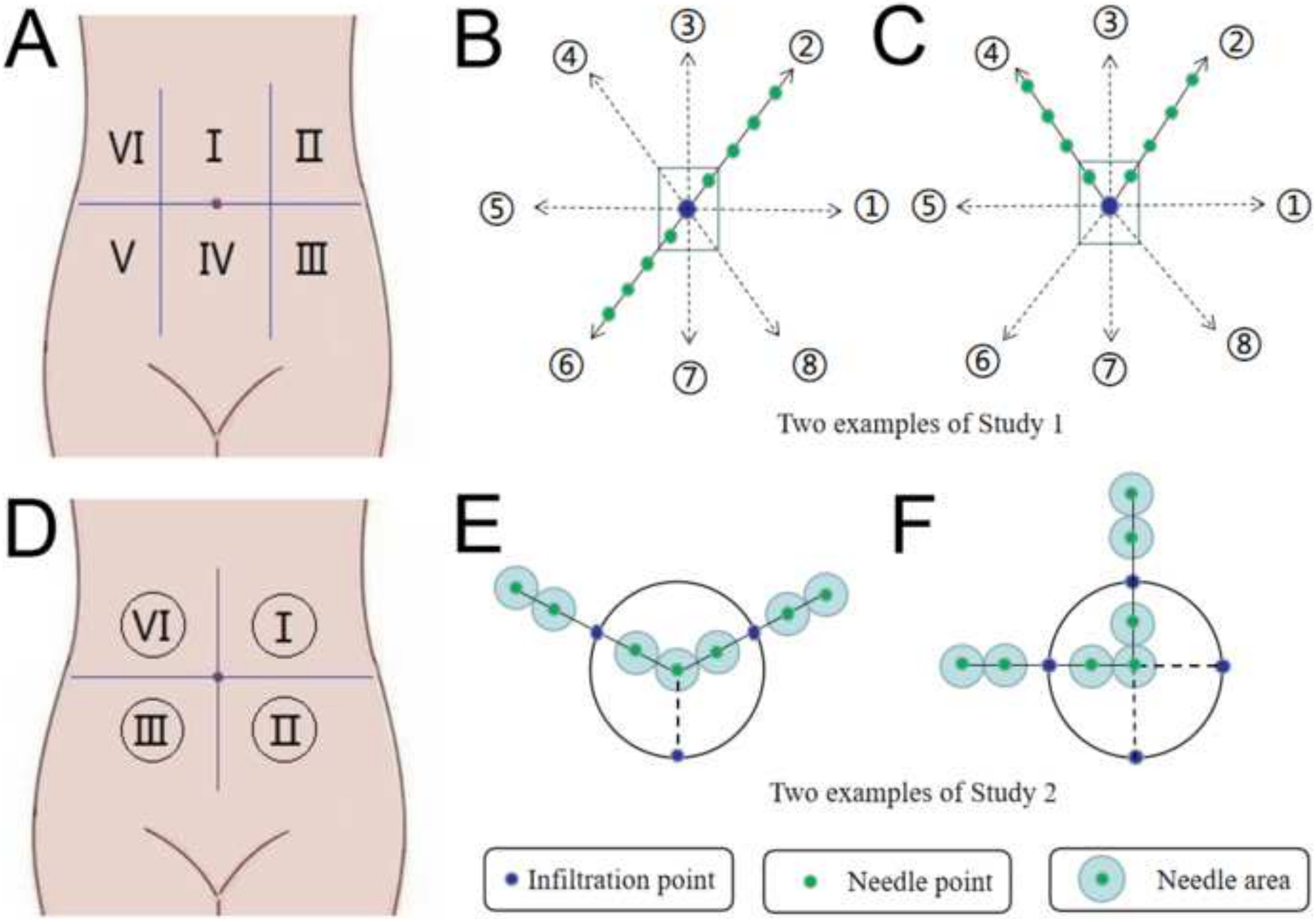

Fig 3. The evaluative process of analgesia range in single-needle abdominal subcutaneous infiltration with RODD in healthy subjects.

Click here to access/download;Figure;Figure-3.png

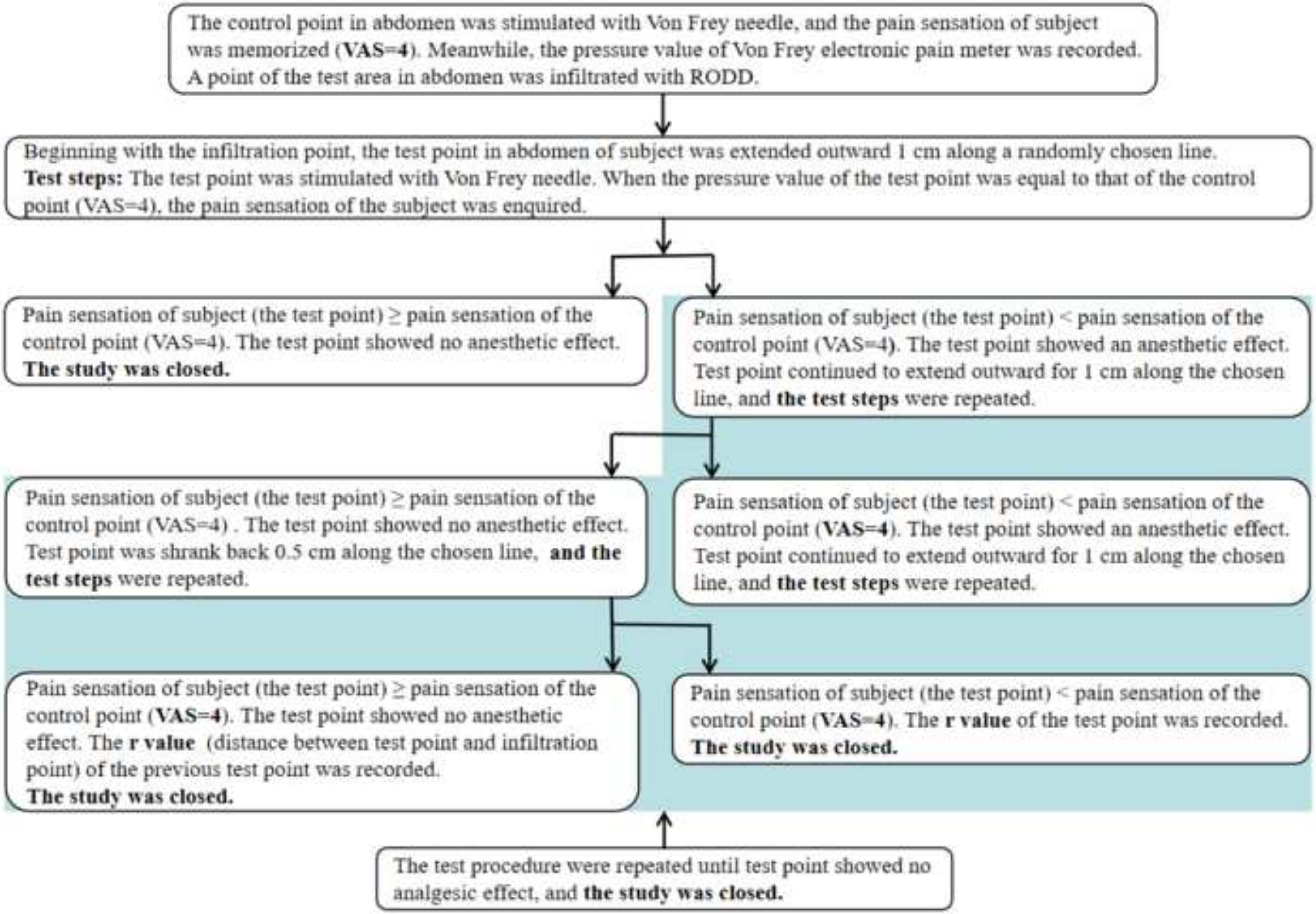

Fig 4. Scoring process of analgesia intensity for subcutaneous multile-needle infiltration of RODD in the abdomens of healthy subjects in Study 2.

Click here to access/download;Figure;Figure-4.png

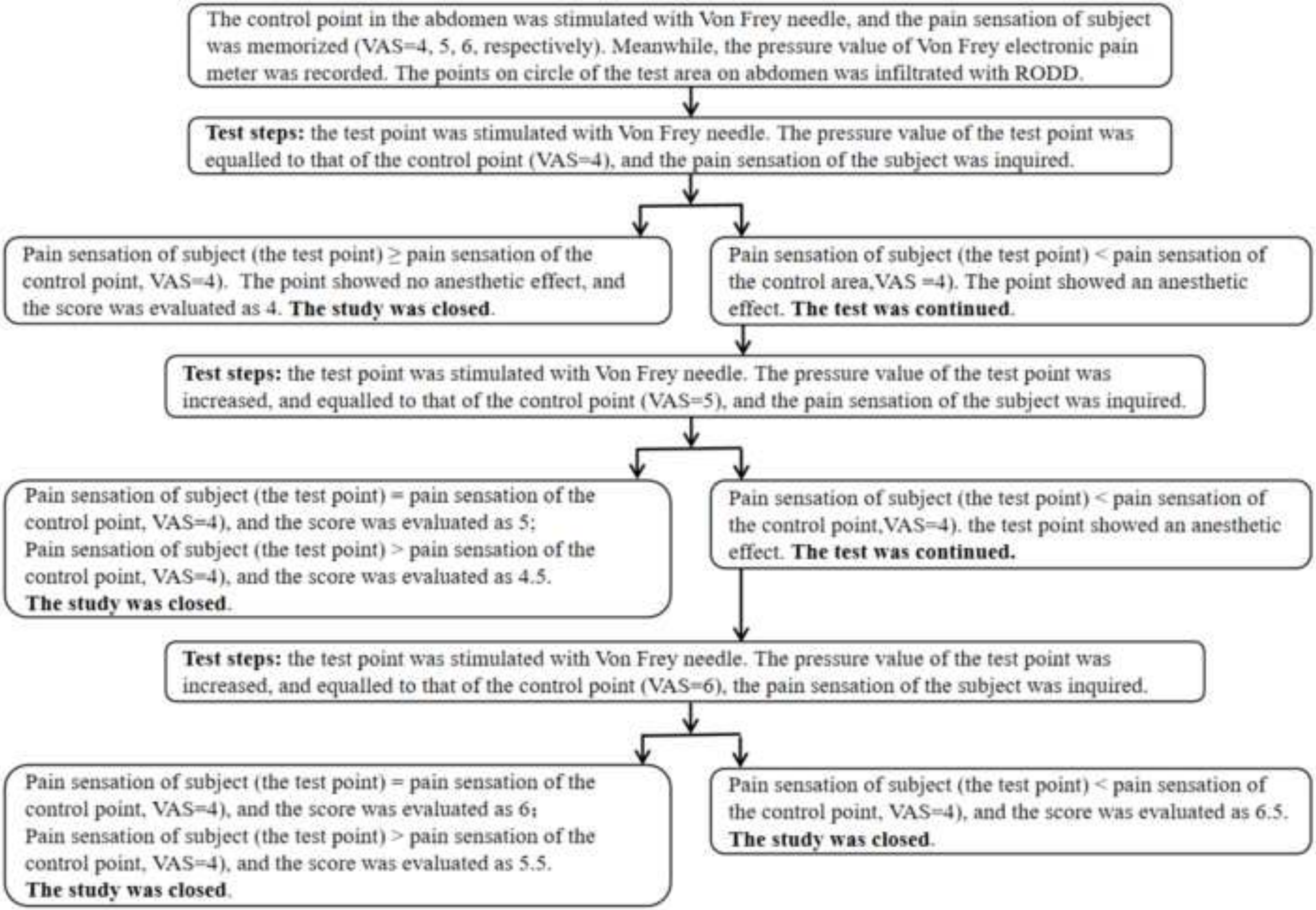

Fig 5. Curves of nerveblock range-time after subcutaneous infiltration 12~30 mg RODD

Click here to access/download;Figure;Figure-5.png

A

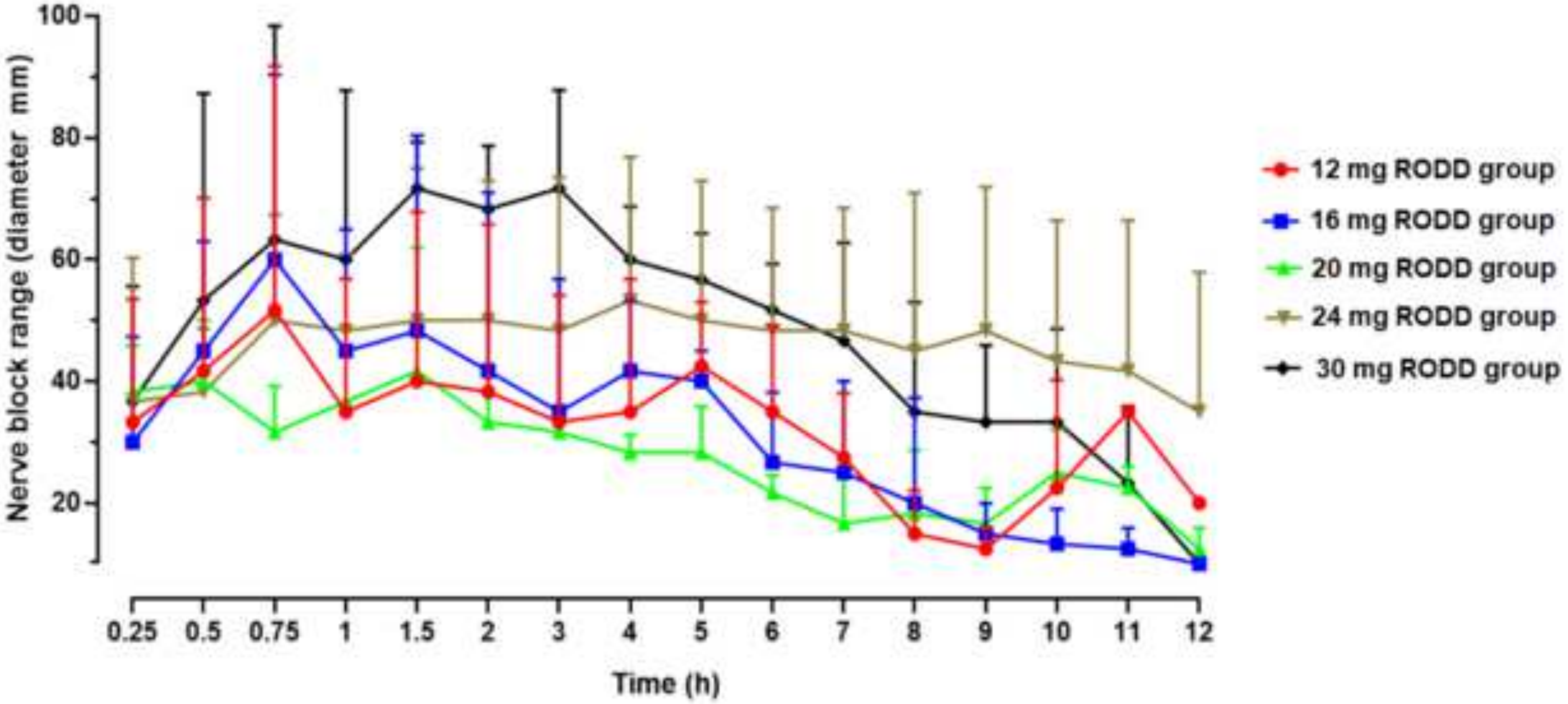

B

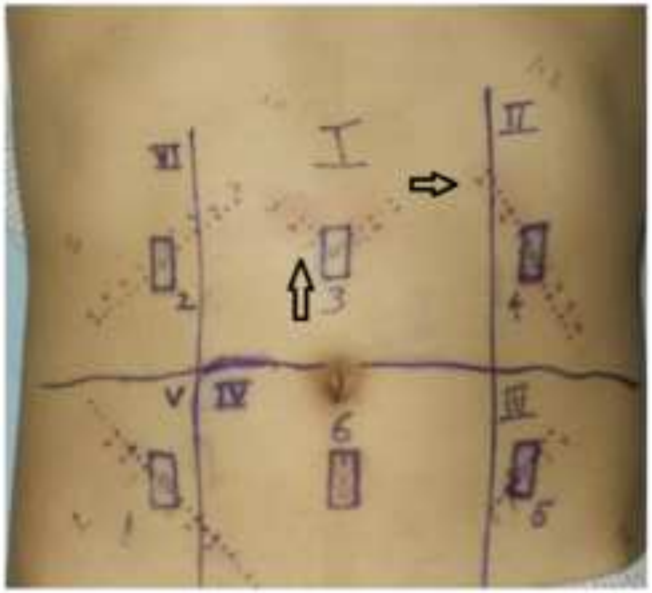

Fig 6. Local skin irritation (arrows) of healthy subjects in Study 2 at 2 h after subcutaneous infiltration RODD.

Click here to access/download;Figure;Figure-6.png

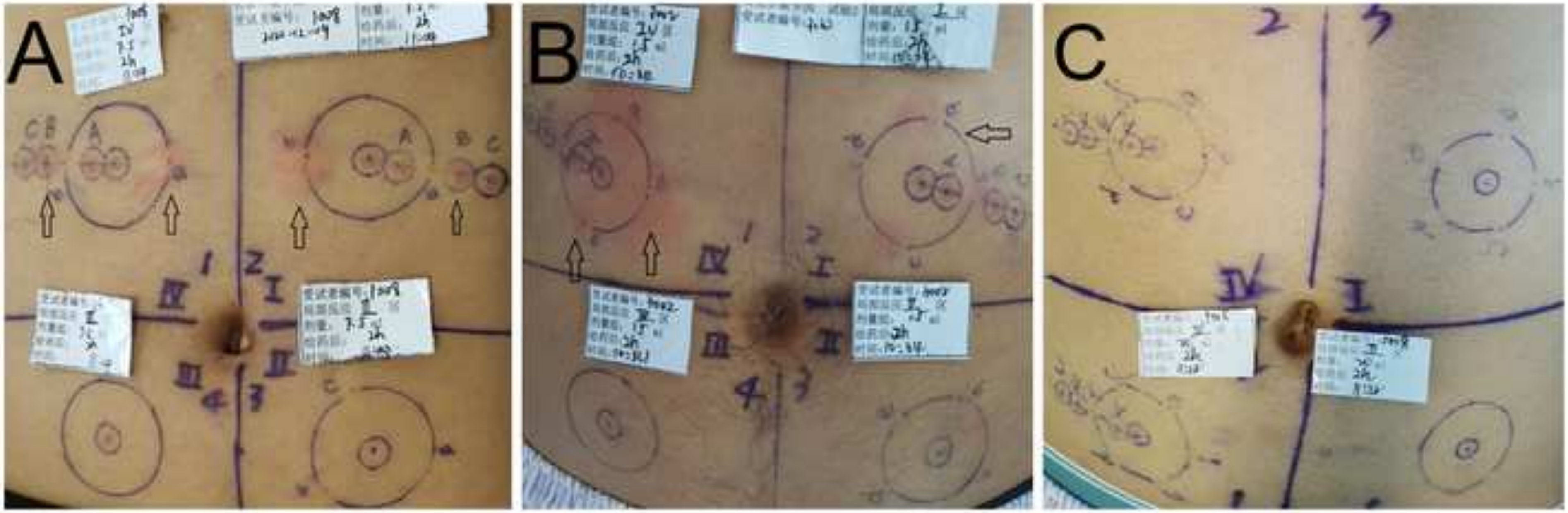

Fig 7. After multineedle subcutaneous infiltration of RODD in healthy subjects' abdomens, curves of ropivacaine concentration-time in plasma, mean±SD.

Click here to access/download;Figure;Figure-7.png

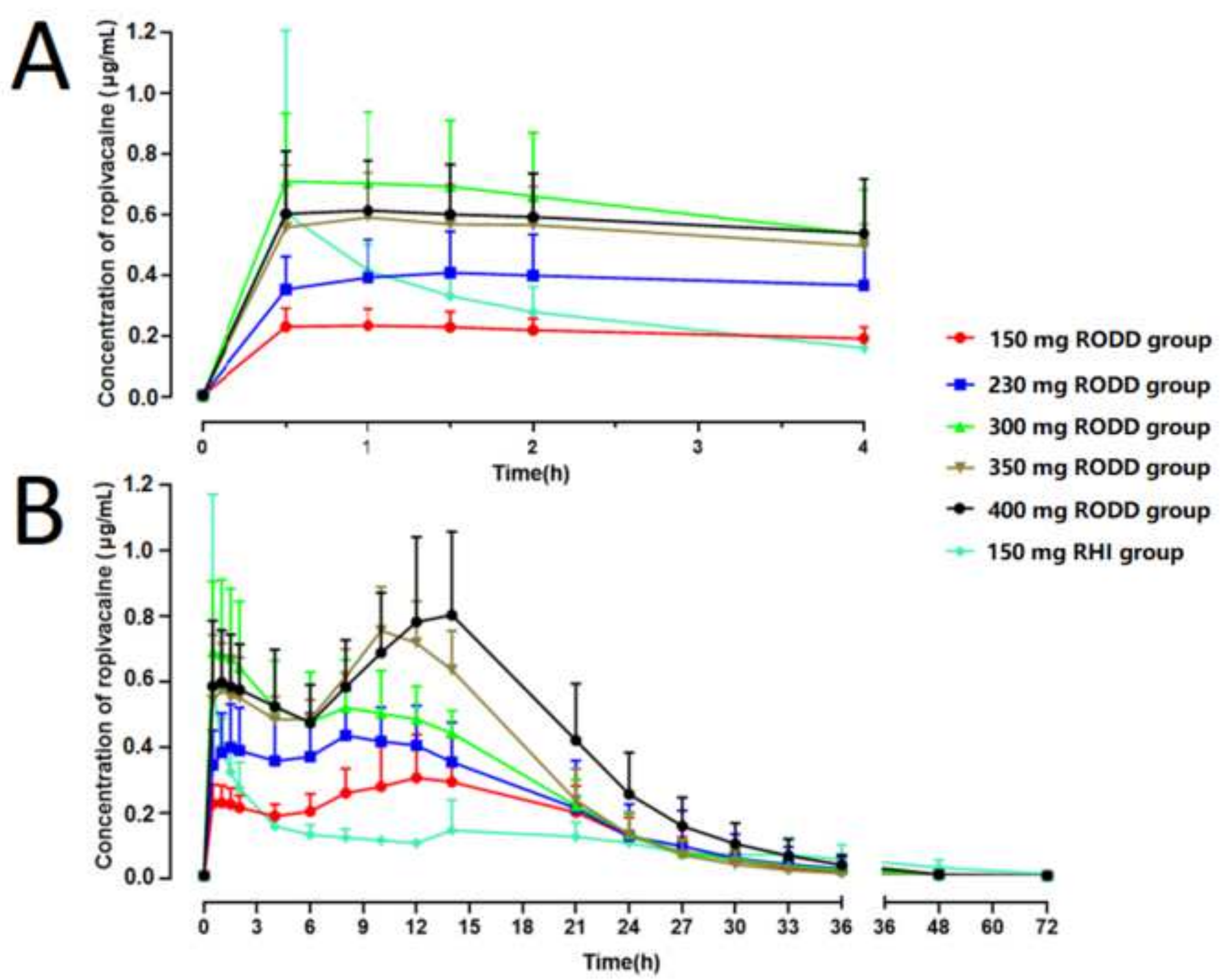

Click here to access/download
Supporting Information
S1. CONSORT 2010 Checklist-10.27.doc

Click here to access/download
Supporting Information
S2. Clinical study protocol (Brief english version)-10.27.pdf

Study protocol (Original chinese version).

Click here to access/download

Supporting Information

S3. Clinical study protocol (Original chinese version)-10.27.pdf

Research article

A randomized trial: the~~A randomized, single-blind study of ropivacaine oily delivery depot:~~ safety, pharmacokinetics and preliminary pharmacodynamics of ropivacaine oily delivery depot~~subcutaneous infiltration~~ in healthy subjects

Formatted: Font: (Default) Times New Roman, 18 pt, Bold, Font color: Text 1

Wu-dang Lu[1,4], Dan-ling Zhao[2], Mei-xia Wang[3], Ya-qi Jiao[4], Ping Chi[3], Min Zhang[2], Bo Ma[2], Jian-ping Dong[1], Hai-bo Zhang[2], Yi Yang[1], Ye Tian[2], Min-quan Hui[1], Bo Yang[2], Yong-xiao Cao[1*]

1 School of Basic Medical Sciences, Xi’an Jiaotong University Health Science Center, Xi~~-~~’an, Shaanxi, China,

2 Beijing Aicomer Pharmaceutical Technology Co., Ltd., Beijing, China,

3 Beijing You An Hospital, Capital Medical University, Beijing, China,

4 Xi’an Libang Pharmaceutical Co., Ltd, Xi’an, Shaanxi, China

*Corresponding author

Address: 76 Yanta West Road, Xi’~~-~~an, Shaanxi 710061, P.R. China

Tel/Fax: +86-029-82655140

Email: yxy@xjtu.edu.cn (YXC)

Formatted: Font color: Text 1

Trial registration

Chictr.org: ChiCTR2200058122;

Chinadrugtrials.org: CTR20192280.

Abstract

Introduction

Ropivacaine oily delivery depot (RODD) can slowly release ropivacaine and block nerves for a long time. The aim of the present work was to investigate reasearch the safety, pharmacokinetics, and preliminary pharmacodynamics of RODD in multi-needlessubcutaneous infiltration into the abdomens of healthy subjects.

Methods

The abdomens of 3 Threesubjects were subcutaneously infiltrated with single-needle subcutaneously infiltrated12~30 mg RODD12~30 mg (RODD containeding 12~30 mg of ropivacaine), severally.– ,and Tthe irritation, nerve blocking range and optimum infiltration dose were investigatedreasearch. researched.Forty-one subjects were divided into RODD containeding ropivacaine 150, 230, 300, 350, 400 mg and ropivacaine hydrochloride injection (RHI) 150 mg groups. Multineedle subcutaneous infiltration of RODD or RHI was performed in the abdomens of the subjects. The subjects' abdomen were subcutaneouslymulti-needlesinfiltrated their RODD or RHI.The primary endpoint was safety or a 400 mg maximum dose. Subjects' vital signs were observed, their blood was analyzed, and their cardiovascular, ,and nervous systems, and irritation were monitored. Secondly, 6 groups randomly to receive subcutaneous infiltration of RODD 150~400 mg or ropivacaine hydrochloride injection (RHI) 150 mg, respectively. The safy indicators of subjects were collected before and 72 h after administration. Tthethe irritation was observed in the infiltrated area, and

theropivacaine in plasma was determined, pharmacokinetic parametersparameners were calculated, and the anesthetic effects of RODD wasere studied, including RODD s'RODDs' onset time, duration and intensity of nerve block were recorded.

Results

RODD 24 mg ofin a A sinsingle-needlegle injected infiltration of RODD 24 mg in 3 subjects the abdomen was optimal, and the range of nerve block was 42.5±20.8 mm. Multi-needles subcutaneous infiltration of RODD in the abdomens of subjects wereas safe, and all adverse reactionevents wasere no more than grade II II. The incidence rate of grade II II adverse events, such as pain ,and abnormal ST and ST-T segment changes on electrocardiography, was approximately of electrocardiogramwas about1%. The irritation of injection was mild. The incidence rate of grade I adverse events, including erythema, papules, hypertriglyceridemia, and hypotension was more than 10%. was more than 10%, and the symptom included erythema, papules, hypertriglyceridemia and hypotension at the infiltrating sites. The incidence rate of grade II adverse events such as pain, abnormal ST and ST-T segment changes of electrocardiogram, etc. was about1%. Erythema and papules were relieved significantly after 24 h, and disappeared basically after 72 h. Other adverse reactions disappeared after 7 days. The curve of ropivacaine concentration-time in plasma presented a double-peak profile,. The resultsis showeds that ropivacaine iwas slowly released from RODD. Compared with the 150 mg RHI groupThe curve of ropivacaine in plasma versus time presented double-peak profile., T_{max} in the RODD groups werewas obviously longerater. In particular, Thetime of sustaining ropivacaine in 400 mgRODD group was the longest.

The T_{max} in RODD groups were obviously later than that in RHI group. T_{max} inthein the 400 mg RODD group of 11.8±4.6 h was longer than that in the RHI group of 0.77±0.06 h (*P*<0.01). Compared with 150 mg RHI group,The the C_{max} in RODDthe 150 mg RODD group was obviously lower than that in the compared with 150 mg RHI group (0.35±0.09 vs. 0.58±0.13 μg·mL^{-1}),. In particularEspeciallyand, the C_{max} increased by 27% and 48% when the dose was increased by 2.4 times in 350 mg group and 2.6 times in the 400 mg group, respectively. C_{max} and AUC were positively correlated with doses. FinallyThese results indicated that RODD had significantly sustained release effect and high safety. Meanwhile, C_{max} and AUC were positively correlated with doses. Llevel 2 of Tthe intensity and duration of nerve block (≥≥levellevel 2) were was positively correlated with RODD doses. The The400 mg RODD 400 mg RODD group presented the strongest nerve block. ,Tthe median of onset time was 0.42 h, and the duration median was 35.7~47.7 h35.7~ ~ ~47.7 h.The mean distance between the two infiltration sites was 21.5±1.5 mm.

Conclusions

RODD The abdomen of subjectRODDcontaining 400 mg of Rropivacaine was multi-needles subcutaneous infiltratedby RODD, the distance between two sites was about 20mm, total dosewas 400mg, which showedis good safety when subcutaneously infiltrated into healthy subjects. Meanwhile, T_{max} was delaysed in the RODD group compared with the RHI group. The median duration medianof nerve block wasiwas more than 35 h. Therefore, RODD hads an obvious sustained release effect.

Formatted: Font: (Default) Times New Roman

showed good safety and sustained release effect in multi-needles subcutaneous infiltration in the abdomen of subjects.

Keywords

Rropivacaine oily delivery depot, safety, pharmacodynamics, pharmacokinetics, healthy subjectsvolunteers, multi-needles infiltration.

Introduction

Ropivacaine is an S-enantiomer amide local anesthetic with high pKa ~~,~~and low lipid solubility~~soluble~~. Its nerve block effects ~~is~~are similar those of~~to~~ bupivacaine, but it has lower neurotoxicity, cardiovascular toxicity, and local muscular toxicity [1-4].

At present, ropivacaine hydrochloride injection is widely used in the clinic. Commonly, the injection is infiltrated subcutaneously at the surgical incision or injected directly at the ganglion for preemptive analgesia~~pre-analgesia~~ before operation [5~~-~~, 6]. During the operation, ropivacaine ~~wa~~is combined with opioids for anesthesia maintenance [7~~-~~, 8]. Moreover, ropivacaine is~~can be~~ used in a variety of ways for postoperative analgesia. 1) A m~~M~~ixture of ropivacaine and opioids is administered for persistent~~administrated for persistence~~ nerve block analgesia by an epidural analgesia pump [9]. 2) Ropivacaine solution is infiltrated subcutaneously~~subcutaneous~~ at the incision, which is suitable for large incision operations with dense cortical nerve distribution, such as in the chest, abdomen and limbs [10-1~~2~~3]. 3) Ropivacaine ~~can be~~is sprayed intraperitoneally~~intraperitoneally~~ to reduce pulling pain,~~,~~ and is often used in digestive system surgery, such as ~~gallbladder,~~ stomach, liver~~;~~, gallbladder, urinary system and gynecological uterus- and ovary-

Formatted: Not Highlight

Formatted: Not Highlight

Formatted: Not Highlight

Formatted: Not Highlight

Formatted: Not Highlight

relatedovaries related laparoscopic surgery [134-18]. Theseis postoperative analgesic managements can effectively relieve postoperative pain ofin patients, meanwhilereduce the amount of prescribed opioidsand the occurrence of adverse reactions, and promote the postoperative recovery.

Formatted: Not Highlight

After the surgical incision beingis infiltrated ofwith ropivacaine hydrochloride solution, a nerve block is produced. The time for the first use of other analgesics is less than 8 h, which cannotcan't meet the needs of postoperative analgesia of more than 24 h [19-, 20]. Althought the increased dose of ropivacaine can prolong the time of nerve block, the increase ofin plasma drug concentration will significantly increase the incidence of related neurotoxicity and cardiovascular toxicity [21]. Therefore, there is obvious clinical significance toin prolonging the postoperative nerve block time of ropivacaine and reducing ethe adverse reactions.

Formatted: Not Highlight

Formatted: Not Highlight

To dateAt present, reasearch of slow-release formulations of ropivacaine havesve been studiedreportedreasearched. Yu et al. preparedRropivacaine liposome gels containing infrared photosensitizers and phospholipids .The efficacy evaluation showed that the gel could be retained *in vivo* for 10 days [22]. Jeon et al. usedPpoloxamer 4087 was used as an excipient to prepare a ropivacaine loaded gel, and used it to treat pain after thoracoscopic pneumonectomy. The analgesic effect was comparable to that of continuous infusion of 0.2% ropivacaine for 48 h [23]. Chen et al. used Ppoloxamer 4087 and polycaprolactone electrospinning fibers wasere used as excipients to prepare ropivacaine hydrogels that which, and studieson blocked rat nerves forblock showed that the efficacy could last for more than 30 h ,etc[24].

Formatted: Not Highlight

Formatted: Not Highlight

Formatted: Not Highlight

However, the evidence of human safety of new excipients is insufficient, and liposomes or hydrogels have the risk of sudden release. ~~However,o~~Oil solutions commonly used in the clinic can avoid the above problems.

Ropivacaine is insoluble in water~~,~~ and ~~wa~~is slowly released into the oil solution~~slowly.~~ ~~Ginosar et al. used~~ C~~c~~astor oil and phospholipids were used as carriers to design a ropivacaine suspension formula ~~, and the results showed that subcutaneous injection of 2.5 mL ropivacaine suspension formula~~~~which~~that could ~~produce~~ sustainabl~~e~~ly block nerve~~s~~ ~~block analgesia~~ for 36 h ~~, about 3 times more than aqueous solution, without adverse reactions~~[225,236]. ~~Teng et al. studied the ropivacaine oil solution based on castor oil administered by epidural. Compared with ropivacaine mesylate aqueous solution, the blocking time of neurotic pain of the ropivacaine oil solution was extended from 4 h to 2 days, and the sustained release effect was significant[24].~~We~~Hui et al.~~ disclosed the dogs' pharmacokinetic data and preliminary toxicity ~~data~~of ropivacaine in soybean oil solution. Compared with ropivacaine hydrochloride injection, the C_{max} value of the ropivacaine oily delivery system was decreased by 80%~~.~~, ~~The average residence time was significantly prolonged. A~~and T_{max} was significantly delayed. ~~The toxicity of ropivacaine oily solution was preliminarily evaluated at the same period. When t~~The dog was given a subcutaneous preparation of 60 mg·kg^{-1}. ~~,~~T~~t~~here ~~were~~ was no significant change in the dog's behaviors, blood count, coagulation, blood, biochemistry urine, etc. ~~,and~~ T~~t~~he oily delivery system of ropivacaine was demonstrated to be ~~highly~~ safe [2576].

Currently, ~~O~~our formula of r~~Ropivacaine formula of Hui et al., was improved, and~~

Formatted: Not Highlight

Formatted: Not Highlight

~~20 mg·mL^{-1} r~~opivacaine oily delivery depot (RODD) ~~was prepared, and~~has been ~~which reduced the amount of benzylalcohol [25].Currently, RODD has been a~~approved by the Center for Durg Evaluation of National Medical Products Administration of China to carry out phase I clinical research. This study simulated the administration method of local infiltration analgesia after abdominal surgery [2~~6~~87], and the safety, pharmacokinetics and preliminary efficacy of RODD in healthy subjects were studied.

Materials and methods

Materials

Ropivacaine was obtained from ~~l~~Libang ~~p~~Pharmaceutical Co., Ltd., China. The purity was 99%. The b~~B~~atch number was C082-190101. Benzyl alcohol and benzyl benzoate were purchased from ~~h~~Hunan ~~e~~Er-kang ~~p~~Pharmaceutical Co., Ltd., China. The b~~B~~atch numbers were 104020190402 and F241C180902, respectively. Soybean oil was produced in ~~t~~Tieling ~~p~~Pharmaceutical Co., Ltd., China. The b~~B~~atch number was 19060203-2-01. Ropivacaine hydrochloride injection (RHI) was manufactured by Astra~~z~~Zeneca ~~Pharmaceutical Co.~~AB, SwedenGermany. The specifications are 100 mg:~~:~~ 10 mL. –The b~~B~~atch number was NBAZ. RODD was ~~mainly~~composed of 2% ropivacaine, 4% benzyl alcohol, 30% benzyl benzoate and approximately~~about~~ 64% soybean oil, ~~which~~and was commissioned to be produced at the GMP (Good Manufacturing Practice)-certified plant of Jiangsu Taikang Biomedical Co., Ltd.~~–~~, ~~B~~batch number~~–was~~ CR20191001. Ropivacaine-D7, a reference substance, was obtained from USP.

Formatted: Font: (Default) Times New Roman

Formatted: Font: (Default) Times New Roman

Formatted: Not Highlight

Formatted: Font: (Default) Times New Roman, 12 pt, Font color: Text 1, Pattern: Clear (Custom Color(RGB(247,248,250)))

RODD preparation

Ropivacaine (200 mg)200mg was added to benzyl alcohol (418 mg)418 mg. After the ropivacaine was dissolved, benzyl benzoate– and soybean oil were added to the ropivacaine solution up to 10 mL. Then, the mixture was heated, and stirred until the solution was clear. After being filtered and sterilized with a filter, the bulk solution, i.e., RODD, was obtained, and divided into vials, filled with nitrogen and sealed. The concentration of RODD was 20 mg·mL^{-1}. In the condition of 2~8℃, the stability study showed that the preparation met the quality standard for more than 2 years., and the stability of RODD was more than 2 years in the condition ofat 2~8°C℃.

Selection of subjectsSubjects

IThe inclusion criteria included an age range of healthy subjects wereof 18~50 years offor men or women, and a body mass index rangeding from 19.0 to 26.0 kg·m^{-2}.

Exclusion criteria included subjects with abnormal vital signs, clinical/laboratory tests (blood routine, urine routine and blood biochemical, serology, electrocardiogram (ECG).), cerebrovascular, liver, kidney, respiratory, blood, endocrine, immune, mental diseases, gastrointestinal system; porphyria patients, malnutrition or blood disease, scar constitution; 2 weeks taking drugs, in particular, analgesics; long-term drinking of tea and coffee, blood donation offering within three monthsthrough blood, and participated in other drug clinical tests within three months subjects tested.

Formatted: Indent: First line: 2 ch

A total of 50 healthy subjects were selected, with no restriction on men and women. males and females

Formatted: Justified, Indent: First line: 2 ch

andThey were informed about the purpose, procedure, possible benefits and risks of the study and, signed the Iinformed Cconsent Fform.

Formatted: Indent: First line: 2 ch

Ethics statement

All of healthy subjects participated in Phase I clinical study, of which 6 were collected blank plasma sample for pharmacokinetic baseline. The age range of subjects was 18~50 years and body mass index ranged from 19.0 to 26.0 kg·m^{-2}. Subjects of vital signs, clinical/laboratory tests (blood routine, urine routine and blood biochemical, serology, electrocardiogram (ECG), etc.) without exception, centerless cerebrovascular, liver, kidney, respiratory, blood, endocrine, immune, mental disease, gastrointestinal system, there was no potential risk for the difficult airway, no long QT syndrome, or QTc interphase> 450 ms, or indoor conduction block and/or QRS > 120 ms, no accidental history of anesthesia, no abnormal pain, etc. After checking the criteria in the study, the healthy subjects were informed about the purpose, procedure, possible benefits and risks of the study and, signed the Free and Informed Consent Form (ICF). The research was designed and conducted in accordance with the current "Good cClinical pPractice (GCP) of China" in China, "Pharmaceutical Administration Law of the People's Republic of China", "The International Council for Harmonisization of Technical Requirements for Pharmaceuticals for Human Use (ICH)ICH-GCP", "Guidelines for eEthical rReview of dDrug cClinical tTrials" and "Declaration of hHelsinki". Clinical trial protocol revision and implementation were approved and supervised by the Hospital Ethics Committee. For the first time, the protocol of the clinical study was approved by the Ethics Committee of Beijing You'an Hospital

affiliated with ~~to~~ Capital Medical University on September 5, 2019. The study ~~was~~ recruited the first subject on December 2, 2019, and enrolled the last participant on June 25, 2021.

The study has completed registration in the drug clinical trial registration and information disclosure platform, ~~and~~ which is the legal registration platform for clinical information used by the drug evaluation center of the state medical products administration of ~~c~~China ~~(CDE)~~. The clinical trial record number is CTR20192280 in ~~c~~China. At present, ~~T~~the study has completed registration in Chinese Clinical Trial Registry (ChiCTR), and the clinical trial record number is ChiCTR2200058122.

Trial design ~~and subjects~~

Three subjects were single-needle infiltrated subcutaneously with different doses of RODD in the abdomen to explore the optimal dose, anesthesia range, and irritation. The abdomens of ~~F~~forty-one subjects ~~were~~had multiple ~~multi-~~needles infiltrated subcutaneously with an escalating ~~incremental doses~~dose of RODD. The primary endpoint was safety, defined as the occurrence of grade III~~Ⅲ~~ adverse events, or the attainment of RODD's maximum (containing 400 mg of ropivacaine). Meanwhile, ~~to study~~ the ~~safety,~~ pharmacokinetics ~~,~~and preliminary effects were studied. The grouping and test process ~~wa~~is shown in Fig. 1.

Formatted: Not Highlight

Fig. 1. Grouping and flow chart of healthy subjects' abdominal subcutaneous infiltration RODD.

Formatted: Not Highlight

Study 1: Single-needle subcutaneous infiltration exploration~~exploratory~~

Three subjects were selected. Fig~~.~~ 2A show~~eds~~ that the abdomen of each subject was divided into six regions from the ensisternum~~,~~ and marked I to VI clockwise. According to the random number table from the computer, any area was selected as the starting point and marked 1~5 clockwise. The infiltration doses of RODD (containing ropivacaine) were 12 mg (0.6 mL), 16 mg (0.8 mL), 20 mg (1.0 mL), 24 mg (1.2 mL) and 30 mg (1.5 mL)~~ orderly (containing 12–30 mg of ropivacaine)~~. Area 6 was used as the blank control. An interval of 1 h between two adjacent ~~different~~doses was used to observe the adverse reactions~~ of RODD~~. This experiment was based~~Basing~~ on the VAS score standard [2~~7~~98]. The range of nerve block was measured through acupuncture by a Von Frey electronic pain meter (IITC ~~l~~Life ~~s~~Science) before and 0.25, 0.5, 0.75, 1, 1.5, 2, 3, 4, 5, 6, 7, 8, 9, 10, 11, and 12 h after subcutaneous administration.

The test methods are shown in Fig 2B and C.

The infiltration points in the abdominal region were taken as the starting point, ~~respectively,~~eight test directions were designed, and two lines (r1 and r2) were randomly selected to test the nerve block effect in 8 directions around the infiltration point that started from the infiltration point. Test points were set along the line from the infiltration point, and the distance between two points was 1 cm. The test steps are shown in Fig 3.

Formatted: Not Highlight

Formatted: Not Highlight

Formatted: Indent: First line: 2 ch

Formatted: Font color: Blue

Formatted: Not Highlight

two directions (r1 and r2) were randomly selected to test the nerve block effect,

Test points were set along the line from the infiltration point, and the distance between two points was 1 cm.

as shown in Fig. 2B and C. The test steps wereare shown in Fig. 3, t

The nerve block range (r1+r2) at each infiltration point was recorded and the area under the block range-time curve was calculated to determine the optimal infiltration dose.

At 0.5 h before administration, and at 0.3, 0.6, 1, 2, 6, 24, and 72 h after administration, the symptoms of infiltration sites were recorded, and the irritation wereas scored.

Fig. 2. Schematic diagram of healthy volunteersubjects' abdominal subcutaneous infiltration of RODD. Wherein, A and D wereare the distribution designs of single-needle and multi-needle infiltration areas under the abdomen subskincutaneously, which were areas I~V I~~VI and I~IV, ~~IV, respectively. B and C wereare examples of single-needle infiltration points and needle test points, respectively.;; Two lines were randomly selected in 8 directions around the infiltration point that which started from the infiltration point. Test points were set along the line from the infiltration point, and the distance between two points was 1 cm. E and F wereare examples of multi-needle infiltration points and acupuncture test points on a circle, respectively. Two infiltration points on a circle arewere randomly selected, that which were connected to the center of the circle and extended outward 2 cm,respectively. The four

Formatted: Not Highlight

Formatted: Highlight

Formatted: Not Highlight

Formatted: Not Highlight

Formatted: Not Highlight

Formatted: Not Highlight

Formatted: Not Highlight

Formatted: Not Highlight

Formatted: Not Highlight

~~test points on the straight line are the center of the circle, 1 cm away from the center of the circle (inside the circle), and 1 and 2 cm away from the infiltration point (outside the circle). The test area was within 0.5 cm around the test point, and each point or area was tested once.~~

Fig~~.~~ 3. The ~~evaluativ~~evaluative ~~e~~process of analgesia range in single-needle abdominal subcutaneous infiltration with RODD ~~was performed~~ in healthy subjects.

Formatted: Not Highlight

Study 2: Multiple-needle~~s~~ subcutaneous infiltration

~~Forty-one s~~The subjects were randomly divided into 150, 230, 300, 350 and 400 mg RODD groups (n=6~8~~)~~ ~~,~~), and the ropivacaine hydrochloride injection (RHI) 150 mg group (n=3)~~randomly~~, and each subject was subcutaneously infiltrated with RODD or RHI. The abdomen was divided into four areas by drawing horizontal and vertical lines through the navel, as shown in Fig~~.~~ 2D, marked with Ⅰ, Ⅱ, Ⅲ~~,~~ and Ⅳ clockwise~~)~~.

Formatted: Not Highlight

One area ~~i~~was randomly selected and marked as 1, while the other areas ~~ar~~were marked clock~~-~~wise as 2~4. Areas 1~3 were infiltrated RODD, and area 4 was the blank control for the nerve block test. A point in each region was selected as the center of the circle (the distance between the two centers w~~ere~~as more than 8 cm), and the optimal block range and dosage w~~as~~ere obtained in ~~s~~Study 1~~,~~. ~~and t~~The number of needles in each dose group was calculated as follows:

Number of needles in the group = total dose of the group/optimal dose;.

Number of needles on circle 1 or 2 = the number of needles on each group/3.

The

remaining needles were allocated to the third circle. The needles on the circumference should be evenly distributed. The intensity of the nerve block in the abdominal infiltrating area was measured in all subjects at different times after administration. The subjects were hospitalized for 72 h, and followed up for 7 days after discharge.

Nerve block intensity

As shown in Fig. 2E and F, two infiltration points on a circle were randomly selected, which were connected to the center of the circle and extended outward 2 cm, respectively. The four test points on the straight line are the center of the circle, 1 cm away from the center of the circle (inside the circle), and 1 and 2 cm away from the infiltration point (outside the circle). The test area was within 0.5 cm around the test point. the center of the circle was aligned withlined to the infiltration point, and the four test points were distributed on the line. The test area was a circle with a radius of 0.5 cm and centered on the test point. Referring to the VAS method, pain intensity was tested by acupuncture in any two directions of circles 1 and 2 [2798], and each point or area was tested once. The evaluative process of nerve block intensity wais shown in Fig. 4.

Fig. 4. Scoring process of analgesia intensity for subcutaneous multile-ple needles

Formatted: Not Highlight

Formatted: Not Highlight

Formatted: Not Highlight

infiltration of RODD in the abdomens of healthy subjects in ~~s~~Study 2.

Safety assessment

Vital signs such as blood pressure, pulse, respiration and oxygen saturation were measured in the supine position ~~of~~for the subjects ~~in~~1 h before and 1, 2, 4, 8, 12, 24, 48 and 72 h after administration. Routine blood tests, electrocardiograms, blood biochemistry and urine tests were performed 24 h before and 72 h after administration. The symptoms of infiltration sites were recorded, and ~~the~~irritation was scored at 0.5 h before and 0.3, 0.6, 1, 2, 6, 24 and 72 h after administration. The criteria for local adverse events ~~were~~are shown in ~~t~~Table 1. The safety evaluation was based on the Evaluation Criteria for "Common Adverse Events" ~~that which~~ published by Chinese Clinical regulatory authorities (V5.0, 2017.11). Routine ~~B~~blood tests~~routine~~ included red blood cell count, white blood cell count, hemoglobin, hematocrit, platelet count, eosinophil count, neutrophil count, and lymph cell count, monocyte count, etc. Routine ~~U~~urine ~~routine~~ examination items included glucose, ketone bod~~y~~ies, protein, urinary occult blood, white blood cells, bilirubin, nitrite, ~~urochologen,~~ specific gravity, etc.~~.~~ Blood biochemical ind~~ex~~icee~~s~~ included potassium, sodium, chlorine, calcium, magnesium, phosphorus, blood glucose, creatinine, urea, glutamine transferase, uric acid, lactate dehydrogenase, total bilirubin, aspartic ammonia acid aminotransferase, alanine aminotransferase, alkaline phosphatase, triglyceride, total cholesterol, total protein, albumin and so on.

Pharmacokinetics

Formatted: Not Highlight

The liquid chromatography coupled with tandem mass spectrometry (LC–MS/MS)LC–-MS/MS analysis analysic method of ropivacaine was optimized on the basis of previousthe reports [283029-30321]. The liquid chromatography analysis conditions wereare shown in tTable 2, including a column temperature of 20°C℃, injection volume of 10 μL, flow rate of 0.40 mL·min^{-1}, and sample chamber temperature of 2~8°C℃. Mass spectrometry conditions included API 4000 mass spectrometer, Turbo Ionspray (APCI^{+}) ion source, MRM detection mode, GS1 and 2 of 60 psi, TEM: 500°C℃, CUR: 30 psi, CAD: 5 psi, ropivacaine ion pair: 275.1/126.1, scanning time 200 ms, DP: 60 V, EP: 10 V, CE: 24 V; CXP: 13 Vv. The extraction and detection method was were verified, and the content of ropivacaine in plasma samples wereas determined.

Formatted: Not Highlight

At 0.25 h before and 0.5, 1, 1.5, 2, 4, 6, 8, 10, 12, 14, 21, 24, 27, 30, 33, 36, 48 and 72 h after administration, 4 mL of venous blood was drawn into a blood collection tube containing EDTA-K_2 anticoagulant, and centrifuged with at 2000 × g for 10 min. The upper layer of plasma was transferred into 2 mL cryopreserved tubes (Axygen, made in USA) ,and then stored in a to refrigerator (-60~-90 °C℃)within 2 h. The plasma was treated with the protein precipitation method before analysis. Specifically, the plasma wereas thawed at room temperature and blended by vortexing, 50 μL of plasma was transferred into a centrifuge tube, and added160 μL of ropivacaine-D7 acetonitrile solution (internal standard ropivacaine-D7 15 ng·mL^{-1}) was added. After 1 min of vorticesexing, the sample was centrifuged at 12000 rpm for 10 min, , and 40 μL supernatant was transferred into the sample tube,. Then,added 160 μL ultra-pure water was added to the same tube, and wellvortexed for testing. The injection

volume was 10 μL for quantitative analysis by LC–MS/MS.

Preliminary pharmacodynamics

At 0.25 h before and 0.25, 1, 4, 8, 10, 12, 14, 18, 21, 24, 27, 30, 33, 36, 48 and 72 h after administration, the analgesia intensity was measured by acupuncture on circles 1 and 2 of the abdomen, and the onset time and effective duration were evaluated. The distance between two adjacent infiltration points was recorded.

Statistical analysis

All data were analyzed by ~~software~~ SAS software (version 9.4), and the indicators of each group were compared with baseline. The ropivacaine concentration-time curves were drawn, and pharmacokinetic parameters were calculated (Phoenix Win Nonlin 8.2). Local irritation scores of RODD were evaluated by descriptive statistics, and the frequency and percentage of categorical or ordinal variables were summarized. The mixed effect model was used to analyze the changes ~~of~~in acupuncture pain intensity at different time points before and after administration. In the model, changes in pain intensity at different time points in different administration areas compared with baseline were dependent variables. Pain intensity before administration was a covariable. ~~Administration~~ Administrative area/blank area, grouping and visit point were fixed effects. The onset time and duration of RODD nerve block were compared by median (95%, CI)~~,~~. The analgesic intensity was calculated by percentage, an~~and~~ the ~~analgesic intensity and~~ range of nerve block~~distance adjacent infiltration points~~ and distance adjacent infiltration points were expressed as the ~~by~~ mean±SD. ~~There was significant difference when *P*< 0.05.~~

Results

Subject disposition and demographics

A total of 44 healthy subjects, with 75% malesmen and 25% womenfemales, with an average age of 29.3±±7.1 years, were enrolled. The Han nationality accounted for 93% and other ethnic groups accounted for 7%. The average height was 168.6±±8.4 cm, the average weight was 63.9±±9.7 kg, and the average body mass index was 22.3± ±1.9 kg·•/m^{-2}. The specific demographic data of healthy subjects are shown in Table 3. In addition, whole blood and blank plasma samples from 6 healthy subjects were collected for the methodology and stability study of the required biological samples. Health subjects were included in study if they met the criteria. Demographic data were shown in table 3.

Optimal dose of single-needle infiltration RODD

After single-needle subcutaneous infiltration of RODD, the block range-time curve wais shown in Fig. 5A. In the 24 mg and 30 mg RODD24 mg and 30 mgRODD groups, the nerve block range (i.e., average diameter, 42.5±20.8 and 46.2±24.3 mm)– and the areas under the curve (574±228 and 573±56 h·mm) were significantly higher than thatose in the 12, 16 and 20 mg groups (Table 4). The anesthesia duration was couldmaintained for more than 12 h. Secondly, slight erythema around the injection site whichwas alleviated after 2 h, with an theincidence of 28%, and disappeared after approximatelyabout 24 h (Fig. 5B). The local irritation of RODD inat the 24 mg infiltration points was slightly lower than that inat the 30 mginfiltration point. It was

Formatted: Font: Times New Roman

Formatted: Not Highlight

Formatted: Not Highlight

Formatted: Not Highlight

Formatted: Not Highlight

suggested that the optimal dosage was 24 mg (1.2 mL) with thea block range of 40 mm.

Fig. 5. Curves of nerveblock range-time after subcutaneous infiltration 12~~≃30 mg RODD (Mmean±SDdian, A). Irritation after subcutaneous infiltration RODD for 2 h on the abdomen of the subject (B), and slight erythema appeared on the skin around some infiltration points (arrows).

Safety of multiple-needles infiltration RODD

The abdomen of the subjects wereas divided into 4 regions, on whichand a circle with a diameter of 40 mm was marked in every region. The number of infiltration points and drug quantity distribution on the circle wereare shown in tTable 5. The incidences of adverse reactions of subjects in the RODD150, 230, 300, 350, 400 mg RODD, andRHI 150 mg RHI groups were 83.3% (5/6), 75.0% (6/8), 100.0% (8/8), 62.5% (5/8), 87.5% (7/8) and 100.0% (3/3), respectively. Common grade I adverse events included erythema at the infiltrating site (40.9%, 18/41), papules (15.9%,— 7/41), hypertriglyceridemia (25.0%, 11/41), andhypotension (20.4%, 9/41). There wereas no correlation between the incidence of adverse events and doses. Erythema and papules wereare shown in Fig. 6. Grade II adverse events mainly included ST segment depression and ST segment abnormality (2.3%, 1/41). There wasere no grade III or above adverse events (Table 6). The mMost of the erythema and papules in the infiltrating site were relieved 24 h after administration, and almost disappeared within

Formatted: Not Highlight

Formatted: Not Highlight

Formatted: Not Highlight

72 h. Blood pressure and ECG returned to normal 24 h after administration.~~ ~~The data suggested that RODD has good safety~~ and tolerability~~.

Fig 6. Local skin irritation (arrows) of healthy subjects in ~~s~~Study 2 at 2h after subcutaneous infiltration RODD. A show~~eds~~ mild erythema around the infiltration point in the ~~RODD~~150 mg ~~RODD~~RODD group. B show~~eds~~ erythema and mild papules around the infiltration site in the ~~RODD~~ 300 mg RODD ~~RODD~~ group, and C show~~eds~~ no abnormalities around the infiltration site in~~ RODD~~ 400 mg ~~RODD~~RODD group.

Methodological verification

The analysis method of ropivacaine had superior selectivity. The standard curve range was 2.5~~~~2000 ng·mL^{-1}, the correlation coefficient was greater than 0.998, the inter batch precision (CV-%) was 0.81%~~-~~~2.05%, the inter batch accuracy (RE-%) was -2.45% ~2.06%, and the lowest limit of quantification was 2.5 ng·mL^{-1}. Quality control samples were prepared by adding different doses of ropivacaine to blank plasma, and then the samples were processed according to the sample handling method. The r~~R~~ecovery rates of the quality control samples (the quality control of low (7.5 ng·mL^{-1}), medium (150 ng·mL^{-1}) and high (1.5 mg·mL^{-1}) concentrations) were 102.1%±4.5%, 102.8%±1.5% and 103.1%±1.9%, respectively. The average recovery was 102.7%±0.5%. The internal standard recovery was 97.9%±2.2%, and the matrix effects were 99.3%±2.8%, 100.6%±3.2% and 99.3%±2.0%, respectively. The average matrix

Formatted: Not Highlight

Formatted: Not Highlight

Formatted: Not Highlight

Formatted: Font: (Default) Times New Roman

Formatted: Font: (Default) Times New Roman

effect was 99.7%±0.8%. The Iinternal standard matrix effect was 99.3%±3.2%, without hemolysis nor hyperlipidemia effects. The in-batch precision (CV-%) and accuracy (RE–%) of the quality control samples were 1.15%~4.99% and -2.45%~6.93%, respectively, – and the interbatch precision (CV_%) wereas 2.87%~3.56%, and the interbatch accuracy (RE–%) wereas 1.32%~4.42%. The quality control sample presented good stability at -20°C℃ and -70°C℃ for 2 years, -70°C℃ for repeated freeze- –thaw cycles, room temperature and 2~8°C℃ for 3 days after 75 h of room temperature storage, which couldmeet the determination requirements.

Pharmacokinetics

The average plasma concentrations-time curve of ropivacaine in each group wereis presented in Fig. 7. RThe ropivacaine concentration in the RHI group reached the peaked at 0.5 h after administration, and then decreased rapidly, suggesting that the release of ropivacaine was rapid. The ropivacaine concentration-time curves in the RODD groups presented double peaks, with the first peak at 1~2 h and the second peak at 610~15 h. After the second peak, the concentration of ropivacaine decreased slowly. The pharmacokinetic Pharmacokinetic parameters wereare shown in tTable 7. Compared with the C_{max} in the RHI group (0.58±0.12 µg·mL^{-1}), the C_{max} of theRODD 150 mg RODD group (0.35±0.09 µg·mL^{-1}) was significantly decreased and had significant difference (P<0.05). And tThe C_{max} of values of the RODD 230 and 300 mg RODD groups increased gradually. Moreover, the C_{max} values of the 350 and 400 mg groups were 0.80±0.12 and 0.86±0.24 µg·mL^{-1}, respectively, and the increases hadwere significant difference (P<0.01). The T_{max} value of the RHI group was 0.77±0.06 h, and

Formatted: Not Highlight

Formatted: Not Highlight

the T_{max} of the RODD groups wereranged from 3.63±4.98 h to 11.81±4.58 h(*P*<0.01). The aApparent volume of distribution (Vd) of the RHI group was 290.6±185.8 L, and the Vd of the RODD groups decreased to the ranges from112.7±35.3 L to 152.3±48.0 L (*P*<0.01), but there was no significant different ofce in the Vd among the RODD groups (*P*>0.05). The result of –average concentration- time semilog of Rropivacaine in each group were wasissuggested showednshowed in Fig. 8 that R ropivacaine was continuously released from the RODD into the blood before the second peak, which offset part of ropivacaine's metabolism and approximated the kinetics of order zero. Ropivacaine's metabolismelimination in vivo exhibited linear pharmacokinetic characteristics after complete release, namely, the first-order atrioventricularmodel. This was the same metabolic pattern as that reported for ropivacaine hydrochloride injection [32] being reported.

Fig. 7. After multi-needles subcutaneous infiltration of RODD oin healthy subjects' abdomens, curves of ropivacaine concentrations-time in plasma, , that whichare expressed astheas the bymean±SD. Wherein, tThe time ranges of A and B were 0~~ 4 h and 0~~72 h, respectively.

Fig. 8. The curves of ropivacaine concentration-time semilog in plasma after abdomen of healthy subjectRODD weremultiple needle s subcutaneously infiltrationedion of RODD into the abdomens of healthy subject. RODD, which expressed by mean±±SD, RHI 150 mg group, n=3; RODD 150 mg group, n=6;

Formatted: Font color: Text 1

Formatted: Font color: Text 1

Formatted: Font color: Text 1

Formatted: Font color: Text 1

Formatted: Not Highlight

Formatted: Not Highlight

Formatted: Not Highlight

Formatted: Font: Not Bold, Not Highlight

Formatted: Font: Not Bold, Not Highlight

Formatted: Not Highlight

Formatted: Font: 12 pt, Highlight

Formatted: Font: 12 pt, Highlight

Formatted: Font: 12 pt, Highlight

RODD 230 mg group ~~400 mg group, n=8, respectively.n=3-8..

Fig 8. The curves of ropivacaine concentration-time semilog in plasma after multineedle subcutaneous infiltration of RODD into the abdomens of healthy subject. mean±SD, 150 mg RHI group, n=3; 150 mg RODD group, n=6; 230 mg~ 400 mg RODD group, n=8.

Onset, duration, and intensity of nerve block

The onset time of nerve block shortened with theincreasing RODD eof dose after subcutaneous infiltration of RODD, and the time of the 400 mg group was the fastest, with a median of 0.42 h in all test areas. The duration of the nerve block was presenteddose-independent, and the median of the 400 mg RODD 400 mgRODD group wereas 35.7~~47.7 h. When the scores of nerve block intensity were greater than 4 (>level 1), the analgesic rate of 150~400~~400 mg RODD groups were 100%. In the scores of nerve block intensity were greater than and equal to 5 (≥ ≥level 2) the analgesic rate of 150, 230, 300, 350, and 400 mg RODD groups were 13.9%, 22.5%, 32.8%, 30.8%, 42.9%, respecfively, and that was positively correlated with the dose of RODD. Finally, in the scores of nerve block intensity were greater than and equal to 6 (level 3), the analgesic rate of the RODD groups ranged from 0.99% to 3.6%, a small percentage with no significant dose correlation. Therefore, the highest nerve block intensity was found in the 400 mg RODD group, and the distance between two infiltration points on the circumference was 21.5±1.5 mm (Table 8).When the scores of

Formatted: Font: 12 pt, Highlight

Formatted: Font: 12 pt, Highlight

Formatted: Font: Not Bold, Highlight

Formatted: Font: Not Bold

Formatted: Not Highlight

Formatted: Not Highlight

Formatted: Font: 12 pt

Formatted: Font: (Default) Times New Roman, 12 pt, Font color: Text 1

Formatted: Font: (Default) Times New Roman

Formatted: Font: 12 pt

nerve block intensity were greater than 4 (≥level 1), the percentage of the RODD groups were 100%; When the anesthesia intensity score were greater than 5 (≥ level 2), the percentage of the RODD groups were 13.9%, 22.5%, 32.8%, 30.8%, 42.9%, and that was positively correlated with the dose of RODD. Finally, when the anesthesia intensity score were greater than 6 (≥ level 3), the percentage score of the RODD groups ranged from 0.99% to 3.6%, a small percentage with no significant dose correlation. Therefore, the highest nerve block intensity was found in the 400 mg RODD group, and the distance between two infiltration points on the circumference was 21.5±1.5 mm (Table 8).

The nerve block intensity was analyzed by percentage, showing a first-order negative correlation ,and a second-order positive correlation. Therein, the percentage of first-grade scores at the center of the circle was 57.1%, the second-grade score was 40.2%, and the third-grade score was 2.7% in the 400 mg 400 mgRODD group. The distance between two infiltration points on the circumference was 21.5±1.5 mm in the 400 mg RODD RODD400 mgRODDgroup (Table 8).

Formatted: Highlight

Formatted: Highlight

Formatted: Highlight

Formatted: Highlight

Discussion

An iInjectable oily delivery depot (ODD) is aone of classic oily drug delivery system, whichthat is a vehicle dosage formfor traditional endocrinehormone or psychotropic drugs. Currently, there have been clinical studies on the application of ODD in the field of anesthesia such as the formulation of ropivacaine with castor oil and phospholipids.

However, the high irritant and sensitization of castor oil isarecallingcuases for concern [31323, 32434]. In contrast, the RODD formulation that we designed for clinical trials, with high concentrations of ropivacaine However, Ropivacaine in formulation ofRODDhas a strongerremarkable nerve block analgesic effect. Benzyl alcohol is a rapid analgesic for commonly injections. Benzyl benzoate has an anti-inflammatory effect, and soybean oil is the main excipient for lipid emulsion for injection [33545-35767]. Therefore, excipients of RODD wereads judged mayto have abetter clinical safety and anesthetic effects.In addition, excipients, benzyl alcohol have potential synergistic effect on ropivacaine in the RODD formulation.

Formatted: Not Highlight

Formatted: Not Highlight

RODD was allowed for further clinical studies in healthy subjects after evaluation in animal studies, which was been further evaluated for the safety, pharmacokinetics andpreliminary efficacy in healthy subjects. In exploratory studies, there was no slight obvious local irritation at a single maximum infiltration dose of 1.5 mL RODD 24 mg. Local irritation performance slightly increased after multi-needles subcutaneous administration,;. iIn most cases, such as erythema and papules appeared after 2 h of multi-points infiltration of RODD, and most of themit was relieved after 24 h and disappeared after 72 h. after It was speculated that the local irritation might be related to the overlapping infiltration range of two adjacent twoneedles. administration.There was no correlation between local irritation and total dose of everyone. For example, irritation in 300 mg RODD group was actually higher than that in the 400 mg group. The incidence of RODD-related adverse events waslow and mild, including hypotension, abnormal ST-segment electrocardiogram, and hypertriglyceridemia,

which returned to normal 72 h after administration. In addition, two subjects in the RHI group had mild erythema in the infiltrated area, and one subject had hypertriglyceridemia, which quickly disappear~~resolv~~ed. These transient and mild adverse reactions may be caused by ropivacaine, excipients, frequent clinical administration, or topical disinfectants. In addition, no abnormal mental reaction ~~swere~~ was observed. Therefore, RODD was demonstrated to be~~is~~ safe~~ty~~. The results support~~ed~~ the follow-up study of subcutaneous infiltration analgesia in patients~~ incisions~~.

The preliminary pharmacodynamic~~s~~ assessment of RODD was divided into two parts. The recommended~~ation~~ amount of RODD in single-needle administration was 24 mg in the exploratory experiment, the duration of analgesia was approximately~~about~~ 12 h, and the infiltration range was approximately~~about~~ 40 mm. The circle on the abdomen~~abdominal~~ of the subject was uniformly infiltrated with 6 needles in the 400 mg RODD~~400mg~~ group, the distance between two adjacent needles ~~i~~was 20 mm, and the subcutaneous infiltration range ~~was~~ overlapped each other, suggesting that the anesthesia time of RODD multi-point infiltration was further prolonged. The efficacy of local infiltration of RHI ha~~d~~s been reported [39~~7~~8-~~41~~~~39~~40]. The analgesic onset time and duration of RHI were approximately~~about~~ 10~45 min and 6~8 h, respectively. Compared with RHI, the analgesic onset time of the 400 mg RODD~~400 mg~~ group was similar~~ (vs. 0.42 h, median)~~, and the duration of analgesia was extended by more than 6 times (vs. 35.7~47.7 h, median), which could ~~basically~~ meet the needs of postoperative analgesia. Therefore, the analgesic effect of RODD was significantly prolonged, and the potential clinical advantages were obvious.

The concentration-time curves of ropivacaine for RODD were significantly different from those for RHI. The RHI curve rose rapidly after administration, and the curve decreased rapidly after 0.77 h (T_{max}) and was then prolonged at a lower concentration. The first peak (589.4+158.9 ng/·mL^{-1}mL,) of the curve in the RODD 400 mg group was reached at 1 h after administration, and the second peak (864.9+237.5 ng·mL^{-1}/ml) was reached at 11.8 h (T_{max}). Then, the curve decreased slowly. The curves of the 150~-350 mg RODD group waeres similar to that of the 400 mg RODD group. This phenomenon has also been found in preclinical animal studies of RODD, including pharmacokinetics in dogs and toxicokinetics in rats [26, 401]. This bimodal phenomenon had not been found with other clinically used oily preparations. The metabolic pathway of ropivacaine in the body is clear; and ropivacaine has it is no t reabsorbed into the bloodstreamhepato-enteric ciculation [32].,. Therefore, we hypothesized that the bimodal ropivacaine-time curve characteristic of RODD in subcutaneous infiltration was caused by the continuous release of ropivacaine, which might be related to the jointcombinative effect of free ropivacaine and ODD.; The ropivacaine outside of RODD releases to produce the first peak. With the metabolism of ODD, then, ropivacaine inside of RODD re-releases to generate the second peakor it might be related to the local vascular distribution of the infiltrated area or the vasoconstriction effect of ropivacaine.

The curves of ropivacaine concentration-time of RODDgroupswith different dosesshowed double peaks in the clinical study.ies,

Formatted: Subscript

Formatted: Font: (Default) Times New Roman, 12 pt

Formatted: Font: Font color: Text 1

Formatted: Not Highlight

Formatted: Font: (Default) Times New Roman, 12 pt, Font color: Text 1, Pattern: Clear

Ropivacaineinjection 注射

Tthe first peakappeared between 1~2h, and the second peak appearedbetween 6~15h.and This phenomenon was found in preclinical animal experiments, and dogs' pharmacokinetic studies of RODD had been reported[42].The first peak appeared at about 1h but was not significant in the ropivacaine concentration-time curve,,and the second peak (C_{max}) appeared at about 11h.Similarly, toxokinetic results in rats have been reported[43], with a significant bimodal phenomenon in the RODD 300mg/kg group, which occurred at about 2h and 8h, respectively. This bimodal phenomenon had not been found with clinically used oily preparations. The metabolic pathway of ropivacaine in the body is clear, it's not reabsorbed into the bloodstream[44]. Therefore, we hypothesized that the bimodal ropivacaine-time curve characteristic of RODD infiltration in the subcutaneous abdomen of healthy subjects was caused by the continuous release of ropivacaine, which might be related to the joint effect of ropivacaine and ODD, or it might be related to the local vascular distribution of the infiltrated area or the vasoconstriction effect of ropivacaine, and so on.

Pharmacokinetic studies of RODD had showned thatropivacaine was continuously released in local tissues for more than 36 h with extended-release. T the T_{max} of the RHI 150 mg group was lower than 1 h, while that of the RODD 150~400 mg groups lagged 2.43.6~11.08 h, respectively, suggesting that ropivacaine can be released slowcontinuously. Compared with the 150 mg RHI group, the C_{max} value inthein the RODD 150 mg RODD group decreased by 40%, and the value increased by 27%, and

48% when the doses were increased to 350 mg and 400 mg, respectively. This valueIt is much lower than the reported C_{max} of ropivacaine hydrochloride injection at the maximum tolerated dose (2.9±±0.5μg·mL^{-1}/mL) [4512]. In addition, the C_{max} value in 400 mgRODD group was only 25% of that as the reported maximum tolerated dose of 2.9±0.5μg/mL of ropivacaine [36].All of the above findings suggest that RODD is safetety. Secondlly, the elimination half-life ($t_{1/2}$) of the 150 mg RHI 150mg group was 7.6±±5.4 h, which was higher than that ofthe 150 mg RODD 150mg group. The reason might be the small number of subjects in the RHI group, or the difference ofin ropivacaine concentration between RODD and RHI. Finally, Ccompared with the 150 mg RHI group, we found that the Vd value of the 150 mg RODD group was reduced by approximatelyabout 67%, and there was no obvious difference among the RODD groups, which demonstrated that there was a certain resistance to the subcutaneous diffusion of RODD. ItThis might be one of the mechanisms of the sustained release of RODD.Therefore, compared with RHI, the sustained release effect of RODD was significant.

Formatted: Not Highlight

Formatted: Subscript

At present, the release mechanism in ODD has been further studied.

The preliminary pharmacodynamics assessment of RODDwas divided into two parts. In exploratory experiment, the reason that the recommendation amount of RODD in single needleadministrationwas 1.2 mL but not 1.5 mL, might be the difference in the distribution of subjects'abdominal subcutaneous nerves. Meanwhile, the analgesic ranges of both doses were about 40 mm. Namely, the diameter of circles in the subjects subcutaneous multi-needles infiltration RODD experiment was 40 mm. RODD was

~~evenly infiltrated in many points on the circle. There were 6 infiltration points on the circle in the 400mg group, and the distance between the two adjacent infiltration points was about 20 mm, which was consistent with the analgesia range of the dose exploratory experiment. This results suggested that the analgesic effect of RODD in patients with subcutaneous multipoint infiltration around abdominal incision would be perfect.~~

~~Secondly, the efficacy of local infiltration of RHI had been reported[37-39]. The analgesic onset time and duration of RHI are about 10~45 min and 6~8 h, respectively. Compared with RHI, the analgesic onset time of RODD 400 mg group was similar (vs 0.42h, median), and the duration of analgesia was extended by more than 6 times (vs, 35.7~47.7 h, median), which basically met the needs of postoperative analgesia. Therefore, the analgesic effect of RODD was significantly prolonged and the potential clinical advantages were obvious.~~

~~With the clinic research of RODD, release mechanisms ofdrug in ODDwere further studied.~~ For instance, Kalicharan et al. reported the metaboli~~e~~sm of ODD in different tissues, and measured its spatial distribution by magnetic resonance imaging technology to visualize the changes of in ODD *in vivo*. They found that ~~injection of~~1 mL ODD was injected into the vastus lateralis and deltoid muscles in healthy ~~volunteer~~subjects, and the ODD could be cleared from the body within a week [4~~062~~3, 4~~173~~4]. This provided support for us to further study the release mode of ropivacaine from RODD *in vivo*. The mechanism of RODD is still being investigated~~studied*in vivo*~~, and we will report it in the future.~~Dorrit et al. compared the pharmacokinetics of~~

bupivacaine hydrochloride aqueous solution and bupivacaine hydrochloride oil-based delivery storage in rats by subcutaneous injection, and found that drug release kinetics was a first-order reaction*in vivo*, which was correlated with the results of rotary dialysis membranerelease kinetics*in vitro* [42,43].

Conclusions

The results of a phase I clinical study studies showed that RODD of subcutaneous infiltration was safe, and has a sustained release analgesic effect.

RODD ,at no more than 400 mg shows a good safety with slight irritation. RODD can slowly release ropivvacaine to produce a long-duration analgesia withfor more than 35 h, which can be used tofor long-term analgesia in the clinic.

Formatted: Indent: First line: 0 ch

Supporting information

S1 Checklist. CONSORT 2010 checklist.

S2 File. Study protocol (Brief english version).

S3 File. Study protocol (Original chinese version).

Acknowledgments

The authors are grateful to all the subjects who took part in the trial, as well as the team of doctors' team in the Department of Anesthesiology and the Phase I Clinical Research Institution at You An Hospital, Capital Medical University.

Author Contributions

Conceptualization: Wu-dang Lu, Yong-xiao Cao.

Formal analysis: Wu-dang Lu, Mei-xia Wang, Bo Yang.

Investigation: Wu-dang Lu, Dan-ling Zhao, Mei-xia Wang, Ya-qi Jiao, Min Zhang, Hai-bo Zhang, Jian-ping Dong, Yi Yang, Ye Tian, Min-quan Hui, Bo Yang.

Validation: Hai-bo Zhang, Ye Tian.

Methodology: Dan-ling Zhao, Ya-qi Jiao, Ping Chi, Bo Ma, Yong-xiao Cao.

Resources: Dan-ling Zhao, Mei-xia Wang, Ping Chi, Min Zhang, Yi Yang.

Project administration: Wu-dang Lu, Dan-ling Zhao, Mei-xia Wang, Ya-qi Jiao, Ping Chi, Bo Ma, Jian-ping Dong, Min Zhang, Hai-bo Zhang, Yong-xiao Cao.

Writing–original draft: Wu-dang Lu.

Writing–review & editing: Wu-dang Lu, Yi Yang, Yong-xiao Cao.

Formatted: Indent: First line: 0 ch

References

~~Mcclellan KJ, Faulds D. Ropivacaine: an update of its use in regional anaesthesia.~~

Formatted: Highlight

Formatted: No bullets or numbering

~~Drugs. 2000;60(5):1065-93.PMID: 11129123.~~

1. Mcclellan KJ, Faulds D. Ropivacaine: an update of its use in regional anaesthesia. Drugs. 2000; 60(5): 1065-93. https://doi.org/10.2165/00003495-200060050-00007 PMID: 11129123
2. Knudsen K, Beckman SM, Blomberg S, Sjövall J, Edvardsson N. Central nervous and cardiovascular effects of i.v. infusions of ropivacaine, bupivacaine and placebo in volunteers. Br J Anaesth. 1997; 78(5): 507-14. https://doi.org/10.1093/bja/78.5.507 PMID: 9175963
3. Yutaka ODA. Ropivacaine: pharmacokinetics and toxicity. The journal of Japan Society for Clinical Anesthesia. 2009; 29(4): 519-27. https://doi.org/10.2199/ jjsca.29.519.
4. Viderman D, Ben-David B, Sarria-Santamera A. Analysis of bupivacaine and ropivacaine-related cardiac arrests in regional anesthesia: a systematic review of case reports. Rev Esp Anestesiol Reanim. 2021; 68(8): 472-83. https://doi.org/10.1016/ j.redare.2020.10.005 PMID: 34538765
5. Zhou HY, Ou MC, Yang YX, Ruan Y, Pan Y, Li Y. Effect of skin infiltration with ropivacaine on postoperative pain in patients undergoing craniotomy. Springerplus. 2016; 5(1): 1180. https://doi.org/10.1186/s40064-016-2856-3 PMID: 27512639
6. Kang KH, Kim BS, Kang H. The benefits of preincision ropivacaine infiltration for reducing postoperative pain after robotic bilateral axillo-breast approach thyroidectomy: a prospective, randomized, double-blind, placebo-controlled study. Ann Surg Treat Res. 2015; 88(4): 193-9. https://doi.org/10.4174/astr.2015.88.4.193 PMID: 25844353
7. Sun XF, Zhou Q, Zhou M, Cao R, Chen ZL, Tang SJ, et al. The effect of epidural nalbuphine combined with ropivacaine on epidural analgesia during labor: a multicenter, randomized, double-blind, controlled study. The Clinical Journal of Pain. 2021; 37(6): 437-42. https://doi.org/10.1097/AJP.0000000000000928 PMID: 33758132
8. Zhou SQ, Wang J, Du WJ, Song YJ, Xu ZD, Liu ZQ. Optimum interval time of programmed intermittent epidural bolus of ropivacaine 0.08 with sufentanyl 0.3

Field Code Changed

µg/mL for labor analgesia: a biased-coin up-and-down sequential allocation trial. Chin Med J. 2020; 133(05): 517-22. https://doi.org/10.1097/CM9.0000000000000669 PMID: 32142491

9. Shelke PD, Nale SR, Babriya J, Tiwari S. A comparative study of epidural ropivacaine 0.2% with nalbuphine and ropivacaine 0.2% with fentanyl in unilateral total knee replacement surgeries. Int J Med Anesthesiology. 2020; 3(3): 14-7. https://doi.org/10.33545/26643766.2020.v3.i3a.140.

10. Mattila I, Pätilä T, Rautiainen P, Korpela R, Nikander S, Puntila J, et al. The effect of continuous wound infusion of ropivacaine on postoperative pain after median sternotomy and mediastinal drain in children. Paediatr Anaesth. 2016; 26(7): 727-33. https://doi.org/10.1111/ pan.12919 PMID: 27184591

11. Choromanski DW, Patel PS, Frederick JM, Lemos SE, Chidiac EJ. The effect of continuous interscalene brachial plexus block with 0.125% bupivacaine vs 0.2% ropivacaine on pain relief, diaphragmatic motility, and ventilatory function. J Clin Anesth. 2015; 27(8): 619-26. https://doi.org/10.1016/j.jclinane.2015.03.006 PMID: 26216250

12. Zhang Y, Liu L, Zheng L, Chen J, Huang L, Wang Q, et al. Comparison of effectiveness of ropivacaine infusion regimens for continuous femoral nerve block for recovery after total knee arthroplasty: a randomized double-blind trial. J Pain Res. 2020; 13: 997-1005. https://doi.org/10.2147/JPR.S247158 PMID: 32494188

13. Li BL, Liu XZ, Cui LH, Zhang WQ, Pang H, Wang MS, et al. Local infiltration analgesia with ropivacaine improves postoperative pain control in ankle fracture patients: a retrospective cohort study. Pain Research and Management. 2020 (Special issue): 1-7. https://doi.org/10.1155/2020/8542849 PMID: 32215137

14. Collins GG, Gadzinski JA, Fitzgerald GD, Sheran J, Wagner S, Edelstein S, et al. Surgical pain control with ropivacaine by atomized delivery (spray): a randomized controlled trial. J Minim Invasive Gynecol. 2016; 23(1): 40-5. https://doi.org/10.1016/j.jmig.2015.07.018 PMID: 26241686

15. Choi SW, Cho SJ, Moon HW, Lee KW, Lee SH, Hong SH, et al. Effect of intercostal nerve block and nephrostomy tract infiltration with ropivacaine on

postoperative pain control after tubeless percutaneous nephrolithotomy: a prospective, randomized, and case-controlled trial. Urology. 2018; 114: 49-55. https://doi.org/10.1016/j.urology.2017.12.004 PMID: 29288788

16. Samal D, Sahoo R, Patra K. Multisite ropivacaine for pain relief after laparoscopic cholecystectomy: a placebo controlled study at a tertiary care teaching hospital. Int Surg J. 2018; 5(1): 115-9. http://dx.doi.org/10.18203/2349- 2902.isj20175550
17. Alevizos L, Zavridis P, Kalles V, Albanopoulos K, Menenakos E, Zografos G, et al. Efficacy of incisional infiltration and intraperitoneal instillation of ropivacaine for the management of pain after laparoscopic sleeve gastrectomy: A randomised clinical trial. Eur J Anaesthesiol. 2020; 37(9): 829-33. https://doi.org/10.1097/EJA.0000000000001206 PMID: 32769515
18. Liu DS, Guan F, Wang B, Zhang T. Combined usage with intraperitoneal and incisional ropivacaine reduces pain severity after laparoscopic cholecystectomy. Int J Clin Exp Med. 2015; 8(12): 22460-8 PMID: 26885228
19. Weinbroum AA. Postoperative hyperalgesia-A clinically applicable narrative review. Pharmacol Res. 2017; 120: 188-205. https://doi.org/10.1016/j.phrs.2017.02.012 PMID: 28365208
20. Sommer M, de Rijke JM, van Kleef M, Kessels AG, Peters ML, Geurts JW, et al. The prevalence of postoperative pain in a sample of 1490 surgical inpatients. Eur J Anaesthesiol. 2008; 25(4): 267-74. https://doi.org/10.1017/S0265021507003031 PMID: 18053314
21. Winkler M, Alt A, Dietzsch S, Kunz SN. Two fatal and four surviving cases after accidental infusion of ropivacaine. Forensic Toxicol. 2021; 39, 506–12. https://doi. org/10.1007/s11419-021-00574-x.
22. Hou Y, Meng X, Zhang S, Sun F, Liu W. Near-infrared triggered ropivacaine liposomal gel for adjustable and prolonged local anaesthesia. Int J Pharm. 2022; 611: 121315. https://doi.org/10.1016/j.ijpharm.2021.121315 PMID: 34826592
23. Jeon JH, Seong YW, Han JE, Cho S, Kim JH, Jheon S, et al. Randomized trial of poloxamer 407-based ropivacaine hydrogel after thoracoscopic pulmonary resection. Ann Thorac Surg. 2021; 114(4): 1189-96. https://doi.org/10.1016/

j.athoracsur PMID: 34653384.

24. Chen S, Yao W, Wang H, Wang T, Xiao X, Sun G, Yang J, Guan Y, Zhang Z, Xia Z, Li M, Tao Y, Hei Z. Injectable electrospun fiber-hydrogel composite sequentially releasing clonidine and ropivacaine for prolonged and walking regional analgesia. Theranostics. 2022; 12(11): 4904-21. https://doi.org/10.7150/thno.74845 PMID: 35836801

25. Li TF, Fan H, Wang YX. Epidural sustained release ropivacaine prolongs anti-allodynia and anti-hyperalgesia in developing and established neuropathic pain. Plos One. 2015; 10(1): e0117321. https://doi.org/10.1371/journal.pone. 0117321 PMID: 25617901

26. Hui MQ, Lu WD, Yu WP, Chen T, Gu JL, Cao YX. Pharmacokinetics and toxicity of oily delivery system of ropivacaine via subcutaneous injection in beagle dogs. J. Pharmacol. Toxicol. 2018; 13(1): 37-44. https://doi.org/10.3923/ jpt.2018.37.44.

27. Das NT, Deshpande C. Effects of intraperitoneal local anaesthetics bupivacaine and ropivacaine versus placebo on postoperative pain after laparoscopic cholecystectomy: a randomised double blind study. J Clin Diagn Res. 2017; 11(7): UC08-UC12. https://doi.org/10.7860/JCDR/2017/26162.10188 PMID: 28893013

28. Karcioglu O, Topacoglu H, Dikme O, Dikme O. A systematic review of the pain scales in adults: Which to use? Am J Emerg Med. 2018; 36(4): 707-14. https://doi.org/10.1016/j.ajem.2018.01.008 PMID: 29321111

29. Lamy E, Fall F, Boigne L, Gromov K, Fabresse N, Grassin-Delyle S. Validation according to european and american regulatory agencies guidelines of an LC-MS/MS method for the quantification of free and total ropivacaine in human plasma. Clin Chem Lab Med. 2020; 58(5): 701-8. https://doi.org/10.1515/cclm-2018-1298 PMID: 30939109

30. Breindahl T, Simonsen O, Andreasen K. Column-switching HPLC-MS/MS analysis of ropivacaine in serum, ultrafiltrate and drainage blood for validating the safety of blood reinfusion. J Chromatogr B Analyt Technol Biomed Life Sci. 2010; 878(1): 76-82. https://doi.org/10.1016/j.jchromb.2009.11.028 PMID: 1995 5023

31. Zhou JZ, Chen ML, Cheng XH, Li Y, Mei XG. Determination of ropivacaine in

rat plasma with liquid chromatography-tandem mass spectrometry. Journal of International Pharmaceutical Research. 2016; 43(4): 736-9. https://doi.org/10. 13220/j. cnki. jipr.2016.04.028.

32. Hansen TG. Ropivacaine: a pharmacological review. Expert Rev Neurother. 2004; 4(5): 781-91. https://doi.org/10.1586/14737175.4.5.781 PMID: 15853505

3~~2~~3. Di BL, Della TF. Side effects to castor oil. Allergy. 2003; 58(8): 826. https://doi.org/10.1034/j.1398-9995.2003.00221.x PMID: 12859571

3~~3~~4. Final report on the safety assessment of ricinus communis (castor) seed oil, hydrogenated castor oil, glyceryl ricinoleate, glyceryl ricinoleate se, ricinoleic acid, potassium ricinoleate, sodium ricinoleate, zinc ricinoleate, cetyl ricinoleate, ethyl ricinoleate, glycol ricinoleate, isopropyl ricinoleate, methyl ricinoleate, and octyldodecyl ricinoleate. Int J Toxicol. 2007; 26 Suppl 3: 31-77. https://doi.org/10.1080/10915810701663150 PMID: 18080873

35. Deckers N, Ruigrok CA, Verhoeve HP, Lourens N. Comparison of pain response after subcutaneous injection of two maropitant formulations to beagle dogs. Veterinary Record Open. 2018; 5(1): e000262. https://doi.org/10.1136/vetreco-2017-000262 PMID: 29531751

36. Jacob S, VanDaele MA, Brown JN. Treatment of demodex-associated inflammatory skin conditions: a systematic review. Dermatol Ther. 2019; 32(6): e13103. https://doi.org/10.1111/dth.13103 PMID: 31583801

37. Araujo F, Fenton TR, Lukmanji S, Raman M. Olive oil and soybean oil based intravenous lipid emulsions, liver biochemistry and clinical outcomes. Nutrients. 2018;10(6): 658. https://doi.org/10.3390/nu10060658 PMID: 29789518

38. Casati A, Vinciguerra F, Santorsola R, Aldegheri G, Putzu M, Fanelli G. Sciatic nerve block with 0.5% levobupivacaine, 0.75% levobupivacaine or 0.75% ropivacaine: a double-blind, randomized comparison. Eur J Anaesthesiol. 2005; 22(6): 452-6. https://doi.org/10.10 17/s0265021505000773 PMID: 15991509

3~~8~~9. Kuthiala G, Chaudhary G. Ropivacaine: a review of its pharmacology and clinical use. Indian Journal of Anaesthesia. 2017; 55(2): 104-10. https://doi.org/

Formatted: No underline

Formatted: Indent: Left: 0", Hanging: 2 ch, First line: -2 ch

10.4103/ 0019-5049.79875 PMID: 21712863

3940. Marhofer P, Oismüller C, Faryniak B, Sitzwohl C, Mayer N, Kapral S. Three-in-one blocks with ropivacaine: evaluation of sensory onset time and quality of sensory block. Anesth Analg. 2000; 90(1): 125-8. https://doi.org/–10.1097/ 00000539-200001000-00028 PMID: 10624992

401. Lu WD, Hui MQ, Gu JL, Liu L, Wu ML, Yang Y, et al. Pharmacodynamics, toxicology and toxicokinetics of ropivacaine oil delivery depot. BMC Anesthesiol. 2022; 22(1): 113. https://doi.org/10.1186/s12871-022-01653-1. PMID: 35448955

412. Griffiths JD, Le NV, Grant S, Bjorksten A, Hebbard P, Royse C. Symptomatic local anaesthetic toxicity and plasma ropivacaine concentrations after transversus abdominis plane block for Caesarean section. Br J Anaesth. 2013; 110(6): 996-1000. https://doi.org/10.1093/bja/aet015 PMID: 23454825

423. Kalicharan RW, Oussoren C, Schot P, de Rijk E, Vromans H. The contribution of the in-vivo fate of an oil depot to drug absorption. Int J Pharm. 2017; 528(1-2): 595-601. https://doi.org/10.1016/j.ijpharm.2017.06.055 PMID: 28629984

434. Kalicharan RW, Baron P, Oussoren C, Bartels LW, Vromans H. Spatial distribution of oil depots monitored in human muscle using MRI. Int J Pharm. 2016; 505(1-2): 52-60. https://doi.org/10.1016/j.ijpharm.2016.03.064 PMID: 27 041126

Larsen DH, Fredholt K, Larsen C. Assessment of rate of drug release from oilvehicle using a rotating dialysis cell. Eur J Pharm Sci. 2000;11(3):223-9. https://doi.org/10.1016/s0928-0987(00)00105-6PMID: 11042228.

Larsen DB, Joergensen S, Olsen NV, Hansen SH, Larsen C. In vivo release of bupivacaine from subcutaneously administered oily solution. Comparison with in vitro release. J Control Release. 2002;17;81(1-2):145-54. https://doi.org/10.1016/s0168-3659 (02) 00055-xPMID: 11992687

Formatted: Indent: Left: 0", Hanging: 2 ch, First line: -2 ch, No bullets or numbering

Table 1. The levels of lLocal adverse event classifications.

Level	Skin reaction
0	No irritation was observed
1	A slight erythema can just be observed
2	Obvious erythema, mild edema or papules were observed
3	Erythema and papules are prominent
4	Edema was significantly
5	Erythema, edema and papules

Table 2. – Liquid chromatography tandem-mass spectrometryLC-MS/MS quantitative liquid phase analysis conditions.

/	Conditions		
Instrument	Agilent 1100 High phase liquid chromatograph (degassing machine G1322A, binary pump G1312A, column temperature chamber G1316A)		
	Automatic sampler (CTC Analytics HTC PAL)		
	Chromatographic column (Hypersil GOLD, 2.1 *50 mm, 5 µm)		
Mobile phase	A: Acetonitrile-water-formic acid (10:90:0.2, v/v/v) （10:90:0.2, v/v/v）		
	B: Acetonitrile-water-formic acid (90:10:0.2, v/v/v) （90:10:0.2, v/v/v）		
Gradient elution condition	Time (min) （min）	A%	B%
	0.00	90.0	10.0
	0.50	90.0	10.0
	0.70	5.0	95.0
	2.20	5.0	95.0
	2.21	0.0	100.0
	2.60	0.0	100.0
	2.61	90.0	10.0
	4.00	90.0	10.0

LC-MS/MS, Liquid chromatography tandem-mass spectrometry.

Table 3. Demographics and baseline characteristics (~~M~~mean±SD).

	Study 1	Study 2						
Group	Ropivacaine oily delivery depot~~ODDDose test~~	Ropivacaine hydrochloride injection~~RHI 150 mg~~	~~ROD 150 mg~~	Ropivacaine oily delivery depot~~ODD~~				
Dose (mg)	12~30 ~~mg~~	150 ~~mg~~	~~150 mg~~	150 ~~mg~~	230 ~~mg~~	300 ~~mg~~	350 ~~mg~~	400 ~~mg~~
n	3	3	~~6~~	6	8	8	8	8
Age Average	28.7±4.1	28.3±2.9	~~26.7±6.3~~	26.7±6.3	28.1±8.1	31.3±10.0	26.3±4.5	33.9±5.1

Formatted Table

Formatted Table

Group	Study 1	Study 2						
	Ropivacaine oily delivery depotODDDose test	Ropivacaine hydrochloride injectionRHI 150 mg	RODD 150 mg	Ropivacaine oily delivery depotODD				
Dose (mg)	12~30 mg	150 mg	150 mg	150 mg	230 mg	300 mg	350 mg	400 mg
n	3	3	6	6	8	8	8	8
Min, Max	25, 33	25, 30	20, 38	20, 38	19, 43	21, 45	20, 32	28, 43

Formatted Table

Group	Study 1	Study 2						
Group	Ropivacaine oily delivery depotODDDose test	Ropivacaine hydrochloride injectionRHI 150 mg	RODD150mg	Ropivacaine oily delivery depotODD				
Dose (mg)	12~30 mg	150 mg	150mg	150 mg	230 mg	300 mg	350 mg	400 mg
n	3	3	6	6	8	8	8	8
Sex n N(%) Male	2 (66.7)	3 (100.00)	5 (83.33)	5 (83.33)	5 (62.50)	6 (75.00)	6 (75.00)	6 (75.00)

Formatted Table

Formatted: Font: 10.5 pt

	Study 1	Study 2						
Group	Ropivacaine oily delivery depot~~ODDDose test~~	Ropivacaine hydrochloride injection~~RHI 150 mg~~	~~RODD 150 mg~~	Ropivacaine oily delivery depot~~ODD~~				
Dose (mg)	12~30 ~~mg~~	150 ~~mg~~	~~150 mg~~	150 ~~mg~~	230 ~~mg~~	300 ~~mg~~	350 ~~mg~~	400 ~~mg~~
n	3	3	~~6~~	6	8	8	8	8
Female	1 (33.3)	0 (0.0~~0~~)	~~1 (16.67)~~	1 (16.~~6~~7)	3 (37.5~~0~~)	2 (25.0~~0~~)	2 (25.0~~0~~)	2 (25.0~~0~~)

Formatted Table

	Study 1	Study 2						
**Group**	**Ropivacaine oily delivery depot~~ODDDose test~~**	**Ropivacaine hydrochloride injection~~RHI 150 mg~~**	**~~RODD 150 mg~~**	**Ropivacaine oily delivery depot~~ODD~~**				
**Dose (mg)**	**12~30 ~~mg~~**	**150 ~~mg~~**	**~~150 mg~~**	**150 ~~mg~~**	**230 ~~mg~~**	**300 ~~mg~~**	**350 ~~mg~~**	**400 ~~mg~~**
**n**	**3**	**3**	**~~6~~**	**6**	**8**	**8**	**8**	**8**
Nation n (%) Ethnic han	2 (66.7)	3 (100.0~~0~~)	~~6 (100.00)~~	6 (100.~~0~~0)	8 (100.0~~0~~)	6 (75.0~~0~~)	8 (100.0~~0~~)	7 (87.5~~0~~)

Formatted Table

	Study 1	Study 2						
Group	Ropivacaine oily delivery depotODDDose test	Ropivacaine hydrochloride injectionRHI 150 mg	RODD150mg150mg	Ropivacaine oily delivery depotODD				
Dose (mg)	12~30 mg	150 mg		150 mg	230 mg	300 mg	350 mg	400 mg
n	3	3	6	6	8	8	8	8
other	1 (33.3)	0 (0.00)	0 (0.00)	0 (0.00)	0 (0.00)	2 (25.00)	0 (0.00)	1 (12.50)

Formatted Table

Group	Study 1	Study 2						
	Ropivacaine oily delivery depot~~ODDDose test~~	Ropivacaine hydrochloride injection~~RHI 150 mg~~	~~ROD D 150 mg~~	Ropivacaine oily delivery depot~~ODD~~				
Dose (mg)	12~30 ~~mg~~	150 ~~mg~~	~~150 mg~~	150 ~~mg~~	230 ~~mg~~	300 ~~mg~~	350 ~~mg~~	400 ~~mg~~
n	3	3	~~6~~	6	8	8	8	8
High (cm) Average	164.3±8.1	171.3~~3~~±12.2~~2~~	~~171.5±6.0~~	171.5±6.0	169.9~~4~~±8.~~07~~1	166.9~~4~~±10.~~06~~1	166.3~~1~~±7.~~16~~2	168.~~19~~2±9.~~55~~6

Formatted Table

	Min, Max	157.0, 173.0	158.0, 182.0	~~164.0, 179.0~~	164.0, 179.0	157.5, 183.0	150.0, 183.0	157.5, 179.0	148.0, 179.0
Weight (kg)	Average	61.1±4.4	64.8~~3~~±9.8~~0~~	~~67.4±10.6~~	67.4±10.6	65.4~~4~~±11.1~~1~~	62.5~~1~~±10.1~~4~~	61.~~16~~2±10.~~18~~2	63.3~~3~~±8.8~~3~~
	Min, Max	57.7, 66.1	54.9, 74.5	~~55.1, 80.9~~	55.1, 80.9	50.0, 80.5	47.8, 79.4	47.9, 77.4	50.9, 74.4
BMI (kg/m^2)	Average	22.6±1.3	22.0~~0~~±0.5~~0~~	~~22.8±~~	22.8±2.5	22.~~49~~5±2.~~08~~1	22.3~~1~~±~~1.99~~2.0	2~~1.96~~2.0±1.9~~1~~	22.3~~4~~±2.~~09~~1

	Study 1	Study 2						
Group	Ropivacaine oily delivery depotODDDose test	Ropivacaine hydrochloride injectionRHI 150 mg	RODD150 mg	Ropivacaine oily delivery depotODD				
Dose (mg)	12~30 mg	150 mg	150 mg	150 mg	230 mg	300 mg	350 mg	400 mg
n	3	3	6	6	8	8	8	8

2.5

Formatted Table

	Study 1		Study 2						
**Group**	**Ropivacaine oily delivery depot~~ODDDose test~~**		**Ropivacaine hydrochloride injection~~RHI 150 mg~~**	~~**RODD 150 mg**~~	**Ropivacaine oily delivery depot~~ODD~~**				
**Dose (mg)**	**12~30 ~~mg~~**		**150 ~~mg~~**	~~**150 mg**~~	**150 ~~mg~~**	**230 ~~mg~~**	**300 ~~mg~~**	**350 ~~mg~~**	**400 ~~mg~~**
**n**	**3**		**3**	~~**6**~~	**6**	**8**	**8**	**8**	**8**
Min, Max	21.7, 24.1	~~21.7, 24.1~~	21.5, 22.5	~~19.5, 25.6~~	19.5, 25.6	20.2, 25.3	20.0, 25.3	19.3, 24.9	19.4, 24.7

BMI, body mass index; SD, standard deviation; Min, minute; Max, maximum.~~; RODD, ropivacaine oily delivery depot; RHI, ropivacaine hydrochloride injection~~.

Formatted Table

Formatted: Font: Not Bold, No underline, Pattern: Clear (White)

Formatted: Font: 10.5 pt, No underline, Font color: Text 1, Pattern: Clear (White)

Table 4. Analgesic range diameter and analgesic range area under time curve of single single-needle abdominal subcutaneous infiltration ropivacaine oily delivery depot (Mmean±SD, n=3).

Group	Ropivacaine oily delivery depotODD 12 mg RODD 16 mg RODD 20 mg RODD 24 mg RODD 30 mg				
Dose (mg)	12 mg	16 mg	20 mg	24 mg	30 mg
Analgesic range diameter (mm)	31.8±20.8	31.8±20.9	27.4 ±11.6	42.5 ±20.8	46.2±24.3
AUC (h·*mm)	304.24 ±229.889	338.444±52.10	286.687 ±28.959.0	574.182 ±227.62	572.81 ±55.67

AUC, area under time –curve; SD, standard deviation; RODD, ropivacaine oily delivery depot.

Formatted: Font: (Default) Times New Roman, Bold, Font color: Text 1

Formatted Table

Formatted: Font: 10.5 pt

Table 5. Circumferential distribution of ropivacaine oily delivery depot~~RODD~~ infiltrated by ~~multiple~~ multiple-~~point~~needles ~~on~~ in the abdomens of the subjects.

**Group**	**Ropivacaine hydrochloride injection~~HI 150 mg (17mL)~~**	**Ropivacaine oily delivery depot~~ODD 150 mg (7.5mL) RODD 230 mg (11.5mL) RODD 300 mg (15mL) RODD 350 mg (17.5mL) RODD 400 mg (20mL)~~**				
**Dose (mg)** ~~**Volum(mL)**~~	**150** ~~**mg (17 mL)**~~	**150** ~~**mg (7.5 mL)**~~	**230** ~~**mg (11.5 mL)**~~	**300** ~~**mg (15 mL)**~~	**350** ~~**mg (17.5 mL)**~~	**400** ~~**mg (20 mL)**~~
**Volum (mL)**	**17**	**7.5**	**11.5**	**15**	**17.5**	**20**
**n**	**3**	**6**	**8**	**8**	**8**	**8**
Circle 1[1], Circle 2[1]	1.2 mL×~~6~~5	1.2 mL×2	1.2 mL×3	1.2 mL×4	1.2mL×5	1.2mL×6
Circle 3	1.2 mL×~~4~~4, 0.~~38~~2 mL×1	1.2 mL×2, 0.3 mL×1	1.2 mL×3, 0.7 mL×1	1.2 mL×4, 0.6 mL×1	1.2 mL×4, 0.7 mL×1	1.2 mL×~~6~~4, 0.8 mL×1

RODD, ropivacaine oily delivery depot; RHI, ropivacaine hydrochloride injection. 150 mg RHI, n=3; 150 mg RODD, n=6; 230~400 mg RODD, n=8, respectively.

Note[1], RODD or RHI given on circumference 1 and 2, respectively. ~~RODD, ropivacaine oily delivery depot; RHI, ropivacaine hydrochloride injection. RHI 150 mg, n=3; RODD 150 mg, n=36; RODD 230 mg~400 mg, n=8, respectively.~~

Formatted: Font: Bold
Formatted Table
Formatted: Font: Bold, No underline
Formatted: Font: 10.5 pt
Formatted: Font: 10.5 pt
Formatted: Font: Bold
Formatted: Font: Bold
Formatted: Font: Bold
Formatted: Font: Bold
Formatted: Font: Bold
Formatted: Font: Bold
Formatted: Font: Bold
Formatted: Font: 10.5 pt
Formatted: Font: 10.5 pt, Not Highlight

Table 6. Incidence of ropivacaine oily delivery depot-related adverse events ~~after administration(n=3~8)~~.

Group ~~(n, %)~~	Ropivacaine hydrochloride injection~~HI 150 mg~~	Ropivacaine oily delivery depot~~ODD 150 mg RODD 230 mg RODD 300 mg RODD 350 mg RODD 400 mg~~				
Dose (mg)	150 ~~mg~~	150 ~~mg~~	230 ~~mg~~	300 ~~mg~~	350 ~~mg~~	400 ~~mg~~
n	3	6	8	8	8	8
Erythema at injection site	2	4	2	5	1	3
Papules at the injection site	0	2	0	5	0	0
Hypertriglyceridemia	1	4	1	1	1	3
Hypotension	0	1	4	1	2	1
Abnormal T wave of ECG	0	0	1	2	0	1
Fervescence	0	0	0	1	0	1
ECG ST segment depression	0	0	0	1	0	0
Abnormal ST segment of ECG	0	0	0	0	0	1
Changes in ST-T segment of ECG	0	0	0	0	0	1
Total	3	5	5	7	5	7

ECG, electrocardiogram~~; RODD, ropivacaine oily delivery depot; RHI, ropivacaine hydrochloride injection~~.

Formatted: Font: 11 pt
Formatted: Font: 11 pt
Formatted: Font: 11 pt
Formatted: Font: 11 pt
Formatted: Font: 11 pt
Formatted: Font: 11 pt
Formatted: Font: 11 pt
Formatted: Font: 11 pt
Formatted: Font: 11 pt
Formatted: Font: 11 pt
Formatted: Font: 11 pt
Formatted: Font: (Default) Times New Roman
Formatted: Left

Table 7. Pharmacokinetic parameters for ropivacaine oily delivery depot after subcutaneous increasing dose infiltration in the abdomens of healthy subjects (Mmean±SD, n=3~8).

Group	RHI150mg	Ropivacaine hydrochloride injectionHI	Ropivacaine oily delivery depot RODD 150mg RODD 230mg RODD 300mg RODD 350mg RODD 400mg				
Dose(mg)		150 mg	150 mg	230 mg	300 mg	350 mg	400 mg
n		3	6	8	8	8	8
C_{max} (μg·mL^{-1})	0.58±	0.58±0.13	0.35±0.09*	0.50±0.09	0.74±0.18	0.80±0.12**	0.86±0.24**

$AUC_{0\text{-}t}$ (h·µg·mL^{-1})	0.135.27±	5.2733±0.5866	6.1622±1.8599	8.63±1.98	10.7988±2.31	12.31±1.72	15.2533±3.74
$AUC_{0\text{-}\infty}$ (h·µg·mL^{-1})	0.585.35±	5.3544±6.7988	6.1922±1.8599	8.6877±2.00	10.85±2.31	12.4335±1.73	15.30±3.74
T_{max} (h)	6.790.7	0.77 ±0.06	8.20 ±5.8599**	10.41 ±5.62**	3.63± 1.98**	8.44± 4.68**	11.81 ±4.5866**

	7±0.06						
$t_{1/2}$ (h)	7.55±5.36	7.556 ±5.364	3.40 ±0.96	3.19520 ±1.13	3.74± 1.12	2.697 ±0.65	3.596±0.80
~~V_d (L)~~	~~290.6±185.8~~	~~290.6± 185.8~~	~~126.6± 55.8~~**	~~124.0± 37.8~~**	~~152.3± 48.0~~*	~~112.7± 35.3~~**	~~143.6± 48.8~~**

Kel (h^{-1})	~~0.17±0.18~~	0.17 ±0.18	0.22± 0.06*	0.24 ±0.08*	0.20 ±0.06*	0.27±0.06**	0.20± 0.05*
CL (L·h^{-1})	~~28.32±3.53~~	28.3 ±3.5	25.8~~3~~ ±6.6~~4~~	27.9~~8~~ ±7.4~~3~~	28.6~~4~~± 5.~~4~~5	28.8~~2~~± 4.~~06~~1	27.~~75~~8± 7.~~76~~8

C_{max}, maximum concentration; AUC, area under curve; T_{max}, time of maximum concentration; $t_{1/2}$, half-life;~~ V_d, apparent volume of distribution;~~ Kel,elimination rate constant; CL, clearance~~; RODD, ropivacaine oily delivery depot; RHI, ropivacaine hydrochloride injection~~.

~~Compared with RHI. *P<0.05, **P<0.01.~~

Formatted: No underline, Highlight

Table 8. ~~Analgesic onset, maintenance time,~~ N~~n~~erve block ~~intensity and distance~~effects ~~between two infiltration sites~~ points ~~of multiple~~ multiple~~-needles in abdominal subcutaneous infiltration~~of ropivacaine oily delivery depot~~RODD~~ ~~(n=3~8)~~.

Group		~~r~~Ropivacaine oily delivery depot~~RODD 150 mg~~ ~~RODD 230 mg~~ ~~RODD 300 mg~~ ~~RODD 350 mg~~ ~~RODD 400 mg~~				
Dose (mg)		150 ~~mg~~	230 ~~mg~~	300 ~~mg~~	350 ~~mg~~	400 ~~mg~~
n		6	8	8	8	8
Onset time (h)[1]	Center of the circle	0.87 (0.43,8.1~~3~~)	0.5 (0.47,0.55)	0.46 (0.38,0.48)	0.47 (0.42,0.63)	0.42 (0.38,0.42)
	1cm from the center of the circle	2.~~36~~4 (0.4~~3~~,4.~~19~~2)	0.5 (0.47,0.55)	0.46 (0.38,0.48)	0.47 (0.40,0.63)	0.42 (0.38,0.47)
	3cm from the center of the circle	0.57 (0.45,4.~~18~~4)	0.5~~0~~ (0.46,0.55)	0.47 (0.42,0.52)	0.78 (0.40,4.18)	0.42 (0.38,0.47)
	4cm from the center of the circle	0.54 (0.45,1.~~18~~2)	0.53 (0.47,4.~~07~~1)	0.82 (0.42,4.0~~3~~)	1.13 (0.42,4.~~15~~2)	0.42 (0.40,1.0~~3~~)

Formatted: Font: Bold

	Center of the circle	31.2~~4~~ (9.02,67.9~~8~~)	47.5 (22.0~~2~~,6~~1.98~~2.0)	52.7~~2~~ (35.6~~3~~,71.6~~0~~)	26.57 (20.9~~7~~,35.~~6~~7)	35.73 (26.7~~2~~,47.9~~3~~)
Maintenance time (h)[1]	1cm from the center of the circle	54.7~~2~~ (13.7~~0~~,67.9~~3~~)	47.~~58~~6 (34.9~~5~~,6~~7.9~~8.0)	71.3~~2~~ (29.7~~2~~,71.~~58~~6)	41.54 (19.9~~3~~,71.~~55~~6)	35.79 (31.9~~8~~,47.7~~3~~)
	3cm from the center of the circle	37.88 (16.9~~7~~,71.6~~0~~)	47.~~68~~7 (47.~~57~~6,71.5~~3~~)	33.8~~3~~ (23.0~~3~~,47.~~57~~6)	43.61 (20.~~6~~7,67.9~~7~~)	47.71 (29.~~7~~8,71.~~68~~7)
	4cm from the center of the circle	69.~~47~~5 (23.1~~0~~,71.~~58~~6)	47.~~68~~7 (35.7~~2~~,67.9~~7~~)	47.7~~1~~ (34.9~~2~~,67.9~~2~~)	35.68 (29.~~55~~6,63.9~~3~~)	47.43 (26.7~~2~~,71.7~~0~~)
Nerve block intensity[1]	Level 1: 5 > analgesic intensity score > 4	86.1~~2~~ %	77.5~~0~~ %	67.2~~2~~ %	69.2~~3~~ %	57.1~~4~~ %
	Level 2: 6 > analgesic intensity score ≥ 5	10.3~~2~~ %	19.5~~0~~ %	29.~~48~~5 %	29.~~7~~8 %	40.~~16~~2 %
	Level 3: analgesic intensity score ≥ 6	3.~~5~~6 %	3.0~~0~~ %	3.30 %	0.99 %	2.7~~0~~ %
Distance between two infiltration points (mm)[2]		40.9±1.3	35.2~~3~~±2.2	29.6±2.3	24.0±1.9	21.5±1.5

Note [1], Median, 95% , CI; Note [2], ~~M~~mean±SD~~, SD, standard deviation; RODD, ropivacaine oily delivery depot.~~

Formatted: Superscript
